# ML216 Prevents DNA Damage-Induced Senescence by Modulating DBC1–BLM Interaction

**DOI:** 10.3390/cells12010145

**Published:** 2022-12-29

**Authors:** Feng Cui, Xueying Han, Xiaoqian Zhang, Siqi Wang, Na Liang, Qing Tan, Wuga Sha, Jun Li

**Affiliations:** State Key Laboratory of Medical Molecular Biology, Department of Biochemistry and Molecular Biology, Institute of Basic Medical Sciences, Chinese Academy of Medical Sciences and Peking Union Medical College, Beijing 100005, China

**Keywords:** senescence, DBC1, BLM, fibrosis, ML216

## Abstract

DNA damage is the major cause of senescence and apoptosis; however, the manner by which DNA-damaged cells become senescent remains unclear. We demonstrate that DNA damage leads to a greater level of senescence rather than apoptosis in DBC1-deficient cells. In addition, we show that BLM becomes degraded during DNA damage, which induces p21 expression and senescence. DBC1 binds to and shields BLM from degradation, thus suppressing senescence. ML216 promotes DBC1–BLM interaction, which aids in the preservation of BLM following DNA damage and suppresses senescence. ML216 enhances pulmonary function by lowering the levels of senescence and fibrosis in both aged mice and a mouse model of bleomycin-induced idiopathic pulmonary fibrosis. Our data reveal a unique mechanism preventing DNA-damaged cells from becoming senescent, which may be regulated by the use of ML216 as a potential treatment for senescence-related diseases.

## 1. Introduction

Senescent cells accumulate in various tissues and organs with aging [1,2], impairing tissue structure, function and regeneration [3,4]. Cellular senescence contributes to the decline in physiological functions and increases the risk of age-related diseases in aged organisms [5,6,7]. Clearance of senescent cells using genetic or pharmacological approaches has been shown to attenuate age-related dysfunctions and improve the lifespan and/or health span of both naturally aged mice and mouse models of age-related diseases [7,8,9,10,11,12]. Currently available intervention strategies targeting senescence include senolytics, which induce apoptosis in senescent cells [13,14], and senomorphics, which suppress the secretory phenotypes of senescent cells [15,16]. While promising, these strategies are flawed by the lack of specificity and targeting pathways required in healthy cells, potentially causing tissues damage [17,18]; therefore, improving the precision with which senescent cells are targeted has become a primary concern.

DNA damage is the major cause of senescence [19]. To preserve tissue integrity, cells possessing unresolved DNA damage may initiate apoptosis or senescence via different pathways [19,20]. Interestingly, DNA-damaged cells in young organisms appear to favor apoptosis, which results in a lower number of senescent cells than that seen in aged organisms [21]. This is thought to be due to efficient DNA damage repair in younger organisms [22,23]; nevertheless, in cells that cannot be completely repaired, there exists no evidence of DNA damage severity distinguishing between the senescence and apoptosis pathways. Therefore, the mechanism underlying the processing of DNA-damaged cells to avert senescence in young organisms, and the reason why this mechanism becomes less efficient with age, remains largely unknown. Understanding this mechanism will aid in the development of targeted strategies to prevent DNA-damaged cells from entering senescence without harming healthy cells. 

Deleted breast cancer 1 (DBC1) is known to inhibit various DNA-damage repair proteins, such as SIRT1, PARP1, BRCA1and HDAC3 [24,25,26]. DNA damage can trigger apoptosis or senescence, both of which may be influenced by DBC1. DBC1 ablation has been shown to protect adipose tissue from entering senescence in obese mice [27], in addition to arresting cells in the G0 phase by prevent cell cycle progression [28], which suggests a confounding role for DBC1 in senescence. BLM, known as the Bloom syndrome protein, is a member of the RecQ DNA helicase family and functions as a key DNA repair protein in the homologous recombination pathway for the repair of double-stranded DNA breaks [29]. Mutations in the BLM gene are the major cause of Bloom syndrome, a rare autosomal recessive genetic disorder characterized by typical premature aging phenotypes [30]. ML216 is a selective inhibitor of helicase activity of BLM and plays an antiproliferative role [31]. In the present study, we demonstrated that DBC1 reduces DNA damage-induced senescence by binding to and protecting BLM from degradation. Moreover, ML216 promotes DBC1–BLM interaction, thereby decreasing DNA damage-induced senescence and fibrosis in both naturally aged mice and an idiopathic pulmonary fibrosis (IPF) mouse model. This demonstrates a potentially novel strategy for specifically reducing the levels of DNA damage-induced senescent cells.

## 2. Materials and Methods

### 2.1. Chemical Reagents and Antibodies

Cisplatin (HY-17394), Z-VAD-FMK (HY-16658), doxorubicin (HY-15142), and staurosporine (62996-74-1) were purchased from MCE (Middlesex, NJ, USA). ML216 was purchased from MCE (HY-12342) and Selleck (S0469, Houston, TX, USA). Hydroxyurea (V900323) and etoposide (E1383) were purchased from Sigma (St. Louis, MO, USA). The antibodies used included anti-DBC1 (Bethyl Labotatories, Montgomery, TX, USA, A300-434A), anti-V5 (Invitrogen, Waltham, MA, USA, R960-25), anti-Flag (Sigma, F7425), anti-BLM (Bethyl Labotatories, A300-110A), anti-GAPDH (Millipore, St. Louis, MO, USA, MAB374), anti-p21 (BD Biosciences, San Jose, CA, USA, 556430), anti-β Tubulin (Santa Cruz, Dallas, TX, USA, SC-166729), anti-p53 (Santa Cruz, SC-126), anti-Bcl-2 (Santa Cruz, SC-7382), anti-p16 (Santa Cruz, SC-56330), anti-phospho-H2A.X (Millipore, 07-164-25UG), anti-β-actin (Absin, Shanghai, China, ABS830031), anti-PARP1 (CST, Danvers, MA, USA, 9542S), anti-cleaved PARP1 (CST, 5625S), anti-BLM (Bioss, Beijing, China, bs-12872R) and anti-p21 (Bioss, bs-0741R). Secondary antibodies were horseradish peroxidase-coupled sheep anti-mouse IgG (GE Healthcare, Chicago, IL, USA, NA931 or ZSGB-BIO, Beijing, China, ZB-2301), donkey anti-rabbit IgG (GE Healthcare, NA934), and horseradish peroxidase-coupled sheep anti-mouse IgG (ZSGB-BIO, ZB-2305).

### 2.2. Transfection and Infection

The 293T cells (The Cell Resource Center, Peking Union Medical College, Beijing, China) were maintained in DMEM (Corning, New York, NYC, USA, 10-013-cv) supplemented with 10% FBS (Thermo, Waltham, MA, USA, 10091148), and 1% penicillin/streptomycin. The IMR-90 cells were maintained in MEM (Hyclone, Logan, UT, USA, SH30024.01) supplemented with 10% FBS, 1% penicillin/streptomycin, 1% non-essential amino acid solution, and 1% sodium pyruvate. All plasmids were constructed in the lab unless otherwise specifically indicated. The plasmid sets of pRS-BLM (Origene, Rockville, MD, USA, TR314471) and pRS-DBC1 (Origene, TR303704) were purchased from Origene. For retrovirus production, the target plasmids were transfected into 293T cells with the packaging plasmids VSV-G, Gag-Pol using the FuGENE HD transfection reagent (Promega, Madison, WI, USA, PRE2311). The virus-containing media was harvested between 48 h and 72 h post-transfection and filtered through a 0.45 mm filter. The cells were infected by the filtered media in the presence of 8 μg/mL polybrene (Sigma, H9268). After a 48 h incubation, stably transfected cells were selected with 2 μg/mL puromycin. Transient transfection was performed using Lipofectamine 2000 (Thermo, 11668019), according to the manufacturer’s instructions. 

### 2.3. Immunoprecipitation (IP)

For immunoprecipitation (IP), whole cell lysates were incubated with anti-FLAG M2 affinity gel (Sigma, A2220), anti-V5 antibody agarose affinity gel (Sigma, A7345) or Protein G PLUS/Protein A-agarose mixture (Sigma, IP05) and the respective antibody overnight. Then, the beads were washed three times using Triton X-100 lysis buffer, and proteins were eluted with 2×SDS loading buffer. Then, the proteins were run on an SDS-PAGE gel and probed with antibodies. Secondary antibodies used were the TrueBlot Anti-Rabbit IgG HRP (Rockland, Montgomery, PA, USA, 18881633), and the TrueBlot ULTRA Anti-Mouse Ig HRP (Rockland, 18881733). 

The In vitro IP was conducted as follows: Lysates of 293T cells transfected with pMSCV-Flag-DBC1 were divided into four equal aliquots. The four aliquots were incubated with anti-FLAG M2 affinity gel together with 0, 20, 50, or 100 μM ML216, respectively, followed by Western blotting for BLM. 

### 2.4. RT-PCR and RNA Sequencing

RNA was isolated with the E.Z.N.A. Total RNA Kit II (Omega, Norcross, GA, USA, R6934-02), according to the manufacturer’s instructions. cDNA was synthesized using the iScript cDNA Synthesis Kit (Bio-Rad, Hercules, CA, 1708891). RT-PCR was performed with the PowerUp SYBR Green Master Mix (Applied Biosystems, Waltham, MA, USA, A25742). The primers used in this study are shown in Table S2. The preparation of an RNA library and transcriptome sequencing was conducted by Novogene (Beijing, China). The gene ontology (GO) biological process terms (biological process) and The Kyoto Encyclopedia of Genes and Genomes (KEGG) pathway terms were determined using the Database for Annotation, Visualization and Integrated Discovery (DAVID; https://david.abcc.ncifcrf.gov/) (accessed on 10 October 2020).

### 2.5. HR Repair Assays Design and Procedure

To construct the pcDNA3.1-HR plasmid, I-Sce I fragment was inserted into the full GFP sequence (EGFP), and the EGFP and iEGFP (truncated GFP) were cloned into pcDNA 3.1(-) in turn. The above pcDNA3.1-HR plasmid, pCBASceI plasmid (Addgene, Watertown, MA, USA, 26477) and dsRed2-N1 plasmid (Addgene, 54493) were transfected into the cells using Lipofectamine 2000, according to the manufacturer’s instructions. Drug treatment was conducted 48 h after transfection, followed by analysis using the BD Accuri C6, BD Accuri C6 plus (BD Biosciences) and Flowjo VX10 (Flowjo X. 10.0.7, Flowjo, San Jose, CA, USA). The ratio of GFP-positive cells to RFP-positive cells was used to calculate the HR repair efficiency.

### 2.6. Cell Viability Assay

Cell viability was assessed using the Cell Counting Kit-8 (Beijing Jude Antau Co., JD226 or Vazyme Biotech Co., A311, Beijing, China). Briefly, after drug treatment, cells were added to a 96-well plate with 10 μl cck8 solution at corresponding time points. After incubating for 1 h, the absorbance (450 nm) was measured by the FlexStation 3 Multi-Mode Microplate Reader (Molecular Devices, Silicon Valley, CA, USA) or the SynergyH1 Multi-Mode Microplate Reader (Biotek, Winooski, VT, USA).

### 2.7. Apoptosis Assay

Apoptosis was assessed by using the Annexin V/ PI kit (BD Biosciences, 556547). After drug treatment, the cells (1 × 10^5^ cells) were washed twice using PBS and resuspended. The cells were stained with FITC Annexin V and PI for 15 min at room temperature. Then, the stained cells were measured using the BD Accuri C6 (BD Biosciences ) and data were analyzed using Flowjo VX10 (Flowjo X. 10.0.7, Flowjo, San Jose, CA, USA).

### 2.8. DNA Damage-Induced Senescence

Early passage IMR-90 cells at passage 16 (Cell Bank/Stem Cell Bank, Chinese Academy of Sciences, Beijing, China) were treated with etoposide (20 μM) for 24 h. The cells were replaced with fresh medium after 24 h, and allowed to continue to grow for 7 days with frequent medium changes to develop senescence.

### 2.9. Immunofluorescence Staining 

The cells grown on coverslips were fixed in 4% paraformaldehyde for 30 min, followed by three 5 min washes in PBS. After washing, the cells were blocked with 1% BSA in PBST (PBS + 0.1% Tween 20) for 30 min and incubated with anti-Ki67 antibody (Abcam, Cambridge, UK, AB15580, 1:1000) in 1% BSA in PBST at 4 °C overnight. The cells were washed three times in PBST for 5 min and then incubated with the secondary antibody (Alexa Fluor^®^ 568 antibody, Abcam, AB150113, 1:500) in 1% BSA for 1 h at room temperature in the dark. To counterstain the nuclei, the coverslips were mounted with mounting medium containing DAPI (Abcam, AB104139). Images were taken under an inverted fluorescence microscope (Nikon Eclipse Ts2, Minato-ku, Tokyo, Japan). At least 200 cells per sample were counted.

### 2.10. Mouse Model and Drug Treatment

All C57BL/6J mice were purchased from SPF biotechnology (Beijing, China). The mice were placed in specific pathogen-free (SPF) barrier facilities and maintained in ventilated cages, with free access to food and water.

Young C57BL/6J mice (male, 6 weeks) were randomly divided into three groups, each group contained 12–15 mice. To induce lung fibrosis, mice from two groups received 2 mg/kg bleomycin (MCE, HY-17565) through intratracheal instillation, and one group received PBS as a sham. After one week, the two bleomycin-treated groups received 0.5 mg/kg ML216 dissolved in a solvent containing saline with 10% DMSO, 20% SBE-β-CD (Sigma, H107) or a solvent control through intraperitoneal injections (i.p.), respectively. The injections were conducted twice a week for 3 weeks, while the sham group received the solvent control at the same frequency. Mouse body weight was measured twice a week.

Aged C57BL/6J mice (female, 22 months) were randomly divided into two groups, each group contained 10–11 mice. The two groups received 1 mg/kg ML216 dissolved in a solvent containing saline with 10% DMSO, 20% SBE-β-CD (Sigma, H107) or a solvent control through intraperitoneal injections (i.p.), respectively. The injections were conducted twice a week for 5 weeks. Mouse body weight was measured twice a week.

### 2.11. Muscle Endurance Capacity Analysis

Treadmill exercise was used to evaluate the muscle endurance capacity. Mice ran on a flat motorized treadmill (ZH-PT/5S, Zhenghua biology, Anhui, China), which was set at 10 m/min, followed by 12 m/min, 15 m/min, 18 m/min, 20 m/min for 5 min, and maintained at 22 m/min. Mice were motivated to run until they are unable or unwilling to continue, as indicated by their inability to move after being shocked for 10 s.

### 2.12. Lung Function Analysis

Invasive lung function was measured after 3 weeks of ML216 or control injections. Mice were anaesthetized with 100 mg/kg pentobarbital solution (Merck, Rahway, NJ, USA, P-010) before trachea intubation. The trachea of the mice was connected to a PFT Pulmonary Maneuvers (Buxco, Salt Lake City, UT, USA) by a metallic conduit. FinePointe software (FinePointe PFT, Buxco, Salt Lake City, UT) was used to acquire data, including functional residual capacity (FRC), pressure volume (PV), flow volume (FV), and resistance and compliance (RC). Lung functions were measured three times and averaged for each mouse. Mice were killed by cervical dislocation after parameter collection. After the mice were sacrificed, the left lungs were collected and placed in the center of a mold filled with cryo-embedding media (OCT) and frozen with liquid nitrogen; the right lungs were snap-frozen in liquid nitrogen for later molecular experiments.

### 2.13. Y-Maze Test

The Y-maze test was used to measure spatial learning and memory. Each arm of the Y-maze was 30 cm long, 8 cm wide, 15 cm high (RWD Life Science, Shenzhen, China, 63006). During training, each mouse was placed at the end of one arm of a symmetrical Y-maze and allowed to explore the two arms of the Y-maze for 10 min, while access to the novel arm was prohibited. After 1 h, the barrier in the novel arm was removed. The older mice were free to explore the apparatus for 10 min from the same start arm. Arm entry was recorded when 50% of a mouse’s body entered the arm. Novelty preference [%] was defined as the time spent in the novel arm divided by the time spent in all arms. An alternation was recorded when the mouse had entered the three different arms during the test (e.g., in the sequence 1232123, three alternations were recorded). Spontaneous alternations were defined as the total alternations divided by the total number of arm entries minus 2.

### 2.14. β-Galactosidase Staining for Cells and Lung Tissues

Cells were fixed in G/F fixative mix (50% glutaraldehyde and 37% formaldehyde in PBS) for 5 min at room temperature. After briefly washing twice in PBS, the cells were stained in staining solution (5 mM potassium ferricyanide, 5 mM potassium ferrocyanide, 2 mM MgCl_2_, 150 mM NaCl and 1 mg/mL X-gal (VWR Life Science, Radnor, PA, USA, 0428) in citrate/sodium phosphate buffer (40 Mm, pH 6)) for 2–4 h in a 37 °C, humidified chamber. For tissue staining, the frozen sections were fixed in the ice-cold fixative solution (2% formaldehyde and 0.2% glutaraldehyde in PBS) for 7 min. Then, the samples were washed in ice-cold PBS three times for 10 min each. The frozen sections were encircled by a PAP pen and stained in staining solution in a 37 °C humidified chamber overnight. Images were taken under an inverted fluorescence microscope (Nikon Eclipse Ts2) or an Evos FL Auto 2 (Thermo). For cells, at least 400 cells per sample were counted. For frozen sections, the results of the SA-β-gal-positive area % were calculated by the stained area of SA-β-gal using Image J (NIH).

### 2.15. Hydroxyproline Assay

The collagen content in the lungs was measured by the Hydroxyproline Assay kit (Solarbio, Beijing, China, BC0255), according to the manufacturer’s instructions. The absorbance (560 nm) was measured by the FlexStation 3 Multi-Mode Microplate Reader (Molecular Devices, Silicon Valley, CA, USA) or the SynergyH1 Multi-Mode Microplate Reader (Biotek, Winooski, VT, USA).

### 2.16. Histology

The frozen tissue blocks used for H&E staining or Masson’s trichrome staining were sectioned at 5 μm. H&E staining was conducted by the histology facility of the State Key Laboratory of Medical Molecular Biology or Wuhan Service biotechnology company. Tissue slides were stained with the Masson’s trichrome staining Kit (Solarbio, Beijing, China, G1343), according to the manufacturer’s instructions. Images were taken under an inverted fluorescence microscope (Nikon Eclipse Ts2, Minato-ku, Tokyo, Japan), a Evos FL Auto 2 (Thermo) and an Inverted Microscope Leica DMi8.

### 2.17. Immunohistochemistry

The frozen tissue sections were fixed in pre-cooled formaldehyde fixative solution (85 mM Na_2_HPO4, 75 mM KH_2_PO_4_, 4% paraformaldehyde, pH 6.4) for 10 min (4 °C) and washed three times for 5 min for PBS. Then, the sections were incubated in 0.3% H_2_O_2_ in methanol at room temperature for 10 min to block endogenous peroxidase activity, and then washed three times for 5 min with PBS. After incubating the tissue sections with blocking buffer (5% BSA in PBS) for 1 h at room temperature, the sections were incubated with the BLM antibody (Bioss, bs-12872R) or the P21 antibody (Bioss, bs-0741R) diluted in blocking buffer (1:50 or 1:200) at 4 °C, overnight. After incubation with the secondary antibody diluted in blocking buffer for 1 h (1:100), the sections were washed three times for 5 min with PBS and the DAB substrate solution (Thermo Scientific, #34002) was applied for 5 min. The slides were counterstained by immersing the slides in hematoxylin (Solarbio, G1080) for 15 s, and then rinsing them in tap water for 15 min before mounting. Images were taken under the Evos FL Auto 2 (Thermo).

### 2.18. ELISA Assay

The Mouse CCL2/JE/MCP-1 ELISA Kit (Absin, ABS520016), Mouse CXCL1/KC (IL-8) ELISA KIT (Absin, ABS520017), and the Mouse IL-6 ELISA Kit (Absin, ABS520004) were used for the ELISA assays, according to the manufacturer’s instructions. The absorbance signals (450 nm) were measured by the FlexStation 3 Multi-Mode Microplate Reader (Molecular Devices, America).

### 2.19. Statistical Analysis

Unless specified in the figure, all statistical significance was analyzed using an unpaired Student’s *t* test in GraphPad Prism. The *p* values are denoted in the figures as: ns (not significant), * *p* < 0.05, ** *p* < 0.01, *** *p* < 0.001, **** *p* < 0.0001.

## 3. Results 

### 3.1. DBC1 Deficiency Increases Senescence Levels and Reduces Apoptosis in Response to DNA Damage

**Figure 1:**
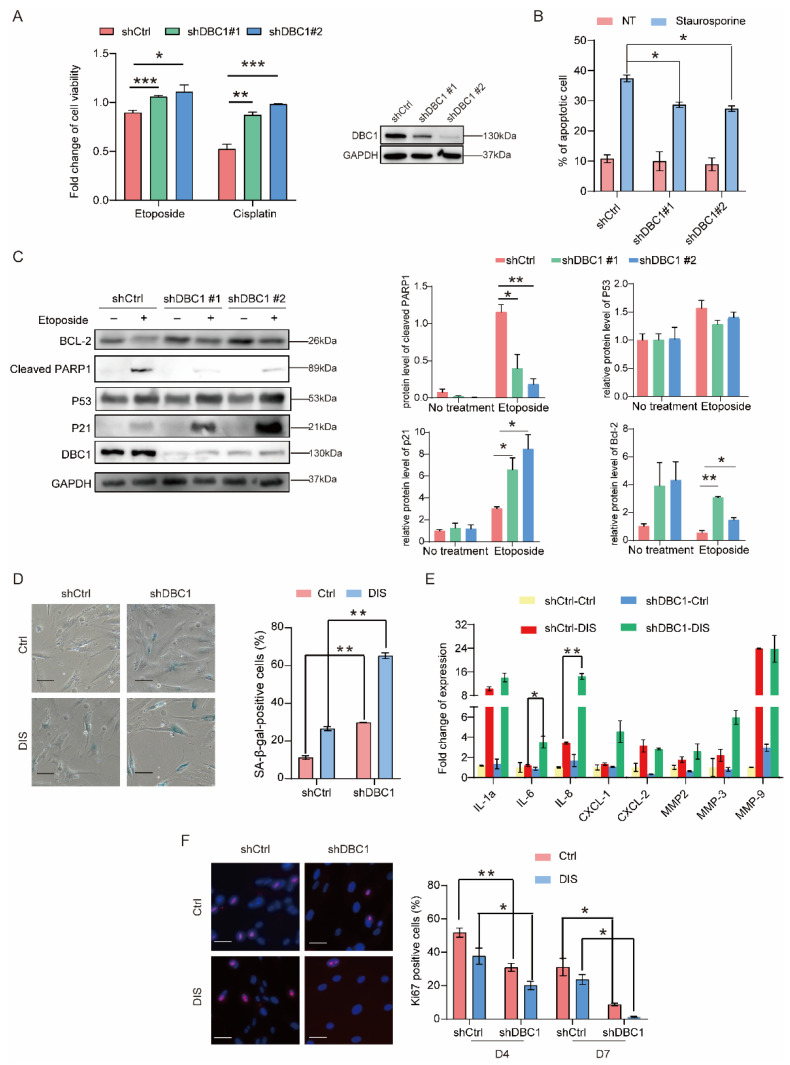
DBC1 deficiency increases senescence levels and reduces apoptosis in response to DNA damage. (**A**) Cell survival analysis of DBC1 knockdown cells treated with etoposide (25 μM, 24 h) or cisplatin (50 μM, 48 h), with the expression levels of DBC1 shown on the right. (**B**) Apoptosis analysis of DBC1 knockdown cells treated with staurosporine (1 μM, 2 h) using Annexin V-FITC staining and flow cytometry for quantification. Representative scatter plots are in the Extended Data (**A**), *n* = 2 biological replicates. (**C**) Western blotting analysis and quantification for BCL-2, cleaved PARP1, p53 and p21 protein levels in DBC1 knockdown cells treated with etoposide (50 μM, 24 h). (**D**) SA-β-gal staining of DBC1 knockdown IMR-90 cells after DNA damage-induced senescence using etoposide or DMSO (see method 2.8 for the experiment procedure). The quantifications of SA-β-gal-positive cells are shown in the right panel. *n* = 3 biological replicates, scale bar = 100 μm. (**E**) Relative gene expression of IL-6, IL-8, IL-1α, CXCL-1, CXCL-2, MMP-2, MMP-3 and MMP-9 in the DBC1 knockdown cells described in (**D**), measured by RT-PCR analysis. (**F**) Immunofluorescence staining of Ki67 positive cells in DBC1 knockdown cells after DNA damage-induced senescence using etoposide or DMSO (see method 2.8 for the experiment procedure). The quantifications are shown in the right panel. Scale bar = 50 μm. All data are presented as mean ± SEM.

Deleted in Breast Cancer 1 (DBC1) is a large multi-domain protein involved various cellular functions, such as DNA damage, cell cycle arrest/progression, and apoptosis [32]. Knocking down DBC1 improved cell survival following DNA damage induced by etoposide or cisplatin (Figure 1A), likely due to a low number of cells undergoing apoptosis Figure 1B and Figure S1A). On the other hand, DBC1 overexpression increased cell death following DNA damage, particularly via apoptosis (Supplementary Figure S1B–D). In DBC1 knockdown cells, DNA damage induced higher expression of apoptosis inhibitor proteins BCL-2 and p21 compared to the control cells, whereas the induction of the pivotal pro-apoptotic factor p53 was not altered by DBC1 deficiency (Figure 1C). Given the elevated p21 induction, we evaluated whether DBC1 deficiency exacerbates senescence using a DNA damage-induced senescence (DIS) model that employs etoposide to induce senescence in IMR-90 cells (see Section 2.8) [14]. The number of senescent cells increased by approximately 2-fold in DBC1 knockdown IMR-90 cells in comparison with the control cells (Figure 1D). The secretion of senescence-associated secretory phenotype (SASP) factors, such as IL-6 and IL-8, was also significantly increased in DBC1 knockdown cells following DNA damage (Figure 1E). As measured by Ki-67 staining, DBC1 deficiency reduced the number of proliferating cells at 4 and 7 days following etoposide-induced DNA damage (Figure 1F), consistent with the increased induction of senescence (Figure 1D). Collectively, these data support the notion that a lack of DBC1 promotes DNA damage-induced senescence and reduces apoptosis.

### 3.2. DBC1 Regulates DNA Damage-Induced Senescence by Maintaining BLM Abundance

**Figure 2:**
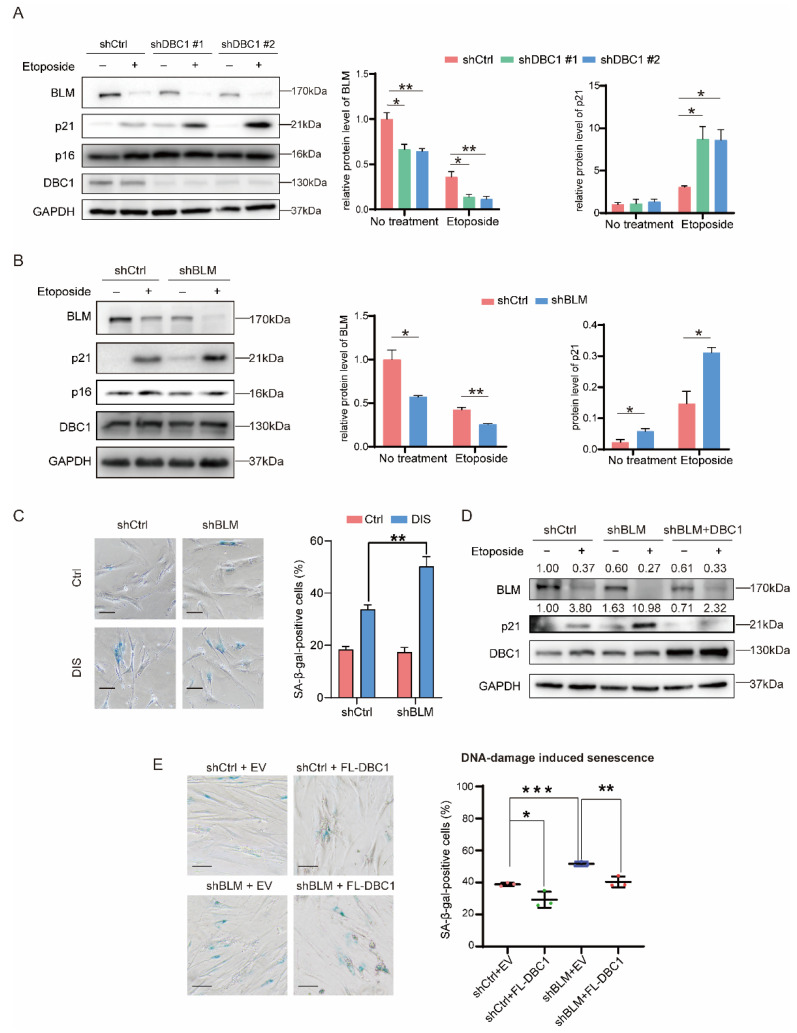
DBC1 regulates DNA damage-induced senescence by maintaining BLM abundance. (**A**) Western blotting analysis and quantification for BLM, p16 and p21 protein levels in DBC1 knockdown cells treated with etoposide (50 μM, 24 h). (**B**) Western blotting analysis and quantification for DBC1, BLM, p16 and p21 protein levels in BLM knockdown cells treated with etoposide (50 μM, 24 h). (**C**) SA-β-gal staining of BLM knockdown IMR-90 cells after DNA damage-induced senescence using etoposide or DMSO (see method 2.8 for the experiment procedure). The quantifications are shown in the right panel, *n* = 4 biological replicates. Scale bar = 100 μm. (**D**) Western blotting analysis for BLM and p21 protein levels in BLM knockdown 293T cells transfected with DBC1 after etoposide-induced DNA damage (50 μM, 24 h). (**E**) Representative images and quantification (*n* = 3 biological replicates) of SA-β-gal staining of BLM knockdown IMR-90 cells transfected with DBC1 after DNA damage-induced senescence using etoposide or DMSO (Control) (see method for the experiment procedure). Scale bar = 100 μm. Data are presented as mean ± SD [E] or SEM [C].

We investigated whether the pathway leading to senescence is influenced by DBC1, and found that p21 expression was highly induced in DBC1 knockdown cells following DNA damage, while p16, another major player in senescence induction [33], was not affected by DBC1 knockdown (Figure 2A). Consistently, p21 induction was lower in DBC1-overexpressing cells following DNA damage (Supplementary Figure S2A). Interestingly, BLM protein level was considerably lower in DBC1 knockdown cells following DNA damage, yet the mRNA of BLM was not significantly affected by DBC1 status (Figure 2A and Figure S2B). BLM levels were reduced following etoposide-induced DNA damage, presumably due to cleavage by activated caspases [34,35]. A lack of DBC1 aggravated this loss of BLM following DNA damage and induced a further increase in p21 expression (Figure 2A), and vice versa (Supplementary Figure S2A). A similar reduction in BLM levels was observed following DNA damage mediated by other reagents such as doxorubicin, hydroxyurea or staurosporine, implying that this is a common event during DNA damage (Supplementary Figure S2C).

During DNA damage p21 is induced to arrest the cell cycle, allowing DNA repair to take place [36]. In comparison with control cells, BLM knockdown cells displayed higher p21 induction (Figure 2B) but unchanged p53 and p16 expression levels (Figure 2B and Figure S2D), similar to that seen in DBC1 knockdown cells (Figure 1C,D). In the DIS model, knocking down BLM in IMR-90 cells generated a greater number of senescent cells (Figure 2C), mirroring the effects of DBC1 knockdown (Figure 1D). We hypothesize that increased senescence induction in DBC1 knockdown cells is due to inefficient preservation of BLM, given the similar senescence induction effects of lacking BLM or DBC1 in response to DNA damage. To test this theory, we overexpressed DBC1 in BLM-deficient HEK293T cells and found that BLM protein levels were preserved and p21 induction was significantly suppressed following DNA damage (Figure 2D). DBC1 overexpression in BLM-deficient IMR-90 cells also reduced the number of SA-β-gal^+^ cells in the DIS model, indicating a low rate of senescence (Figure 2E and Figure S2E). A similar trend was observed in these cells even without the induction of DNA damage: however, the difference was not significant (Supplementary Figure S2F). In DBC1-deficient cells, overexpressing BLM also attenuated the increased p21 induction to a level similar to the knockdown control cells following DNA damage (Supplementary Figure S2G). These data indicate that DBC1 can alleviate the induction of senescence caused by BLM deficiency, which supports our hypothesis that DBC1 regulates DNA damage-induced senescence via BLM. Unlike the DBC1 knockdown cells, which displayed increased BCL-2 expression and decreased apoptosis (Figure 1B,C), the knock down of BLM had no effect on the level of either BCL-2 or apoptosis (Supplementary Figure S2D,H), indicating that BLM primarily mediates the effect of DBC1’s effect on senescence rather than its effect on apoptosis. 

**Figure 3:**
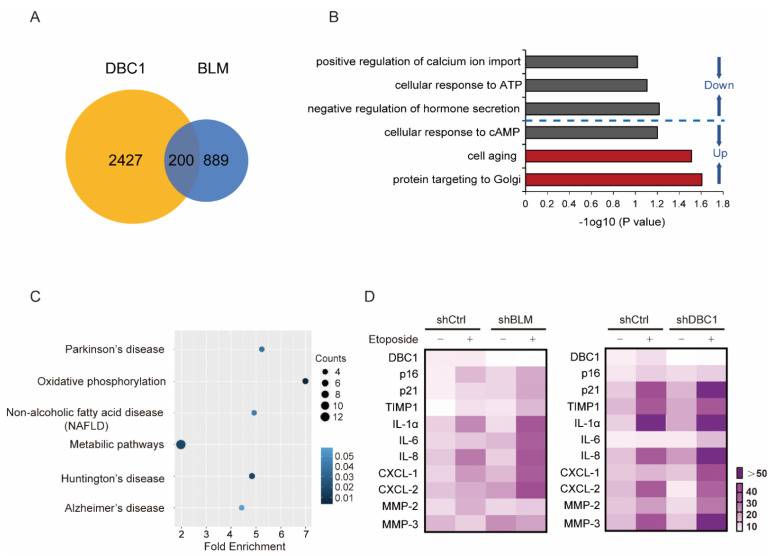
DBC1 and BLM co-regulate the expression of senescence-associated genes. (**A**) The common differentially expressed genes in DBC1 knockdown and BLM knockdown cells presented in the form of a Venn diagram. (**B**) Gene ontological analysis of the common differentially expressed genes in DBC1 knockdown and BLM knockdown cells, performed with –log_10_ (*p* value) plotted as a function of classification meeting a *p* value of < 0.05. (**C**) KEGG enrichment analysis of the common differentially expressed genes in DBC1 knockdown and BLM knockdown cells compared to control cells. *p* value of < 0.05. (**D**) Gene expression changes in senescence-associated genes in DBC1 knockdown or BLM knockdown cells, with or without etoposide-induced DNA damage (50 μM, 24 h).

To further assess the correlation between DBC1 and BLM during senescence induction, we conducted RNA sequencing analysis of both DBC1 knockdown and BLM knockdown cells, which revealed 200 overlapping differentially expressed genes (Figure 3A and Table S1). Subsequent GO enrichment analysis indicated that “cell aging” was one of the most enriched up-regulated pathways shared by the two data sets (Figure 3B). Further KEGG analysis of the commonly regulated genes by both DBC1 and BLM showed that the most enriched pathways were associated with oxidative phosphorylation, Huntington’s disease, metabolic pathways, Parkinson’s disease and Alzheimer’s disease, all of which are either aging-related diseases or contribute to aging when their functions are defective (Figure 3C). In this context, transcriptional analysis comparing the effects of BLM or DBC1 knockdown on the signature senescence-related genes following DNA damage revealed similar patterns (Figure 3D). These findings add to the accumulating evidence supporting the notion that DBC1 and BLM work in concert to regulate the induction of senescence.

### 3.3. DBC1 Binds to BLM to Prevent its Degradation

**Figure 4:**
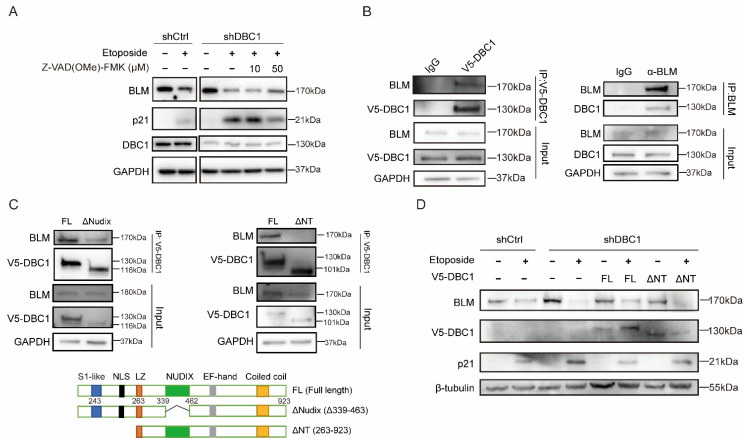
DBC1 binds to BLM to prevent its degradation. (**A**) Western blotting analysis of BLM and p21 protein levels in DBC1 knockdown cells treated with etoposide (100 μM) or Z-VAD-FMK for 24 h. (**B**) The reciprocal co-IP analysis on DBC1 and BLM interaction. (**C**) Co-IP of the full-length or truncated DBC1 with BLM. Schematic structures of full-length and truncated mutants of DBC1 are shown at the bottom. FL represents full-length; ΔNudix represents deletion of the Nudix domain (residues 339–463); ΔNT represents deletion of the N-terminal domain (residues 1–263). (**D**) Western blotting analysis of BLM and p21 protein levels in DBC1 knockdown cells with the reintroduction of the full-length or N-terminal truncated mutant DBC1 (ΔNT) after etoposide-induced DNA damage (50 μM, 24 h).

BLM expression level was less well-preserved in DBC1 knockdown cells following DNA damage, the levels of which could be increased to those comparable to the control cells by incubation with the pan-caspase inhibitor Z-VAD-FMK (Figure 4A), suggesting that DBC1 may reduce caspase-mediated BLM degradation. Moreover, according to reciprocal immunoprecipitation, DBC1 and BLM interact with each other (Figure 4B). A DBC1 mutant lacking the N-terminal 243 amino acid residues failed to interact with BLM, whereas deletion of the Nudix domain, the center portion of DBC1, had no effect on the DBC1–BLM interaction (Figure 4C). The N-terminal domain (1-243 amino acids) of DBC1 was not characterized due to its poor expression profile (data not shown). DBC1 has been reported to bind to and protect p53 from MDM2-mediated degradation [37]; therefore, to explore the possibility that DBC1 is responsible for protecting BLM from degradation via binding, we reintroduced the N-terminal of the truncated DBC1 mutant which does not interact with BLM into DBC1 knockdown cells, and BLM degradation was not affected; however, reintroduction of the full-length DBC1 led to a smaller decrease in BLM levels, similar to those seen in the control cells (Figure 4D). p21 induction caused by DNA damage was also reduced following the reintroduction of full-length DBC1 but not with the non-interacting N-terminal truncated DBC1 (Figure 4D). These results support the concept that DBC1 binds to BLM to protect it from DNA damage-induced degradation. 

### 3.4. ML216 Protects BLM from Degradation by Promoting the DBC1–BLM Interaction and Suppresses DNA Damage-Induced Senescence

**Figure 5:**
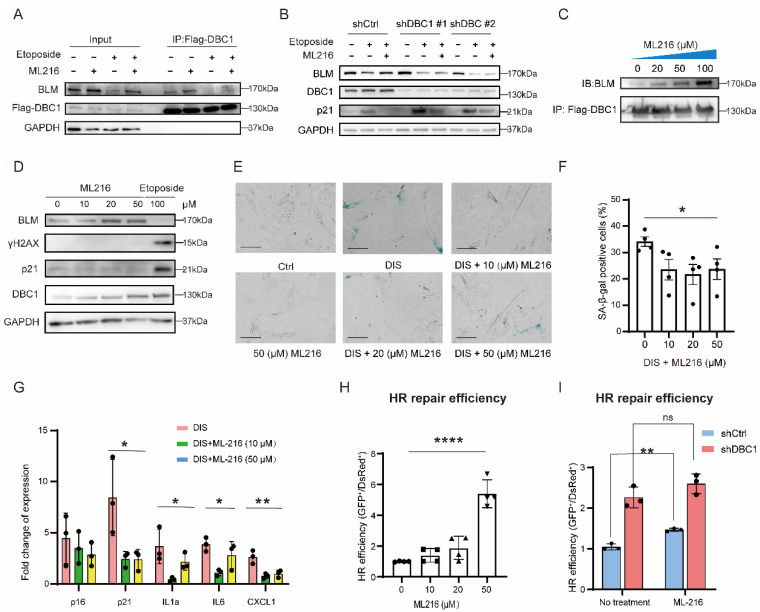
ML216 protects BLM from degradation by promoting DBC1–BLM interaction and suppresses DNA damage-induced senescence. (**A**) Co-IP of Flag-DBC1 and BLM after the treatment with etoposide (100 μM), ML216 (50 μM) or both for 24 h. (**B**) Western blotting analysis of BLM and p21 protein levels in DBC1 knockdown cells treated with etoposide (100 μM), ML216 (50 μM) or both for 24 h. (**C**) In vitro immunoprecipitation of DBC1 with BLM in the presence of different concentrations of ML216. Four equal aliquots of lysates from cells overexpressing Flag-DBC1 were incubated with 0, 20, 50, 100 μM of ML216 for 12 h, followed by immunoprecipitating DBC1 with Flag antibody and the detection of BLM using Western blotting. (see method 2.3 for the experiment procedure). (**D**) Western blotting analysis of BLM, γ-H2AX and p21 protein levels in 293T cells treated with different concentrations of ML216 (24 h); etoposide was used as positive control for DNA damage (100 μM, 24 h). (**E**,**F**) Representative images and quantification of SA-β-gal-positive IMR-90 cells after DNA damage-induced senescence using etoposide or DMSO (see method 2.8 for the experiment procedure). Various concentrations of ML216 were added in cell culture with etoposide as a co-treatment. *n* = 4 biological replicates as indicated by the black dots, Kruskal–Wallis test. Scale bar = 100 μm. (**G**) Gene expression analysis of SASP-related genes p16, p21, IL-1α, IL-6 and CXCL-1 in the IMR-90 cells described in (**E**), measured by RT-PCR, one-way ANOVA test. (**H**) Homologous recombination (HR) repair efficiency of 293T cells treated with various concentrations of ML216 for 24 h, *n* = 4 biological replicates, one-way ANOVA test. (**I**) HR repair efficiency of DBC1 knockdown 293T cells treated with ML216 (50 μM, 24 h), *n* = 4 biological replicates. Data are presented as mean ± SD (**G–I**); Data are presented as mean ± SEM (**F**).

We evaluated whether DBC1–BLM interaction is mediated by the DNA unwinding activity of BLM. Surprisingly, treatment with the BLM inhibitor ML216 led to an increase in BLM protein levels and resulted in more BLM protein being pulled down by Flag-DBC1 in the presence or absence of DNA damage (Figure 5A). Additionally, p21 induction following DNA damage was almost completely suppressed in ML216-treated cells. Nevertheless, both the ML216-mediated changes were diminished in DBC1 knockdown cells, indicating the relevance of DBC1 (Figure 5B). Since ML216 had no effect on BLM transcription (Supplementary Figure S3A), we investigated whether ML216 affected BLM degradation by modulating the DBC1–BLM interaction. To eliminate the interference that ML216 caused by raising cellular BLM levels, we performed in vitro immunoprecipitation (see Section 2) of Flag-DBC1 in the presence of increasing concentrations of ML216. BLM pulled down by Flag-DBC1 displayed a clear ML216 concentration-dependent increase (Figure 5C), suggesting that ML216 enhances DBC1–BLM interaction and may raise cellular BLM levels by protecting BLM from degradation. It is noteworthy that ML216 still increased BLM levels in the absence of DNA damage, implying that the DBC1–BLM interaction may not only prevent caspase-mediated degradation, but also other types of degradation. This idea is supported by the fact that caspase inhibitors only partially reduced BLM loss in DBC1 knockdown cells (Figure 4A). 

Several lines of evidence suggest that ML216 treatment has different effects on cells compared to knocking down BLM. According to the data presented herein and in a previous study [38], ML216 treatment has no effect on senescence markers, such as p21 and γ-H2AX, in the absence of DNA damage (Figure 5D), while BLM knockdown increases the number of senescent cells (Figure 2D). When used in conjunction with the DNA damage reagents etoposide or mitomycin C, ML216 decreased the levels of p21 and γ-H2AX (Supplementary Figure S3B) [31]. Consistently, ML216 significantly reduced the number of senescent IMR-90 cells in the DIS model (Figure 5E,F). Moreover, the expression levels of SASP factors, such as IL-1a, IL-6 and CXCL1, were also reduced by the addition of ML216 (Figure 5G). BLM plays a critical role in the homologous recombination (HR) repair of double-stranded DNA breaks [39]; therefore, we performed an HR reporter assay (Supplementary Figure S3C). BLM deficiency reduced HR repair efficiency (Supplementary Figure S3D), which was increased following ML216 treatment (Figure 5H). This is consistent with BLM levels being increased by ML216 (Figure 5D); and since DBC1 is deficient, ML216 can no longer increase HR repair efficiency (Figure 5I), indicating a dependence on DBC1. Together, these data show that, opposite to knocking down BLM, ML216 treatment decreased DNA damage, reduced senescence induction, and increased HR repair, all of which disagrees with the inhibition of BLM activity but supported the preservation of BLM levels. Persistent p21 induction is responsible for permanent cell cycle arrest and the induction of senescence. In addition to lowering p21 induction (Figure 5B), ML216 also reduced senescence induction in the DIS model (Figure 5E,F). Meanwhile, ML216 facilitated apoptosis following DNA damage (Supplementary Figure S3E,F), presumably as a result of suppressed p21 induction, since p21 could no longer suppress JNK and caspase signaling in response to persistent DNA damage [40]. Overall, these data demonstrate that ML216 efficiently reduces the level of DNA damage-induced senescence. 

### 3.5. ML216 Alleviates Senescence In Vivo and Improves the Health in Naturally Aged Mice

**Figure 6:**
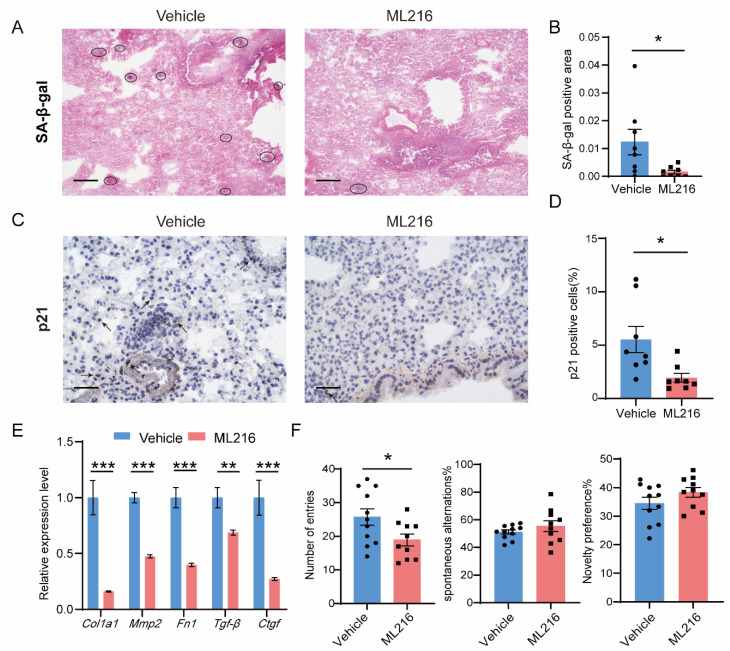
ML216 alleviates senescence in vivo and improves health in naturally aged mice. Two groups of 22-month-old mice were subjected to intraperitoneal injection with vehicle or 1 mg/kg ML216 twice a week for 5 weeks. (**A**) Representative images and (**B**) the quantification of SA-β-gal-positive cells in 22-month-old mice lungs (Vehicle, *n* = 8; ML216, *n* = 8). Scale bar = 100 μm. SA-β-gal-positive cells are blue areas indicated by black circles. (**C**) Representative images and (**D**), the quantification of p21-positive cells in mice lungs (Vehicle, *n* = 8; ML216, *n* = 8). Scale bar = 50 μm. P21-positive cells are indicated with arrowheads. (**E**) Gene expression levels of fibrosis marker genes *COL1A1*, *MMP2*, *FN1*, *TGF-β* and *CTGF* in mice lungs (Vehicle, *n*= 9–11; ML216, *n* = 8). (**F**) Number of arm entries, spontaneous alternations and novelty percentage in Y-maze test (Vehicle, *n* = 11; ML216, *n* = 10). In (**B**,**D**,**F**), the black circles represent mice in vehicle group, the black squares represent mice in ML216 group. All data were presented as mean ± SEM.

Age-related processes, particularly senescence and inflammation, are predisposing factors that contribute to fibrosis of the lung, heart and liver [41]. With aging, the levels of p21 increased in multiple tissues such as the liver and muscle, while DBC1 and BLM levels simultaneously declined (Supplementary Figure S4A,B). To test ML216′s effect of repressing senescence in vivo, 22-month-old mice from two group were injected intraperitoneally with ML216 or vehicle twice a week for 5 weeks (Supplementary Figure S5A). The physiological metrics, such as body weight and running time, in ML216-treated mice remained comparable with those of the control mice (Supplementary Figure S5B,C). The protein levels of inflammatory factors, such as IL-8 and MCP-1, in the lungs were not different between the two groups, although IL-6 displayed a non-significant downward trend in ML216-treated mice (Supplementary Figure S5D). According to immunostaining, the number of senescent cells was significantly lower in the lungs of the ML216-treated mice (Figure 6A,B), as was the level of p21; while BLM levels showed an increasing trend (Figure 6C,D and Supplementary Figure S6A,B). The expression levels of fibrosis markers in the lungs decreased significantly following ML216 treatment (Figure 6E), as were hydroxyproline levels though without significance (Supplementary Figure S6C). ML216 administration reduced p21 induction in the livers and lowered hydroxyproline to a level comparable to that of young mice (Supplementary Figure S7A–C). Age-related cognitive decline includes deficits in spatial learning and memory [42]. ML216 improved short-term memory in aged mice, as indicated by a significantly lower number of total entries in the Y-maze test, as well as a tendency of improving spontaneous alternation and novelty preferences (Figure 6F). These results suggest that ML216 may effectively reduce senescence and fibrosis in vivo and improve the health of aged mice. 

### 3.6. ML216 Reduces Senescence-Associated Pathological Changes in IPF Mice

**Figure 7:**
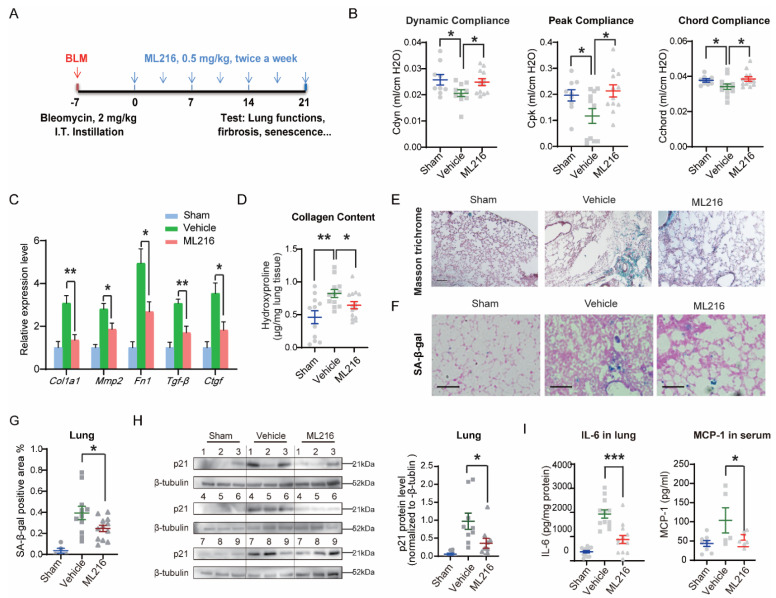
ML216 reduces senescence-associated pathological changes in IPF mice. (**A**) Schematic to illustrate the experimental design of bleomycin-induced lung fibrosis in C57BL/6 J mice. (**B**) Measurements of the pulmonary function parameters: dynamic compliance, peak compliance and chord compliance in mice (Sham, *n* = 7–9; vehicle, *n* = 10–12; ML216, *n* = 12). (**C**) Gene expression levels of fibrosis marker genes *COL1A1*, *MMP2*, *FN1*, *TGF-β* and *CTGF* in mice lungs (*n* = 5 for each group). (**D**) The collagen content in mice lungs measured by hydroxyproline assay (Sham, *n* = 11; vehicle, *n* = 12; ML216, *n* = 14). (**E**) Representative images of the Masson’s trichrome staining of mice lungs. Scale bar = 100 μm. (**F**) Representative images and (**G**) the quantification of SA-β-gal-positive cells in mice lungs (Sham, *n* = 5; Vehicle, *n* = 11; ML216, *n* = 12). Scale bar = 100 μm. (**H**) Western blotting analysis of p21 protein levels in mouse lungs (*n* = 9 for each group). The quantification of p21 by ImageJ are shown on the right. (**I**) Protein levels of SASP factors: IL-6 in lung and MCP-1 in serum, measured by ELISA assay (Sham, *n* = 5–13; Vehicle, *n*= 5–12; ML216, *n* = 7–13). In B, D, and G-I, the gray circles represent mice in sham group, the gray square represent mice in vehicle group, the gray triangles represent mice in ML216 group. All data are presented as mean ± SEM.

Senescent fibroblasts contribute to the etiology of IPF (idiopathic pulmonary fibrosis), and their selective removal improves the health and functions of fibrotic lungs [14,43]. We used a single intratracheal instillation of bleomycin to induce DNA damage and senescence in mouse lungs [14,44] (Figure 7A). One week after bleomycin instillation, two groups of mice received intraperitoneal injections of ML216 or vehicle twice a week for three weeks. Neither the body weight nor the survival rate of the two groups differed significantly during this course (Supplementary Figure S8A,B). Massive fibrosis in IPF mice causes a profound reduction in lung mechanics, including compliance and volume [45]. Three weeks post-injection of ML216 or vehicle, we evaluated the pulmonary functions in both groups of mice. Bleomycin reduced lung elasticity by 20–50%, according to compliance index, such as dynamic compliance, peak compliance, and chord compliance, all of which were significantly improved by the administration of ML216 (Figure 7B). Tidal volume, time to occlusion, and other lung volume metrics displayed an improvement following ML216 treatment, although the difference was not statistically significant (Supplementary Figure S8C). Physical functions, as indicated by riding time in the rotarod performance test, showed the same non-significant trend (Supplementary Figure S8D). The mRNA expressions levels of fibrosis markers were also reduced in ML216-treated mice (Figure 7C). Moreover, the collagen content in the lungs of mice treated with ML216 was significantly lower than in mice treated with the vehicle (Figure 7D). Masson’s trichrome and H&E staining of the lung sections indicated less fibrosis in ML216-treated mice (Figure 7E and Figure S9A). The number of SA-β-gal^+^ cells was also reduced in the lungs of ML216-treated mice (Figure 7F,G and Figure S9B), suggesting that the overall senescence status was less severe than in the vehicle-treated mice. The p21 levels in the lungs of ML216-treated mice was significantly lower than in those of their vehicle-treated counterparts, while the BLM protein level was increased (Figure 7H and Figure S9C). Evaluation of the key SASP factors in mouse lung and serum by ELISA demonstrated fibrosis caused a considerable elevation in IL-6, CXCL1 and MCP-1 levels. ML216 significantly reduced the level of IL-6 in the lung and the level of MCP-1 in serum, while the other factors displayed a downward trend (Figure 7I and Figure S9D,E). Taken together, these results demonstrate that ML216 alleviates bleomycin-induced fibrosis by decreasing the level of cellular senescence in the lungs of IPF mice, improving their pulmonary functions.

## 4. Discussion 

A multitude of data [38,46,47], including ours (Figure 2C), indicate that a lack of BLM accelerates senescence. According to our present findings, DBC1 acts as a protector of BLM, especially during DNA damage. As severe DNA damage occurs, activated caspases breakdown BLM, which presumably induces p21 and a subsequent state of senescence. This may serve as a salvage mechanism to retain tissue integrity by converting damaged cells into senescent cells instead of losing them to apoptosis. Excessive senescence induction, however, can be detrimental. As a countermeasure, DBC1 protects BLM from degradation by binding to it, repressing p21 and the induction of senescence, thus preventing too many cells with unresolved damage from entering a senescent state. This mechanism appears to manage the fate of cells possessing DNA damage by balancing the levels of senescence and apoptosis (Supplementary Figure S10), which may be skewed as DBC1 and BLM expression decreases during aging. Following DNA damage, the reduced DBC1 levels in aged mice may aggravate BLM depletion and result in further senescence induction, leading to the accumulation of senescent cells. 

DBC1 may affect senescence differently depending on the type of tissue or stimulus. For example, DBC1 deletion protects adipose tissues from senescence in obese mice [27]; yet, vascular smooth muscle cells in DBC1 knockout mice fail to proliferate with an increased expressions of MMPs in response to ANGII [48]; and we found that DBC1 suppressed senescence following DNA damage. DBC1 knockout mice have a shorter lifespan and an almost 2-fold higher tumorigenesis rate than wild-types [37], implying the overall functions of DBC1 in vivo are favorable. Interestingly, patients with the Bloom’s syndrome have an average lifespan of 25 years [30], and BLM-deficient cells display a high sister chromatid exchange (SCE) ratio that is positively linked to mutation rate and cancer incidence [31,49,50]. Our findings explain the similar phenotypes seen when lacking BLM and DBC1. It is possible that unprotected BLM in DBC1 knockout mice diminishes faster with aging, resulting in a higher SCE ratio, an increased number of mutations, and eventually the onset of tumorigenesis. 

Senescence is characterized by high p21 induction and permanent cell cycle arrest [36]. Knocking down the p21 gene can bypass senescence induction [51]. We showed here that ML216 promoted DBC1–BLM interaction, which suppressed p21 and senescence induction and aided in the preservation of BLM following DNA damage. Furthermore, we demonstrated that ML216 significantly reduced the number of DNA damage-induced senescent cells and improved the pulmonary function in IPF mice. These findings suggest that ML216 may be a potential treatment for senescence-related diseases. In contrast to current techniques for the removal of senescent cells, ML216 prevents cells with irreparable DNA damage from becoming senescent by lowering p21 induction and presumably resulting in apoptosis. Bleomycin-induced lung injury exhibits the molecular signatures of senescence, which is the major pathogenic driving force [52]. ML216 significantly reduced lung fibrosis and improved pulmonary function in bleomycin-induced IPF mice. Furthermore, since aging causes increased fibrosis in multiple organs due to the accumulation of senescent cells, the effect of ML216 administration on aged mice was evaluated, and the fibrotic status of the lung and liver was found to be improved. In both mouse models, a low number of senescent cells and/or less p21 induction was seen following ML216 treatment, in addition to a greater preservation of BLM, consistent with alleviated senescence in vivo. Taken together, our data suggest that ML216 may be a specific and effective therapy candidate for senescence-associated pathologies. 

Previous studies have shown that knocking down BLM induces a greater number of senescent cells [38,46,47]; however, ML216 administration significantly reduced the level of DNA damage-induced senescence (Figure 5E,F). BLM is important for the homologous recombination repair of DNA double-stranded breaks [29,53]. Cells with BLM deficiency or mutations possess a greater number of γ-H2AX or 53BP1 foci [47,54,55], indicating a higher level of DNA damage. However, co-treatment of ML216 with DNA damage reagents reduces the number of γ-H2AX foci (Supplementary Figure S3B) [31]. These discrepancies between BLM knockdown and ML216 treatment do not support the function of ML216 as an inhibitor of the helicase activity of BLM but are in agreement with ML216 acting to protect BLM integrity. This suggests that the functions of BLM in senescence induction or DNA repair may rely on its enzymatic activity to a lesser extent than previously thought. BLM may mainly affect DNA repair and senescence by physically interacting with various DNA repair proteins, such as ATM [56], RAD51 [57], RAD54 [58], topoisomerase III alpha [59], and MLH1 [60]. In this context, BLM integrity is more crucial than its activity, especially since other RecQ helicase family members, such as WRN helicase, are more active than BLM and can easily compensate for its loss in activity [61]. Thus, ML216 acts primarily as a protector of BLM, reducing senescence and DNA damage, rather than as an inhibitor that would have the opposite effect. 

Here, we uncovered a novel mechanism by which DBC1 suppresses senescence induction by preserving the integrity of BLM and subsequently repressing p21 induction following DNA damage. By manipulating this mechanism, ML216 can reduce senescence induction in vivo, providing a novel strategy for reducing the number of senescent cells and their associated pathologies. Further research is necessary to investigate the long-term effects of ML216 treatment in vivo; however, ML216 appears promising as a potential therapeutic treatment for senescence-related diseases.

## Data Availability

All data are available in the main text or in the Supplementary Materials.

## Supplementary Materials

The following supporting information can be downloaded at: https://www.mdpi.com/article/10.3390/cells12010145/s1, Figure S1: Overexpressing DBC1 promotes apoptosis and decreases cell survival after DNA damage; Figure S2: DBC1 regulates cell cycle, apoptosis and senescence by preserving BLM integrity; Figure S3: ML216 reduces DNA damage response and promotes apoptosis in a DBC1-dependent fashion; Figure S4: DBC1 or BLM decrease in liver and muscle with aging, while p21 increases; Figure S5: ML216 reduces inflammation and improves physiological functions in old mice; Figure S6: ML216 alleviates fibrosis in lung in old mice; Figure S7: ML216 alleviates fibrosis in livers of old mice; Figure S8: ML216 improves physiological functions in IPF mice; Figure S9: ML216 reduces senescence and inflammation in IPF mice; Figure S10: A model for BLM-DBC1 interaction in regulating senescence induction; Table S1: The list for the commonly changed transcripts in both datasets of RNA-Sequencing analysis on DBC1 knockdown cells and BLM knockdown cells, related to Figure 3A; Table S2: The list for the primer sequences used in RT-PCR.
