# Supplementary material for: ML216 Prevents DNA Damage-Induced Senescence by Modulating DBC1–BLM Interaction
Source: Cells. 2022 Dec 29;12(1):145. doi: 10.3390/cells12010145 (PMC9818470; doi:10.3390/cells12010145)
Supplement: Supplementary file 1 [file cells-12-00145-s001.zip › cells-2034577-supplementary Figures.pdf]

SUPPLEMENTARY DATA

ML216 prevents DNA-damage induced senescence by modulating DBC1-BLM interaction

Feng Cui [#], Xueying Han [#], Xiaoqian Zhang [#], Siqi Wang, Na Liang, Qing Tan, Wuga Sha, and Jun Li *

*Correspondence: Jun Li@ibms.pumc.edu.cn

The PDF file includes:

Supplementary Figures S1–S10

Supplementary Tables S1 and S2

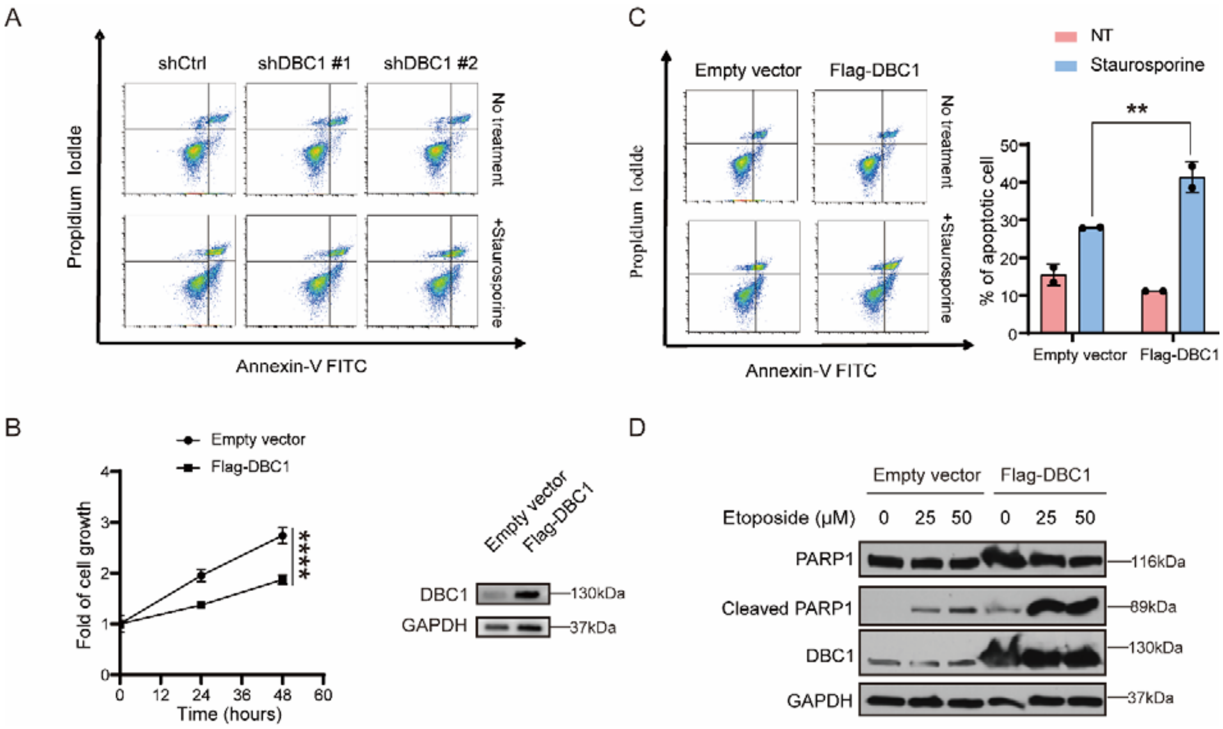
A
shCtrl
shDBC1 #1
shDBC1 #2
No treatment
+Staurosporine
Propidium Iodide
Annexin-V FITC
C
Empty vector
Flag-DBC1
NT
Staurosporine
**
% of apoptotic cell
0
10
20
30
40
50
Empty vector
Flag-DBC1
B
Empty vector
Flag-DBC1
Fold of cell growth
Time (hours)
0
12
24
36
48
60
****
DBC1
GAPDH
130kDa
37kDa
D
Etoposide (μM)
0
25
50
PARP1
Cleaved PARP1
116kDa
89kDa

Supplementary Figure S1. Overexpressing DBC1 promotes apoptosis and decreases cell survival after DNA damage. (A) Representative scatter plots of PI (y-axis) vs. annexin V (x-axis) of the apoptosis analysis described in Fig. 1B. (B) Cell survival analysis of DBC1 overexpression cells with cisplatin (50 μM) treatment using cell counting kit 8, the overexpression level of DBC1 is shown in the right, two-way ANOVA test. (C) Representative scatter plots and quantification of apoptosis analysis on DBC1 overexpression cells treated with Staurosporine (1 μM, 2 hrs) using Annexin V-FITC staining and flow cytometry, n= 2 biological replicates. (D) Western blotting analysis of PARP1 and cleaved PARP1 protein levels in DBC1 overexpression cells treated with Etoposide (25 or 50 μM, 24hrs). All data were presented as mean ± SD.

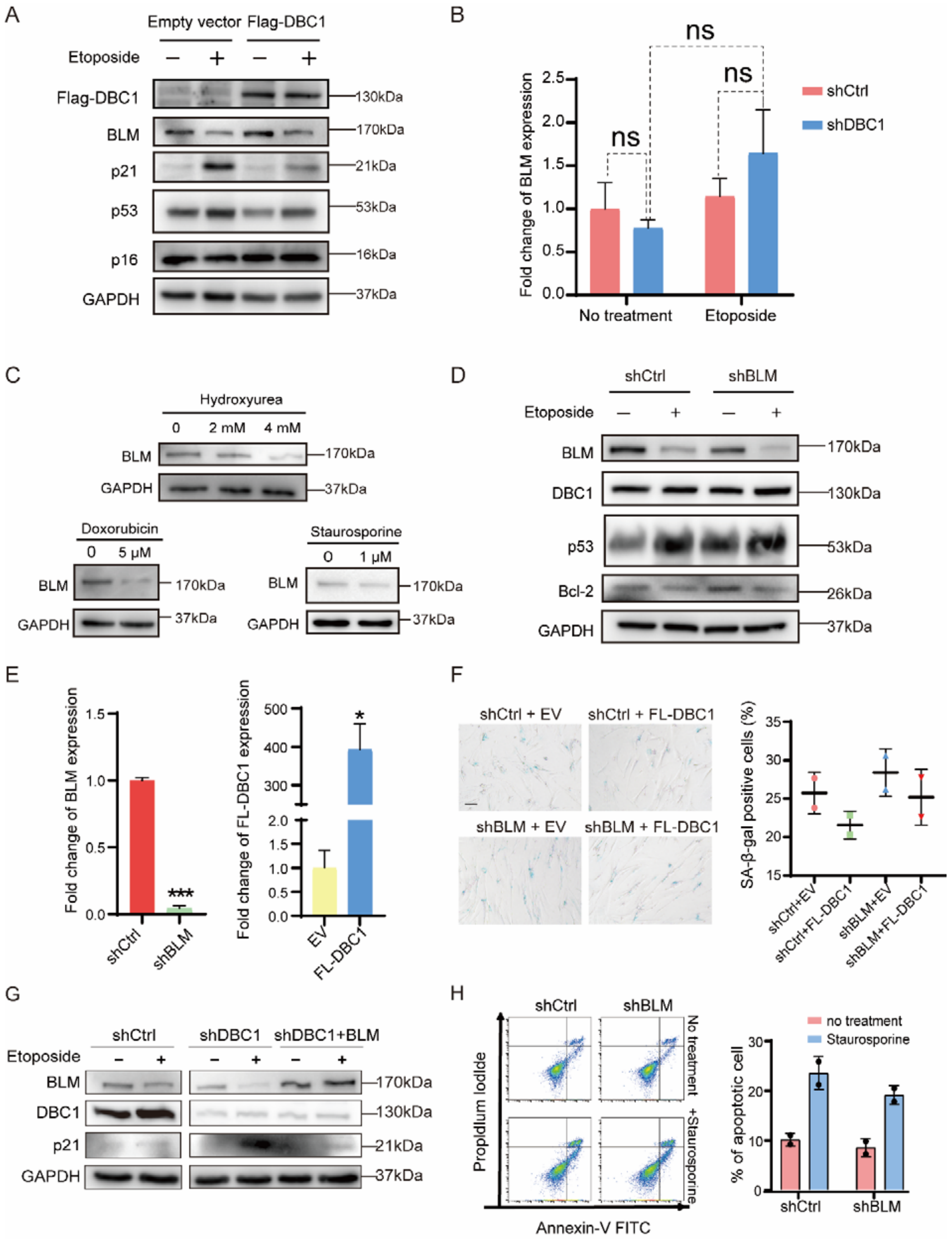
A
Empty vector
Flag-DBC1
Etoposide
Flag-DBC1
BLM
p21
p53
p16
GAPDH
130kDa
170kDa
21kDa
53kDa
16kDa
37kDa
B
ns
ns
ns
shCtrl
shDBC1
Fold change of BLM expression
No treatment
Etoposide
C
Hydroxyurea
0
2 mM
4 mM
BLM
GAPDH
170kDa
37kDa
Doxorubicin
0
5 μM
Staurosporine
0
1 μM
D
shCtrl
shBLM
Etoposide
BLM
DBC1
p53
Bcl-2
GAPDH
170kDa
130kDa
53kDa
26kDa
37kDa
E
Fold change of BLM expression
shCtrl
shBLM
***
Fold change of FL-DBC1 expression
EV
FL-DBC1
*
F
shCtrl + EV
shCtrl + FL-DBC1
shBLM + EV
shBLM + FL-DBC1
SA-β-gal positive cells (%)
shCtrl+EV
shCtrl+FL-DBC1
shBLM+EV
shBLM+FL-DBC1
G
shCtrl
shDBC1
shDBC1+BLM
Etoposide
BLM
DBC1
p21
GAPDH
170kDa
130kDa
21kDa
37kDa
H
shCtrl
shBLM
No treatment
+Staurosporine
Propidium Iodide
Annexin-V FITC
no treatment
Staurosporine
% of apoptotic cell
shCtrl
shBLM

Supplementary Figure S2. DBC1 regulates cell cycle, apoptosis and senescence by preserving BLM integrity. **(A) Western blotting analysis of BLM, p53, p16 and p21 protein levels in DBC1 overexpression cells treated with Etoposide (50 µM, 24 hrs). (B) RT-PCR measurement of BLM expression with DBC1 knockdown upon Etoposide induced DNA damage in the IMR-90 cells. (C) Western blotting analysis of BLM protein levels in 293T cells treated with DNA damage reagents Hydroxyurea (48 hrs), Doxorubicin (24 hrs) and Staurosporine (6 hrs). (D) Western blotting analysis of BLM, p53, BCL-2 and DBC1 protein levels in BLM knockdown cells treated with Etoposide (100 µM, 24 hrs). (E) RT-PCR measurement of knockdown level of BLM and overexpression level of DBC1 in the IMR-90 cells used in Fig. 2F. (F) Representative images and quantification of SA-β-Gal staining of BLM knockdown IMR-90 cells transfected with DBC1, n = 2 biological replicates. Scale bar = 100 µm. (G) Western blotting analysis of BLM, DBC1, and p21 protein levels in DBC1 knockdown 293T cells after overexpressing BLM using lentivirus (Addgene, #127641). (H) Representative scatter plots of PI (y-axis) vs. annexin V (x-axis) in the apoptosis analysis of BLM knockdown cells treated staurosporine (1 µM, 2 hrs) using Annexin V-FITC staining and flow cytometry. The quantifications of apoptotic cells are**

shown in the right, n = 2 biological replicates. Except for [B] ±SEM, all data were presented as mean ± SD.

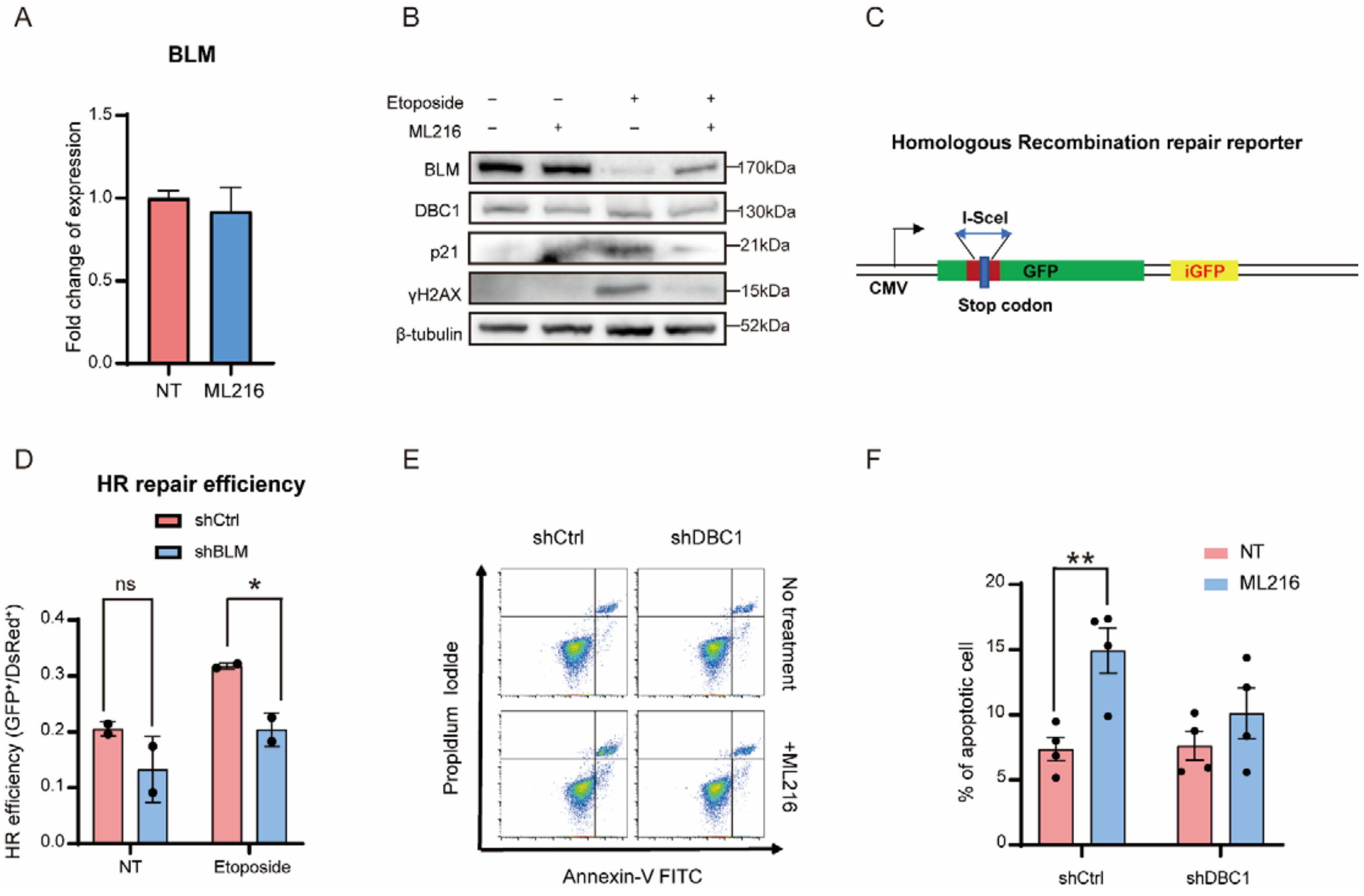

Supplementary Figure S3. ML216 reduces DNA damage response and promotes apoptosis in a DBC1-dependent fashion.**(A) The effect of ML216 on BLM mRNA expression in 293T cells, measured by RT-PCR analysis. (B) Western blotting analysis of BLM, p21, γH2AX and DBC1 protein levels in 293T cells treated with Etoposide (100 μM), ML216 (50 μM) or both for 24 hrs. (C) Schematic design of homologous recombination (HR) repair reporter construct. (D) HR repair efficiency in BLM knockdown cells treated with Etoposide (100 μM, 24 hrs), n = 2 biological replicates. (E) and (F), Representative scatter plots and quantification of apoptosis analysis in BLM knockdown cells treated with ML216 (10 μM, 24 hrs), n = 4 biological replicates. Data were presented as mean ± SD [A, D]; Data were presented as mean ± SEM [F]**

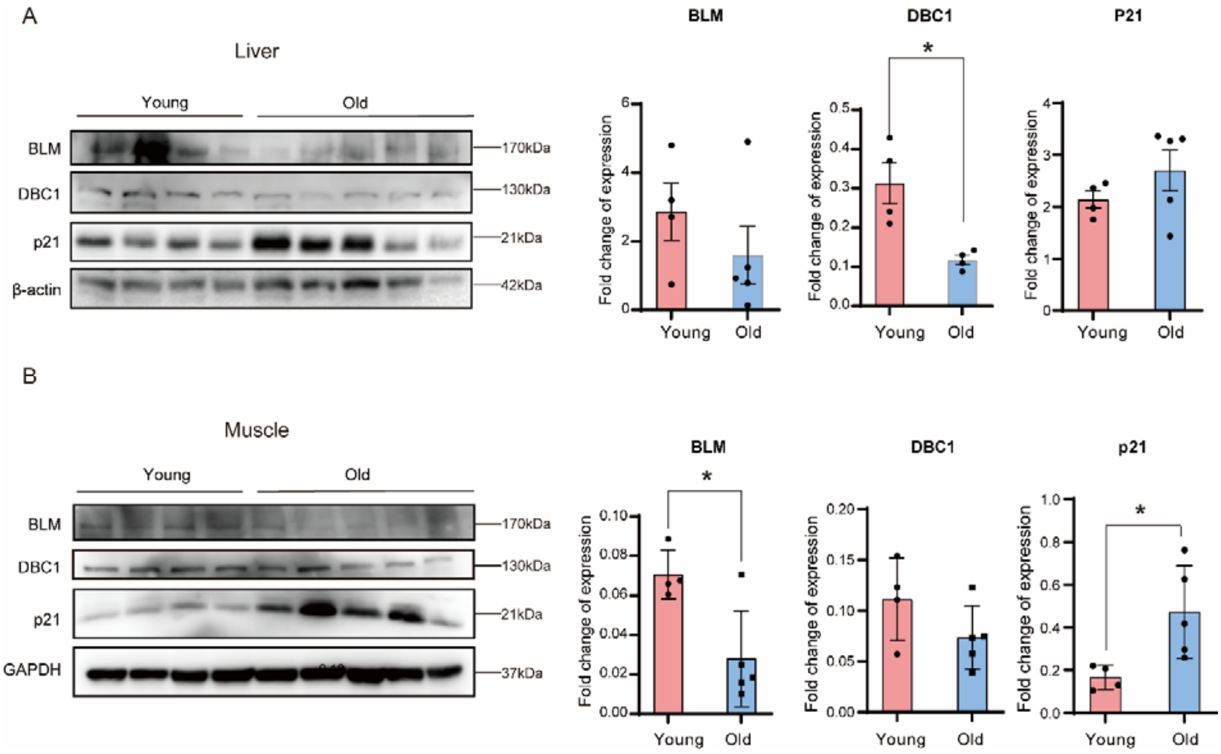
A
Liver
Young
Old
BLM
DBC1
p21
β-actin
170kDa
130kDa
21kDa
42kDa
BLM
DBC1
P21
Fold change of expression
Young
Old
*
B
Muscle
Young
Old
BLM
DBC1
p21
GAPDH
170kDa
130kDa
21kDa
37kDa
BLM
DBC1
p21
Fold change of expression
Young
Old
*
*

.

Supplementary Figure S4 DBC1 or BLM decrease in liver and muscle with aging, while p21 increases. **(A-B) Western blotting analysis of BLM, DBC1 and p21 protein levels in liver and muscle of young (6 months, n=4) and old (20-22 months, n=5) C57BL/6 mice. All data were presented as mean ±SEM.**

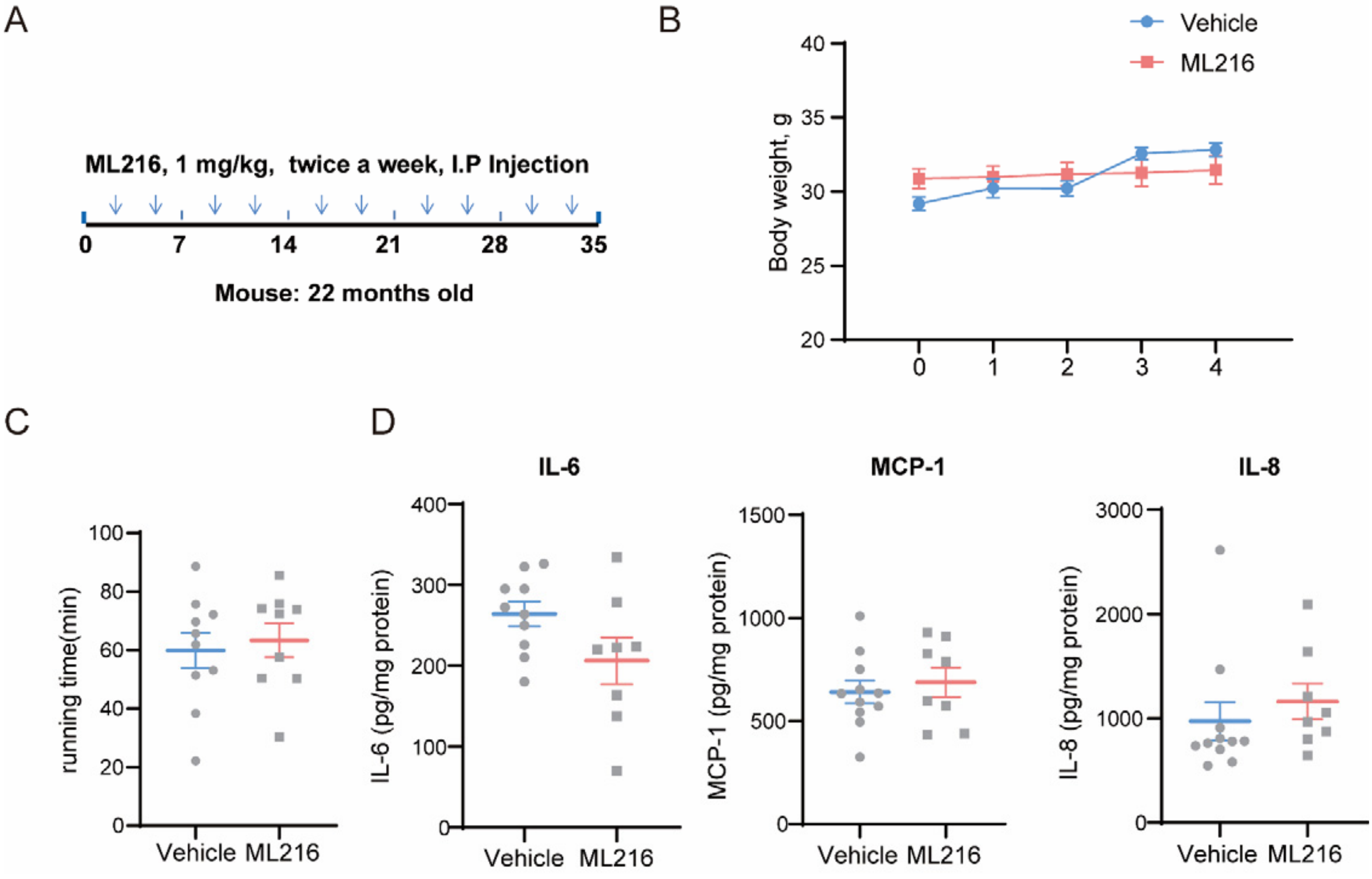
A
ML216, 1 mg/kg, twice a week, I.P Injection
0
7
14
21
28
35
Mouse: 22 months old
B
Vehicle
ML216
Body weight, g
40
35
30
25
20
0
1
2
3
4
C
running time(min)
100
80
60
40
20
0
Vehicle
ML216
D
IL-6
IL-6 (pg/mg protein)
400
300
200
100
0
Vehicle
ML216
MCP-1
MCP-1 (pg/mg protein)
1500
1000
500
0
Vehicle
ML216
IL-8
IL-8 (pg/mg protein)
3000
2000
1000
0
Vehicle
ML216

Supplementary Figure S5 ML216 reduces inflammation and improves physiological functions in old mice.**(A) Schematic to illustrate experimental design of naturally aging C57BL/6 J mice. (B) Body weight of vehicle (n = 11) or ML216 (n = 8) treated mice. (C) Quantification of maximal running time in the rotarod test for vehicle (n = 12) or ML216 (n = 14) treated mice. (D) Protein levels of SASP factors IL-6, MCP-1 and IL-8 in lung, measured by ELISA assay (Vehicle, n = 5-11; ML216, n = 7-8). All data were presented as mean ±SEM.**

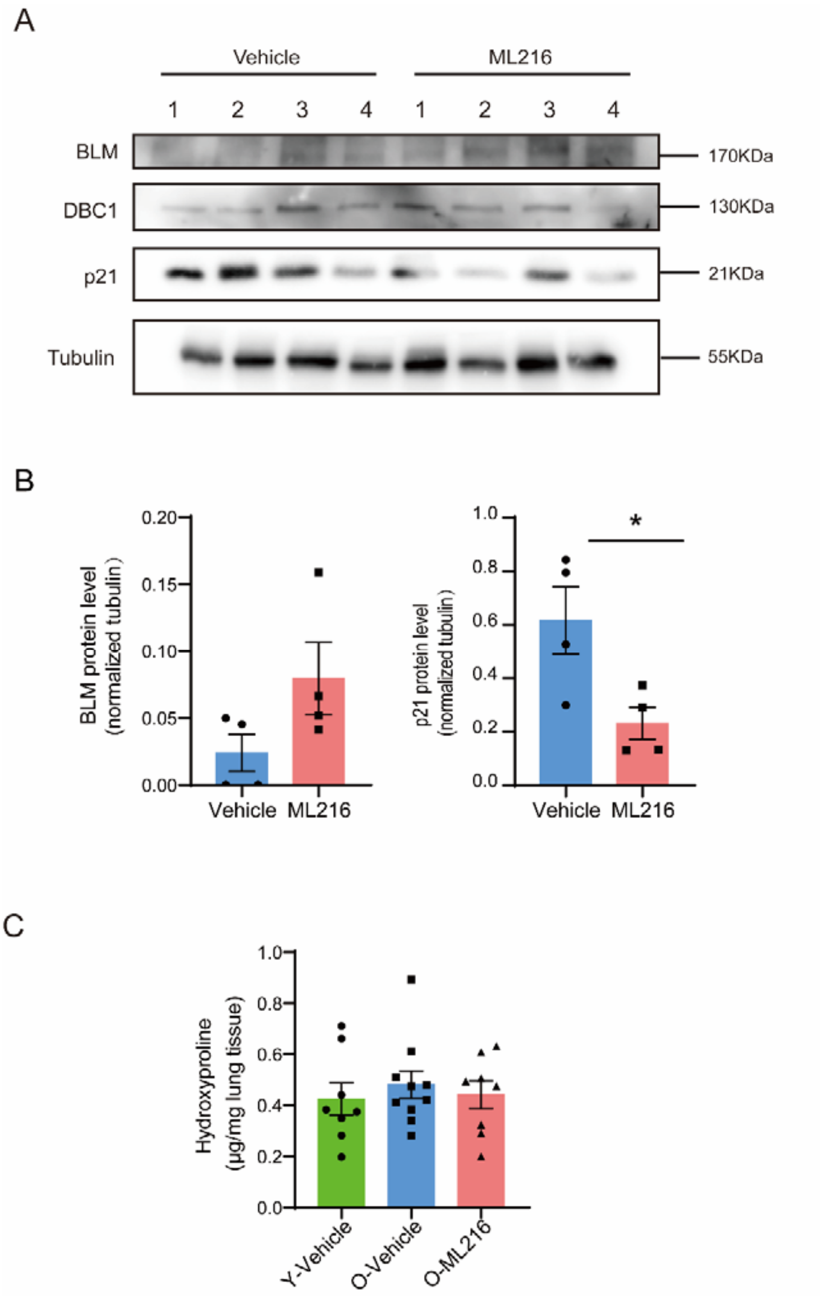
A
Vehicle
ML216
1 2 3 4 1 2 3 4
BLM
DBC1
p21
Tubulin
170KDa
130KDa
21KDa
55KDa
B
BLM protein level (normalized tubulin)
0.20
0.15
0.10
0.05
0.00
Vehicle ML216
p21 protein level (normalized tubulin)
1.0
0.8
0.6
0.4
0.2
0.0
*
Vehicle ML216
C
Hydroxyproline (μg/mg lung tissue)
1.0
0.8
0.6
0.4
0.2
0.0
Y-Vehicle
O-Vehicle
O-ML216

Supplementary Figure S6 ML216 alleviates fibrosis in lung in old mice. **(A-B) Western blotting analysis of BLM, DBC1 and p21 protein levels in lung of vehicle-treated (n = 4) and ML216-treated (n = 4) aging C57BL/6 mice, the quantifications of BLM and p21 protein levels in mouse lungs by ImageJ. (C) The collagen contents in lungs measured by hydroxyproline assay of old mouse treated with vehicle groups (O-Vehicle, n = 11) or ML216 groups (O-ML216, n = 8), young mice (4 months) were used as control (Y-Vehicle, n = 8). All data were presented as mean ±SEM.**

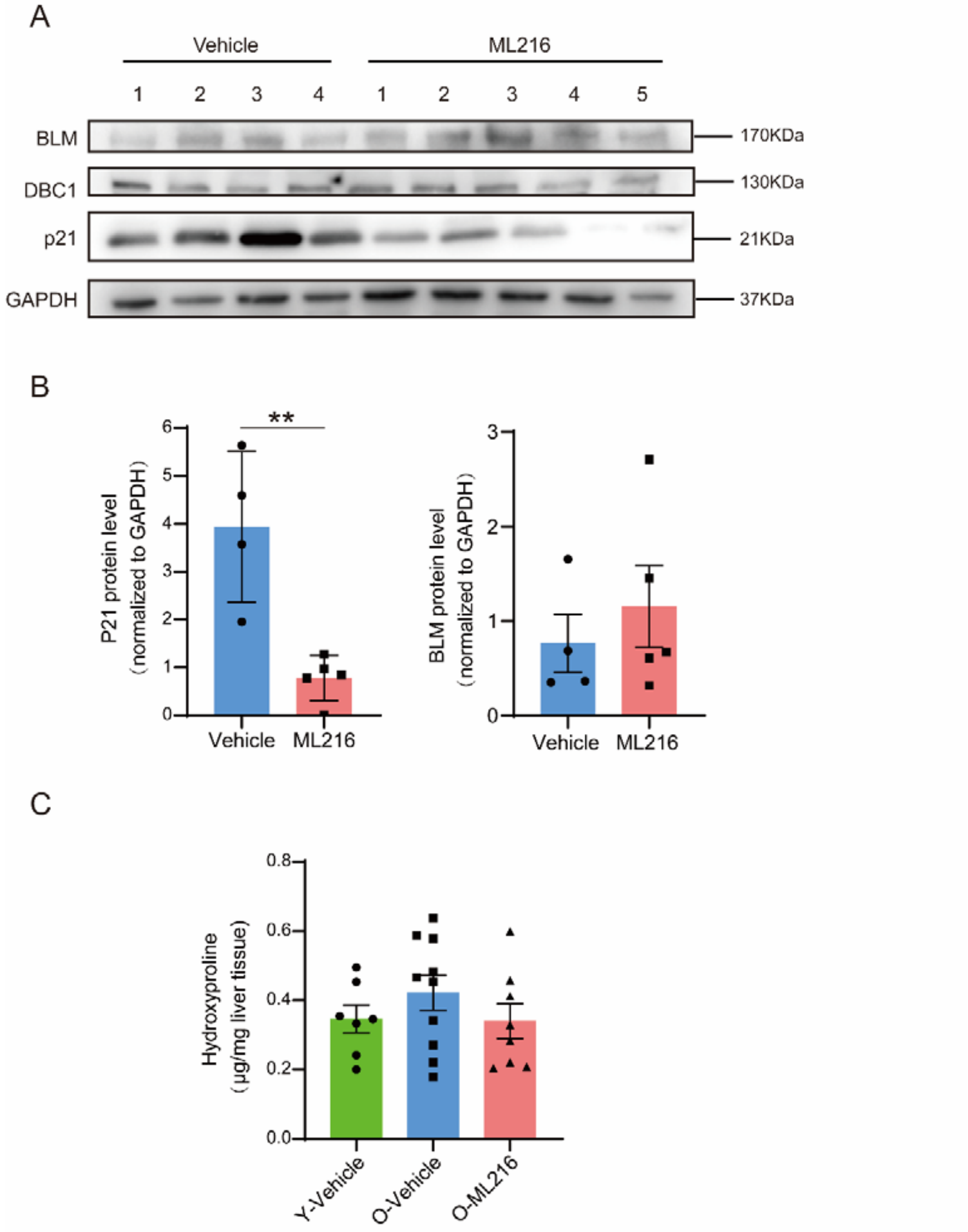

A
Vehicle
ML216
1
2
3
4
1
2
3
4
5
BLM
DBC1
p21
GAPDH
170KDa
130KDa
21KDa
37KDa
B
P21 protein level
（normalized to GAPDH）
**
Vehicle
ML216
BLM protein level
（normalized to GAPDH）
Vehicle
ML216
C
Hydroxyproline
（μg/mg liver tissue）
Y-Vehicle
O-Vehicle
O-ML216

Supplementary Figure S7 ML216 alleviates fibrosis in livers of old mice. **(A-B) Western blotting analysis of BLM, DBC1 and p21 protein levels in liver of vehicle-treated (n = 4) and ML216-treated (n = 5) aging C57BL/6 mice, the quantifications of BLM and p21 protein levels in mouse livers by ImageJ. (C) The collagen contents in livers measured by hydroxyproline assay of old mouse treated with vehicle groups (O-Vehicle, n = 11) or ML216 groups (O-ML216, n = 8), young mice (4 months) were used as control (Y-Vehicle, n = 7). All data were presented as mean ±SEM.**

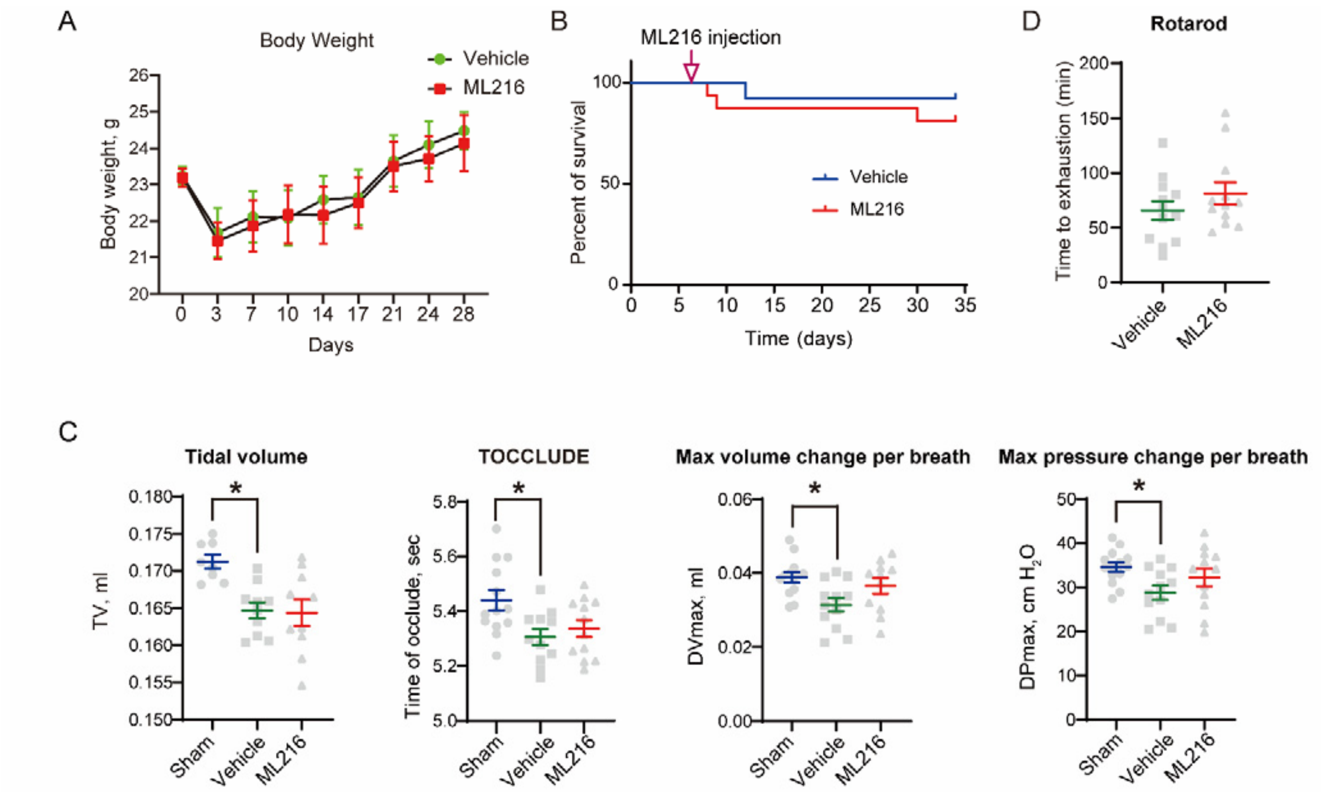
A
Body Weight
Vehicle
ML216
Body weight, g
Days
B
ML216 injection
Percent of survival
Vehicle
ML216
Time (days)
D
Rotarod
Time to exhaustion (min)
Vehicle
ML216
C
Tidal volume
TV, ml
TOCCLUDE
Time of occlude, sec
Max volume change per breath
DVmax, ml
Max pressure change per breath
DPmax, cm H2O
Sham
Vehicle
ML216

Supplementary Figure S8. ML216 improves physiological functions in IPF mice.

(**A**) Body weight of vehicle (n = 12) or ML216 (n = 14) treated mice. (**B**) Survival curves of vehicle (n = 12) or ML216 (n = 14) treated mice, long-rank test p=0.3909. (**C**) Pulmonary function measurements of Tidal volume, Tocclude, max volume change per breath and max pressure change per breath in mice (Sham, n = 13-14; Vehicle, n = 11-12; ML216, n = 11-12). (**D**) Quantification of maximal running time in the rotarod test for vehicle (n = 12) or ML216 (n = 14) treated mice. All data were presented as mean ± SEM.

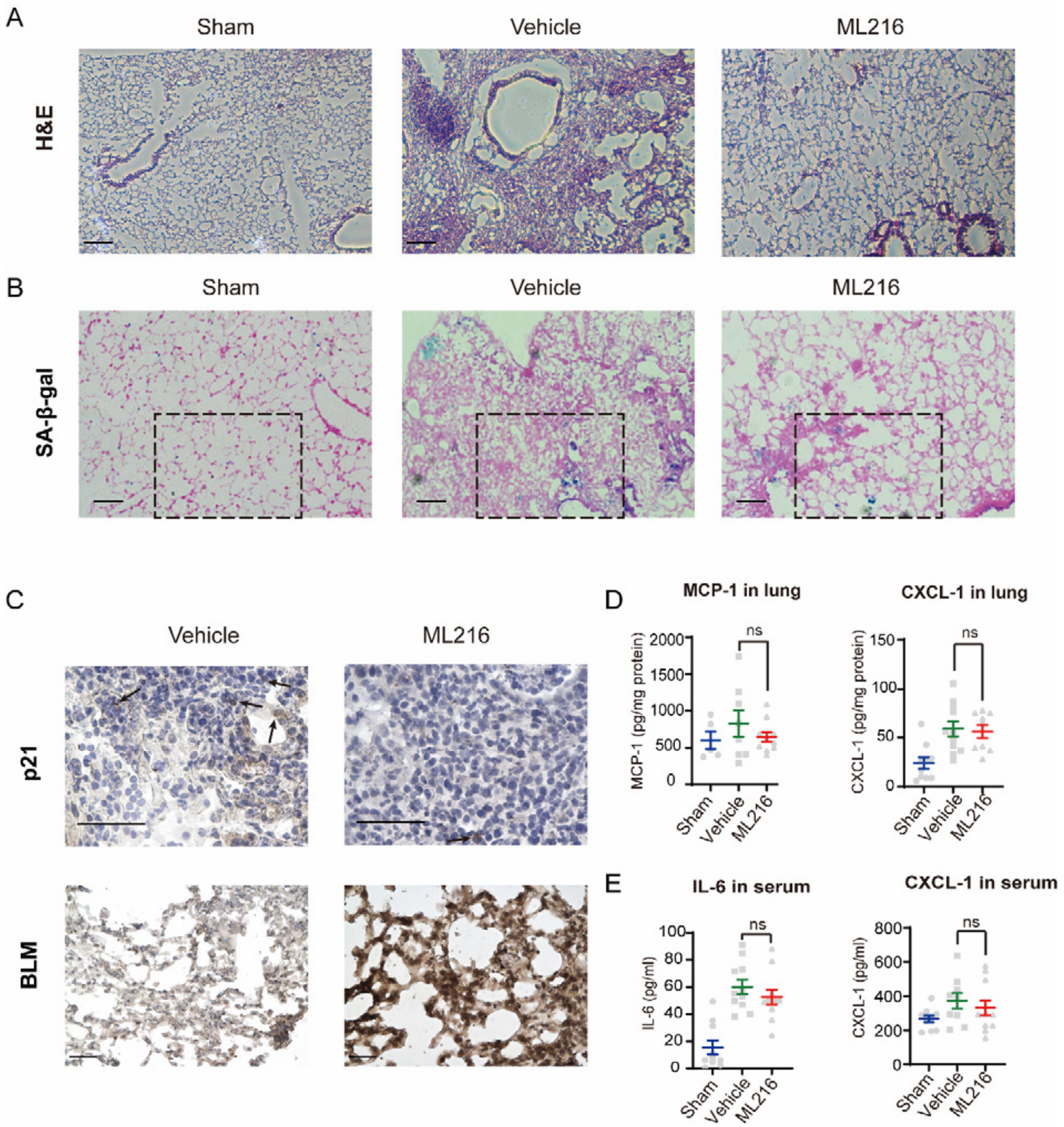
A
Sham
Vehicle
ML216
H&E
B
Sham
Vehicle
ML216
SA-β-gal
C
Vehicle
ML216
p21
BLM
D
MCP-1 in lung
MCP-1 (pg/mg protein)
2000
1500
1000
500
0
ns
Sham
Vehicle
ML216
CXCL-1 in lung
CXCL-1 (pg/mg protein)
150
100
50
0
ns
Sham
Vehicle
ML216
E
IL-6 in serum
IL-6 (pg/ml)
100
80
60
40
20
0
ns
Sham
Vehicle
ML216
CXCL-1 in serum
CXCL-1 (pg/ml)
800
600
400
200
0
ns
Sham
Vehicle
ML216

Supplementary Figure S9 ML216 reduces senescence and inflammation in IPF mice.

(**A**) Representative images of H&E staining on lung sections of mice. Scale bar = 100 μm. (**B**) Representative images of SA-β-gal staining positive cells in mouse lungs. Scale bar= 100 μm. (**C**) Representative images of immunohistochemistry (IHC) staining for p21 and BLM, Scale bar = 25 μm. (**D**) and (**E**) Protein levels of SASP factors IL-6, MCP-1 and CXCL-1 in lung [C] and serum [F], measured by ELISA assay (Sham, n = 5-13; Vehicle, n = 5-12; ML216, n = 7-13). All data are presented as mean ± SEM.

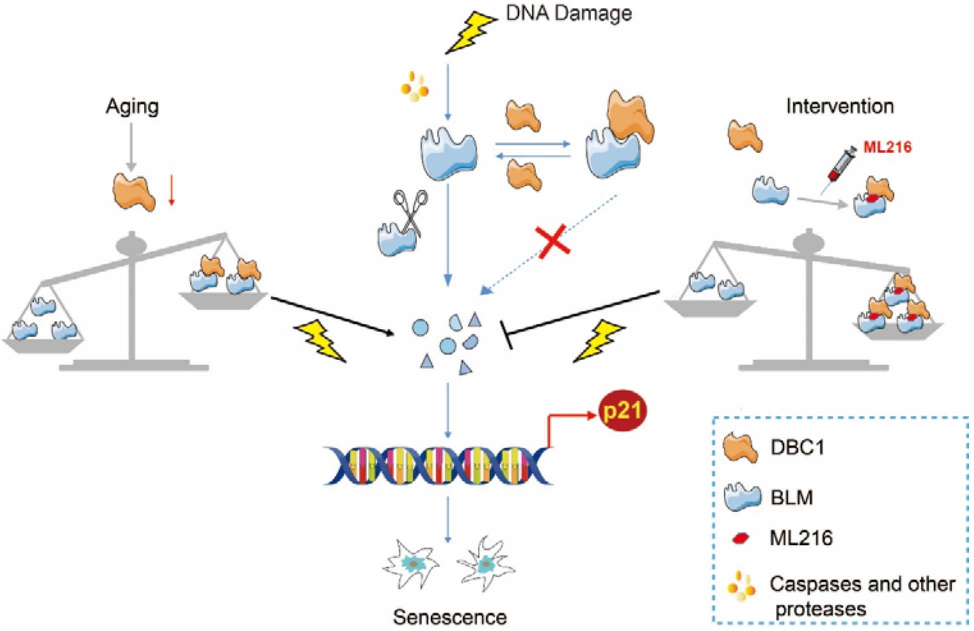

Supplementary Figure S10 A model for BLM-DBC1 interaction in regulating senescence induction.**This Fig. was made with Servier Medical Art templates, which are licensed under a Creative Commons Attribution 3.0 Unported Licence (www.smart.servier.com).**
