# Supplementary material for: ML216 Prevents DNA Damage-Induced Senescence by Modulating DBC1–BLM Interaction
Source: Cells. 2022 Dec 29;12(1):145. doi: 10.3390/cells12010145 (PMC9818470; doi:10.3390/cells12010145)
Supplement: Supplementary file 1 [file cells-12-00145-s001.zip › cells-2034577-supplementary Table S1.pdf]

Supplementary Table S1 The list for the commonly changed transcripts in both datasets of RNA-Sequencing analysis on DBC1 knockdown cells and BLM knockdown cells, related to Fig. 3A.

Genes differentially expressed in shDBC1 cells (p<0.05; log2FoldChang>1)				
**Gene_id**	**Gene_name**	**log2FoldChange**	**P value**	**Gene_biotype**
ENSG00000260287	TBC1D3G	-7.811398504	1.09E-06	protein_coding
ENSG00000189001	SBSN	-7.06091978	0.000193974	protein_coding
ENSG00000077080	ACTL6B	-6.525254434	0.002434933	protein_coding
ENSG00000237330	RNF223	-6.389316369	0.004174171	protein_coding
ENSG00000214402	LCNL1	-6.23922431	0.007243414	protein_coding
ENSG00000162006	MSLNL	-6.23922431	0.007243414	protein_coding
ENSG00000166592	RRAD	-6.23922431	0.007243414	protein_coding
ENSG00000283189	AC104452.1	-6.071684547	0.012730848	protein_coding
ENSG00000070731	ST6GALNAC2	-6.071684547	0.012730848	protein_coding
ENSG00000135114	OASL	-5.882100421	0.022676824	protein_coding
ENSG00000126856	PRDM7	-5.882100421	0.022676824	protein_coding
ENSG00000284820	AC068946.2	-5.663779193	0.040964585	protein_coding
ENSG00000130173	ANGPTL8	-5.663779193	0.040964585	protein_coding
ENSG00000157087	ATP2B2	-5.663779193	0.040964585	protein_coding
ENSG00000104879	CKM	-5.663779193	0.040964585	protein_coding
ENSG00000169393	ELSPBP1	-5.663779193	0.040964585	protein_coding
ENSG00000151948	GLT1D1	-5.663779193	0.040964585	protein_coding
ENSG00000150782	IL18	-5.663779193	0.040964585	protein_coding
ENSG00000005844	ITGAL	-5.663779193	0.040964585	protein_coding
ENSG00000243709	LEFTY1	-5.663779193	0.040964585	protein_coding
ENSG00000283654	LMLN2	-5.663779193	0.040964585	protein_coding
ENSG00000139679	LPAR6	-5.663779193	0.040964585	protein_coding
ENSG00000102854	MSLN	-5.663779193	0.040964585	protein_coding
ENSG00000170476	MZB1	-5.663779193	0.040964585	protein_coding
ENSG00000179256	SMCO3	-5.663779193	0.040964585	protein_coding
ENSG00000269404	SPIB	-5.663779193	0.040964585	protein_coding
ENSG00000120738	EGR1	-4.437832218	3.73E-20	protein_coding
ENSG00000128482	RNF112	-4.020758111	0.000884859	protein_coding
ENSG00000169885	CALML6	-3.967917634	1.47E-05	protein_coding
ENSG00000182853	VMO1	-3.933931	0.001369576	protein_coding
ENSG00000198520	ARMH1	-3.841541751	0.002133716	protein_coding
ENSG00000215217	C5orf49	-3.841541751	0.002133716	protein_coding
ENSG00000179388	EGR3	-3.764467024	2.58E-12	protein_coding
ENSG00000135625	EGR4	-3.742828672	0.003346055	protein_coding
ENSG00000242252	BGLAP	-3.636862034	0.005281544	protein_coding
ENSG00000126895	AVPR2	-3.522490688	0.008390282	protein_coding
ENSG00000172232	AZU1	-3.398265654	0.013412089	protein_coding
ENSG00000167157	PRRX2	-3.338724529	0.000739506	protein_coding

ENSG00000101883	RHOXF1	-3.338724529	0.000739506	protein_coding
ENSG00000099840	IZUMO4	-3.336258471	8.34E-08	protein_coding
ENSG00000124249	KCNK15	-3.265179506	0.001084588	protein_coding
ENSG00000213937	CLDN9	-3.262327589	0.021566548	protein_coding
ENSG00000148655	LRMDA	-3.262327589	0.021566548	protein_coding
ENSG00000176945	MUC20	-3.262327589	0.021566548	protein_coding
ENSG00000010379	SLC6A13	-3.262327589	0.021566548	protein_coding
ENSG00000105427	CNFN	-3.260066247	8.69E-10	protein_coding
ENSG00000121552	CSTA	-3.213969277	0.000214972	protein_coding
ENSG00000068976	PYGM	-3.213969277	0.000214972	protein_coding
ENSG00000130643	CALY	-3.200161788	2.79E-08	protein_coding
ENSG00000111678	C12orf57	-3.189774211	2.02E-11	protein_coding
ENSG00000182179	UBA7	-3.166696499	2.30E-08	protein_coding
ENSG00000268041	AC010616.1	-3.159768494	0.000299755	protein_coding
ENSG00000105479	CCDC114	-3.130090117	7.20E-06	protein_coding
ENSG00000089101	CFAP61	-3.112235529	0.034866602	protein_coding
ENSG00000105523	FAM83E	-3.112235529	0.034866602	protein_coding
ENSG00000261949	GFY	-3.112235529	0.034866602	protein_coding
ENSG00000108405	P2RX1	-3.112235529	0.034866602	protein_coding
ENSG00000176358	TAC4	-3.112235529	0.034866602	protein_coding
ENSG00000179930	ZNF648	-3.112235529	0.034866602	protein_coding
ENSG00000174521	TTC9B	-3.105786171	0.003515158	protein_coding
ENSG00000197921	HES5	-3.084823973	9.18E-08	protein_coding
ENSG00000176046	NUPR1	-3.084010425	5.36E-11	protein_coding
ENSG00000278817	AC007325.4	-3.000964481	3.17E-10	protein_coding
ENSG00000178522	AMBN	-2.985608339	3.61E-08	protein_coding
ENSG00000100033	PRODH	-2.983760269	0.000829981	protein_coding
ENSG00000173991	TCAP	-2.981856656	3.84E-07	protein_coding
ENSG00000125740	FOSB	-2.972509594	4.39E-11	protein_coding
ENSG00000107796	ACTA2	-2.965268706	0.000252157	protein_coding
ENSG00000198881	ASB12	-2.92656981	0.007864773	protein_coding
ENSG00000180644	PRF1	-2.92656981	0.007864773	protein_coding
ENSG00000160188	RSPH1	-2.92656981	0.007864773	protein_coding
ENSG00000103269	RHBDL1	-2.912298415	2.57E-10	protein_coding
ENSG00000122877	EGR2	-2.848032956	5.38E-06	protein_coding
ENSG00000168918	INPP5D	-2.827856732	0.011832427	protein_coding
ENSG00000142538	PTH2	-2.827856732	0.011832427	protein_coding
ENSG00000131096	PYY	-2.827856732	0.011832427	protein_coding
ENSG00000125508	SRMS	-2.827856732	0.011832427	protein_coding
ENSG00000168350	DEGS2	-2.823005296	4.60E-05	protein_coding
ENSG00000170345	FOS	-2.765356114	4.88E-10	protein_coding
ENSG00000228727	SAPCD1	-2.745149476	0.000372105	protein_coding
ENSG00000205090	TMEM240	-2.728100079	9.35E-05	protein_coding

ENSG00000197599	CCDC154	-2.721890093	0.017862218	protein_coding
ENSG00000183615	FAM167B	-2.721890093	0.017862218	protein_coding
ENSG00000128310	GALR3	-2.721890093	0.017862218	protein_coding
ENSG00000109758	HGFAC	-2.721890093	0.017862218	protein_coding
ENSG00000205323	SARNP	-2.721890093	0.017862218	protein_coding
ENSG00000167617	CDC42EP5	-2.709711	0.003385132	protein_coding
ENSG00000179085	DPM3	-2.70476857	1.28E-09	protein_coding
ENSG00000196275	GTF2IRD2	-2.703390415	0.001197455	protein_coding
ENSG00000126583	PRKCG	-2.650246531	6.52E-06	protein_coding
ENSG00000220201	ZGLP1	-2.617005594	0.000391938	protein_coding
ENSG00000213977	TAX1BP3	-2.611755277	5.93E-05	protein_coding
ENSG00000128564	VGF	-2.603691343	0.000883969	protein_coding
ENSG00000181085	MAPK15	-2.592090822	2.46E-08	protein_coding
ENSG00000137474	MYO7A	-2.590991293	0.000246499	protein_coding
ENSG00000182472	CAPN12	-2.583699004	0.002275868	protein_coding
ENSG00000198502	HLA-DRB5	-2.571062232	0.000162828	protein_coding
ENSG00000185633	NDUFA4L2	-2.555772014	2.01E-05	protein_coding
ENSG00000185291	IL3RA	-2.55530542	9.06E-05	protein_coding
ENSG00000248712	CCDC153	-2.553278878	0.001184832	protein_coding
ENSG00000239282	CASTOR1	-2.550317664	0.010033723	protein_coding
ENSG00000147003	CLTRN	-2.550317664	0.010033723	protein_coding
ENSG00000142233	NTN5	-2.550317664	0.010033723	protein_coding
ENSG00000064787	BCAS1	-2.519911068	0.003149424	protein_coding
ENSG00000101222	SPEF1	-2.505653433	0.000258125	protein_coding
ENSG00000146700	SSC4D	-2.489214068	9.78E-05	protein_coding
ENSG00000170209	ANKK1	-2.483293713	0.041032031	protein_coding
ENSG00000151023	ENKUR	-2.483293713	0.041032031	protein_coding
ENSG00000101188	NTSR1	-2.483293713	0.041032031	protein_coding
ENSG00000170835	CEL	-2.478868536	0.000519431	protein_coding
ENSG00000008517	IL32	-2.463490553	0.014485426	protein_coding
ENSG00000133246	PRAM1	-2.463490553	0.014485426	protein_coding
ENSG00000185615	PDIA2	-2.449236765	1.98E-07	protein_coding
ENSG00000100359	SGSM3	-2.448531967	2.72E-06	protein_coding
ENSG00000072182	ASIC4	-2.442558694	0.00115274	protein_coding
ENSG00000214967	NPIPA7	-2.442558694	0.00115274	protein_coding
ENSG00000184351	KRTAP19-1	-2.43947051	0.000668181	protein_coding
ENSG00000229637	PRAC2	-2.435313356	0.000266845	protein_coding
ENSG00000008128	CDK11A	-2.431638192	4.03E-05	protein_coding
ENSG00000162755	KLHDC9	-2.430052228	2.44E-05	protein_coding
ENSG00000213199	ASIC3	-2.418613875	1.55E-07	protein_coding
ENSG00000185522	LMNTD2	-2.416072969	1.36E-06	protein_coding
ENSG00000163499	CRYBA2	-2.390523257	0.00288962	protein_coding
ENSG00000244242	IFITM10	-2.383194758	0.006072876	protein_coding

ENSG00000136732	GYPC	-2.374809761	1.02E-07	protein_coding
ENSG00000118307	CASC1	-2.371101304	0.020952111	protein_coding
ENSG00000250506	CDK3	-2.371101304	0.020952111	protein_coding
ENSG00000213999	MEF2B	-2.371101304	0.020952111	protein_coding
ENSG00000025423	HSD17B6	-2.365613353	7.51E-07	protein_coding
ENSG00000203896	LIME1	-2.362412098	2.85E-06	protein_coding
ENSG00000108309	RUNDC3A	-2.359029249	7.11E-05	protein_coding
ENSG00000262484	CCER2	-2.353385233	9.78E-05	protein_coding
ENSG00000198156	NPIPB6	-2.34659764	0.000336783	protein_coding
ENSG00000117407	ARTN	-2.337077196	3.46E-05	protein_coding
ENSG00000125995	ROMO1	-2.332029059	8.20E-08	protein_coding
ENSG00000114270	COL7A1	-2.330062313	2.01E-07	protein_coding
ENSG00000069696	DRD4	-2.323320272	1.08E-05	protein_coding
ENSG00000256061	DNAAF4	-2.309649736	0.008458934	protein_coding
ENSG00000255150	EID3	-2.309649736	0.008458934	protein_coding
ENSG00000130208	APOC1	-2.305615738	2.25E-07	protein_coding
ENSG00000222009	BTBD19	-2.272388225	0.030348148	protein_coding
ENSG00000172543	CTSW	-2.272388225	0.030348148	protein_coding
ENSG00000139914	FITM1	-2.272388225	0.030348148	protein_coding
ENSG00000121905	HPCA	-2.272388225	0.030348148	protein_coding
ENSG00000276600	RAB7B	-2.272388225	0.030348148	protein_coding
ENSG00000204389	HSPA1A	-2.265459839	5.79E-07	protein_coding
ENSG00000234776	C11orf94	-2.244078844	0.003489554	protein_coding
ENSG00000171703	TCEA2	-2.231089452	3.35E-07	protein_coding
ENSG00000182196	ARL6IP4	-2.219696813	0.000159888	protein_coding
ENSG00000241468	ATP5MF	-2.216325986	2.64E-07	protein_coding
ENSG00000142102	PGGHG	-2.205895487	3.99E-07	protein_coding
ENSG00000214212	C19orf38	-2.189878061	0.004621058	protein_coding
ENSG00000213123	TCTEX1D2	-2.177400242	0.003128224	protein_coding
ENSG00000089820	ARHGAP4	-2.170501849	1.38E-05	protein_coding
ENSG00000105219	CNTD2	-2.166421587	0.043991311	protein_coding
ENSG00000154545	MAGED4	-2.166421587	0.043991311	protein_coding
ENSG00000148826	NKX6-2	-2.166421587	0.043991311	protein_coding
ENSG00000213658	LAT	-2.1502564	0.016492881	protein_coding
ENSG00000153902	LGI4	-2.1502564	0.016492881	protein_coding
ENSG00000184678	HIST2H2BE	-2.1475694	5.01E-06	protein_coding
ENSG00000052344	PRSS8	-2.140304631	0.009732694	protein_coding
ENSG00000105672	ETV2	-2.138834945	0.000236256	protein_coding
ENSG00000142765	SYTL1	-2.131308576	1.80E-05	protein_coding
ENSG00000205593	DENND6B	-2.130583264	8.17E-06	protein_coding
ENSG00000184163	C1QTNF12	-2.125586431	8.17E-05	protein_coding
ENSG00000188573	FBLL1	-2.121303817	5.71E-05	protein_coding
ENSG00000187848	P2RX2	-2.111123709	0.000176356	protein_coding

ENSG00000221821	C6orf226	-2.109805748	0.000135269	protein_coding
ENSG00000160539	PLPP7	-2.106188001	4.56E-05	protein_coding
ENSG00000127415	IDUA	-2.10166063	7.18E-06	protein_coding
ENSG00000104899	AMH	-2.100895125	1.17E-06	protein_coding
ENSG00000266714	MYO15B	-2.095723623	1.11E-05	protein_coding
ENSG00000150281	CTF1	-2.090976439	0.000156638	protein_coding
ENSG00000183054	RGPD6	-2.080776579	0.00360343	protein_coding
ENSG00000138100	TRIM54	-2.078277573	0.005290723	protein_coding
ENSG00000101213	PTK6	-2.066901712	0.000284711	protein_coding
ENSG00000130203	APOE	-2.064591187	1.25E-05	protein_coding
ENSG00000188596	CFAP54	-2.063429289	0.032292239	protein_coding
ENSG00000198223	CSF2RA	-2.063429289	0.032292239	protein_coding
ENSG00000205560	CPT1B	-2.059590851	0.000498242	protein_coding
ENSG00000187479	C11orf96	-2.057365248	3.49E-05	protein_coding
ENSG00000100429	HDAC10	-2.057365248	3.49E-05	protein_coding
ENSG00000119632	IFI27L2	-2.052632112	1.37E-05	protein_coding
ENSG00000130707	ASS1	-2.048518379	1.93E-06	protein_coding
ENSG00000205155	PSENEN	-2.044559931	7.15E-06	protein_coding
ENSG00000100031	GGT1	-2.038489626	5.95E-05	protein_coding
ENSG00000260428	SCX	-2.035147809	0.004613369	protein_coding
ENSG00000184838	PRR16	-2.026039549	0.006891763	protein_coding
ENSG00000137101	CD72	-2.025391811	0.000931128	protein_coding
ENSG00000221916	C19orf73	-2.025122134	0.000334323	protein_coding
ENSG00000215440	NPEPL1	-2.024663972	1.95E-05	protein_coding
ENSG00000142530	FAM71E1	-2.021347741	2.78E-05	protein_coding
ENSG00000103145	HCFC1R1	-2.01753908	4.90E-06	protein_coding
ENSG00000159588	CCDC17	-2.013869835	0.010811714	protein_coding
ENSG00000127588	GNG13	-2.013622696	3.61E-05	protein_coding
ENSG00000127578	WFIKKN1	-2.002796114	0.000392948	protein_coding
ENSG00000135502	SLC26A10	-2.001477623	7.03E-05	protein_coding
ENSG00000140675	SLC5A2	-2.000546585	0.004060649	protein_coding
ENSG00000012171	SEMA3B	-1.994819138	2.48E-05	protein_coding
ENSG00000205143	ARID3C	-1.988028651	0.005911861	protein_coding
ENSG00000176973	FAM89B	-1.987805988	3.19E-05	protein_coding
ENSG00000177106	EPS8L2	-1.983445534	0.000189057	protein_coding
ENSG00000007952	NOX1	-1.980119157	0.000462155	protein_coding
ENSG00000135441	BLOC1S1	-1.976320667	1.84E-05	protein_coding
ENSG00000257704	INAFM1	-1.972842091	1.73E-05	protein_coding
ENSG00000232388	SMIM26	-1.972335978	6.52E-06	protein_coding
ENSG00000184697	CLDN6	-1.971838766	0.00898543	protein_coding
ENSG00000244731	C4A	-1.97104004	0.045202178	protein_coding
ENSG00000089199	CHGB	-1.97104004	0.045202178	protein_coding
ENSG00000130427	EPO	-1.97104004	0.045202178	protein_coding

ENSG00000163497	FEV	-1.97104004	0.045202178	protein_coding
ENSG00000182687	GALR2	-1.97104004	0.045202178	protein_coding
ENSG00000243708	PLA2G4B	-1.97104004	0.045202178	protein_coding
ENSG00000176978	DPP7	-1.959427296	1.08E-05	protein_coding
ENSG00000143369	ECM1	-1.95763255	0.00512978	protein_coding
ENSG00000165695	AK8	-1.957080052	0.000543898	protein_coding
ENSG00000089692	LAG3	-1.950081899	0.014380224	protein_coding
ENSG00000206140	TMEM191C	-1.948458168	0.0032271	protein_coding
ENSG00000129673	AANAT	-1.939318447	0.007582043	protein_coding
ENSG00000164405	UQCRQ	-1.934688244	5.68E-06	protein_coding
ENSG00000197568	HHLA3	-1.927271129	1.88E-05	protein_coding
ENSG00000182685	BRICD5	-1.920286768	0.000158397	protein_coding
ENSG00000197530	MIB2	-1.907208648	9.19E-06	protein_coding
ENSG00000182809	CRIP2	-1.892734611	9.90E-05	protein_coding
ENSG00000100604	CHGA	-1.892262009	0.00561466	protein_coding
ENSG00000074660	SCARF1	-1.892262009	0.00561466	protein_coding
ENSG00000100097	LGALS1	-1.890924613	0.000412542	protein_coding
ENSG00000135409	AMHR2	-1.888905982	0.009730816	protein_coding
ENSG00000188243	COMMD6	-1.8887785	1.21E-05	protein_coding
ENSG00000105699	LSR	-1.887594566	1.31E-05	protein_coding
ENSG00000165349	SLC7A3	-1.884028644	2.80E-05	protein_coding
ENSG00000170458	CD14	-1.88334262	0.019137366	protein_coding
ENSG00000188672	RHCE	-1.88334262	0.019137366	protein_coding
ENSG00000158815	FGF17	-1.880802338	0.000552409	protein_coding
ENSG00000125611	CHCHD5	-1.880230021	1.95E-05	protein_coding
ENSG00000184381	PLA2G6	-1.873834913	2.44E-05	protein_coding
ENSG00000169020	ATP5ME	-1.870229522	1.63E-05	protein_coding
ENSG00000152082	MZT2B	-1.8669941	1.25E-05	protein_coding
ENSG00000240583	AQP1	-1.859842361	0.002617946	protein_coding
ENSG00000100399	CHADL	-1.856917147	0.015305394	protein_coding
ENSG00000187688	TRPV2	-1.856917147	0.015305394	protein_coding
ENSG00000134825	TMEM258	-1.856210634	1.41E-05	protein_coding
ENSG00000239779	WBP1	-1.853602288	3.88E-05	protein_coding
ENSG00000126759	CFP	-1.850587697	0.00701327	protein_coding
ENSG00000105696	TMEM59L	-1.847985297	0.000257696	protein_coding
ENSG00000185201	IFITM2	-1.845006547	9.10E-05	protein_coding
ENSG00000215788	TNFRSF25	-1.844799823	0.000131231	protein_coding
ENSG00000173762	CD7	-1.837389242	0.033376343	protein_coding
ENSG00000124159	MATN4	-1.837389242	0.033376343	protein_coding
ENSG00000179954	SSC5D	-1.837389242	0.033376343	protein_coding
ENSG00000261408	TEN1-CDK3	-1.836144045	0.002183911	protein_coding
ENSG00000183617	MRPL54	-1.83572062	1.87E-05	protein_coding
ENSG00000166595	CIAO2B	-1.818349915	2.28E-05	protein_coding

ENSG00000078018	MAP2	-1.813365589	0.025475297	protein_coding
ENSG00000178226	PRSS36	-1.813365589	0.025475297	protein_coding
ENSG00000149575	SCN2B	-1.813365589	0.025475297	protein_coding
ENSG00000105404	RABAC1	-1.812559449	4.33E-05	protein_coding
ENSG00000182326	C1S	-1.811740233	0.000679363	protein_coding
ENSG00000254995	STX16-NPEPL1	-1.810539058	0.0014637	protein_coding
ENSG00000233198	RNF224	-1.807673662	0.008764824	protein_coding
ENSG00000140400	MAN2C1	-1.798478435	2.46E-05	protein_coding
ENSG00000198918	RPL39	-1.797595829	2.13E-05	protein_coding
ENSG00000179698	WDR97	-1.796937385	0.007498916	protein_coding
ENSG00000273154	AL121845.3	-1.795830541	0.019987884	protein_coding
ENSG00000092295	TGM1	-1.782783081	0.001103785	protein_coding
ENSG00000100312	ACR	-1.782467175	0.016051556	protein_coding
ENSG00000105472	CLEC11A	-1.782379277	7.03E-05	protein_coding
ENSG00000160888	IER2	-1.779166811	3.22E-05	protein_coding
ENSG00000163453	IGFBP7	-1.77825559	0.000675908	protein_coding
ENSG00000183011	NAA38	-1.777318329	3.05E-05	protein_coding
ENSG00000095059	DHPS	-1.773175841	0.000158409	protein_coding
ENSG00000101282	RSPO4	-1.771944636	0.013149317	protein_coding
ENSG00000198258	UBL5	-1.767708205	3.19E-05	protein_coding
ENSG00000231389	HLA-DPA1	-1.767217061	0.000114831	protein_coding
ENSG00000186352	ANKRD37	-1.76344388	0.010958385	protein_coding
ENSG00000112695	COX7A2	-1.756794141	3.46E-05	protein_coding
ENSG00000123358	NR4A1	-1.745893606	4.88E-05	protein_coding
ENSG00000054148	PHPT1	-1.743324743	4.04E-05	protein_coding
ENSG00000134940	ACRV1	-1.739820566	0.033910222	protein_coding
ENSG00000179057	IGSF22	-1.739820566	0.033910222	protein_coding
ENSG00000170906	NDUFA3	-1.737486303	5.03E-05	protein_coding
ENSG00000196436	NPIPB15	-1.736895585	0.001306375	protein_coding
ENSG00000142046	TMEM91	-1.736895585	0.001306375	protein_coding
ENSG00000177595	PIDD1	-1.735940904	4.87E-05	protein_coding
ENSG00000260001	TGFBR3L	-1.734195283	0.002638872	protein_coding
ENSG00000139410	SDSL	-1.730934173	0.000330356	protein_coding
ENSG00000204427	ABHD16A	-1.728681866	0.002229387	protein_coding
ENSG00000111653	ING4	-1.727831088	9.32E-05	protein_coding
ENSG00000105755	ETHE1	-1.723926572	0.000236059	protein_coding
ENSG00000042493	CAPG	-1.722469002	0.000128574	protein_coding
ENSG00000144908	ALDH1L1	-1.721532171	0.016654638	protein_coding
ENSG00000163354	DCST2	-1.721532171	0.016654638	protein_coding
ENSG00000163873	GRIK3	-1.721532171	0.016654638	protein_coding
ENSG00000228253	MT-ATP8	-1.720496336	7.67E-05	protein_coding
ENSG00000182902	SLC25A18	-1.71781511	0.013705036	protein_coding

ENSG00000172346	CSDC2	-1.717561594	0.001490418	protein_coding
ENSG00000151366	NDUFC2	-1.71615386	7.91E-05	protein_coding
ENSG00000243449	C4orf48	-1.715722509	6.42E-05	protein_coding
ENSG00000135940	COX5B	-1.715000509	5.08E-05	protein_coding
ENSG00000134595	SOX3	-1.712201589	0.006068669	protein_coding
ENSG00000007255	TRAPPC6A	-1.712145525	8.89E-05	protein_coding
ENSG00000108602	ALDH3A1	-1.708163774	0.004688294	protein_coding
ENSG00000250067	YJEFN3	-1.707410614	0.000175395	protein_coding
ENSG00000197756	RPL37A	-1.703614366	5.35E-05	protein_coding
ENSG00000130255	RPL36	-1.701486858	5.47E-05	protein_coding
ENSG00000213741	RPS29	-1.696537896	5.88E-05	protein_coding
ENSG00000134419	RPS15A	-1.689364183	6.36E-05	protein_coding
ENSG00000148450	MSRB2	-1.682721601	0.000125718	protein_coding
ENSG00000189171	S100A13	-1.682680147	0.000126994	protein_coding
ENSG00000170961	HAS2	-1.679871769	0.000884383	protein_coding
ENSG00000085514	PILRA	-1.677339809	0.002399103	protein_coding
ENSG00000172803	SNX32	-1.675913736	0.003389794	protein_coding
ENSG00000175701	MTLN	-1.674012113	0.000186426	protein_coding
ENSG00000153093	ACOXL	-1.672803563	0.007498916	protein_coding
ENSG00000172638	EFEMP2	-1.671844827	0.01417872	protein_coding
ENSG00000111432	FZD10	-1.671756898	0.000162217	protein_coding
ENSG00000160282	FTCD	-1.670695952	0.017143019	protein_coding
ENSG00000185885	IFITM1	-1.667800528	0.000309601	protein_coding
ENSG00000149926	FAM57B	-1.665303326	0.034088382	protein_coding
ENSG00000147036	LANCL3	-1.665303326	0.034088382	protein_coding
ENSG00000116785	CFHR3	-1.662324062	0.045117465	protein_coding
ENSG00000276180	HIST1H4I	-1.660416828	0.00015472	protein_coding
ENSG00000108465	CDK5RAP3	-1.651310534	0.000145216	protein_coding
ENSG00000155428	TRIM74	-1.651220856	0.00330135	protein_coding
ENSG00000204839	MROH6	-1.648302618	0.000496612	protein_coding
ENSG00000167103	PIP5KL1	-1.642674236	0.001830438	protein_coding
ENSG00000197191	CYSRT1	-1.637229005	0.000762609	protein_coding
ENSG00000186395	KRT10	-1.636999699	0.00013948	protein_coding
ENSG00000076944	STXBP2	-1.632970329	0.000162218	protein_coding
ENSG00000172809	RPL38	-1.62904977	0.0001094	protein_coding
ENSG00000239382	ALKBH6	-1.628460522	0.001834907	protein_coding
ENSG00000105290	APLP1	-1.628245419	0.000208019	protein_coding
ENSG00000131097	HIGD1B	-1.627615045	0.017539069	protein_coding
ENSG00000198892	SHISA4	-1.627615045	0.017539069	protein_coding
ENSG00000172428	COPS9	-1.6239771	0.000138578	protein_coding
ENSG00000177600	RPLP2	-1.622631749	0.000114942	protein_coding
ENSG00000188372	ZP3	-1.622005393	0.004272904	protein_coding
ENSG00000140264	SERF2	-1.620717571	0.000123894	protein_coding

ENSG00000160323	ADAMTS13	-1.620624266	0.000178751	protein_coding
ENSG00000158373	HIST1H2BD	-1.619514	0.002684397	protein_coding
ENSG00000131400	NAPSA	-1.615093364	0.026720022	protein_coding
ENSG00000183889	PKD1P1	-1.615093364	0.026720022	protein_coding
ENSG00000163191	S100A11	-1.614105219	0.000154599	protein_coding
ENSG00000177954	RPS27	-1.610913073	0.000128493	protein_coding
ENSG00000105258	POLR2I	-1.609934366	0.000222837	protein_coding
ENSG00000204444	APOM	-1.609508286	0.007259967	protein_coding
ENSG00000185482	STAC3	-1.60318844	0.004091611	protein_coding
ENSG00000110492	MDK	-1.601169095	0.000148524	protein_coding
ENSG00000137818	RPLP1	-1.599340392	0.000141576	protein_coding
ENSG00000160781	PAQR6	-1.596121351	0.000277794	protein_coding
ENSG00000198576	ARC	-1.594565012	0.001027208	protein_coding
ENSG00000174886	NDUFA11	-1.593788572	0.000218501	protein_coding
ENSG00000171858	RPS21	-1.592580809	0.000154168	protein_coding
ENSG00000128218	VPREB3	-1.59062504	0.017860441	protein_coding
ENSG00000128298	BAIAP2L2	-1.588663836	0.005639186	protein_coding
ENSG00000132635	PCED1A	-1.585699554	0.000189297	protein_coding
ENSG00000160948	VPS28	-1.583862023	0.000193508	protein_coding
ENSG00000149599	DUSP15	-1.583434831	0.001114197	protein_coding
ENSG00000111775	COX6A1	-1.582496006	0.000182669	protein_coding
ENSG00000143303	RRNAD1	-1.580068389	0.000238503	protein_coding
ENSG00000107874	CUEDC2	-1.5779055	0.000198178	protein_coding
ENSG00000062524	LTK	-1.577873532	0.000624485	protein_coding
ENSG00000120075	HOXB5	-1.575852226	0.000384392	protein_coding
ENSG00000106400	ZNHIT1	-1.574026991	0.000196892	protein_coding
ENSG00000163344	PMVK	-1.573230797	0.000223981	protein_coding
ENSG00000176476	SGF29	-1.57236758	0.000276105	protein_coding
ENSG00000100889	PCK2	-1.571972216	0.000201043	protein_coding
ENSG00000174938	SEZ6L2	-1.571693106	0.000228324	protein_coding
ENSG00000174917	C19orf70	-1.570199641	0.000213713	protein_coding
ENSG00000196296	ATP2A1	-1.567902722	0.000863527	protein_coding
ENSG00000089847	ANKRD24	-1.567715484	0.010138421	protein_coding
ENSG00000089163	SIRT4	-1.567715484	0.010138421	protein_coding
ENSG00000115257	PCSK4	-1.566586763	0.000909076	protein_coding
ENSG00000063180	CA11	-1.566141341	0.00030809	protein_coding
ENSG00000176894	PXMP2	-1.564934498	0.000585308	protein_coding
ENSG00000187961	KLHL17	-1.564433817	0.000232465	protein_coding
ENSG00000173272	MZT2A	-1.564226095	0.000206742	protein_coding
ENSG00000274386	TMEM269	-1.564174743	0.004529785	protein_coding
ENSG00000181218	HIST3H2A	-1.560985963	0.00025556	protein_coding
ENSG00000115596	WNT6	-1.558776549	0.033832332	protein_coding
ENSG00000143994	ABHD1	-1.558367201	0.006686163	protein_coding

ENSG00000185499	MUC1	-1.558367201	0.006686163	protein_coding
ENSG00000124140	SLC12A5	-1.558367201	0.006686163	protein_coding
ENSG00000161265	U2AF1L4	-1.558141548	0.002517475	protein_coding
ENSG00000105538	RASIP1	-1.556362233	0.000714343	protein_coding
ENSG00000130066	SAT1	-1.555929279	0.00026419	protein_coding
ENSG00000204435	CSNK2B	-1.55329927	0.000363062	protein_coding
ENSG00000188368	PRR19	-1.551769023	0.005549857	protein_coding
ENSG00000182154	MRPL41	-1.550611296	0.000242333	protein_coding
ENSG00000189283	FHIT	-1.55053097	0.000694733	protein_coding
ENSG00000175894	TSPEAR	-1.547711006	0.021872151	protein_coding
ENSG00000117480	FAAH	-1.543176996	0.001073018	protein_coding
ENSG00000135636	DYSF	-1.54099257	0.010511842	protein_coding
ENSG00000196126	HLA-DRB1	-1.540588284	0.001044809	protein_coding
ENSG00000187840	EIF4EBP1	-1.539702069	0.00025014	protein_coding
ENSG00000187608	ISG15	-1.53858666	0.000635717	protein_coding
ENSG00000129559	NEDD8	-1.538339291	0.000304925	protein_coding
ENSG00000171160	MORN4	-1.537582773	0.000453295	protein_coding
ENSG00000125652	ALKBH7	-1.536862458	0.000328104	protein_coding
ENSG00000138495	COX17	-1.531130278	0.000341726	protein_coding
ENSG00000105379	ETFB	-1.530867428	0.000366743	protein_coding
ENSG00000163666	HESX1	-1.530801392	0.004943748	protein_coding
ENSG00000229117	RPL41	-1.530419129	0.000264326	protein_coding
ENSG00000125730	C3	-1.530357944	0.012302827	protein_coding
ENSG00000182870	GALNT9	-1.530357944	0.012302827	protein_coding
ENSG00000102057	KCND1	-1.530357944	0.012302827	protein_coding
ENSG00000272899	ATP6V1FNB	-1.527420664	0.009406001	protein_coding
ENSG00000213398	LCAT	-1.52737361	0.003420538	protein_coding
ENSG00000162300	ZFPL1	-1.526141516	0.000672463	protein_coding
ENSG00000119650	IFT43	-1.524792124	0.00045964	protein_coding
ENSG00000102886	GDPD3	-1.523823861	0.005443567	protein_coding
ENSG00000154134	ROBO3	-1.520828897	0.000452963	protein_coding
ENSG00000259803	SLC22A31	-1.51956634	0.001213375	protein_coding
ENSG00000203780	FANK1	-1.519335259	0.033533086	protein_coding
ENSG00000151715	TMEM45B	-1.519335259	0.033533086	protein_coding
ENSG00000189143	CLDN4	-1.517169485	0.010850785	protein_coding
ENSG00000137880	GCHFR	-1.517169485	0.010850785	protein_coding
ENSG00000185324	CDK10	-1.514245549	0.000349292	protein_coding
ENSG00000090006	LTBP4	-1.513728975	0.000333753	protein_coding
ENSG00000141552	ANAPC11	-1.512929449	0.000334065	protein_coding
ENSG00000203724	C1orf53	-1.512204134	0.000922867	protein_coding
ENSG00000130748	TMEM160	-1.510443252	0.000383742	protein_coding
ENSG00000185236	RAB11B	-1.507904792	0.000373044	protein_coding
ENSG00000198467	TPM2	-1.507841291	0.000746859	protein_coding

ENSG00000169627	BOLA2B	-1.506806644	0.008232207	protein_coding
ENSG00000011028	MRC2	-1.504915076	0.00043781	protein_coding
ENSG00000173818	ENDOV	-1.504104329	0.000592747	protein_coding
ENSG00000104814	MAP4K1	-1.503117869	0.004137476	protein_coding
ENSG00000185838	GNB1L	-1.498752582	0.000438605	protein_coding
ENSG00000143545	RAB13	-1.498261433	0.000869728	protein_coding
ENSG00000012822	CALCOCO1	-1.498094737	0.000465108	protein_coding
ENSG00000149922	TBX6	-1.496273701	0.005055914	protein_coding
ENSG00000135519	KCNH3	-1.495517403	0.002050888	protein_coding
ENSG00000160200	CBS	-1.494426394	0.000409673	protein_coding
ENSG00000103056	SMPD3	-1.492007286	0.014931034	protein_coding
ENSG00000235034	C19orf81	-1.486657059	0.001216383	protein_coding
ENSG00000015413	DPEP1	-1.486156913	0.033173684	protein_coding
ENSG00000183793	NPIPA5	-1.485798414	0.006469214	protein_coding
ENSG00000196683	TOMM7	-1.485713848	0.000429236	protein_coding
ENSG00000130653	PNPLA7	-1.484659843	0.002383913	protein_coding
ENSG00000205544	TMEM256	-1.484492922	0.000656602	protein_coding
ENSG00000163156	SCNM1	-1.484453203	0.000464144	protein_coding
ENSG00000188290	HES4	-1.484151289	0.000414045	protein_coding
ENSG00000205670	SMIM11A	-1.483318328	0.01296303	protein_coding
ENSG00000131495	NDUFA2	-1.482088145	0.00049504	protein_coding
ENSG00000185189	NRBP2	-1.48076292	0.000629961	protein_coding
ENSG00000205309	NT5M	-1.479633788	0.000930458	protein_coding
ENSG00000165698	SPACA9	-1.476883277	0.001217772	protein_coding
ENSG00000136371	MTHFS	-1.476782421	0.002474866	protein_coding
ENSG00000107147	KCNT1	-1.476331008	0.017860441	protein_coding
ENSG00000112667	DNPH1	-1.474994878	0.000446185	protein_coding
ENSG00000126562	WNK4	-1.47357435	0.004109782	protein_coding
ENSG00000113196	HAND1	-1.473274709	0.000537223	protein_coding
ENSG00000165233	CARD19	-1.472881525	0.000673874	protein_coding
ENSG00000162148	PPP1R32	-1.472216885	0.006676975	protein_coding
ENSG00000125356	NDUFA1	-1.471831609	0.00047381	protein_coding
ENSG00000135929	CYP27A1	-1.471031328	0.008781446	protein_coding
ENSG00000118231	CRYGD	-1.468686557	0.001770006	protein_coding
ENSG00000196976	LAGE3	-1.46827679	0.00048094	protein_coding
ENSG00000142871	CYR61	-1.467471418	0.000566016	protein_coding
ENSG00000105619	TFPT	-1.467251953	0.00082603	protein_coding
ENSG00000108107	RPL28	-1.466520889	0.000465637	protein_coding
ENSG00000197982	C1orf122	-1.465667505	0.000559456	protein_coding
ENSG00000109743	BST1	-1.465134476	0.041903977	protein_coding
ENSG00000160318	CLDND2	-1.465134476	0.041903977	protein_coding
ENSG00000185883	ATP6V0C	-1.465017117	0.000927317	protein_coding
ENSG00000224420	ADM5	-1.46492182	0.001318982	protein_coding

ENSG00000167702	KIFC2	-1.464576319	0.000628857	protein_coding
ENSG00000176340	COX8A	-1.46415266	0.000499128	protein_coding
ENSG00000088881	EBF4	-1.461864963	0.000733422	protein_coding
ENSG00000126458	RRAS	-1.46160644	0.001575434	protein_coding
ENSG00000203950	RTL8A	-1.45945732	0.000537625	protein_coding
ENSG00000099377	HSD3B7	-1.459019104	0.009979849	protein_coding
ENSG00000063177	RPL18	-1.458828463	0.000493444	protein_coding
ENSG00000184343	SRPK3	-1.457981228	0.001947286	protein_coding
ENSG00000125813	PAX1	-1.457852453	0.032778547	protein_coding
ENSG00000180089	TMEM86B	-1.457852453	0.032778547	protein_coding
ENSG00000242114	MTFP1	-1.455832957	0.001929017	protein_coding
ENSG00000110719	TCIRG1	-1.454899025	0.001009041	protein_coding
ENSG00000151651	ADAM8	-1.454508048	0.002304491	protein_coding
ENSG00000162004	CCDC78	-1.453147717	0.001141424	protein_coding
ENSG00000162032	SPSB3	-1.45260926	0.018121122	protein_coding
ENSG00000181029	TRAPPC5	-1.45047514	0.004891669	protein_coding
ENSG00000125971	DYNLRB1	-1.450467735	0.000574406	protein_coding
ENSG00000160783	PMF1	-1.450250691	0.000669127	protein_coding
ENSG00000131094	C1QL1	-1.449811911	0.000946334	protein_coding
ENSG00000188729	OSTN	-1.448899505	0.00161946	protein_coding
ENSG00000147123	NDUFB11	-1.448014145	0.000570403	protein_coding
ENSG00000163923	RPL39L	-1.447792284	0.000953096	protein_coding
ENSG00000131187	F12	-1.446546247	0.000832559	protein_coding
ENSG00000167526	RPL13	-1.445207593	0.000552924	protein_coding
ENSG00000171222	SCAND1	-1.442929359	0.000668778	protein_coding
ENSG00000130649	CYP2E1	-1.442647348	0.00241602	protein_coding
ENSG00000225663	MCRIP1	-1.44207422	0.00064399	protein_coding
ENSG00000125434	SLC25A35	-1.442073368	0.001655274	protein_coding
ENSG00000158473	CD1D	-1.441556097	0.005033362	protein_coding
ENSG00000151006	PRSS53	-1.440424186	0.004816357	protein_coding
ENSG00000246922	UBAP1L	-1.439346302	0.008470153	protein_coding
ENSG00000100319	ZMAT5	-1.438005102	0.000947781	protein_coding
ENSG00000173917	HOXB2	-1.437799322	0.001110202	protein_coding
ENSG00000170634	ACYP2	-1.437545924	0.002483067	protein_coding
ENSG00000111012	CYP27B1	-1.436501877	0.001146867	protein_coding
ENSG00000100271	TTLL1	-1.436118038	0.001392619	protein_coding
ENSG00000075826	SEC31B	-1.434650522	0.001055181	protein_coding
ENSG00000137100	DCTN3	-1.432650576	0.000700264	protein_coding
ENSG00000130724	CHMP2A	-1.431160474	0.000735367	protein_coding
ENSG00000085465	OVGP1	-1.429894912	0.001224114	protein_coding
ENSG00000173486	FKBP2	-1.427509404	0.000763876	protein_coding
ENSG00000126524	SBDS	-1.425776866	0.000783149	protein_coding
ENSG00000116586	LAMTOR2	-1.424661734	0.000768614	protein_coding

ENSG00000006282	SPATA20	-1.422111957	0.000978713	protein_coding
ENSG00000228594	FNDC10	-1.421684359	0.00079817	protein_coding
ENSG00000184903	IMMP2L	-1.41806425	0.003349499	protein_coding
ENSG00000197903	HIST1H2BK	-1.417655404	0.000859523	protein_coding
ENSG00000136942	RPL35	-1.417260715	0.000703921	protein_coding
ENSG00000133243	BTBD2	-1.41675855	0.000735951	protein_coding
ENSG00000105048	TNNT1	-1.416676729	0.000933168	protein_coding
ENSG00000285043	AC093512.2	-1.415547057	0.009693408	protein_coding
ENSG00000126432	PRDX5	-1.414674515	0.000744723	protein_coding
ENSG00000126267	COX6B1	-1.411460689	0.000785783	protein_coding
ENSG00000183114	FAM43B	-1.411005328	0.002258129	protein_coding
ENSG00000171786	NHLH1	-1.410733295	0.040101413	protein_coding
ENSG00000182379	NXPH4	-1.410103819	0.000963774	protein_coding
ENSG00000163702	IL17RC	-1.406564387	0.001219274	protein_coding
ENSG00000090238	YPEL3	-1.405765192	0.001742836	protein_coding
ENSG00000145494	NDUFS6	-1.404898875	0.00083829	protein_coding
ENSG00000158106	RHPN1	-1.404543375	0.001032848	protein_coding
ENSG00000059915	PSD	-1.404174802	0.009889231	protein_coding
ENSG00000240053	LY6G5B	-1.403096446	0.003220672	protein_coding
ENSG00000183172	SMDT1	-1.402578778	0.000977262	protein_coding
ENSG00000126768	TIMM17B	-1.401424172	0.000856325	protein_coding
ENSG00000103253	HAGHL	-1.400809585	0.001050881	protein_coding
ENSG00000173531	MST1	-1.400173326	0.001573704	protein_coding
ENSG00000212123	PRR22	-1.399047271	0.001289165	protein_coding
ENSG00000151778	SERP2	-1.398461208	0.004047714	protein_coding
ENSG00000146066	HIGD2A	-1.396158153	0.000958195	protein_coding
ENSG00000162572	SCNN1D	-1.396005259	0.001810352	protein_coding
ENSG00000185262	UBALD2	-1.393471307	0.001029758	protein_coding
ENSG00000172037	LAMB2	-1.392733149	0.000963362	protein_coding
ENSG00000157514	TSC22D3	-1.392216186	0.000884929	protein_coding
ENSG00000171443	ZNF524	-1.39009716	0.002036645	protein_coding
ENSG00000102901	CENPT	-1.39008504	0.000973415	protein_coding
ENSG00000041880	PARP3	-1.389987802	0.001649724	protein_coding
ENSG00000231500	RPS18	-1.389305089	0.000886152	protein_coding
ENSG00000149743	TRPT1	-1.38879576	0.0015099	protein_coding
ENSG00000009950	MLXIPL	-1.388034983	0.00129015	protein_coding
ENSG00000105223	PLD3	-1.387912777	0.000937175	protein_coding
ENSG00000104863	LIN7B	-1.38737749	0.002473919	protein_coding
ENSG00000104859	CLASRP	-1.38679681	0.000992451	protein_coding
ENSG00000089327	FXYD5	-1.386708792	0.001193527	protein_coding
ENSG00000169169	CPT1C	-1.386270259	0.002008881	protein_coding
ENSG00000178531	CTXN1	-1.386181324	0.001126986	protein_coding
ENSG00000090539	CHRD	-1.385419305	0.00440473	protein_coding

ENSG00000182310	SPACA6	-1.385014088	0.003531724	protein_coding
ENSG00000178449	COX14	-1.383207857	0.001209883	protein_coding
ENSG00000179862	CITED4	-1.382503926	0.003698436	protein_coding
ENSG00000013364	MVP	-1.379022547	0.002049499	protein_coding
ENSG00000164713	BRI3	-1.377706652	0.001136919	protein_coding
ENSG00000065621	GSTO2	-1.377406221	0.002478246	protein_coding
ENSG00000010361	FUZ	-1.375847955	0.001196592	protein_coding
ENSG00000133250	ZNF414	-1.375773952	0.001287211	protein_coding
ENSG00000002330	BAD	-1.375570562	0.001298888	protein_coding
ENSG00000196502	SULT1A1	-1.373840932	0.002897627	protein_coding
ENSG00000188818	ZDHHC11	-1.373840932	0.002897627	protein_coding
ENSG00000204248	COL11A2	-1.37359296	0.006842984	protein_coding
ENSG00000004777	ARHGAP33	-1.372818828	0.001933081	protein_coding
ENSG00000171159	C9orf16	-1.372360547	0.001203407	protein_coding
ENSG00000183426	NPIPA1	-1.372240917	0.002226046	protein_coding
ENSG00000143543	JTB	-1.371211763	0.00151604	protein_coding
ENSG00000178685	PARP10	-1.370430737	0.026682384	protein_coding
ENSG00000099795	NDUFB7	-1.369124652	0.00113534	protein_coding
ENSG00000115226	FNDC4	-1.367885676	0.001787151	protein_coding
ENSG00000160972	PPP1R16A	-1.367215854	0.001473017	protein_coding
ENSG00000100417	PMM1	-1.365865253	0.001485799	protein_coding
ENSG00000187118	CMC1	-1.365847255	0.001517031	protein_coding
ENSG00000034510	TMSB10	-1.365775013	0.001097412	protein_coding
ENSG00000203778	FAM229B	-1.365449213	0.001811129	protein_coding
ENSG00000182899	RPL35A	-1.365416136	0.001087602	protein_coding
ENSG00000173113	TRMT112	-1.363576878	0.001136297	protein_coding
ENSG00000110148	CCKBR	-1.362023091	0.049028188	protein_coding
ENSG00000166816	LDHD	-1.361871214	0.022113468	protein_coding
ENSG00000125744	RTN2	-1.361542892	0.002021001	protein_coding
ENSG00000143314	MRPL24	-1.361176419	0.001206495	protein_coding
ENSG00000158062	UBXN11	-1.360907022	0.001426827	protein_coding
ENSG00000185100	ADSSL1	-1.360163992	0.002982967	protein_coding
ENSG00000099330	OCEL1	-1.359599913	0.002359281	protein_coding
ENSG00000100300	TSPO	-1.357325871	0.001202777	protein_coding
ENSG00000167969	ECI1	-1.354714645	0.001226619	protein_coding
ENSG00000111716	LDHB	-1.354436208	0.001290493	protein_coding
ENSG00000105640	RPL18A	-1.352461095	0.001200879	protein_coding
ENSG00000127419	TMEM175	-1.351984329	0.001702886	protein_coding
ENSG00000167508	MVD	-1.351548501	0.001349208	protein_coding
ENSG00000198937	CCDC167	-1.351309221	0.001540232	protein_coding
ENSG00000129991	TNNI3	-1.350074039	0.010860269	protein_coding
ENSG00000163064	EN1	-1.350035582	0.003460177	protein_coding
ENSG00000119227	PIGZ	-1.349322788	0.008095347	protein_coding

ENSG00000104823	ECH1	-1.3490256	0.001359375	protein_coding
ENSG00000172456	FGGY	-1.348826366	0.005440345	protein_coding
ENSG00000197757	HOXC6	-1.347883091	0.001826722	protein_coding
ENSG00000180953	ST20	-1.347529739	0.009128294	protein_coding
ENSG00000159335	PTMS	-1.346832811	0.001296777	protein_coding
ENSG00000072778	ACADVL	-1.346361777	0.001358866	protein_coding
ENSG00000103363	ELOB	-1.346165859	0.001308455	protein_coding
ENSG00000172183	ISG20	-1.345117628	0.011889469	protein_coding
ENSG00000225828	FAM229A	-1.343918902	0.002005095	protein_coding
ENSG00000126467	TSKS	-1.343558421	0.032778547	protein_coding
ENSG00000105443	CYTH2	-1.342024005	0.001507714	protein_coding
ENSG00000133134	BEX2	-1.341364865	0.001481066	protein_coding
ENSG00000105372	RPS19	-1.340368151	0.001322893	protein_coding
ENSG00000175482	POLD4	-1.339363656	0.026398783	protein_coding
ENSG00000186648	CARMIL3	-1.338082401	0.00833992	protein_coding
ENSG00000198546	ZNF511	-1.337895684	0.001829747	protein_coding
ENSG00000127152	BCL11B	-1.336578515	0.004969131	protein_coding
ENSG00000197558	SSPO	-1.336230426	0.00351424	protein_coding
ENSG00000198003	CCDC151	-1.336067689	0.009997001	protein_coding
ENSG00000188747	NOXA1	-1.335337672	0.00249085	protein_coding
ENSG00000099797	TECR	-1.335089869	0.001429955	protein_coding
ENSG00000228474	OST4	-1.334914745	0.001556795	protein_coding
ENSG00000105519	CAPS	-1.334811061	0.009396064	protein_coding
ENSG00000228439	TSTD3	-1.334370282	0.016452689	protein_coding
ENSG00000115286	NDUFS7	-1.332925607	0.001661903	protein_coding
ENSG00000160213	CSTB	-1.332511324	0.001506865	protein_coding
ENSG00000167619	TMEM145	-1.331225384	0.002977635	protein_coding
ENSG00000123689	G0S2	-1.32780817	0.0070897	protein_coding
ENSG00000130313	PGLS	-1.327746553	0.001707925	protein_coding
ENSG00000197093	GAL3ST4	-1.327516702	0.032364314	protein_coding
ENSG00000100246	DNAL4	-1.327382612	0.002144444	protein_coding
ENSG00000106211	HSPB1	-1.326538494	0.001670738	protein_coding
ENSG00000124587	PEX6	-1.326008792	0.00245964	protein_coding
ENSG00000078804	TP53INP2	-1.324968116	0.003020308	protein_coding
ENSG00000196998	WDR45	-1.324477683	0.001754918	protein_coding
ENSG00000228300	C19orf24	-1.323602912	0.001722139	protein_coding
ENSG00000184470	TXNRD2	-1.323568961	0.001649686	protein_coding
ENSG00000110711	AIP	-1.322494641	0.001664892	protein_coding
ENSG00000074842	MYDGF	-1.322048935	0.001598872	protein_coding
ENSG00000131584	ACAP3	-1.321691824	0.001705969	protein_coding
ENSG00000160284	SPATC1L	-1.320370408	0.00227673	protein_coding
ENSG00000163132	MSX1	-1.319965821	0.001725416	protein_coding
ENSG00000169733	RFNG	-1.319848975	0.001831818	protein_coding

ENSG00000103723	AP3B2	-1.316984346	0.004241204	protein_coding
ENSG00000204618	RNF39	-1.316218315	0.016632924	protein_coding
ENSG00000241360	PDXP	-1.316090887	0.001867228	protein_coding
ENSG00000127922	SEM1	-1.315498708	0.001736597	protein_coding
ENSG00000175287	PHYHD1	-1.313897363	0.002049816	protein_coding
ENSG00000051523	CYBA	-1.313707131	0.001734256	protein_coding
ENSG00000167397	VKORC1	-1.311639937	0.002763684	protein_coding
ENSG00000158552	ZFAND2B	-1.311467302	0.002144375	protein_coding
ENSG00000132432	SEC61G	-1.310834946	0.001798407	protein_coding
ENSG00000184897	H1FX	-1.310714249	0.001727495	protein_coding
ENSG00000184489	PTP4A3	-1.310460917	0.002187542	protein_coding
ENSG00000166136	NDUFB8	-1.310049138	0.002335168	protein_coding
ENSG00000167797	CDK2AP2	-1.309935656	0.002168281	protein_coding
ENSG00000169660	HEXDC	-1.309931195	0.002209902	protein_coding
ENSG00000159753	CARMIL2	-1.308428692	0.00249193	protein_coding
ENSG00000204308	RNF5	-1.308317693	0.001851853	protein_coding
ENSG00000131408	NR1H2	-1.307543519	0.002170773	protein_coding
ENSG00000142173	COL6A2	-1.307076411	0.001969687	protein_coding
ENSG00000167283	ATP5MG	-1.306823991	0.002141126	protein_coding
ENSG00000198832	SELENOM	-1.30678162	0.002061259	protein_coding
ENSG00000146707	POMZP3	-1.306421433	0.018991473	protein_coding
ENSG00000134590	RTL8C	-1.30638105	0.001878646	protein_coding
ENSG00000102109	PCSK1N	-1.30268501	0.002015555	protein_coding
ENSG00000188760	TMEM198	-1.30268071	0.002714644	protein_coding
ENSG00000161016	RPL8	-1.302060932	0.001790414	protein_coding
ENSG00000143977	SNRPG	-1.301877411	0.001905477	protein_coding
ENSG00000270170	NCBP2-AS2	-1.301387403	0.002443051	protein_coding
ENSG00000213654	GPSM3	-1.301012997	0.031520396	protein_coding
ENSG00000114315	HES1	-1.299491529	0.002099796	protein_coding
ENSG00000140682	TGFB1I1	-1.297339543	0.010823137	protein_coding
ENSG00000130332	LSM7	-1.296639748	0.00195681	protein_coding
ENSG00000133315	MACROD1	-1.294152362	0.002068481	protein_coding
ENSG00000166823	MESP1	-1.292712658	0.00445551	protein_coding
ENSG00000167565	SERTAD3	-1.292402627	0.00296135	protein_coding
ENSG00000178026	LRRC75B	-1.291643484	0.00355062	protein_coding
ENSG00000091592	NLRP1	-1.291592706	0.003496573	protein_coding
ENSG00000165507	DEPP1	-1.29072748	0.0074743	protein_coding
ENSG00000188338	SLC38A3	-1.290238759	0.003857405	protein_coding
ENSG00000115268	RPS15	-1.29009395	0.001972643	protein_coding
ENSG00000142089	IFITM3	-1.28993864	0.031103696	protein_coding
ENSG00000112699	GMDS	-1.289059514	0.002438384	protein_coding
ENSG00000241837	ATP5PO	-1.288667029	0.00225294	protein_coding
ENSG00000149257	SERPINH1	-1.287494928	0.002145153	protein_coding

ENSG00000172663	TMEM134	-1.287472938	0.002700642	protein_coding
ENSG00000233822	HIST1H2BN	-1.284860959	0.015253738	protein_coding
ENSG00000042753	AP2S1	-1.28423561	0.002140241	protein_coding
ENSG00000173511	VEGFB	-1.283570187	0.002518725	protein_coding
ENSG00000047662	FAM184B	-1.28328692	0.039238489	protein_coding
ENSG00000141744	PNMT	-1.28328692	0.039238489	protein_coding
ENSG00000180573	HIST1H2AC	-1.283114448	0.019044598	protein_coding
ENSG00000134107	BHLHE40	-1.282422315	0.002674002	protein_coding
ENSG00000163536	SERPINI1	-1.281593005	0.011863208	protein_coding
ENSG00000100092	SH3BP1	-1.281467979	0.00542632	protein_coding
ENSG00000204228	HSD17B8	-1.281073037	0.005068057	protein_coding
ENSG00000137070	IL11RA	-1.280189322	0.004134325	protein_coding
ENSG00000100982	PCIF1	-1.278678986	0.002372785	protein_coding
ENSG00000204387	C6orf48	-1.278436284	0.002440842	protein_coding
ENSG00000147813	NAPRT	-1.27831005	0.003742182	protein_coding
ENSG00000149489	ROM1	-1.277920426	0.021919674	protein_coding
ENSG00000105193	RPS16	-1.276759906	0.00219424	protein_coding
ENSG00000145945	FAM50B	-1.276572614	0.00352447	protein_coding
ENSG00000223802	CERS1	-1.275971635	0.00524849	protein_coding
ENSG00000166171	DPCD	-1.27559526	0.002783055	protein_coding
ENSG00000137720	C11orf1	-1.272816125	0.004488797	protein_coding
ENSG00000131116	ZNF428	-1.272765197	0.002489215	protein_coding
ENSG00000102125	TAZ	-1.271630814	0.002540044	protein_coding
ENSG00000232112	TMA7	-1.271276724	0.002665321	protein_coding
ENSG00000131652	THOC6	-1.270684321	0.002480298	protein_coding
ENSG00000039650	PNKP	-1.269586892	0.002606505	protein_coding
ENSG00000173918	C1QTNF1	-1.268823253	0.004610118	protein_coding
ENSG00000092621	PHGDH	-1.268779431	0.002335568	protein_coding
ENSG00000234965	SHISA8	-1.268772352	0.004176455	protein_coding
ENSG00000213347	MXD3	-1.266802769	0.00293064	protein_coding
ENSG00000171223	JUNB	-1.266727789	0.003248398	protein_coding
ENSG00000205542	TMSB4X	-1.265366186	0.003058144	protein_coding
ENSG00000100142	POLR2F	-1.265038051	0.002494622	protein_coding
ENSG00000134248	LAMTOR5	-1.264479237	0.002651022	protein_coding
ENSG00000099385	BCL7C	-1.263321794	0.002713189	protein_coding
ENSG00000118640	VAMP8	-1.262871297	0.004354816	protein_coding
ENSG00000161395	PGAP3	-1.262224487	0.003399879	protein_coding
ENSG00000185386	MAPK11	-1.260619741	0.004293054	protein_coding
ENSG00000170412	GPRC5C	-1.259253371	0.004459107	protein_coding
ENSG00000102904	TSNAXIP1	-1.259116054	0.019024949	protein_coding
ENSG00000174851	YIF1A	-1.257461652	0.002714502	protein_coding
ENSG00000167311	ART5	-1.256646262	0.007994347	protein_coding
ENSG00000142544	CTU1	-1.255265322	0.003162525	protein_coding

ENSG00000160799	CCDC12	-1.255063671	0.002988949	protein_coding
ENSG00000198816	ZNF358	-1.254860389	0.003121961	protein_coding
ENSG00000176101	SSNA1	-1.254851019	0.002720046	protein_coding
ENSG00000138085	ATRAID	-1.254175249	0.002863858	protein_coding
ENSG00000147889	CDKN2A	-1.253988315	0.002638051	protein_coding
ENSG00000106333	PCOLCE	-1.253814241	0.004510333	protein_coding
ENSG00000120896	SORBS3	-1.253344517	0.00307386	protein_coding
ENSG00000066379	ZNRD1	-1.253199904	0.003935473	protein_coding
ENSG00000005448	WDR54	-1.251938335	0.003142383	protein_coding
ENSG00000237489	C10orf143	-1.251854782	0.021631495	protein_coding
ENSG00000107872	FBXL15	-1.251762912	0.003513312	protein_coding
ENSG00000175573	C11orf68	-1.250567293	0.003322476	protein_coding
ENSG00000183828	NUDT14	-1.250330957	0.003638683	protein_coding
ENSG00000211450	SELENOH	-1.250321461	0.002858921	protein_coding
ENSG00000204710	SPDYC	-1.250048799	0.006576598	protein_coding
ENSG00000117691	NENF	-1.249279624	0.002895825	protein_coding
ENSG00000185988	PLK5	-1.24897185	0.007158799	protein_coding
ENSG00000149679	CABLES2	-1.248112768	0.003720281	protein_coding
ENSG00000105204	DYRK1B	-1.248112768	0.003720281	protein_coding
ENSG00000149932	TMEM219	-1.248062065	0.003065276	protein_coding
ENSG00000141933	TPGS1	-1.248000819	0.004038311	protein_coding
ENSG00000168894	RNF181	-1.246236393	0.003330122	protein_coding
ENSG00000198818	SFT2D1	-1.24615356	0.003150241	protein_coding
ENSG00000153391	INO80C	-1.245429601	0.003821322	protein_coding
ENSG00000170296	GABARAP	-1.244989964	0.011287195	protein_coding
ENSG00000141376	BCAS3	-1.244596205	0.003449403	protein_coding
ENSG00000213889	PPM1N	-1.244534985	0.00687511	protein_coding
ENSG00000127838	PNKD	-1.244357954	0.003116729	protein_coding
ENSG00000175193	PARL	-1.2432536	0.006974979	protein_coding
ENSG00000104979	C19orf53	-1.242058159	0.003023384	protein_coding
ENSG00000170801	HTRA3	-1.240996594	0.004550747	protein_coding
ENSG00000125734	GPR108	-1.240754657	0.003368489	protein_coding
ENSG00000154710	RABGEF1	-1.239933552	0.007582972	protein_coding
ENSG00000120129	DUSP1	-1.2393406	0.003144475	protein_coding
ENSG00000125835	SNRPB	-1.238130315	0.002973362	protein_coding
ENSG00000100276	RASL10A	-1.23765815	0.04754413	protein_coding
ENSG00000230510	PPP5D1	-1.237217571	0.035481962	protein_coding
ENSG00000236104	ZBTB22	-1.237194613	0.003723724	protein_coding
ENSG00000213563	C8orf82	-1.236891016	0.003329035	protein_coding
ENSG00000198417	MT1F	-1.236790034	0.021394294	protein_coding
ENSG00000092010	PSME1	-1.236597666	0.00360384	protein_coding
ENSG00000099958	DERL3	-1.23634354	0.009068762	protein_coding
ENSG00000167799	NUDT8	-1.235256848	0.005184026	protein_coding

ENSG00000142632	ARHGEF19	-1.233591952	0.012195035	protein_coding
ENSG00000142449	FBN3	-1.230630728	0.034842559	protein_coding
ENSG00000065029	ZNF76	-1.23050851	0.003525541	protein_coding
ENSG00000114395	CYB561D2	-1.230172001	0.004457902	protein_coding
ENSG00000188186	LAMTOR4	-1.229295451	0.003359519	protein_coding
ENSG00000090013	BLVRB	-1.228576039	0.003898147	protein_coding
ENSG00000197483	ZNF628	-1.228498381	0.004496722	protein_coding
ENSG00000126254	RBM42	-1.228202883	0.00328784	protein_coding
ENSG00000204498	NFKBIL1	-1.228171635	0.004112839	protein_coding
ENSG00000114626	ABTB1	-1.2279636	0.007643605	protein_coding
ENSG00000168010	ATG16L2	-1.227856887	0.005354677	protein_coding
ENSG00000127952	STYXL1	-1.2276305	0.004731238	protein_coding
ENSG00000067836	ROGDI	-1.226686592	0.005590184	protein_coding
ENSG00000122026	RPL21	-1.226554136	0.003243142	protein_coding
ENSG00000101439	CST3	-1.225821395	0.003413545	protein_coding
ENSG00000173207	CKS1B	-1.22545728	0.003395614	protein_coding
ENSG00000169231	THBS3	-1.224137479	0.007333432	protein_coding
ENSG00000148985	PGAP2	-1.223326941	0.004609656	protein_coding
ENSG00000160226	CFAP410	-1.222972827	0.004599822	protein_coding
ENSG00000132581	SDF2	-1.22257669	0.004692524	protein_coding
ENSG00000102977	ACD	-1.222559633	0.00399107	protein_coding
ENSG00000229809	ZNF688	-1.220536665	0.005796594	protein_coding
ENSG00000183648	NDUFB1	-1.22048097	0.0039924	protein_coding
ENSG00000213523	SRA1	-1.22048097	0.0039924	protein_coding
ENSG00000101986	ABCD1	-1.220188002	0.004525199	protein_coding
ENSG00000120068	HOXB8	-1.2196637	0.004342042	protein_coding
ENSG00000158825	CDA	-1.218058409	0.024220592	protein_coding
ENSG00000105617	LENG1	-1.217838385	0.006465983	protein_coding
ENSG00000162585	FAAP20	-1.217345086	0.004009569	protein_coding
ENSG00000002822	MAD1L1	-1.21663422	0.00396942	protein_coding
ENSG00000175550	DRAP1	-1.216111618	0.003683287	protein_coding
ENSG00000246705	H2AFJ	-1.215623196	0.00599004	protein_coding
ENSG00000164414	SLC35A1	-1.215596637	0.010052987	protein_coding
ENSG00000117308	GALE	-1.214298569	0.004495377	protein_coding
ENSG00000121900	TMEM54	-1.212964001	0.006907013	protein_coding
ENSG00000049089	COL9A2	-1.21142499	0.014307373	protein_coding
ENSG00000162076	FLYWCH2	-1.210864654	0.004327507	protein_coding
ENSG00000187601	MAGEH1	-1.210002631	0.011940437	protein_coding
ENSG00000277400	AC145212.1	-1.209541842	0.02851012	protein_coding
ENSG00000172382	PRSS27	-1.209513365	0.009143534	protein_coding
ENSG00000164306	PRIMPOL	-1.208090982	0.007150876	protein_coding
ENSG00000198723	TEX45	-1.208081448	0.042571852	protein_coding
ENSG00000125743	SNRPD2	-1.207458705	0.003778878	protein_coding

ENSG00000110717	NDUFS8	-1.207382932	0.00384271	protein_coding
ENSG00000167815	PRDX2	-1.205681981	0.003815916	protein_coding
ENSG00000110628	SLC22A18	-1.205333297	0.012603554	protein_coding
ENSG00000105669	COPE	-1.204742481	0.003979527	protein_coding
ENSG00000164587	RPS14	-1.2037434	0.003816678	protein_coding
ENSG00000104946	TBC1D17	-1.203501613	0.004929066	protein_coding
ENSG00000182324	KCNJ14	-1.202723462	0.012174901	protein_coding
ENSG00000066248	NGEF	-1.202542368	0.00642467	protein_coding
ENSG00000110697	PITPNM1	-1.200402038	0.004655614	protein_coding
ENSG00000173915	ATP5MD	-1.199500614	0.004194732	protein_coding
ENSG00000112514	CUTA	-1.199286492	0.00412699	protein_coding
ENSG00000099625	CBARP	-1.198988643	0.006050715	protein_coding
ENSG00000161996	WDR90	-1.198544821	0.004251308	protein_coding
ENSG00000172590	MRPL52	-1.198510129	0.004291818	protein_coding
ENSG00000123349	PFDN5	-1.19728004	0.004183408	protein_coding
ENSG00000129038	LOXL1	-1.197065454	0.007677452	protein_coding
ENSG00000248487	ABHD14A	-1.195816461	0.008817052	protein_coding
ENSG00000170291	ELP5	-1.194672181	0.004376164	protein_coding
ENSG00000165949	IFI27	-1.193327143	0.015683452	protein_coding
ENSG00000084234	APLP2	-1.193117285	0.004168628	protein_coding
ENSG00000132004	FBXW9	-1.191209976	0.005440864	protein_coding
ENSG00000064490	RFXANK	-1.190834435	0.0047132	protein_coding
ENSG00000105419	MEIS3	-1.190800833	0.005297412	protein_coding
ENSG00000178149	DALRD3	-1.19076876	0.005810382	protein_coding
ENSG00000106399	RPA3	-1.189119461	0.004795079	protein_coding
ENSG00000260456	C16orf95	-1.187344084	0.015760642	protein_coding
ENSG00000107281	NPDC1	-1.186175898	0.004916932	protein_coding
ENSG00000091640	SPAG7	-1.185450043	0.005033306	protein_coding
ENSG00000110011	DNAJC4	-1.184907807	0.006802961	protein_coding
ENSG00000090266	NDUFB2	-1.184804514	0.004653731	protein_coding
ENSG00000126709	IFI6	-1.184481367	0.010713731	protein_coding
ENSG00000103227	LMF1	-1.183464274	0.006819575	protein_coding
ENSG00000170860	LSM3	-1.183225621	0.00469844	protein_coding
ENSG00000173163	COMMD1	-1.18301165	0.008164549	protein_coding
ENSG00000179922	ZNF784	-1.182950789	0.006452018	protein_coding
ENSG00000232859	LYRM9	-1.182674389	0.026300005	protein_coding
ENSG00000115289	PCGF1	-1.180459018	0.006277645	protein_coding
ENSG00000186998	EMID1	-1.180419749	0.006541573	protein_coding
ENSG00000065717	TLE2	-1.179965149	0.006094247	protein_coding
ENSG00000125458	NT5C	-1.179826091	0.005499836	protein_coding
ENSG00000182195	LDOC1	-1.178897596	0.005568311	protein_coding
ENSG00000104897	SF3A2	-1.178646247	0.004784026	protein_coding
ENSG00000103254	FAM173A	-1.178448997	0.005464485	protein_coding

ENSG00000168890	TMEM150A	-1.17838779	0.008971824	protein_coding
ENSG00000241404	EGFL8	-1.177873117	0.025850844	protein_coding
ENSG00000135473	PAN2	-1.176422803	0.005291361	protein_coding
ENSG00000105516	DBP	-1.176354975	0.006414892	protein_coding
ENSG00000149131	SERPING1	-1.175396961	0.031655681	protein_coding
ENSG00000174428	GTF2IRD2B	-1.174495226	0.010081102	protein_coding
ENSG00000167107	ACSF2	-1.174488473	0.005909755	protein_coding
ENSG00000141504	SAT2	-1.172783418	0.005971072	protein_coding
ENSG00000182264	IZUMO1	-1.172632957	0.031215974	protein_coding
ENSG00000149273	RPS3	-1.171759405	0.004823711	protein_coding
ENSG00000178096	BOLA1	-1.171285344	0.006744312	protein_coding
ENSG00000053918	KCNQ1	-1.171032051	0.017130461	protein_coding
ENSG00000063854	HAGH	-1.170767108	0.005769143	protein_coding
ENSG00000128709	HOXD9	-1.170383739	0.006336435	protein_coding
ENSG00000104808	DHDH	-1.170060723	0.030798047	protein_coding
ENSG00000150779	TIMM8B	-1.169116989	0.00536684	protein_coding
ENSG00000188486	H2AFX	-1.168696941	0.005021877	protein_coding
ENSG00000178896	EXOSC4	-1.168539299	0.0057474	protein_coding
ENSG00000104835	SARS2	-1.168506097	0.010437524	protein_coding
ENSG00000077463	SIRT6	-1.168142564	0.005900545	protein_coding
ENSG00000135390	ATP5MC2	-1.167667017	0.005073893	protein_coding
ENSG00000204922	UQCC3	-1.167123012	0.006385895	protein_coding
ENSG00000170889	RPS9	-1.167062124	0.005001521	protein_coding
ENSG00000178623	GPR35	-1.167034997	0.048597149	protein_coding
ENSG00000004838	ZMYND10	-1.167034997	0.048597149	protein_coding
ENSG00000105373	NOP53	-1.166631325	0.005080624	protein_coding
ENSG00000123810	B9D2	-1.164974323	0.014980637	protein_coding
ENSG00000173846	PLK3	-1.164974323	0.014980637	protein_coding
ENSG00000102796	DHRS12	-1.164663695	0.022421406	protein_coding
ENSG00000158164	TMSB15A	-1.16394438	0.015000965	protein_coding
ENSG00000131471	AOC3	-1.163017731	0.015888096	protein_coding
ENSG00000122971	ACADS	-1.161266914	0.006517531	protein_coding
ENSG00000086506	HBQ1	-1.161239539	0.009377543	protein_coding
ENSG00000139625	MAP3K12	-1.161104939	0.00965497	protein_coding
ENSG00000241370	RPP21	-1.160682637	0.045931264	protein_coding
ENSG00000228672	PROB1	-1.160146591	0.008038774	protein_coding
ENSG00000268916	CSAG3	-1.159639782	0.015926495	protein_coding
ENSG00000267796	LIN37	-1.159480726	0.028988998	protein_coding
ENSG00000149806	FAU	-1.158925585	0.005356791	protein_coding
ENSG00000174871	CNIH2	-1.158917654	0.018649935	protein_coding
ENSG00000099624	ATP5F1D	-1.158596017	0.005475974	protein_coding
ENSG00000171984	SHLD1	-1.158591028	0.015937655	protein_coding
ENSG00000127184	COX7C	-1.158343194	0.005398425	protein_coding

ENSG00000182511	FES	-1.157928361	0.044743519	protein_coding
ENSG00000131143	COX4I1	-1.157442947	0.005428244	protein_coding
ENSG00000167549	CORO6	-1.15742793	0.007617721	protein_coding
ENSG00000164081	TEX264	-1.156752798	0.005704979	protein_coding
ENSG00000068001	HYAL2	-1.156701926	0.006114789	protein_coding
ENSG00000173928	SWSAP1	-1.155934966	0.012735494	protein_coding
ENSG00000167513	CDT1	-1.155580367	0.005546015	protein_coding
ENSG00000108518	PFN1	-1.155162844	0.005485595	protein_coding
ENSG00000187796	CARD9	-1.154732585	0.015975477	protein_coding
ENSG00000165644	COMTD1	-1.154502796	0.006085334	protein_coding
ENSG00000115350	POLE4	-1.154099625	0.006370479	protein_coding
ENSG00000105393	BABAM1	-1.15388562	0.006525479	protein_coding
ENSG00000214026	MRPL23	-1.153744762	0.006039387	protein_coding
ENSG00000095906	NUBP2	-1.152918258	0.005728528	protein_coding
ENSG00000152669	CCNO	-1.152370233	0.012115752	protein_coding
ENSG00000105655	ISYNA1	-1.151019114	0.005761411	protein_coding
ENSG00000159761	C16orf86	-1.150957335	0.036582079	protein_coding
ENSG00000169696	ASPSCR1	-1.150936647	0.006405576	protein_coding
ENSG00000157379	DHRS1	-1.150393635	0.008587569	protein_coding
ENSG00000163040	CCDC74A	-1.150367507	0.007528712	protein_coding
ENSG00000110076	NRXN2	-1.150299344	0.012357251	protein_coding
ENSG00000137106	GRHPR	-1.148520826	0.00594037	protein_coding
ENSG00000114923	SLC4A3	-1.14805049	0.011849192	protein_coding
ENSG00000163170	BOLA3	-1.147273884	0.006628342	protein_coding
ENSG00000099866	MADCAM1	-1.147152318	0.035303813	protein_coding
ENSG00000141574	SECTM1	-1.146428706	0.017086892	protein_coding
ENSG00000161618	ALDH16A1	-1.145922302	0.006690849	protein_coding
ENSG00000173992	CCS	-1.145878773	0.00724784	protein_coding
ENSG00000163703	CRELD1	-1.144207101	0.008876724	protein_coding
ENSG00000186468	RPS23	-1.143867343	0.005967196	protein_coding
ENSG00000167646	DNAAF3	-1.142337563	0.01086116	protein_coding
ENSG00000132016	C19orf57	-1.14203933	0.008906977	protein_coding
ENSG00000140691	ARMC5	-1.141768212	0.007687602	protein_coding
ENSG00000169035	KLK7	-1.140987817	0.033145797	protein_coding
ENSG00000133027	PEMT	-1.140248893	0.006998752	protein_coding
ENSG00000129158	SERGEF	-1.139468477	0.008138967	protein_coding
ENSG00000171119	NRTN	-1.13858975	0.009884901	protein_coding
ENSG00000197114	ZGPAT	-1.137189736	0.010953087	protein_coding
ENSG00000177556	ATOX1	-1.137005326	0.007192565	protein_coding
ENSG00000168701	TMEM208	-1.135890867	0.007161565	protein_coding
ENSG00000174460	ZCCHC12	-1.134909863	0.007716546	protein_coding
ENSG00000114388	NPRL2	-1.134615485	0.007554192	protein_coding
ENSG00000084764	MAPRE3	-1.134314938	0.009590027	protein_coding

ENSG00000101473	ACOT8	-1.133994316	0.007996007	protein_coding
ENSG00000106268	NUDT1	-1.133703376	0.007530418	protein_coding
ENSG00000142227	EMP3	-1.133123221	0.010323124	protein_coding
ENSG00000169567	HINT1	-1.13286702	0.006554662	protein_coding
ENSG00000167770	OTUB1	-1.132784928	0.006588752	protein_coding
ENSG00000167615	LENG8	-1.132582506	0.006561791	protein_coding
ENSG00000137404	NRM	-1.132413002	0.008154573	protein_coding
ENSG00000184076	UQCR10	-1.132007667	0.006748972	protein_coding
ENSG00000161992	PRR35	-1.131854106	0.018217997	protein_coding
ENSG00000105518	TMEM205	-1.131504172	0.010526643	protein_coding
ENSG00000117262	GPR89A	-1.13086606	0.009249524	protein_coding
ENSG00000127586	CHTF18	-1.130445125	0.006767134	protein_coding
ENSG00000158019	BABAM2	-1.130290452	0.007475502	protein_coding
ENSG00000092758	COL9A3	-1.130122671	0.010735251	protein_coding
ENSG00000131435	PDLIM4	-1.129437262	0.010491154	protein_coding
ENSG00000176428	VPS37D	-1.129392403	0.013935477	protein_coding
ENSG00000161328	LRRC56	-1.12928275	0.022310375	protein_coding
ENSG00000100968	NFATC4	-1.129220142	0.008108579	protein_coding
ENSG00000167281	RBFOX3	-1.128303874	0.009892358	protein_coding
ENSG00000105248	YJU2	-1.128131433	0.007683123	protein_coding
ENSG00000101457	DNTTIP1	-1.127969886	0.007302316	protein_coding
ENSG00000213465	ARL2	-1.127425462	0.007390414	protein_coding
ENSG00000130758	MAP3K10	-1.127158036	0.007616206	protein_coding
ENSG00000183963	SMTN	-1.126767919	0.008093463	protein_coding
ENSG00000177548	RABEP2	-1.126269669	0.008779467	protein_coding
ENSG00000054392	HHAT	-1.125554573	0.021709332	protein_coding
ENSG00000179526	SHARPIN	-1.12544649	0.007719282	protein_coding
ENSG00000168994	PXDC1	-1.125172382	0.011848749	protein_coding
ENSG00000130811	EIF3G	-1.124971848	0.006877047	protein_coding
ENSG00000167468	GPX4	-1.124935933	0.006837913	protein_coding
ENSG00000140474	ULK3	-1.124925753	0.007068285	protein_coding
ENSG00000243147	MRPL33	-1.124527545	0.007940942	protein_coding
ENSG00000150630	VEGFC	-1.124016563	0.042612006	protein_coding
ENSG00000129194	SOX15	-1.124009494	0.019893	protein_coding
ENSG00000065057	NTHL1	-1.123422262	0.00746635	protein_coding
ENSG00000142655	PEX14	-1.123171726	0.007991111	protein_coding
ENSG00000115657	ABCB6	-1.123008486	0.041652925	protein_coding
ENSG00000142534	RPS11	-1.122184457	0.006903326	protein_coding
ENSG00000223865	HLA-DPB1	-1.122076945	0.040756224	protein_coding
ENSG00000148362	PAXX	-1.121868817	0.007338622	protein_coding
ENSG00000125821	DTD1	-1.121778255	0.008474586	protein_coding
ENSG00000277791	PSMB3	-1.121598646	0.00712256	protein_coding
ENSG00000269335	IKBKG	-1.120890846	0.010358224	protein_coding

ENSG00000183479	TREX2	-1.11992893	0.019410249	protein_coding
ENSG00000114125	RNF7	-1.11986733	0.007926042	protein_coding
ENSG00000274419	TBC1D3D	-1.119663278	0.038387234	protein_coding
ENSG00000026036	RTEL1-TNFRSF6B	-1.118947388	0.019284915	protein_coding
ENSG00000176473	WDR25	-1.117437298	0.013149533	protein_coding
ENSG00000197696	NMB	-1.117242098	0.009126742	protein_coding
ENSG00000168061	SAC3D1	-1.117154855	0.007825219	protein_coding
ENSG00000129911	KLF16	-1.116821612	0.007619489	protein_coding
ENSG00000167705	RILP	-1.116666384	0.011280336	protein_coding
ENSG00000285589	AC010422.8	-1.116577576	0.035267165	protein_coding
ENSG00000185664	PMEL	-1.116577576	0.035267165	protein_coding
ENSG00000104870	FCGRT	-1.115632351	0.010210284	protein_coding
ENSG00000123576	ESX1	-1.115581027	0.034238954	protein_coding
ENSG00000169738	DCXR	-1.1150221	0.007587052	protein_coding
ENSG00000102265	TIMP1	-1.114376125	0.008178462	protein_coding
ENSG00000204305	AGER	-1.113884347	0.02963984	protein_coding
ENSG00000131469	RPL27	-1.112452313	0.007399045	protein_coding
ENSG00000167645	YIF1B	-1.111905966	0.007850191	protein_coding
ENSG00000090932	DLL3	-1.111888724	0.011639595	protein_coding
ENSG00000126756	UXT	-1.111628126	0.00849128	protein_coding
ENSG00000164978	NUDT2	-1.110900597	0.010163482	protein_coding
ENSG00000094631	HDAC6	-1.110814133	0.008338089	protein_coding
ENSG00000163794	UCN	-1.110127449	0.021362683	protein_coding
ENSG00000170965	PLAC1	-1.110089126	0.013298416	protein_coding
ENSG00000159713	TPPP3	-1.109912907	0.021281883	protein_coding
ENSG00000284976	BX255925.3	-1.109854522	0.00862165	protein_coding
ENSG00000100445	SDR39U1	-1.109215708	0.009366129	protein_coding
ENSG00000166295	ANAPC16	-1.108936033	0.008049079	protein_coding
ENSG00000105376	ICAM5	-1.108920019	0.008973516	protein_coding
ENSG00000104957	CCDC130	-1.108595084	0.009254004	protein_coding
ENSG00000128309	MPST	-1.108030588	0.007896497	protein_coding
ENSG00000168273	SMIM4	-1.10756992	0.010222191	protein_coding
ENSG00000125520	SLC2A4RG	-1.106981457	0.008122335	protein_coding
ENSG00000100983	GSS	-1.106630472	0.008358631	protein_coding
ENSG00000164162	ANAPC10	-1.105842238	0.016068231	protein_coding
ENSG00000166311	SMPD1	-1.105588968	0.01607251	protein_coding
ENSG00000162244	RPL29	-1.105135088	0.007769151	protein_coding
ENSG00000006757	PNPLA4	-1.105098791	0.010903269	protein_coding
ENSG00000104936	DMPK	-1.104452921	0.008618915	protein_coding
ENSG00000143198	MGST3	-1.104433333	0.008485057	protein_coding
ENSG00000141994	DUS3L	-1.104184561	0.008305781	protein_coding
ENSG00000123144	TRIR	-1.104179135	0.00798282	protein_coding

ENSG00000137038	DMAC1	-1.103899497	0.008618592	protein_coding
ENSG00000144589	STK11IP	-1.103316668	0.009446968	protein_coding
ENSG00000165502	RPL36AL	-1.103053532	0.00819123	protein_coding
ENSG00000213366	GSTM2	-1.101805948	0.01167373	protein_coding
ENSG00000187244	BCAM	-1.101716173	0.009413643	protein_coding
ENSG00000163481	RNF25	-1.101384755	0.009217019	protein_coding
ENSG00000065518	NDUFB4	-1.099501144	0.008503691	protein_coding
ENSG00000083845	RPS5	-1.099189057	0.008120481	protein_coding
ENSG00000130770	ATP5IF1	-1.099040756	0.00832818	protein_coding
ENSG00000102030	NAA10	-1.098376709	0.008661291	protein_coding
ENSG00000141741	MIEN1	-1.098221727	0.009624968	protein_coding
ENSG00000104976	SNAPC2	-1.097969069	0.011352458	protein_coding
ENSG00000133247	KMT5C	-1.097349894	0.008996676	protein_coding
ENSG00000167930	FAM234A	-1.096832856	0.009453588	protein_coding
ENSG00000266472	MRPS21	-1.096797067	0.00864517	protein_coding
ENSG00000104960	PTOV1	-1.096338274	0.008518131	protein_coding
ENSG00000163462	TRIM46	-1.096214161	0.02638362	protein_coding
ENSG00000132017	DCAF15	-1.095815878	0.008988734	protein_coding
ENSG00000157326	DHRS4	-1.095665651	0.010558285	protein_coding
ENSG00000204852	TCTN1	-1.095607027	0.010470579	protein_coding
ENSG00000145777	TSLP	-1.095553019	0.039100151	protein_coding
ENSG00000122481	RWDD3	-1.095271755	0.041363994	protein_coding
ENSG00000170043	TRAPPC1	-1.094776369	0.008923787	protein_coding
ENSG00000165655	ZNF503	-1.094686404	0.008549538	protein_coding
ENSG00000161920	MED11	-1.094298811	0.011851392	protein_coding
ENSG00000101608	MYL12A	-1.093693372	0.009458242	protein_coding
ENSG00000177700	POLR2L	-1.093015557	0.008927547	protein_coding
ENSG00000184209	SNRNP35	-1.092240014	0.011436999	protein_coding
ENSG00000140365	COMMD4	-1.091848607	0.008889148	protein_coding
ENSG00000164087	POC1A	-1.091241676	0.009926959	protein_coding
ENSG00000143774	GUK1	-1.091225898	0.008726643	protein_coding
ENSG00000100949	RABGGTA	-1.091008954	0.009872746	protein_coding
ENSG00000214253	FIS1	-1.090748477	0.009307678	protein_coding
ENSG00000139180	NDUFA9	-1.089672928	0.011157658	protein_coding
ENSG00000143320	CRABP2	-1.089433492	0.009278392	protein_coding
ENSG00000178821	TMEM52	-1.089333153	0.017663488	protein_coding
ENSG00000168393	DTYMK	-1.088840219	0.009045689	protein_coding
ENSG00000133874	RNF122	-1.088744807	0.01355047	protein_coding
ENSG00000117305	HMGCL	-1.088641965	0.0116265	protein_coding
ENSG00000102878	HSF4	-1.087924931	0.018855048	protein_coding
ENSG00000102032	RENBP	-1.087787726	0.018887039	protein_coding
ENSG00000220205	VAMP2	-1.087711367	0.010430575	protein_coding
ENSG00000185340	GAS2L1	-1.087551955	0.012296922	protein_coding

ENSG00000175334	BANF1	-1.08750544	0.008982535	protein_coding
ENSG00000140990	NDUFB10	-1.086711387	0.009031601	protein_coding
ENSG00000103024	NME3	-1.086665206	0.009290175	protein_coding
ENSG00000166173	LARP6	-1.085639285	0.0104451	protein_coding
ENSG00000143554	SLC27A3	-1.08535462	0.015490837	protein_coding
ENSG00000107902	LHPP	-1.08533502	0.013009806	protein_coding
ENSG00000128524	ATP6V1F	-1.08505298	0.009256894	protein_coding
ENSG00000169976	SF3B5	-1.084271185	0.00930586	protein_coding
ENSG00000174080	CTSF	-1.08421912	0.010950486	protein_coding
ENSG00000166228	PCBD1	-1.084196803	0.009952824	protein_coding
ENSG00000169750	RAC3	-1.08410829	0.00921217	protein_coding
ENSG00000130005	GAMT	-1.083237672	0.010001925	protein_coding
ENSG00000160446	ZDHHC12	-1.082301079	0.011114081	protein_coding
ENSG00000064961	HMG20B	-1.082243838	0.009352735	protein_coding
ENSG00000162591	MEGF6	-1.082107284	0.014591355	protein_coding
ENSG00000125826	RBCK1	-1.082006694	0.010031932	protein_coding
ENSG00000167676	PLIN4	-1.08200215	0.027282044	protein_coding
ENSG00000132522	GPS2	-1.081744624	0.01456437	protein_coding
ENSG00000005075	POLR2J	-1.081601129	0.010358903	protein_coding
ENSG00000137288	UQCC2	-1.081199084	0.010178092	protein_coding
ENSG00000196517	SLC6A9	-1.08097749	0.01055273	protein_coding
ENSG00000105701	FKBP8	-1.079349433	0.009421392	protein_coding
ENSG00000118292	C1orf54	-1.078373788	0.0350671	protein_coding
ENSG00000007516	BAIAP3	-1.07833488	0.019938131	protein_coding
ENSG00000243477	NAA80	-1.07802666	0.012969495	protein_coding
ENSG00000124172	ATP5F1E	-1.077955703	0.009713283	protein_coding
ENSG00000168477	TNXB	-1.077869452	0.035558915	protein_coding
ENSG00000170604	IRF2BP1	-1.077607426	0.009792541	protein_coding
ENSG00000237441	RGL2	-1.075726277	0.011976281	protein_coding
ENSG00000099800	TIMM13	-1.075293882	0.009820929	protein_coding
ENSG00000158109	TPRG1L	-1.074320064	0.012752296	protein_coding
ENSG00000177239	MAN1B1	-1.074110427	0.010210942	protein_coding
ENSG00000196155	PLEKHG4	-1.07400321	0.011244704	protein_coding
ENSG00000183978	COA3	-1.073554297	0.010584869	protein_coding
ENSG00000176209	SMIM19	-1.07351514	0.013425731	protein_coding
ENSG00000140988	RPS2	-1.073303889	0.009663325	protein_coding
ENSG00000095066	HOOK2	-1.073117797	0.011511598	protein_coding
ENSG00000109736	MFSD10	-1.073008891	0.010298409	protein_coding
ENSG00000100151	PICK1	-1.072415411	0.010600033	protein_coding
ENSG00000156411	ATP5MPL	-1.071629131	0.010197767	protein_coding
ENSG00000119655	NPC2	-1.071228445	0.011303355	protein_coding
ENSG00000067829	IDH3G	-1.071104848	0.010285007	protein_coding
ENSG00000100908	EMC9	-1.071096573	0.011792891	protein_coding

ENSG00000239789	MRPS17	-1.070725443	0.01434397	protein_coding
ENSG00000172301	COPRS	-1.070673827	0.010760197	protein_coding
ENSG00000163159	VPS72	-1.070601123	0.010381593	protein_coding
ENSG00000147684	NDUFB9	-1.0705757	0.010226634	protein_coding
ENSG00000142552	RCN3	-1.069147086	0.028700142	protein_coding
ENSG00000076201	PTPN23	-1.069062894	0.010677387	protein_coding
ENSG00000172336	POP7	-1.068940291	0.010299826	protein_coding
ENSG00000140983	RHOT2	-1.068913927	0.010196624	protein_coding
ENSG00000244165	P2RY11	-1.068302483	0.012985353	protein_coding
ENSG00000085552	IGSF9	-1.067180269	0.021309186	protein_coding
ENSG00000116691	MIIP	-1.066756934	0.01168199	protein_coding
ENSG00000102890	ELMO3	-1.066733372	0.029757221	protein_coding
ENSG00000147383	NSDHL	-1.066577147	0.010677309	protein_coding
ENSG00000099282	TSPAN15	-1.066049814	0.012602534	protein_coding
ENSG00000171530	TBCA	-1.065853071	0.010477521	protein_coding
ENSG00000243927	MRPS6	-1.065203842	0.012529871	protein_coding
ENSG00000151689	INPP1	-1.06507576	0.016064668	protein_coding
ENSG00000159958	TNFRSF13C	-1.064748503	0.014840566	protein_coding
ENSG00000205572	SERF1B	-1.064585232	0.020389329	protein_coding
ENSG00000146540	C7orf50	-1.06368193	0.01086731	protein_coding
ENSG00000119013	NDUFB3	-1.063267315	0.011389978	protein_coding
ENSG00000169857	AVEN	-1.062027109	0.015177624	protein_coding
ENSG00000063241	ISOC2	-1.061228678	0.011131806	protein_coding
ENSG00000133112	TPT1	-1.06030106	0.010644259	protein_coding
ENSG00000177854	TMEM187	-1.058827856	0.016535348	protein_coding
ENSG00000198840	MT-ND3	-1.058778024	0.01071665	protein_coding
ENSG00000156587	UBE2L6	-1.058509838	0.039576581	protein_coding
ENSG00000132793	LPIN3	-1.058074258	0.013942289	protein_coding
ENSG00000143578	CREB3L4	-1.058067031	0.018807095	protein_coding
ENSG00000168758	SEMA4C	-1.058014208	0.012011654	protein_coding
ENSG00000184986	TMEM121	-1.057276194	0.013785389	protein_coding
ENSG00000180879	SSR4	-1.056651186	0.011055751	protein_coding
ENSG00000127445	PIN1	-1.056353668	0.011222214	protein_coding
ENSG00000185000	DGAT1	-1.056290697	0.012158542	protein_coding
ENSG00000142541	RPL13A	-1.056144244	0.010869269	protein_coding
ENSG00000100075	SLC25A1	-1.055300815	0.011182119	protein_coding
ENSG00000070540	WIPI1	-1.055141574	0.046016399	protein_coding
ENSG00000156482	RPL30	-1.05510699	0.01105522	protein_coding
ENSG00000153443	UBALD1	-1.053891202	0.01176088	protein_coding
ENSG00000167543	TP53I13	-1.053835169	0.012372492	protein_coding
ENSG00000180096	1-Sep	-1.053505944	0.031516168	protein_coding
ENSG00000239672	NME1	-1.052661813	0.011718273	protein_coding
ENSG00000262814	MRPL12	-1.052659287	0.012394482	protein_coding

ENSG00000160957	RECQL4	-1.052618187	0.011475462	protein_coding
ENSG00000182362	YBEY	-1.052200474	0.015315515	protein_coding
ENSG00000204237	OXLD1	-1.051800875	0.012711441	protein_coding
ENSG00000115685	PPP1R7	-1.05108065	0.012137911	protein_coding
ENSG00000169189	NSMCE1	-1.050851198	0.013424827	protein_coding
ENSG00000142546	NOSIP	-1.049284195	0.012088209	protein_coding
ENSG00000100387	RBX1	-1.04843142	0.01196522	protein_coding
ENSG00000242802	AP5Z1	-1.048315863	0.01293722	protein_coding
ENSG00000104881	PPP1R13L	-1.048012406	0.015639156	protein_coding
ENSG00000160813	PPP1R35	-1.047863299	0.014186512	protein_coding
ENSG00000197106	SLC6A17	-1.047585924	0.023760611	protein_coding
ENSG00000037042	TUBG2	-1.047178636	0.017604501	protein_coding
ENSG00000140025	EFCAB11	-1.047151541	0.019875295	protein_coding
ENSG00000137312	FLOT1	-1.045242122	0.012218368	protein_coding
ENSG00000103249	CLCN7	-1.045039589	0.012349942	protein_coding
ENSG00000143258	USP21	-1.044990622	0.012766812	protein_coding
ENSG00000101940	WDR13	-1.044916643	0.012522225	protein_coding
ENSG00000077312	SNRPA	-1.044776835	0.012109456	protein_coding
ENSG00000204348	DXO	-1.044722249	0.013294431	protein_coding
ENSG00000178980	SELENOW	-1.044292361	0.013493988	protein_coding
ENSG00000100348	TXN2	-1.044234166	0.012245448	protein_coding
ENSG00000104522	TSTA3	-1.043323761	0.012922748	protein_coding
ENSG00000170421	KRT8	-1.04331889	0.014740123	protein_coding
ENSG00000061656	SPAG4	-1.043284644	0.030316257	protein_coding
ENSG00000161904	LEMD2	-1.042890857	0.012840203	protein_coding
ENSG00000114391	RPL24	-1.042842465	0.011924617	protein_coding
ENSG00000179943	FIZ1	-1.041369409	0.014982094	protein_coding
ENSG00000167925	GHDC	-1.041098576	0.013734915	protein_coding
ENSG00000106330	MOSPD3	-1.041093234	0.016394291	protein_coding
ENSG00000170892	TSEN34	-1.040367924	0.012765565	protein_coding
ENSG00000213983	AP1G2	-1.040171884	0.014457863	protein_coding
ENSG00000171148	TADA3	-1.04005121	0.012914392	protein_coding
ENSG00000206503	HLA-A	-1.038970592	0.013220877	protein_coding
ENSG00000186577	SMIM29	-1.03880907	0.020540817	protein_coding
ENSG00000114767	RRP9	-1.038482361	0.013049901	protein_coding
ENSG00000131067	GGT7	-1.03842314	0.013288363	protein_coding
ENSG00000143224	PPOX	-1.037956898	0.016279163	protein_coding
ENSG00000182208	MOB2	-1.036406304	0.014399097	protein_coding
ENSG00000170577	SIX2	-1.035509069	0.013519306	protein_coding
ENSG00000214078	CPNE1	-1.035480766	0.01316086	protein_coding
ENSG00000132702	HAPLN2	-1.035079879	0.035255976	protein_coding
ENSG00000170122	FOXD4	-1.034840021	0.028479922	protein_coding
ENSG00000107223	EDF1	-1.034366174	0.012902808	protein_coding

ENSG00000162496	DHRS3	-1.033845091	0.021183107	protein_coding
ENSG00000106628	POLD2	-1.033842723	0.012945535	protein_coding
ENSG00000067221	STOML1	-1.032573954	0.01925397	protein_coding
ENSG00000187634	SAMD11	-1.032340365	0.013387146	protein_coding
ENSG00000198680	TUSC1	-1.03205496	0.014026727	protein_coding
ENSG00000130731	METTL26	-1.031452633	0.013568034	protein_coding
ENSG00000088543	C3orf18	-1.03108894	0.02362233	protein_coding
ENSG00000152475	ZNF837	-1.03108894	0.02362233	protein_coding
ENSG00000071082	RPL31	-1.030662326	0.012936553	protein_coding
ENSG00000126456	IRF3	-1.030373808	0.014077826	protein_coding
ENSG00000172270	BSG	-1.029952504	0.012997336	protein_coding
ENSG00000175600	SUGCT	-1.029682403	0.02484791	protein_coding
ENSG00000103266	STUB1	-1.028347387	0.0135205	protein_coding
ENSG00000108826	MRPL27	-1.028340749	0.014285054	protein_coding
ENSG00000149557	FEZ1	-1.028254143	0.026421638	protein_coding
ENSG00000109475	RPL34	-1.027900391	0.013228864	protein_coding
ENSG00000185043	CIB1	-1.02703344	0.014223223	protein_coding
ENSG00000105559	PLEKHA4	-1.02694883	0.026504484	protein_coding
ENSG00000172500	FIBP	-1.026458854	0.014311745	protein_coding
ENSG00000106367	AP1S1	-1.026084098	0.014062385	protein_coding
ENSG00000136280	CCM2	-1.025898452	0.016129217	protein_coding
ENSG00000158747	NBL1	-1.025762516	0.016375215	protein_coding
ENSG00000164896	FASTK	-1.025433118	0.013778877	protein_coding
ENSG00000171960	PPIH	-1.024712551	0.015191472	protein_coding
ENSG00000149925	ALDOA	-1.024342789	0.02032646	protein_coding
ENSG00000162384	C1orf123	-1.022631248	0.015195586	protein_coding
ENSG00000064687	ABCA7	-1.022488924	0.01546097	protein_coding
ENSG00000062282	DGAT2	-1.021564445	0.017907462	protein_coding
ENSG00000112561	TFEB	-1.021142486	0.026066943	protein_coding
ENSG00000187609	EXD3	-1.021110368	0.019437551	protein_coding
ENSG00000214160	ALG3	-1.020946164	0.014919252	protein_coding
ENSG00000196465	MYL6B	-1.020943617	0.014661989	protein_coding
ENSG00000186501	TMEM222	-1.020720328	0.015473967	protein_coding
ENSG00000215375	MYL5	-1.020358375	0.020930363	protein_coding
ENSG00000250571	GLI4	-1.020292346	0.016664765	protein_coding
ENSG00000149591	TAGLN	-1.018809693	0.034196404	protein_coding
ENSG00000071889	FAM3A	-1.017714342	0.014802867	protein_coding
ENSG00000166292	TMEM100	-1.017606298	0.038729376	protein_coding
ENSG00000214279	SCART1	-1.017340096	0.023133282	protein_coding
ENSG00000126461	SCAF1	-1.017117681	0.014481572	protein_coding
ENSG00000143256	PFDN2	-1.016418065	0.014910745	protein_coding
ENSG00000065268	WDR18	-1.016300257	0.014699953	protein_coding
ENSG00000072506	HSD17B10	-1.016040337	0.014597843	protein_coding

ENSG00000147403	RPL10	-1.015935125	0.014223229	protein_coding
ENSG00000180992	MRPL14	-1.014229398	0.015133343	protein_coding
ENSG00000123975	CKS2	-1.01411795	0.01498988	protein_coding
ENSG00000102309	PIN4	-1.013933492	0.015963674	protein_coding
ENSG00000177943	MAMDC4	-1.013007413	0.027246764	protein_coding
ENSG00000129235	TXNDC17	-1.013000346	0.01643557	protein_coding
ENSG00000105135	ILVBL	-1.012758524	0.015292416	protein_coding
ENSG00000247596	TWF2	-1.012393459	0.016103422	protein_coding
ENSG00000172889	EGFL7	-1.011884652	0.016194018	protein_coding
ENSG00000204227	RING1	-1.011273079	0.015829813	protein_coding
ENSG00000104805	NUCB1	-1.009985318	0.015233636	protein_coding
ENSG00000181404	WASHC1	-1.009919442	0.019861739	protein_coding
ENSG00000167641	PPP1R14A	-1.009454602	0.019433496	protein_coding
ENSG00000184752	NDUFA12	-1.009301106	0.015991987	protein_coding
ENSG00000158941	CCAR2	-1.008180124	0.015679182	protein_coding
ENSG00000082014	SMARCD3	-1.00796609	0.015930608	protein_coding
ENSG00000174177	CTU2	-1.007533476	0.016663152	protein_coding
ENSG00000173660	UQCRH	-1.007300475	0.015261206	protein_coding
ENSG00000090263	MRPS33	-1.006580057	0.016357682	protein_coding
ENSG00000105997	HOXA3	-1.005370013	0.019854368	protein_coding
ENSG00000112306	RPS12	-1.004947798	0.015350404	protein_coding
ENSG00000166681	BEX3	-1.004491383	0.015518922	protein_coding
ENSG00000125931	CITED1	-1.004018087	0.019083153	protein_coding
ENSG00000167632	TRAPPC9	-1.003746525	0.020166624	protein_coding
ENSG00000030582	GRN	-1.002138235	0.015956573	protein_coding
ENSG00000032444	PNPLA6	-1.002009171	0.017095958	protein_coding
ENSG00000174775	HRAS	-1.001613108	0.016517577	protein_coding
ENSG00000189227	C15orf61	-1.001503179	0.020446683	protein_coding
ENSG00000162385	MAGOH	-1.001437776	0.016679157	protein_coding
ENSG00000136908	DPM2	-1.001397621	0.016549416	protein_coding
ENSG00000174021	GNG5	-1.000720498	0.021307459	protein_coding
ENSG00000076641	PAG1	1.000009382	0.017656512	protein_coding
ENSG00000019995	ZRANB1	1.000065837	0.021158063	protein_coding
ENSG00000180357	ZNF609	1.000201325	0.017302611	protein_coding
ENSG00000057704	TMCC3	1.000238013	0.026514585	protein_coding
ENSG00000170832	USP32	1.000294312	0.017863895	protein_coding
ENSG00000107105	ELAVL2	1.001107802	0.020516696	protein_coding
ENSG00000162769	FLVCR1	1.002229515	0.017629413	protein_coding
ENSG00000111912	NCOA7	1.002418542	0.020583307	protein_coding
ENSG00000120690	ELF1	1.003107225	0.019115182	protein_coding
ENSG00000121892	PDS5A	1.003251622	0.016109874	protein_coding
ENSG00000178694	NSUN3	1.003370048	0.024321	protein_coding
ENSG00000141425	RPRD1A	1.003515119	0.015726872	protein_coding

ENSG00000110422	HIPK3	1.003772117	0.017970486	protein_coding
ENSG00000065150	IPO5	1.003946681	0.015519103	protein_coding
ENSG00000146376	ARHGAP18	1.004715583	0.02398421	protein_coding
ENSG00000126016	AMOT	1.005641374	0.015317789	protein_coding
ENSG00000090060	PAPOLA	1.006223149	0.015478516	protein_coding
ENSG00000028203	VEZT	1.006573313	0.016933104	protein_coding
ENSG00000068650	ATP11A	1.006737394	0.016828552	protein_coding
ENSG00000059758	CDK17	1.007782733	0.018545767	protein_coding
ENSG00000152223	EPG5	1.008181839	0.023142029	protein_coding
ENSG00000005483	KMT2E	1.00847907	0.017086501	protein_coding
ENSG00000113569	NUP155	1.008640888	0.015433038	protein_coding
ENSG00000205269	TMEM170B	1.008813578	0.017743419	protein_coding
ENSG00000102230	PCYT1B	1.008852095	0.046668662	protein_coding
ENSG00000155158	TTC39B	1.009475867	0.02376846	protein_coding
ENSG00000151338	MIPOL1	1.01016201	0.021426035	protein_coding
ENSG00000144747	TMF1	1.010206897	0.017400344	protein_coding
ENSG00000150471	ADGRL3	1.010235815	0.019212639	protein_coding
ENSG00000183495	EP400	1.010571042	0.015341925	protein_coding
ENSG00000105887	MTPN	1.011364335	0.014908158	protein_coding
ENSG00000144791	LIMD1	1.012004908	0.015943165	protein_coding
ENSG00000152413	HOMER1	1.012387339	0.016297217	protein_coding
ENSG00000088854	C20orf194	1.01326061	0.021520398	protein_coding
ENSG00000162733	DDR2	1.013416155	0.018824934	protein_coding
ENSG00000254598	CSNK2A3	1.01403971	0.030157284	protein_coding
ENSG00000254004	ZNF260	1.014116496	0.020150659	protein_coding
ENSG00000250305	TRMT9B	1.015379901	0.036217426	protein_coding
ENSG00000093000	NUP50	1.01576698	0.01473054	protein_coding
ENSG00000107036	RIC1	1.016217949	0.016626757	protein_coding
ENSG00000198879	SFMBT2	1.016428558	0.016963761	protein_coding
ENSG00000211455	STK38L	1.016549171	0.016213113	protein_coding
ENSG00000138386	NAB1	1.017070512	0.016675609	protein_coding
ENSG00000118965	WDR35	1.017475042	0.015731661	protein_coding
ENSG00000196482	ESRRG	1.017494032	0.039567381	protein_coding
ENSG00000102781	KATNAL1	1.017968198	0.017666696	protein_coding
ENSG00000168116	KIAA1586	1.018095987	0.018261392	protein_coding
ENSG00000109572	CLCN3	1.018317528	0.015024794	protein_coding
ENSG00000007202	KIAA0100	1.018453274	0.01444396	protein_coding
ENSG00000134324	LPIN1	1.01847084	0.016822049	protein_coding
ENSG00000050405	LIMA1	1.018908842	0.015314111	protein_coding
ENSG00000180574	EIF2S3B	1.019094708	0.030870438	protein_coding
ENSG00000165671	NSD1	1.019182659	0.014290304	protein_coding
ENSG00000039068	CDH1	1.019309861	0.032524508	protein_coding
ENSG00000164327	RICTOR	1.019474526	0.017837558	protein_coding

ENSG00000114346	ECT2	1.019848643	0.014679238	protein_coding
ENSG00000182150	ERCC6L2	1.02001298	0.019104317	protein_coding
ENSG00000100167	3-Sep	1.020925852	0.015463132	protein_coding
ENSG00000162852	CNST	1.021491582	0.018922978	protein_coding
ENSG00000219481	NBPF1	1.021698628	0.017154593	protein_coding
ENSG00000147894	C9orf72	1.022105075	0.018182159	protein_coding
ENSG00000154124	OTULIN	1.022320295	0.015578709	protein_coding
ENSG00000214357	NEURL1B	1.022342562	0.023408739	protein_coding
ENSG00000108424	KPNB1	1.022405229	0.013718863	protein_coding
ENSG00000163513	TGFBR2	1.022655323	0.023862253	protein_coding
ENSG00000145990	GFOD1	1.022664651	0.015904068	protein_coding
ENSG00000101972	STAG2	1.023045739	0.013801063	protein_coding
ENSG00000182957	SPATA13	1.023763255	0.02122763	protein_coding
ENSG00000240694	PNMA2	1.023995029	0.017578132	protein_coding
ENSG00000150712	MTMR12	1.024065585	0.015555999	protein_coding
ENSG00000156535	CD109	1.024133487	0.018992632	protein_coding
ENSG00000160179	ABCG1	1.024381671	0.046991593	protein_coding
ENSG00000152061	RABGAP1L	1.024469797	0.016105017	protein_coding
ENSG00000117461	PIK3R3	1.025088586	0.015261292	protein_coding
ENSG00000108175	ZMIZ1	1.025117809	0.014530295	protein_coding
ENSG00000178074	C2orf69	1.025268215	0.016691227	protein_coding
ENSG00000095203	EPB41L4B	1.025592586	0.017984942	protein_coding
ENSG00000137822	TUBGCP4	1.027377268	0.015024813	protein_coding
ENSG00000172466	ZNF24	1.027677312	0.013447718	protein_coding
ENSG00000141027	NCOR1	1.027789111	0.013767721	protein_coding
ENSG00000177463	NR2C2	1.02792596	0.014238728	protein_coding
ENSG00000163638	ADAMTS9	1.027950829	0.022871413	protein_coding
ENSG00000137177	KIF13A	1.028233491	0.015543268	protein_coding
ENSG00000170871	KIAA0232	1.029248438	0.01521127	protein_coding
ENSG00000112319	EYA4	1.029464987	0.014853251	protein_coding
ENSG00000133056	PIK3C2B	1.02951904	0.015333097	protein_coding
ENSG00000125772	GPCPD1	1.029870049	0.01692027	protein_coding
ENSG00000123191	ATP7B	1.029983929	0.014793903	protein_coding
ENSG00000159256	MORC3	1.030014129	0.017665352	protein_coding
ENSG00000065526	SPEN	1.030413017	0.013099777	protein_coding
ENSG00000116747	TROVE2	1.030460237	0.01406998	protein_coding
ENSG00000170145	SIK2	1.030734799	0.014391392	protein_coding
ENSG00000012174	MBTPS2	1.032238988	0.014438001	protein_coding
ENSG00000173575	CHD2	1.032457522	0.014064451	protein_coding
ENSG00000111371	SLC38A1	1.032489084	0.013025023	protein_coding
ENSG00000224470	ATXN1L	1.032497499	0.014025365	protein_coding
ENSG00000127946	HIP1	1.032843001	0.014269818	protein_coding
ENSG00000082516	GEMIN5	1.033267358	0.013549305	protein_coding

ENSG00000173064	HECTD4	1.034399646	0.013579237	protein_coding
ENSG00000157657	ZNF618	1.034522443	0.013861378	protein_coding
ENSG00000167981	ZNF597	1.034714032	0.034958896	protein_coding
ENSG00000123094	RASSF8	1.034853831	0.015483637	protein_coding
ENSG00000198887	SMC5	1.035140517	0.013763348	protein_coding
ENSG00000142556	ZNF614	1.035397709	0.016675525	protein_coding
ENSG00000125630	POLR1B	1.035670084	0.013217909	protein_coding
ENSG00000134294	SLC38A2	1.035970791	0.01277468	protein_coding
ENSG00000109586	GALNT7	1.036639639	0.014804977	protein_coding
ENSG00000183943	PRKX	1.036724294	0.013998079	protein_coding
ENSG00000082438	COBLL1	1.037574904	0.017926768	protein_coding
ENSG00000131370	SH3BP5	1.037816885	0.016783658	protein_coding
ENSG00000120256	LRP11	1.03998087	0.038182333	protein_coding
ENSG00000139970	RTN1	1.040101689	0.038333506	protein_coding
ENSG00000180884	ZNF792	1.040101689	0.038333506	protein_coding
ENSG00000179841	AKAP5	1.040710827	0.021150635	protein_coding
ENSG00000070367	EXOC5	1.041399309	0.013024013	protein_coding
ENSG00000196369	SRGAP2B	1.042009454	0.021200137	protein_coding
ENSG00000163535	SGO2	1.042011817	0.014482625	protein_coding
ENSG00000107863	ARHGAP21	1.042084154	0.012897716	protein_coding
ENSG00000137942	FNBP1L	1.042382327	0.013708761	protein_coding
ENSG00000167258	CDK12	1.043108073	0.012381582	protein_coding
ENSG00000068878	PSME4	1.044293779	0.012412028	protein_coding
ENSG00000104635	SLC39A14	1.04480898	0.0121851	protein_coding
ENSG00000040933	INPP4A	1.044876625	0.012932479	protein_coding
ENSG00000196586	MYO6	1.047096632	0.012744742	protein_coding
ENSG00000153956	CACNA2D1	1.047146287	0.01445366	protein_coding
ENSG00000139668	WDFY2	1.048697305	0.016485881	protein_coding
ENSG00000186951	PPARA	1.048787415	0.014831719	protein_coding
ENSG00000029725	RABEP1	1.048880122	0.01201361	protein_coding
ENSG00000154639	CXADR	1.048985238	0.013556772	protein_coding
ENSG00000139618	BRCA2	1.049188689	0.013348187	protein_coding
ENSG00000123600	METTL8	1.049567733	0.013863345	protein_coding
ENSG00000101695	RNF125	1.050459495	0.013572857	protein_coding
ENSG00000173281	PPP1R3B	1.051190362	0.020463786	protein_coding
ENSG00000198836	OPA1	1.05131747	0.012193007	protein_coding
ENSG00000078747	ITCH	1.051380507	0.012329594	protein_coding
ENSG00000015133	CCDC88C	1.051523363	0.01375655	protein_coding
ENSG00000125885	MCM8	1.051741511	0.012097322	protein_coding
ENSG00000131791	PRKAB2	1.052172551	0.012666097	protein_coding
ENSG00000083642	PDS5B	1.0523078	0.01252313	protein_coding
ENSG00000154309	DISP1	1.052421629	0.022573217	protein_coding
ENSG00000166147	FBN1	1.052612395	0.015636978	protein_coding

ENSG00000181104	F2R	1.053290222	0.013152333	protein_coding
ENSG00000165732	DDX21	1.053802642	0.011149429	protein_coding
ENSG00000125676	THOC2	1.054123814	0.011327741	protein_coding
ENSG00000076356	PLXNA2	1.054259037	0.015634731	protein_coding
ENSG00000114062	UBE3A	1.055478603	0.012162303	protein_coding
ENSG00000062650	WAPL	1.055954293	0.011554441	protein_coding
ENSG00000101639	CEP192	1.056605928	0.01358116	protein_coding
ENSG00000134369	NAV1	1.05686542	0.012339222	protein_coding
ENSG00000167065	DUSP18	1.057333905	0.030043599	protein_coding
ENSG00000131263	RLIM	1.058434199	0.011160968	protein_coding
ENSG00000150938	CRIM1	1.059446698	0.012506716	protein_coding
ENSG00000138592	USP8	1.059484638	0.012503211	protein_coding
ENSG00000162702	ZNF281	1.059624762	0.011790922	protein_coding
ENSG00000147050	KDM6A	1.0599423	0.011906489	protein_coding
ENSG00000151151	IPMK	1.060709418	0.014206664	protein_coding
ENSG00000116106	EPHA4	1.060988326	0.01294825	protein_coding
ENSG00000162688	AGL	1.061403133	0.011448473	protein_coding
ENSG00000001629	ANKIB1	1.061467302	0.012762665	protein_coding
ENSG00000196268	ZNF493	1.061745591	0.024992401	protein_coding
ENSG00000139926	FRMD6	1.061826128	0.012902258	protein_coding
ENSG00000167191	GPRC5B	1.062407187	0.013842964	protein_coding
ENSG00000173218	VANGL1	1.062521103	0.012031739	protein_coding
ENSG00000100393	EP300	1.063036933	0.010815328	protein_coding
ENSG00000141429	GALNT1	1.063092325	0.010802301	protein_coding
ENSG00000225830	ERCC6	1.064946261	0.015219495	protein_coding
ENSG00000151320	AKAP6	1.065325099	0.036686451	protein_coding
ENSG00000185352	HS6ST3	1.066325493	0.03089312	protein_coding
ENSG00000048471	SNX29	1.066407245	0.014208595	protein_coding
ENSG00000154642	C21orf91	1.066518952	0.013909178	protein_coding
ENSG00000159579	RSPRY1	1.06700502	0.012192885	protein_coding
ENSG00000151233	GXYLT1	1.067080068	0.011352074	protein_coding
ENSG00000172071	EIF2AK3	1.067337847	0.015578498	protein_coding
ENSG00000086712	TXLNG	1.06772276	0.01061385	protein_coding
ENSG00000143190	POU2F1	1.068159503	0.0113022	protein_coding
ENSG00000196935	SRGAP1	1.068752284	0.018303645	protein_coding
ENSG00000092439	TRPM7	1.069345334	0.011116046	protein_coding
ENSG00000171943	SRGAP2C	1.069456963	0.013265315	protein_coding
ENSG00000140575	IQGAP1	1.069747019	0.010267916	protein_coding
ENSG00000159433	STARD9	1.069995939	0.012463491	protein_coding
ENSG00000138759	FRAS1	1.071197379	0.011655897	protein_coding
ENSG00000185532	PRKG1	1.071952566	0.015119084	protein_coding
ENSG00000205060	SLC35B4	1.07226359	0.010231393	protein_coding
ENSG00000196376	SLC35F1	1.072803587	0.012158262	protein_coding

ENSG00000145087	STXBP5L	1.072920237	0.029208486	protein_coding
ENSG00000132326	PER2	1.073396602	0.016070052	protein_coding
ENSG00000168743	NPNT	1.073672108	0.039811039	protein_coding
ENSG00000108061	SHOC2	1.073710275	0.010871977	protein_coding
ENSG00000082269	FAM135A	1.073767273	0.011996713	protein_coding
ENSG00000168282	MGAT2	1.073998569	0.048419861	protein_coding
ENSG00000120063	GNA13	1.074981015	0.010615099	protein_coding
ENSG00000149289	ZC3H12C	1.075158127	0.017366448	protein_coding
ENSG00000185722	ANKFY1	1.075339957	0.010156588	protein_coding
ENSG00000070882	OSBPL3	1.075747154	0.011333251	protein_coding
ENSG00000133026	MYH10	1.075873697	0.009683531	protein_coding
ENSG00000118985	ELL2	1.075908087	0.011956481	protein_coding
ENSG00000116678	LEPR	1.076518543	0.011551466	protein_coding
mk	IGF2BP3	1.076596495	0.011227663	protein_coding
ENSG00000184661	CDCA2	1.077228158	0.011797658	protein_coding
ENSG00000175216	CKAP5	1.077293355	0.009607117	protein_coding
ENSG00000182010	RTKN2	1.077565548	0.01217923	protein_coding
ENSG00000139517	LNX2	1.077585734	0.012492165	protein_coding
ENSG00000125285	SOX21	1.077816875	0.0232316	protein_coding
ENSG00000138182	KIF20B	1.078109723	0.010677182	protein_coding
ENSG00000173542	MOB1B	1.078174407	0.011976281	protein_coding
ENSG00000049246	PER3	1.078261211	0.023682354	protein_coding
ENSG00000006607	FARP2	1.078269765	0.010146262	protein_coding
ENSG00000100815	TRIP11	1.07856508	0.011775411	protein_coding
ENSG00000121743	GJA3	1.078742611	0.01468817	protein_coding
ENSG00000001167	NFYA	1.079355352	0.010269611	protein_coding
ENSG00000173821	RNF213	1.079375531	0.010430504	protein_coding
ENSG00000170385	SLC30A1	1.079415059	0.011068486	protein_coding
ENSG00000109805	NCAPG	1.080651757	0.009862947	protein_coding
ENSG00000170448	NFXL1	1.081721117	0.011760997	protein_coding
ENSG00000184368	MAP7D2	1.08189269	0.01058941	protein_coding
ENSG00000148158	SNX30	1.082885646	0.010606117	protein_coding
ENSG00000111670	GNPTAB	1.083274281	0.010353301	protein_coding
ENSG00000152457	DCLRE1C	1.083304491	0.016739349	protein_coding
ENSG00000004700	RECQL	1.083413431	0.011412025	protein_coding
ENSG00000126777	KTN1	1.083528906	0.009322522	protein_coding
ENSG00000082258	CCNT2	1.083746359	0.010298582	protein_coding
ENSG00000046604	DSG2	1.083845856	0.009221969	protein_coding
ENSG00000157890	MEGF11	1.083962307	0.014625505	protein_coding
ENSG00000170802	FOXN2	1.084013961	0.012596877	protein_coding
ENSG00000082898	XPO1	1.084015649	0.009127393	protein_coding
ENSG00000101966	XIAP	1.085351242	0.009693935	protein_coding
ENSG00000121903	ZSCAN20	1.085485427	0.019846827	protein_coding

ENSG00000197147	LRRC8B	1.085575699	0.011800622	protein_coding
ENSG00000174106	LEMD3	1.086486369	0.010004243	protein_coding
ENSG00000165392	WRN	1.087163813	0.011410424	protein_coding
ENSG00000129534	MIS18BP1	1.087404645	0.010254609	protein_coding
ENSG00000106789	CORO2A	1.087755844	0.013844841	protein_coding
ENSG00000101974	ATP11C	1.088695522	0.009181906	protein_coding
ENSG00000171467	ZNF318	1.08869895	0.009617773	protein_coding
ENSG00000172888	ZNF621	1.08906903	0.009649859	protein_coding
ENSG00000107186	MPDZ	1.089107453	0.010204693	protein_coding
ENSG00000110172	CHORDC1	1.089454861	0.009232023	protein_coding
ENSG00000134352	IL6ST	1.089715448	0.009882086	protein_coding
ENSG00000163110	PDLIM5	1.090214155	0.009917943	protein_coding
ENSG00000122482	ZNF644	1.090544839	0.009394944	protein_coding
ENSG00000161813	LARP4	1.090727006	0.009151893	protein_coding
ENSG00000089057	SLC23A2	1.090895929	0.009445496	protein_coding
ENSG00000081189	MEF2C	1.090921327	0.01276696	protein_coding
ENSG00000203727	SAMD5	1.091602562	0.015474095	protein_coding
ENSG00000175175	PPM1E	1.092170826	0.011347225	protein_coding
ENSG00000143469	SYT14	1.092290462	0.026660679	protein_coding
ENSG00000118482	PHF3	1.092341971	0.009295413	protein_coding
ENSG00000115159	GPD2	1.093153501	0.009952303	protein_coding
ENSG00000138767	CNOT6L	1.093517551	0.010064972	protein_coding
ENSG00000188559	RALGAPA2	1.09468193	0.010037466	protein_coding
ENSG00000175155	YPEL2	1.095836641	0.014902143	protein_coding
ENSG00000162636	FAM102B	1.095956226	0.009925405	protein_coding
ENSG00000140382	HMG20A	1.096106615	0.009093066	protein_coding
ENSG00000068615	REEP1	1.096384826	0.012364955	protein_coding
ENSG00000164048	ZNF589	1.096785793	0.009138365	protein_coding
ENSG00000156052	GNAQ	1.096986706	0.00933819	protein_coding
ENSG00000135074	ADAM19	1.09714098	0.012146503	protein_coding
ENSG00000178691	SUZ12	1.097718562	0.008756457	protein_coding
ENSG00000089177	KIF16B	1.097864482	0.011237693	protein_coding
ENSG00000138741	TRPC3	1.098203468	0.027736032	protein_coding
ENSG00000092847	AGO1	1.098402729	0.00891084	protein_coding
ENSG00000163689	C3orf67	1.098556148	0.020520452	protein_coding
ENSG00000152409	JMY	1.098671428	0.009428196	protein_coding
ENSG00000143674	MAP3K21	1.099216465	0.008662727	protein_coding
ENSG00000184226	PCDH9	1.099446448	0.008854737	protein_coding
ENSG00000113163	COL4A3BP	1.099729569	0.009851842	protein_coding
ENSG00000198901	PRC1	1.099854744	0.027067397	protein_coding
ENSG00000163661	PTX3	1.100071244	0.036410837	protein_coding
ENSG00000107957	SH3PXD2A	1.100317421	0.008470301	protein_coding
ENSG00000175305	CCNE2	1.101560173	0.014191029	protein_coding

ENSG00000130856	ZNF236	1.101909814	0.011392861	protein_coding
ENSG00000213047	DENND1B	1.102105972	0.012067315	protein_coding
ENSG00000135842	FAM129A	1.102319841	0.013861412	protein_coding
ENSG00000163781	TOPBP1	1.102885416	0.008310982	protein_coding
ENSG00000171634	BPTF	1.103972899	0.008367226	protein_coding
ENSG00000054267	ARID4B	1.104591709	0.008637058	protein_coding
ENSG00000068305	MEF2A	1.104640138	0.008932358	protein_coding
ENSG00000169641	LUZP1	1.104992286	0.008825416	protein_coding
ENSG00000068366	ACSL4	1.105283871	0.008297571	protein_coding
ENSG00000078674	PCM1	1.105950611	0.008554698	protein_coding
ENSG00000174010	KLHL15	1.107254648	0.008401053	protein_coding
ENSG00000079308	TNS1	1.107317663	0.016977762	protein_coding
ENSG00000102053	ZC3H12B	1.107317663	0.016977762	protein_coding
ENSG00000198677	TTC37	1.108614874	0.007940994	protein_coding
ENSG00000112739	PRPF4B	1.10925909	0.007889241	protein_coding
ENSG00000173905	GOLIM4	1.109413156	0.008003863	protein_coding
ENSG00000108924	HLF	1.109525488	0.018055853	protein_coding
ENSG00000196967	ZNF585A	1.110576882	0.02705543	protein_coding
ENSG00000197782	ZNF780A	1.110804395	0.015351668	protein_coding
ENSG00000196535	MYO18A	1.111042334	0.008790301	protein_coding
ENSG00000158417	EIF5B	1.111356736	0.007677781	protein_coding
ENSG00000156140	ADAMTS3	1.111430501	0.011920075	protein_coding
ENSG00000165868	HSPA12A	1.111487322	0.009164374	protein_coding
ENSG00000184867	ARMCX2	1.111917612	0.045117399	protein_coding
ENSG00000285053	TBCE	1.113580042	0.012434369	protein_coding
ENSG00000152217	SETBP1	1.113783761	0.022887841	protein_coding
ENSG00000134313	KIDINS220	1.114194436	0.007984826	protein_coding
ENSG00000285976	AL135905.2	1.114358342	0.0210522	protein_coding
ENSG00000137142	IGFBPL1	1.114358342	0.0210522	protein_coding
ENSG00000106392	C1GALT1	1.114876979	0.010133152	protein_coding
ENSG00000154310	TNIK	1.114969216	0.008543037	protein_coding
ENSG00000147133	TAF1	1.115155002	0.008471868	protein_coding
ENSG00000163393	SLC22A15	1.115332042	0.018169896	protein_coding
ENSG00000184445	KNTC1	1.115938442	0.007794543	protein_coding
ENSG00000095015	MAP3K1	1.116125775	0.01052267	protein_coding
ENSG00000130396	AFDN	1.116834432	0.007407588	protein_coding
ENSG00000090905	TNRC6A	1.117185543	0.007858139	protein_coding
ENSG00000130508	PXDN	1.117368689	0.007955596	protein_coding
ENSG00000114331	ACAP2	1.11880525	0.008598588	protein_coding
ENSG00000178295	GEN1	1.119357825	0.008652584	protein_coding
ENSG00000165895	ARHGAP42	1.119810518	0.016931851	protein_coding
ENSG00000100027	YPEL1	1.120010916	0.046223025	protein_coding
ENSG00000138411	HECW2	1.120261919	0.01459975	protein_coding

ENSG00000152127	MGAT5	1.120763121	0.007535584	protein_coding
ENSG00000062370	ZNF112	1.121075312	0.028375462	protein_coding
ENSG00000205213	LGR4	1.12108462	0.007712337	protein_coding
ENSG00000177565	TBL1XR1	1.121114377	0.008118432	protein_coding
ENSG00000177707	NECTIN3	1.121178997	0.008164468	protein_coding
ENSG00000120756	PLS1	1.121571451	0.00884327	protein_coding
ENSG00000152332	UHMK1	1.12187664	0.007306434	protein_coding
ENSG00000110321	EIF4G2	1.121965092	0.006933677	protein_coding
ENSG00000075420	FNDC3B	1.122264813	0.009777446	protein_coding
ENSG00000118263	KLF7	1.122497814	0.016030979	protein_coding
ENSG00000171617	ENC1	1.122877788	0.01346087	protein_coding
ENSG00000146263	MMS22L	1.122904965	0.007851866	protein_coding
ENSG00000102531	FNDC3A	1.12301963	0.007839222	protein_coding
ENSG00000108256	NUFIP2	1.124610481	0.006994181	protein_coding
ENSG00000213020	ZNF611	1.12556494	0.015152451	protein_coding
ENSG00000139971	ARMH4	1.125814561	0.018169896	protein_coding
ENSG00000147234	FRMPD3	1.125814561	0.018169896	protein_coding
ENSG00000101109	STK4	1.125828052	0.00721163	protein_coding
ENSG00000166450	PRTG	1.126113297	0.010410923	protein_coding
ENSG00000071073	MGAT4A	1.126257924	0.011129538	protein_coding
ENSG00000186448	ZNF197	1.126607319	0.009471113	protein_coding
ENSG00000128872	TMOD2	1.126863778	0.00883645	protein_coding
ENSG00000151690	MFSD6	1.127401719	0.028675344	protein_coding
ENSG00000053747	LAMA3	1.127444224	0.00811088	protein_coding
ENSG00000170264	FAM161A	1.127800109	0.009738555	protein_coding
ENSG00000133059	DSTYK	1.128108814	0.007874025	protein_coding
ENSG00000048028	USP28	1.128526015	0.007520203	protein_coding
ENSG00000145833	DDX46	1.128544631	0.006805721	protein_coding
ENSG00000180488	MIGA1	1.128724999	0.008037186	protein_coding
ENSG00000185219	ZNF445	1.12912046	0.007278296	protein_coding
ENSG00000181544	FANCB	1.129239076	0.010821947	protein_coding
ENSG00000176595	KBTBD11	1.130359206	0.009451912	protein_coding
ENSG00000196705	ZNF431	1.130605704	0.008656678	protein_coding
ENSG00000173376	NDNF	1.132093598	0.012847833	protein_coding
ENSG00000177119	ANO6	1.132501783	0.00739013	protein_coding
ENSG00000197442	MAP3K5	1.133376423	0.008594256	protein_coding
ENSG00000109618	SEPSECS	1.134162364	0.012346948	protein_coding
ENSG00000158201	ABHD3	1.134180586	0.008476529	protein_coding
ENSG00000128731	HERC2	1.135373117	0.006631012	protein_coding
ENSG00000133985	TTC9	1.13549171	0.021709332	protein_coding
ENSG00000196876	SCN8A	1.13562999	0.016010439	protein_coding
ENSG00000148053	NTRK2	1.136525289	0.03991628	protein_coding
ENSG00000151718	WWC2	1.136549823	0.007507958	protein_coding

ENSG00000173706	HEG1	1.136727036	0.008024057	protein_coding
ENSG00000241343	RPL36A	1.136886767	0.007394402	protein_coding
ENSG00000015568	RGPD5	1.138225808	0.048686192	protein_coding
ENSG00000109654	TRIM2	1.138394487	0.007775591	protein_coding
ENSG00000095637	SORBS1	1.139676991	0.015217445	protein_coding
ENSG00000085719	CPNE3	1.140009692	0.006421113	protein_coding
ENSG00000184898	RBM43	1.140378509	0.02180232	protein_coding
ENSG00000130684	ZNF337	1.140378509	0.02180232	protein_coding
ENSG00000125257	ABCC4	1.141692603	0.00679721	protein_coding
ENSG00000146648	EGFR	1.141746527	0.008748302	protein_coding
ENSG00000185920	PTCH1	1.141791994	0.007322396	protein_coding
ENSG00000177853	ZNF518A	1.142443916	0.007177843	protein_coding
ENSG00000198399	ITSN2	1.142937908	0.007383045	protein_coding
ENSG00000118946	PCDH17	1.144565059	0.025239518	protein_coding
ENSG00000138685	FGF2	1.144899399	0.008633404	protein_coding
ENSG00000184110	EIF3C	1.145063571	0.006503007	protein_coding
ENSG00000124406	ATP8A1	1.14576443	0.008008687	protein_coding
ENSG00000112276	BVES	1.145794803	0.008416128	protein_coding
ENSG00000164663	USP49	1.145834144	0.007609191	protein_coding
ENSG00000069702	TGFBR3	1.14628283	0.00705522	protein_coding
ENSG00000117069	ST6GALNAC5	1.14647788	0.025850844	protein_coding
ENSG00000164741	DLC1	1.146848516	0.006953409	protein_coding
ENSG00000204262	COL5A2	1.147469845	0.019893	protein_coding
ENSG00000198265	HELZ	1.149658445	0.006314503	protein_coding
ENSG00000135541	AHI1	1.150030785	0.007579927	protein_coding
ENSG00000023287	RB1CC1	1.150196317	0.006177487	protein_coding
ENSG00000122591	FAM126A	1.150337281	0.006238098	protein_coding
ENSG00000032219	ARID4A	1.150564781	0.008314231	protein_coding
ENSG00000206530	CFAP44	1.150975044	0.01167195	protein_coding
ENSG00000175471	MCTP1	1.151609118	0.034718451	protein_coding
ENSG00000134909	ARHGAP32	1.152626759	0.007141562	protein_coding
ENSG00000104447	TRPS1	1.152626759	0.007141562	protein_coding
ENSG00000085449	WDFY1	1.152853131	0.006453321	protein_coding
ENSG00000163697	APBB2	1.153559123	0.007701262	protein_coding
ENSG00000089505	CMTM1	1.155162721	0.041652925	protein_coding
ENSG00000129292	PHF20L1	1.155988291	0.006330826	protein_coding
ENSG00000152894	PTPRK	1.156077239	0.006433773	protein_coding
ENSG00000159023	EPB41	1.156242407	0.005580024	protein_coding
ENSG00000180628	PCGF5	1.156345915	0.006339729	protein_coding
ENSG00000164023	SGMS2	1.156696565	0.007806019	protein_coding
ENSG00000135976	ANKRD36	1.157204349	0.008474514	protein_coding
ENSG00000170471	RALGAPB	1.157585041	0.005952155	protein_coding
ENSG00000188092	GPR89B	1.158168666	0.009288424	protein_coding

ENSG00000091157	WDR7	1.159394014	0.008953747	protein_coding
ENSG00000197121	PGAP1	1.159667998	0.006215736	protein_coding
ENSG00000128923	MINDY2	1.159693343	0.007928641	protein_coding
ENSG00000159399	HK2	1.159787905	0.00569113	protein_coding
ENSG00000008869	HEATR5B	1.159868867	0.006243643	protein_coding
ENSG00000198018	ENTPD7	1.160902875	0.006503797	protein_coding
ENSG00000058272	PPP1R12A	1.161339235	0.005902502	protein_coding
ENSG00000084676	NCOA1	1.161478684	0.006262382	protein_coding
ENSG00000171033	PKIA	1.161478684	0.006262382	protein_coding
ENSG00000112293	GPLD1	1.161816344	0.022901013	protein_coding
ENSG00000198947	DMD	1.161964356	0.007235003	protein_coding
ENSG00000254535	PABPC4L	1.162524881	0.007143204	protein_coding
ENSG00000197045	GMFB	1.16256989	0.005571159	protein_coding
ENSG00000105778	AVL9	1.16272964	0.005964546	protein_coding
ENSG00000103657	HERC1	1.162799106	0.006001294	protein_coding
ENSG00000101745	ANKRD12	1.163943986	0.008162223	protein_coding
ENSG00000153993	SEMA3D	1.164855193	0.010938752	protein_coding
ENSG00000118513	MYB	1.164873934	0.011851263	protein_coding
ENSG00000120278	PLEKHG1	1.164895273	0.012955916	protein_coding
ENSG00000165525	NEMF	1.165582389	0.006063089	protein_coding
ENSG00000170873	MTSS1	1.166094893	0.009618724	protein_coding
ENSG00000131389	SLC6A6	1.166362297	0.0056118	protein_coding
ENSG00000164164	OTUD4	1.166714094	0.005536523	protein_coding
ENSG00000108582	CPD	1.166882869	0.005320749	protein_coding
ENSG00000145819	ARHGAP26	1.166920814	0.007778645	protein_coding
ENSG00000140807	NKD1	1.166965773	0.006921836	protein_coding
ENSG00000171940	ZNF217	1.167365064	0.007176051	protein_coding
ENSG00000091039	OSBPL8	1.167449015	0.005454422	protein_coding
ENSG00000107854	TNKS2	1.167950337	0.005435881	protein_coding
ENSG00000171914	TLN2	1.16819193	0.007472681	protein_coding
ENSG00000185760	KCNQ5	1.168537605	0.008007671	protein_coding
ENSG00000053770	AP5M1	1.169047017	0.005876926	protein_coding
ENSG00000048649	RSF1	1.169466621	0.00558201	protein_coding
ENSG00000110841	PPFIBP1	1.169650477	0.006145153	protein_coding
ENSG00000105877	DNAH11	1.169813831	0.012318672	protein_coding
ENSG00000168172	HOOK3	1.169942146	0.005595973	protein_coding
ENSG00000089916	GPATCH2L	1.169978443	0.006072561	protein_coding
ENSG00000175414	ARL10	1.170218889	0.005374219	protein_coding
ENSG00000119684	MLH3	1.170522416	0.03592403	protein_coding
ENSG00000159496	RGL4	1.170522416	0.03592403	protein_coding
ENSG00000143776	CDC42BPA	1.170834274	0.005197762	protein_coding
ENSG00000084710	EFR3B	1.171150426	0.008276617	protein_coding
ENSG00000119139	TJP2	1.172075924	0.045931264	protein_coding

ENSG00000149212	SESN3	1.172175502	0.00550344	protein_coding
ENSG00000143869	GDF7	1.172218601	0.007022374	protein_coding
ENSG00000103995	CEP152	1.172276948	0.007173549	protein_coding
ENSG00000145416	1-Mar	1.172532653	0.017114431	protein_coding
ENSG00000213079	SCAF8	1.173322456	0.005297886	protein_coding
ENSG00000137941	TTLL7	1.17456715	0.007615088	protein_coding
ENSG00000221818	EBF2	1.174599644	0.015901775	protein_coding
ENSG00000153561	RMND5A	1.175641551	0.004948431	protein_coding
ENSG00000121274	TENT4B	1.176200652	0.00568697	protein_coding
ENSG00000121879	PIK3CA	1.176897368	0.008165538	protein_coding
ENSG00000136925	TSTD2	1.17693208	0.00743448	protein_coding
ENSG00000101868	POLA1	1.17701459	0.005100946	protein_coding
ENSG00000114857	NKTR	1.177712353	0.004923595	protein_coding
ENSG00000001630	CYP51A1	1.178143925	0.007751496	protein_coding
ENSG00000024048	UBR2	1.178694749	0.005462541	protein_coding
ENSG00000186496	ZNF396	1.181097522	0.036582079	protein_coding
ENSG00000115604	IL18R1	1.181355563	0.017128527	protein_coding
ENSG00000136731	UGGT1	1.181379007	0.004812694	protein_coding
ENSG00000197608	ZNF841	1.181423989	0.013159239	protein_coding
ENSG00000163507	CIP2A	1.182855932	0.004966228	protein_coding
ENSG00000170017	ALCAM	1.182875091	0.004929785	protein_coding
ENSG00000075340	ADD2	1.183081391	0.004892911	protein_coding
ENSG00000198862	LTN1	1.184619298	0.005332629	protein_coding
ENSG00000080561	MID2	1.186183622	0.0052037	protein_coding
ENSG00000100731	PCNX1	1.186900356	0.004840491	protein_coding
ENSG00000112309	B3GAT2	1.187242306	0.017130461	protein_coding
ENSG00000213533	STIMATE	1.187424216	0.02704513	protein_coding
ENSG00000123575	FAM199X	1.188255859	0.004436324	protein_coding
ENSG00000156103	MMP16	1.188376235	0.009234027	protein_coding
ENSG00000104356	POP1	1.189736023	0.004667878	protein_coding
ENSG00000060237	WNK1	1.19016527	0.004339058	protein_coding
ENSG00000189127	ANKRD34B	1.190190302	0.017121806	protein_coding
ENSG00000140090	SLC24A4	1.190462343	0.012229212	protein_coding
ENSG00000138735	PDE5A	1.192351752	0.007018704	protein_coding
ENSG00000176597	B3GNT5	1.192572339	0.008118329	protein_coding
ENSG00000168807	SNTB2	1.193773194	0.004718996	protein_coding
ENSG00000092148	HECTD1	1.194357512	0.004352082	protein_coding
ENSG00000170113	NIPA1	1.194541882	0.005911009	protein_coding
ENSG00000179104	TMTC2	1.195584374	0.007861262	protein_coding
ENSG00000196914	ARHGEF12	1.19627792	0.004257165	protein_coding
ENSG00000198604	BAZ1A	1.196424767	0.004284213	protein_coding
ENSG00000150054	MPP7	1.197051179	0.00682873	protein_coding
ENSG00000145725	PPIP5K2	1.197353934	0.004707506	protein_coding

ENSG00000197102	DYNC1H1	1.197468857	0.00405455	protein_coding
ENSG00000107443	CCNJ	1.198222922	0.004798515	protein_coding
ENSG00000151458	ANKRD50	1.198650441	0.005185987	protein_coding
ENSG00000186908	ZDHHC17	1.199748794	0.004711675	protein_coding
ENSG00000120549	KIAA1217	1.199794239	0.017131865	protein_coding
ENSG00000169629	RGPD8	1.199794239	0.017131865	protein_coding
ENSG00000175893	ZDHHC21	1.20016053	0.006163913	protein_coding
ENSG00000143756	FBXO28	1.200616082	0.004248241	protein_coding
ENSG00000196247	ZNF107	1.200802243	0.005948618	protein_coding
ENSG00000174695	TMEM167A	1.201621793	0.004282224	protein_coding
ENSG00000169855	ROBO1	1.202280043	0.004021017	protein_coding
ENSG00000181690	PLAG1	1.202333617	0.00533536	protein_coding
ENSG00000175066	GK5	1.202835212	0.004789764	protein_coding
ENSG00000153922	CHD1	1.203241886	0.004127184	protein_coding
ENSG00000115525	ST3GAL5	1.203538067	0.007525076	protein_coding
ENSG00000196233	LCOR	1.204288371	0.004109727	protein_coding
ENSG00000261609	GAN	1.205675207	0.004751672	protein_coding
ENSG00000135338	LCA5	1.207304721	0.015760642	protein_coding
ENSG00000164190	NIPBL	1.207504999	0.004164305	protein_coding
ENSG00000198743	SLC5A3	1.207729782	0.004409269	protein_coding
ENSG00000065923	SLC9A7	1.209131091	0.005567035	protein_coding
ENSG00000101680	LAMA1	1.209762524	0.004488494	protein_coding
ENSG00000116191	RALGPS2	1.212200013	0.004120511	protein_coding
ENSG00000163629	PTPN13	1.212205642	0.003803323	protein_coding
ENSG00000273136	NBPF26	1.212365977	0.017098491	protein_coding
ENSG00000136381	IREB2	1.212575204	0.003730048	protein_coding
ENSG00000114450	GNB4	1.214234742	0.00419398	protein_coding
ENSG00000197323	TRIM33	1.214324484	0.003816026	protein_coding
ENSG00000172493	AFF1	1.214416258	0.004203528	protein_coding
ENSG00000140526	ABHD2	1.21461636	0.003975886	protein_coding
ENSG00000139737	SLAIN1	1.214736758	0.004094524	protein_coding
ENSG00000118007	STAG1	1.214776614	0.004162846	protein_coding
ENSG00000069956	MAPK6	1.215022558	0.004299137	protein_coding
ENSG00000184675	AMER1	1.215501649	0.004255314	protein_coding
ENSG00000079482	OPHN1	1.215598895	0.006065334	protein_coding
ENSG00000044524	EPHA3	1.216190894	0.006116414	protein_coding
ENSG00000173611	SCAI	1.216505144	0.005800395	protein_coding
ENSG00000008086	CDKL5	1.216546975	0.007386173	protein_coding
ENSG00000138778	CENPE	1.217066011	0.004029999	protein_coding
ENSG00000031081	ARHGAP31	1.217095575	0.009728415	protein_coding
ENSG00000137992	DBT	1.217764903	0.004333489	protein_coding
ENSG00000139211	AMIGO2	1.219325074	0.027887688	protein_coding
ENSG00000070961	ATP2B1	1.219397616	0.003681775	protein_coding

ENSG00000124151	NCOA3	1.219490819	0.004156668	protein_coding
ENSG00000176681	LRRC37A	1.219913934	0.021314597	protein_coding
ENSG00000100344	PNPLA3	1.220204794	0.004746314	protein_coding
ENSG00000100354	TNRC6B	1.220223675	0.00400174	protein_coding
ENSG00000163349	HIPK1	1.221186842	0.003671967	protein_coding
ENSG00000132953	XPO4	1.221195423	0.003817981	protein_coding
ENSG00000080345	RIF1	1.221205586	0.003550965	protein_coding
ENSG00000139354	GAS2L3	1.221291302	0.00500998	protein_coding
ENSG00000198648	STK39	1.221862458	0.004622279	protein_coding
ENSG00000039560	RAI14	1.224468531	0.003652426	protein_coding
ENSG00000086758	HUWE1	1.227579557	0.003235186	protein_coding
ENSG00000181827	RFX7	1.228633815	0.004267074	protein_coding
ENSG00000163681	SLMAP	1.229958332	0.003454346	protein_coding
ENSG00000171865	RNASEH1	1.230066549	0.003846212	protein_coding
ENSG00000156299	TIAM1	1.230169601	0.003700707	protein_coding
ENSG00000137760	ALKBH8	1.23052485	0.004242061	protein_coding
ENSG00000177034	MTX3	1.23053528	0.003720145	protein_coding
ENSG00000169213	RAB3B	1.230758535	0.005177661	protein_coding
ENSG00000165650	PDZD8	1.230881988	0.003464882	protein_coding
ENSG00000101333	PLCB4	1.230887342	0.008296396	protein_coding
ENSG00000048707	VPS13D	1.231298783	0.003825481	protein_coding
ENSG00000148516	ZEB1	1.2318162	0.0042942	protein_coding
ENSG00000119900	OGFRL1	1.232420651	0.003761721	protein_coding
ENSG00000006432	MAP3K9	1.232882357	0.004430174	protein_coding
ENSG00000151694	ADAM17	1.236190504	0.003879697	protein_coding
ENSG00000198369	SPRED2	1.236339287	0.005650375	protein_coding
ENSG00000169379	ARL13B	1.237752616	0.004093629	protein_coding
ENSG00000154447	SH3RF1	1.237906744	0.004755714	protein_coding
ENSG00000173480	ZNF417	1.238970891	0.010967701	protein_coding
ENSG00000203667	COX20	1.239225809	0.01701213	protein_coding
ENSG00000129595	EPB41L4A	1.240337953	0.012477586	protein_coding
ENSG00000146197	SCUBE3	1.240337953	0.012477586	protein_coding
ENSG00000154080	CHST9	1.240340583	0.019009984	protein_coding
ENSG00000078269	SYNJ2	1.240590326	0.003855677	protein_coding
ENSG00000176018	LYSMD3	1.24119635	0.003594807	protein_coding
ENSG00000123200	ZC3H13	1.241445629	0.003104751	protein_coding
ENSG00000150764	DIXDC1	1.241550867	0.003976638	protein_coding
ENSG00000138018	SELENOI	1.241948556	0.003047558	protein_coding
ENSG00000128567	PODXL	1.242040252	0.003639436	protein_coding
ENSG00000056097	ZFR	1.243592718	0.002979233	protein_coding
ENSG00000156802	ATAD2	1.243602927	0.003133252	protein_coding
ENSG00000129317	PUS7L	1.243644719	0.003327774	protein_coding
ENSG00000080298	RFX3	1.244348355	0.010264258	protein_coding

ENSG00000057294	PKP2	1.24456974	0.003809524	protein_coding
ENSG00000115183	TANC1	1.24493668	0.003401525	protein_coding
ENSG00000119912	IDE	1.247189455	0.003100366	protein_coding
ENSG00000204519	ZNF551	1.247762422	0.006049189	protein_coding
ENSG00000136928	GABBR2	1.247866859	0.017035557	protein_coding
ENSG00000116406	EDEM3	1.248399434	0.00316659	protein_coding
ENSG00000047365	ARAP2	1.24948925	0.00766319	protein_coding
ENSG00000112297	CRYBG1	1.249572428	0.034842559	protein_coding
ENSG00000149308	NPAT	1.250045934	0.003548928	protein_coding
ENSG00000108510	MED13	1.250357647	0.003022885	protein_coding
ENSG00000100592	DAAM1	1.251011066	0.003567302	protein_coding
ENSG00000067992	PDK3	1.251072865	0.003801391	protein_coding
ENSG00000139324	TMTC3	1.251701952	0.003142383	protein_coding
ENSG00000143195	ILDR2	1.252976645	0.00473243	protein_coding
ENSG00000130038	CRACR2A	1.253546532	0.00570038	protein_coding
ENSG00000197044	ZNF441	1.253635819	0.021553211	protein_coding
ENSG00000077157	PPP1R12B	1.254047072	0.003535271	protein_coding
ENSG00000152270	PDE3B	1.254146812	0.003169466	protein_coding
ENSG00000170537	TMC7	1.254275868	0.012341791	protein_coding
ENSG00000198087	CD2AP	1.254631883	0.002937117	protein_coding
ENSG00000180626	ZNF594	1.254805944	0.005302615	protein_coding
ENSG00000147526	TACC1	1.255070993	0.003200811	protein_coding
ENSG00000166750	SLFN5	1.257389961	0.010923125	protein_coding
ENSG00000149311	ATM	1.257771656	0.002926852	protein_coding
ENSG00000179295	PTPN11	1.258568774	0.002591764	protein_coding
ENSG00000174469	CNTNAP2	1.258848998	0.003020605	protein_coding
ENSG00000033867	SLC4A7	1.259534871	0.003162945	protein_coding
ENSG00000169554	ZEB2	1.259727227	0.003543923	protein_coding
ENSG00000118200	CAMSAP2	1.259877317	0.002786698	protein_coding
ENSG00000162139	NEU3	1.26046636	0.003152465	protein_coding
ENSG00000135968	GCC2	1.261308723	0.003030894	protein_coding
ENSG00000102189	EEA1	1.261315999	0.00336665	protein_coding
ENSG00000159140	SON	1.261723673	0.002512339	protein_coding
ENSG00000187605	TET3	1.262222047	0.002934861	protein_coding
ENSG00000137561	TTPA	1.262325878	0.049028188	protein_coding
ENSG00000159082	SYNJ1	1.263441995	0.003879575	protein_coding
ENSG00000134278	SPIRE1	1.263709383	0.003271053	protein_coding
ENSG00000173889	PHC3	1.264004919	0.003479319	protein_coding
ENSG00000038382	TRIO	1.26591638	0.002582354	protein_coding
ENSG00000186462	NAP1L2	1.267522672	0.035481962	protein_coding
ENSG00000182253	SYNM	1.267621855	0.006778375	protein_coding
ENSG00000119314	PTBP3	1.268557653	0.002531559	protein_coding
ENSG00000183723	CMTM4	1.269250812	0.002514189	protein_coding

ENSG00000204186	ZDBF2	1.270602917	0.002901067	protein_coding
ENSG00000163714	U2SURP	1.27093422	0.002340693	protein_coding
ENSG00000132466	ANKRD17	1.271913133	0.002379147	protein_coding
ENSG00000129003	VPS13C	1.272285642	0.003020387	protein_coding
ENSG00000156273	BACH1	1.272816113	0.003174928	protein_coding
ENSG00000168702	LRP1B	1.273383188	0.013848142	protein_coding
ENSG00000101384	JAG1	1.273443495	0.006305941	protein_coding
ENSG00000075391	RASAL2	1.273856134	0.002849171	protein_coding
ENSG00000145391	SETD7	1.274193071	0.002355438	protein_coding
ENSG00000117724	CENPF	1.274412535	0.002283339	protein_coding
ENSG00000143951	WDPCP	1.274967172	0.011287195	protein_coding
ENSG00000107290	SETX	1.276361575	0.002545998	protein_coding
ENSG00000171502	COL24A1	1.276637702	0.013676595	protein_coding
ENSG00000213694	S1PR3	1.277273111	0.005828011	protein_coding
ENSG00000198589	LRBA	1.278382383	0.002397096	protein_coding
ENSG00000139292	LGR5	1.278965769	0.00450753	protein_coding
ENSG00000187068	C3orf70	1.280266744	0.009874341	protein_coding
ENSG00000134982	APC	1.281961651	0.002561151	protein_coding
ENSG00000177570	SAMD12	1.28387792	0.013765123	protein_coding
ENSG00000170325	PRDM10	1.284546675	0.003221749	protein_coding
ENSG00000167377	ZNF23	1.284610776	0.016874371	protein_coding
ENSG00000138758	11-Sep	1.285211017	0.002121439	protein_coding
ENSG00000164930	FZD6	1.286137234	0.003251821	protein_coding
ENSG00000089682	RBM41	1.28637593	0.002710795	protein_coding
ENSG00000153904	DDAH1	1.287274514	0.002456858	protein_coding
ENSG00000143702	CEP170	1.287388694	0.002161066	protein_coding
ENSG00000196712	NF1	1.287392021	0.002272214	protein_coding
ENSG00000073712	FERMT2	1.287505758	0.002313751	protein_coding
ENSG00000258102	MAP1LC3B2	1.287936013	0.013582069	protein_coding
ENSG00000115084	SLC35F5	1.287978336	0.002790225	protein_coding
ENSG00000144909	OSBPL11	1.29051358	0.002407419	protein_coding
ENSG00000133687	TMTC1	1.291801343	0.003205838	protein_coding
ENSG00000183091	NEB	1.292046702	0.029542889	protein_coding
ENSG00000148358	GPR107	1.294162403	0.002131069	protein_coding
ENSG00000123066	MED13L	1.295392249	0.002136913	protein_coding
ENSG00000163637	PRICKLE2	1.295396959	0.021782468	protein_coding
ENSG00000117523	PRRC2C	1.296027494	0.001919239	protein_coding
ENSG00000138756	BMP2K	1.299141356	0.003796442	protein_coding
ENSG00000126070	AGO3	1.299560112	0.002407141	protein_coding
ENSG00000166025	AMOTL1	1.299561867	0.002008676	protein_coding
ENSG00000162722	TRIM58	1.299850236	0.030298791	protein_coding
ENSG00000051341	POLQ	1.300360311	0.002320423	protein_coding
ENSG00000115977	AAK1	1.300863771	0.002555936	protein_coding

ENSG00000196628	TCF4	1.302774644	0.002259881	protein_coding
ENSG00000153107	ANAPC1	1.305116302	0.001880104	protein_coding
ENSG00000243156	MICAL3	1.306107684	0.002085984	protein_coding
ENSG00000091844	RGS17	1.306174247	0.00395452	protein_coding
ENSG00000196110	ZNF699	1.307942343	0.040101413	protein_coding
ENSG00000110427	KIAA1549L	1.308314513	0.00299219	protein_coding
ENSG00000170153	RNF150	1.310072675	0.002181483	protein_coding
ENSG00000151116	UEVLD	1.310681038	0.004425893	protein_coding
ENSG00000082805	ERC1	1.310857736	0.001928541	protein_coding
ENSG00000152056	AP1S3	1.311187382	0.003209325	protein_coding
ENSG00000101596	SMCHD1	1.311595439	0.001908598	protein_coding
ENSG00000134318	ROCK2	1.311880345	0.001909946	protein_coding
ENSG00000162378	ZYG11B	1.31277731	0.001997961	protein_coding
ENSG00000150995	ITPR1	1.312921517	0.002602824	protein_coding
ENSG00000023516	AKAP11	1.314214116	0.001948325	protein_coding
ENSG00000118058	KMT2A	1.314377573	0.001744004	protein_coding
ENSG00000065613	SLK	1.314496428	0.00174504	protein_coding
ENSG00000104067	TJP1	1.314800684	0.001940283	protein_coding
ENSG00000112249	ASCC3	1.31593227	0.001819647	protein_coding
ENSG00000140396	NCOA2	1.316768288	0.001974792	protein_coding
ENSG00000129422	MTUS1	1.317931617	0.002524632	protein_coding
ENSG00000060749	QSER1	1.31797037	0.001759877	protein_coding
ENSG00000138448	ITGAV	1.321079609	0.001937253	protein_coding
ENSG00000182866	LCK	1.321281194	0.030695741	protein_coding
ENSG00000094975	SUCO	1.321763425	0.001789976	protein_coding
ENSG00000124251	TP53TG5	1.322356415	0.046163285	protein_coding
ENSG00000101746	NOL4	1.322791584	0.00868018	protein_coding
ENSG00000091656	ZFHX4	1.323485821	0.001905143	protein_coding
ENSG00000204217	BMPR2	1.32406381	0.001861793	protein_coding
ENSG00000134250	NOTCH2	1.326804877	0.001536936	protein_coding
ENSG00000092531	SNAP23	1.326872366	0.004091456	protein_coding
ENSG00000198597	ZNF536	1.327031184	0.003259596	protein_coding
ENSG00000164151	ICE1	1.32933756	0.001627379	protein_coding
ENSG00000095564	BTAF1	1.329934127	0.001557033	protein_coding
ENSG00000143479	DYRK3	1.330727411	0.002318466	protein_coding
ENSG00000116132	PRRX1	1.330997078	0.010994238	protein_coding
ENSG00000165997	ARL5B	1.331650203	0.001589295	protein_coding
ENSG00000128000	ZNF780B	1.333586029	0.00489751	protein_coding
ENSG00000167637	ZNF283	1.334518408	0.004302505	protein_coding
ENSG00000141424	SLC39A6	1.338610138	0.001430901	protein_coding
ENSG00000136874	STX17	1.338662448	0.002432595	protein_coding
ENSG00000138593	SECISBP2L	1.338702357	0.002257411	protein_coding
ENSG00000129167	TPH1	1.339444877	0.040993521	protein_coding

ENSG00000146859	TMEM140	1.33996799	0.002333097	protein_coding
ENSG00000139220	PPFIA2	1.34047278	0.025675336	protein_coding
ENSG00000143322	ABL2	1.343899557	0.001468436	protein_coding
ENSG00000111961	SASH1	1.344340781	0.001696407	protein_coding
ENSG00000153827	TRIP12	1.345138649	0.001339954	protein_coding
ENSG00000048405	ZNF800	1.348389785	0.001984889	protein_coding
ENSG00000065534	MYLK	1.348900802	0.002712831	protein_coding
ENSG00000157741	UBN2	1.3489697	0.001494564	protein_coding
ENSG00000083168	KAT6A	1.350424799	0.001378741	protein_coding
ENSG00000164236	ANKRD33B	1.353053287	0.002047218	protein_coding
ENSG00000183718	TRIM52	1.353240506	0.001808726	protein_coding
ENSG00000257093	KIAA1147	1.354784439	0.001300362	protein_coding
ENSG00000187079	TEAD1	1.355554067	0.001365024	protein_coding
ENSG00000086570	FAT2	1.355666968	0.018991473	protein_coding
ENSG00000187240	DYNC2H1	1.356452227	0.001328441	protein_coding
ENSG00000198707	CEP290	1.357207935	0.001541717	protein_coding
ENSG00000151617	EDNRA	1.357619694	0.003522678	protein_coding
ENSG00000183044	ABAT	1.358898563	0.002277639	protein_coding
ENSG00000145703	IQGAP2	1.36007383	0.001531074	protein_coding
ENSG00000188994	ZNF292	1.360527512	0.00162678	protein_coding
ENSG00000144824	PHLDB2	1.362722229	0.00189671	protein_coding
ENSG00000132938	MTUS2	1.36285093	0.038410682	protein_coding
ENSG00000221843	C2orf16	1.366131116	0.008838507	protein_coding
ENSG00000058063	ATP11B	1.366674414	0.00136989	protein_coding
ENSG00000134852	CLOCK	1.368332891	0.001530874	protein_coding
ENSG00000137968	SLC44A5	1.369008817	0.00200933	protein_coding
ENSG00000188738	FSIP2	1.369543206	0.02203199	protein_coding
ENSG00000123636	BAZ2B	1.369990474	0.002313244	protein_coding
ENSG00000170456	DENND5B	1.370049169	0.001439554	protein_coding
ENSG00000172915	NBEA	1.370195642	0.002051901	protein_coding
ENSG00000010818	HIVEP2	1.371085209	0.001614058	protein_coding
ENSG00000120868	APAF1	1.373579251	0.001377125	protein_coding
ENSG00000100580	TMED8	1.374221968	0.001140051	protein_coding
ENSG00000138829	FBN2	1.374717329	0.001072353	protein_coding
ENSG00000141449	GREB1L	1.376566229	0.001192915	protein_coding
ENSG00000088387	DOCK9	1.377427196	0.001388758	protein_coding
ENSG00000066084	DIP2B	1.378392983	0.001120922	protein_coding
ENSG00000091409	ITGA6	1.378875656	0.001468922	protein_coding
ENSG00000180008	SOCS4	1.37980562	0.001153415	protein_coding
ENSG00000169760	NLGN1	1.380395829	0.00220407	protein_coding
ENSG00000154153	RETREG1	1.381980396	0.003129389	protein_coding
ENSG00000213949	ITGA1	1.382427664	0.002177706	protein_coding
ENSG00000114805	PLCH1	1.382660314	0.001662164	protein_coding

ENSG00000169116	PARM1	1.382660698	0.001830807	protein_coding
ENSG00000163328	GPR155	1.385189214	0.001835247	protein_coding
ENSG00000111647	UHRF1BP1L	1.385453945	0.001283402	protein_coding
ENSG00000164742	ADCY1	1.385921812	0.001146011	protein_coding
ENSG00000170743	SYT9	1.386550912	0.033173684	protein_coding
ENSG00000130779	CLIP1	1.386734427	0.001101247	protein_coding
ENSG00000131019	ULBP3	1.38689479	0.002997333	protein_coding
ENSG00000103064	SLC7A6	1.38748454	0.001130026	protein_coding
ENSG00000169871	TRIM56	1.387703987	0.001751714	protein_coding
ENSG00000083857	FAT1	1.388148698	0.001007604	protein_coding
ENSG00000116991	SIPA1L2	1.388805354	0.001183196	protein_coding
ENSG00000163428	LRRC58	1.389553607	0.00095226	protein_coding
ENSG00000170921	TANC2	1.39072382	0.001116803	protein_coding
ENSG00000127914	AKAP9	1.393355622	0.001079737	protein_coding
ENSG00000151914	DST	1.393563213	0.000985145	protein_coding
ENSG00000096696	DSP	1.393673346	0.000889575	protein_coding
ENSG00000155850	SLC26A2	1.393816551	0.001107355	protein_coding
ENSG00000145147	SLIT2	1.393909626	0.000916878	protein_coding
ENSG00000196417	ZNF765	1.394121677	0.002840449	protein_coding
ENSG00000164309	CMYA5	1.395063713	0.039238489	protein_coding
ENSG00000065361	ERBB3	1.395290267	0.002994583	protein_coding
ENSG00000123908	AGO2	1.395378752	0.000903201	protein_coding
ENSG00000053254	FOXN3	1.397061988	0.001074531	protein_coding
ENSG00000066739	ATG2B	1.39716328	0.001028425	protein_coding
ENSG00000174373	RALGAPA1	1.39743726	0.001103594	protein_coding
ENSG00000164251	F2RL1	1.397546457	0.001357072	protein_coding
ENSG00000180660	MAB21L1	1.398039412	0.018902448	protein_coding
ENSG00000096968	JAK2	1.39923931	0.002975043	protein_coding
ENSG00000151806	GUF1	1.399311383	0.00105104	protein_coding
ENSG00000135333	EPHA7	1.399512186	0.001028039	protein_coding
ENSG00000155846	PPARGC1B	1.402527532	0.000958942	protein_coding
ENSG00000132405	TBC1D14	1.405553177	0.001024446	protein_coding
ENSG00000083097	DOP1A	1.407857083	0.0012748	protein_coding
ENSG00000100505	TRIM9	1.409643913	0.001152358	protein_coding
ENSG00000106261	ZKSCAN1	1.410006062	0.000889163	protein_coding
ENSG00000110395	CBL	1.410257773	0.000865736	protein_coding
ENSG00000188647	PTAR1	1.410325411	0.000893112	protein_coding
ENSG00000115808	STRN	1.413357164	0.000888161	protein_coding
ENSG00000197472	ZNF695	1.413670443	0.022113468	protein_coding
ENSG00000093217	XYLB	1.415238841	0.001087881	protein_coding
ENSG00000178917	ZNF852	1.416905977	0.004026014	protein_coding
ENSG00000158528	PPP1R9A	1.417921867	0.0026832	protein_coding
ENSG00000067900	ROCK1	1.417963046	0.000783589	protein_coding

ENSG00000125686	MED1	1.417965999	0.000769038	protein_coding
ENSG00000137812	KNL1	1.418578081	0.000891411	protein_coding
ENSG00000115306	SPTBN1	1.420025259	0.000723409	protein_coding
ENSG00000164715	LMTK2	1.420103713	0.000884505	protein_coding
ENSG00000114439	BBX	1.420653416	0.00076483	protein_coding
ENSG00000106086	PLEKHA8	1.421915785	0.000864017	protein_coding
ENSG00000127603	MACF1	1.422167022	0.000726481	protein_coding
ENSG00000182141	ZNF708	1.422672557	0.002291731	protein_coding
ENSG00000058668	ATP2B4	1.423079493	0.00084901	protein_coding
ENSG00000173930	SLCO4C1	1.425983644	0.016215654	protein_coding
ENSG00000048740	CELF2	1.427929663	0.000849472	protein_coding
ENSG00000113658	SMAD5	1.428140649	0.000703288	protein_coding
ENSG00000103994	ZNF106	1.428656509	0.000737839	protein_coding
ENSG00000127481	UBR4	1.431306342	0.000661028	protein_coding
ENSG00000106714	CNTNAP3	1.433718867	0.001920418	protein_coding
ENSG00000177311	ZBTB38	1.4339114	0.000995527	protein_coding
ENSG00000263001	GTF2I	1.433983448	0.000671257	protein_coding
ENSG00000167548	KMT2D	1.434135573	0.000634666	protein_coding
ENSG00000196391	ZNF774	1.434507728	0.002136254	protein_coding
ENSG00000112893	MAN2A1	1.434749365	0.000851112	protein_coding
ENSG00000137145	DENND4C	1.434942865	0.000900819	protein_coding
ENSG00000197535	MYO5A	1.437068109	0.000812815	protein_coding
ENSG00000072364	AFF4	1.437497583	0.000677711	protein_coding
ENSG00000214029	ZNF891	1.437932546	0.001296262	protein_coding
ENSG00000163960	UBXN7	1.438554842	0.000694268	protein_coding
ENSG00000172869	DMXL1	1.438794507	0.000827875	protein_coding
ENSG00000038219	BOD1L1	1.440017267	0.00070418	protein_coding
ENSG00000164199	ADGRV1	1.442616119	0.001114626	protein_coding
ENSG00000003989	SLC7A2	1.44274108	0.000656907	protein_coding
ENSG00000165185	KIAA1958	1.443526931	0.00086897	protein_coding
ENSG00000134709	HOOK1	1.445799753	0.000669691	protein_coding
ENSG00000169499	PLEKHA2	1.447323388	0.001820048	protein_coding
ENSG00000165105	RASEF	1.448227086	0.000763221	protein_coding
ENSG00000075223	SEMA3C	1.448589384	0.001616556	protein_coding
ENSG00000174197	MGA	1.4489608	0.000636936	protein_coding
ENSG00000166833	NAV2	1.45040959	0.00209876	protein_coding
ENSG00000118193	KIF14	1.450580429	0.000645856	protein_coding
ENSG00000144674	GOLGA4	1.451469652	0.000598131	protein_coding
ENSG00000152495	CAMK4	1.45202491	0.001299058	protein_coding
ENSG00000054523	KIF1B	1.452519995	0.00060047	protein_coding
ENSG00000137502	RAB30	1.452798921	0.001947286	protein_coding
ENSG00000203668	CHML	1.454257351	0.000559216	protein_coding
ENSG00000124177	CHD6	1.455200546	0.000634542	protein_coding

ENSG00000085563	ABCB1	1.458296611	0.001297139	protein_coding
ENSG00000075539	FRYL	1.458598876	0.000815624	protein_coding
ENSG00000153201	RANBP2	1.459565837	0.000521798	protein_coding
ENSG00000083312	TNPO1	1.461451784	0.000499952	protein_coding
ENSG00000184305	CCSER1	1.461701009	0.006876664	protein_coding
ENSG00000179240	GVQW3	1.462041413	0.001668912	protein_coding
ENSG00000164684	ZNF704	1.463419888	0.000560617	protein_coding
ENSG00000167306	MYO5B	1.463824232	0.001757906	protein_coding
ENSG00000162409	PRKAA2	1.466117602	0.00057315	protein_coding
ENSG00000038427	VCAN	1.466711263	0.000547947	protein_coding
ENSG00000138336	TET1	1.468057455	0.000720364	protein_coding
ENSG00000144645	OSBPL10	1.469138724	0.001189141	protein_coding
ENSG00000009694	TENM1	1.470691607	0.00082607	protein_coding
ENSG00000144339	TMEFF2	1.472633245	0.043692991	protein_coding
ENSG00000083223	TUT7	1.473471943	0.000715676	protein_coding
ENSG00000143970	ASXL2	1.4738572	0.000563292	protein_coding
ENSG00000145555	MYO10	1.473890608	0.000527703	protein_coding
ENSG00000198948	MFAP3L	1.474402862	0.000924416	protein_coding
ENSG00000132549	VPS13B	1.474879939	0.000625583	protein_coding
ENSG00000139496	NUP58	1.474945977	0.000484452	protein_coding
ENSG00000153207	AHCTF1	1.474950312	0.000497448	protein_coding
ENSG00000005249	PRKAR2B	1.47514382	0.000607829	protein_coding
ENSG00000186472	PCLO	1.475619869	0.001125185	protein_coding
ENSG00000170759	KIF5B	1.476699713	0.000453282	protein_coding
ENSG00000146247	PHIP	1.47697632	0.000498543	protein_coding
ENSG00000072858	SIDT1	1.477222275	0.026682384	protein_coding
ENSG00000162402	USP24	1.479521646	0.00043611	protein_coding
ENSG00000136111	TBC1D4	1.482050673	0.000424901	protein_coding
ENSG00000085224	ATRX	1.482460431	0.000458415	protein_coding
ENSG00000112246	SIM1	1.483002437	0.003585358	protein_coding
ENSG00000184384	MAML2	1.483301032	0.002967311	protein_coding
ENSG00000152601	MBNL1	1.484685649	0.000470307	protein_coding
ENSG00000124198	ARFGEF2	1.486249645	0.000483795	protein_coding
ENSG00000144357	UBR3	1.489042889	0.000514864	protein_coding
ENSG00000198162	MAN1A2	1.49146366	0.000449887	protein_coding
ENSG00000159459	UBR1	1.492695436	0.000590106	protein_coding
ENSG00000174718	KIAA1551	1.492816212	0.000750605	protein_coding
ENSG00000182263	FIGN	1.493163535	0.000809177	protein_coding
ENSG00000160218	TRAPPC10	1.494568698	0.000511676	protein_coding
ENSG00000152104	PTPN14	1.49516858	0.000427819	protein_coding
ENSG00000164506	STXBP5	1.495172462	0.000567517	protein_coding
ENSG00000171488	LRRC8C	1.495655204	0.000592921	protein_coding
ENSG00000115355	CCDC88A	1.496284693	0.000441979	protein_coding

ENSG00000145246	ATP10D	1.497614731	0.001196827	protein_coding
ENSG00000068796	KIF2A	1.498086912	0.000412272	protein_coding
ENSG00000112144	ICK	1.500948431	0.000503528	protein_coding
ENSG00000115760	BIRC6	1.501314148	0.000383074	protein_coding
ENSG00000154556	SORBS2	1.502360967	0.005858889	protein_coding
ENSG00000160551	TAOK1	1.504119281	0.000376958	protein_coding
ENSG00000145715	RASA1	1.504165756	0.000498026	protein_coding
ENSG00000051825	MPHOSPH9	1.50451694	0.0004173	protein_coding
ENSG00000155657	TTN	1.505743027	0.000632275	protein_coding
ENSG00000188906	LRRK2	1.507880476	0.011696955	protein_coding
ENSG00000078687	TNRC6C	1.50796655	0.000517891	protein_coding
ENSG00000178662	CSRNP3	1.508459471	0.001305356	protein_coding
ENSG00000009413	REV3L	1.508509036	0.000438028	protein_coding
ENSG00000122008	POLK	1.508595117	0.000616516	protein_coding
ENSG00000143355	LHX9	1.509196723	0.013488043	protein_coding
ENSG00000122545	7-Sep	1.510231799	0.00035993	protein_coding
ENSG00000197822	OCLN	1.51055612	0.000665918	protein_coding
ENSG00000105971	CAV2	1.510593829	0.000704118	protein_coding
ENSG00000164070	HSPA4L	1.510961314	0.000371008	protein_coding
ENSG00000064393	HIPK2	1.512129909	0.000343472	protein_coding
ENSG00000163959	SLC51A	1.512383522	0.015487289	protein_coding
ENSG00000164008	C1orf50	1.512710189	0.002916468	protein_coding
ENSG00000099204	ABLIM1	1.513884252	0.000346694	protein_coding
ENSG00000102385	DRP2	1.51433776	0.018332243	protein_coding
ENSG00000164300	SERINC5	1.514732025	0.000704118	protein_coding
ENSG00000100503	NIN	1.516174706	0.000336437	protein_coding
ENSG00000116539	ASH1L	1.51795609	0.000354458	protein_coding
ENSG00000102384	CENPI	1.523328938	0.000455588	protein_coding
ENSG00000185483	ROR1	1.523876553	0.000529526	protein_coding
ENSG00000173320	STOX2	1.524453756	0.000533085	protein_coding
ENSG00000053524	MCF2L2	1.526381376	0.026720022	protein_coding
ENSG00000100697	DICER1	1.526740099	0.000300756	protein_coding
ENSG00000055609	KMT2C	1.526973754	0.000324524	protein_coding
ENSG00000100147	CCDC134	1.528624385	0.000932164	protein_coding
ENSG00000163625	WDFY3	1.529090855	0.000368542	protein_coding
ENSG00000181450	ZNF678	1.529464692	0.00036904	protein_coding
ENSG00000128512	DOCK4	1.529935088	0.000494434	protein_coding
ENSG00000138246	DNAJC13	1.530608447	0.000328102	protein_coding
ENSG00000005100	DHX33	1.531905824	0.000284731	protein_coding
ENSG00000171444	MCC	1.532198613	0.000339791	protein_coding
ENSG00000196584	XRCC2	1.533421851	0.000293343	protein_coding
ENSG00000169330	KIAA1024	1.535378704	0.000903621	protein_coding
ENSG00000198932	GPRASP1	1.537388931	0.044489089	protein_coding

ENSG00000101752	MIB1	1.538507803	0.000270912	protein_coding
ENSG00000160352	ZNF714	1.539256826	0.000339808	protein_coding
ENSG00000143858	SYT2	1.540152201	0.000828899	protein_coding
ENSG00000128965	CHAC1	1.544735755	0.000676564	protein_coding
ENSG00000146072	TNFRSF21	1.544876866	0.000366539	protein_coding
ENSG00000241697	TMEFF1	1.545407546	0.001553639	protein_coding
ENSG00000152527	PLEKHH2	1.546915204	0.000802826	protein_coding
ENSG00000197603	CPLANE1	1.547064699	0.000466596	protein_coding
ENSG00000165288	BRWD3	1.548170364	0.000313688	protein_coding
ENSG00000162601	MYSM1	1.548840839	0.000258015	protein_coding
ENSG00000102780	DGKH	1.548868142	0.00034466	protein_coding
ENSG00000006459	KDM7A	1.55092682	0.000308933	protein_coding
ENSG00000117322	CR2	1.551790528	0.003276705	protein_coding
ENSG00000118412	CASP8AP2	1.554386168	0.000291275	protein_coding
ENSG00000171988	JMJD1C	1.555443752	0.000335561	protein_coding
ENSG00000137962	ARHGAP29	1.55562013	0.000323269	protein_coding
ENSG00000125107	CNOT1	1.556291359	0.000215487	protein_coding
ENSG00000170500	LONRF2	1.556880596	0.000387151	protein_coding
ENSG00000174485	DENND4A	1.558440987	0.000332434	protein_coding
ENSG00000151693	ASAP2	1.559963117	0.000464187	protein_coding
ENSG00000066933	MYO9A	1.56010281	0.000276046	protein_coding
ENSG00000115020	PIKFYVE	1.561117654	0.000264946	protein_coding
ENSG00000163590	PPM1L	1.564347096	0.000287948	protein_coding
ENSG00000104164	BLOC1S6	1.566470141	0.000267991	protein_coding
ENSG00000169860	P2RY1	1.567154164	0.000301637	protein_coding
ENSG00000123612	ACVR1C	1.568105443	0.017143019	protein_coding
ENSG00000101265	RASSF2	1.568429952	0.000697959	protein_coding
ENSG00000284906	AC091057.6	1.571692592	0.00313105	protein_coding
ENSG00000176563	CNTD1	1.57677882	0.021096335	protein_coding
ENSG00000113594	LIFR	1.577345315	0.000247113	protein_coding
ENSG00000147041	SYTL5	1.577822614	0.000683479	protein_coding
ENSG00000197077	KIAA1671	1.578114965	0.000215822	protein_coding
ENSG00000133657	ATP13A3	1.582815864	0.000187501	protein_coding
ENSG00000171385	KCND3	1.584505776	0.012652254	protein_coding
ENSG00000139116	KIF21A	1.584753645	0.000230013	protein_coding
ENSG00000169570	DTWD2	1.585244496	0.000411463	protein_coding
ENSG00000180875	GREM2	1.587515822	0.026497687	protein_coding
ENSG00000111859	NEDD9	1.587515822	0.026497687	protein_coding
ENSG00000119778	ATAD2B	1.588011266	0.00026291	protein_coding
ENSG00000075213	SEMA3A	1.589108885	0.001514254	protein_coding
ENSG00000165240	ATP7A	1.589462321	0.000336573	protein_coding
ENSG00000140993	TIGD7	1.591146216	0.004127818	protein_coding
ENSG00000204577	LILRB3	1.592875369	0.002983684	protein_coding

ENSG00000136014	USP44	1.593146999	0.000860906	protein_coding
ENSG00000081026	MAGI3	1.59508587	0.000203674	protein_coding
ENSG00000073910	FRY	1.595196709	0.000882878	protein_coding
ENSG00000135837	CEP350	1.595561605	0.000184324	protein_coding
ENSG00000100605	ITPK1	1.600208148	0.000175365	protein_coding
ENSG00000069667	RORA	1.60084916	0.000376629	protein_coding
ENSG00000166736	HTR3A	1.60115282	0.034088382	protein_coding
ENSG00000070018	LRP6	1.601160991	0.00018209	protein_coding
ENSG00000172348	RCAN2	1.601756527	0.002312478	protein_coding
ENSG00000065413	ANKRD44	1.601847735	0.000956767	protein_coding
ENSG00000116675	DNAJC6	1.601951112	0.000200597	protein_coding
ENSG00000157077	ZFYVE9	1.610490067	0.000199798	protein_coding
ENSG00000128585	MKLN1	1.613564807	0.000175186	protein_coding
ENSG00000183808	RBM12B	1.614344008	0.000175273	protein_coding
ENSG00000100490	CDKL1	1.617973875	0.001303686	protein_coding
ENSG00000115423	DNAH6	1.61835016	0.010511842	protein_coding
ENSG00000140873	ADAMTS18	1.619049114	0.045117465	protein_coding
ENSG00000117281	CD160	1.619049114	0.045117465	protein_coding
ENSG00000148488	ST8SIA6	1.619049114	0.045117465	protein_coding
ENSG00000129204	USP6	1.619049114	0.045117465	protein_coding
ENSG00000148773	MKI67	1.620178399	0.000121026	protein_coding
ENSG00000261115	TMEM178B	1.620795311	0.000209648	protein_coding
ENSG00000260097	SPDYE6	1.622120824	0.006984897	protein_coding
ENSG00000054654	SYNE2	1.622148252	0.000150131	protein_coding
ENSG00000206538	VGLL3	1.623341956	0.000846283	protein_coding
ENSG00000011405	PIK3C2A	1.624680205	0.00013574	protein_coding
ENSG00000176542	USF3	1.628449648	0.000186887	protein_coding
ENSG00000103489	XYLT1	1.630072381	0.000257275	protein_coding
ENSG00000088305	DNMT3B	1.632640001	0.000138126	protein_coding
ENSG00000109756	RAPGEF2	1.636713555	0.000143959	protein_coding
ENSG00000037749	MFAP3	1.637047203	0.000177332	protein_coding
ENSG00000176148	TCP11L1	1.63753662	0.000229425	protein_coding
ENSG00000109265	KIAA1211	1.638705317	0.000242381	protein_coding
ENSG00000160145	KALRN	1.639049613	0.000711031	protein_coding
ENSG00000104093	DMXL2	1.64034536	0.000154523	protein_coding
ENSG00000162989	KCNJ3	1.640836162	0.009270117	protein_coding
ENSG00000066405	CLDN18	1.641699242	0.020623756	protein_coding
ENSG00000082293	COL19A1	1.641699242	0.020623756	protein_coding
ENSG00000152402	GUCY1A2	1.643077755	0.000881171	protein_coding
ENSG00000111727	HCFC2	1.643246968	0.00038367	protein_coding
ENSG00000113396	SLC27A6	1.647376498	0.003723589	protein_coding
ENSG00000240891	PLCXD2	1.648836976	0.000446563	protein_coding
ENSG00000170542	SERPINB9	1.649022868	0.005927856	protein_coding

ENSG00000137642	SORL1	1.64978141	0.000184284	protein_coding
ENSG00000092853	CLSPN	1.650082848	0.00011775	protein_coding
ENSG00000136237	RAPGEF5	1.651115671	0.00025224	protein_coding
ENSG00000164330	EBF1	1.651418749	0.008734897	protein_coding
ENSG00000109819	PPARGC1A	1.651418749	0.008734897	protein_coding
ENSG00000113742	CPEB4	1.651542118	0.000179574	protein_coding
ENSG00000117500	TMED5	1.654776731	9.78E-05	protein_coding
ENSG00000188152	NUTM2G	1.65575335	0.004091611	protein_coding
ENSG00000072736	NFATC3	1.655792559	0.000128208	protein_coding
ENSG00000242689	CNTF	1.659152604	0.006686163	protein_coding
ENSG00000152818	UTRN	1.659693577	0.000106424	protein_coding
ENSG00000169314	C22orf15	1.662217371	0.026105331	protein_coding
ENSG00000180071	ANKRD18A	1.663548779	0.000394533	protein_coding
ENSG00000186469	GNG2	1.664419208	0.000639909	protein_coding
ENSG00000116127	ALMS1	1.674599051	0.000105951	protein_coding
ENSG00000142396	ERVK3-1	1.67649588	0.000334703	protein_coding
ENSG00000124942	AHNAK	1.677722972	8.13E-05	protein_coding
ENSG00000065060	UHRF1BP1	1.680383083	8.62E-05	protein_coding
ENSG00000157404	KIT	1.681794336	0.000119576	protein_coding
ENSG00000120800	UTP20	1.681877796	7.82E-05	protein_coding
ENSG00000048052	HDAC9	1.682145192	0.002565378	protein_coding
ENSG00000162885	B3GALNT2	1.683633572	0.000106381	protein_coding
ENSG00000205238	SPDYE2	1.683746314	0.007259967	protein_coding
ENSG00000196912	ANKRD36B	1.685253921	0.000443499	protein_coding
ENSG00000164171	ITGA2	1.688662886	0.000199232	protein_coding
ENSG00000173567	ADGRF3	1.688997205	0.033910222	protein_coding
ENSG00000133019	CHRM3	1.688997205	0.033910222	protein_coding
ENSG00000176771	NCKAP5	1.689659304	0.003863477	protein_coding
ENSG00000149256	TENM4	1.693340472	0.000180889	protein_coding
ENSG00000178033	CALHM5	1.693921139	0.016051556	protein_coding
ENSG00000174501	ANKRD36C	1.695887206	0.0003108	protein_coding
ENSG00000152377	SPOCK1	1.69689086	0.008764824	protein_coding
ENSG00000112159	MDN1	1.698847211	5.99E-05	protein_coding
ENSG00000184588	PDE4B	1.698877121	0.008356463	protein_coding
ENSG00000150893	FREM2	1.703059914	0.000135322	protein_coding
ENSG00000114541	FRMD4B	1.70414796	0.000255176	protein_coding
ENSG00000172733	PURG	1.704867089	0.000826852	protein_coding
ENSG00000140836	ZFHX3	1.706499866	0.00011899	protein_coding
ENSG00000145675	PIK3R1	1.708158629	0.000101321	protein_coding
ENSG00000066279	ASPM	1.714529846	5.26E-05	protein_coding
ENSG00000108021	FAM208B	1.7165249	5.10E-05	protein_coding
ENSG00000158427	TMSB15B	1.718197502	0.010384446	protein_coding
ENSG00000137393	RNF144B	1.721995339	0.001490418	protein_coding

ENSG00000150627	WDR17	1.722449857	0.000119358	protein_coding
ENSG00000153707	PTPRD	1.725045925	8.52E-05	protein_coding
ENSG00000136040	PLXNC1	1.728552161	0.001127459	protein_coding
ENSG00000145734	BDP1	1.730600264	5.09E-05	protein_coding
ENSG00000180263	FGD6	1.730736588	0.000136106	protein_coding
ENSG00000162849	KIF26B	1.734513932	0.000286059	protein_coding
ENSG00000164187	LMBRD2	1.736457661	0.00010159	protein_coding
ENSG00000197969	VPS13A	1.737418406	4.57E-05	protein_coding
ENSG00000197312	DDI2	1.738304609	4.58E-05	protein_coding
ENSG00000104517	UBR5	1.742879689	4.03E-05	protein_coding
ENSG00000156675	RAB11FIP1	1.744321703	5.44E-05	protein_coding
ENSG00000141367	CLTC	1.745486264	3.70E-05	protein_coding
ENSG00000187801	ZFP69B	1.747700817	0.001240795	protein_coding
ENSG00000166446	CDYL2	1.750101932	0.000159047	protein_coding
ENSG00000131711	MAP1B	1.750780864	4.59E-05	protein_coding
ENSG00000138688	KIAA1109	1.755052342	5.93E-05	protein_coding
ENSG00000136197	C7orf25	1.755710159	0.025475297	protein_coding
ENSG00000087494	PTHLH	1.755710159	0.025475297	protein_coding
ENSG00000121101	TEX14	1.755710159	0.025475297	protein_coding
ENSG00000180530	NRIP1	1.756846255	6.88E-05	protein_coding
ENSG00000038295	TLL1	1.762415722	0.008204261	protein_coding
ENSG00000081479	LRP2	1.762987354	5.20E-05	protein_coding
ENSG00000110344	UBE4A	1.777797759	3.83E-05	protein_coding
ENSG00000130783	CCDC62	1.777899623	0.045202178	protein_coding
ENSG00000176928	GCNT4	1.777899623	0.045202178	protein_coding
ENSG00000203814	HIST2H2BF	1.777899623	0.045202178	protein_coding
ENSG00000137261	KIAA0319	1.777899623	0.045202178	protein_coding
ENSG00000188064	WNT7B	1.777899623	0.045202178	protein_coding
ENSG00000145794	MEGF10	1.785809915	0.00374952	protein_coding
ENSG00000198780	FAM169A	1.789625708	6.15E-05	protein_coding
ENSG00000261150	EPPK1	1.791082152	0.000306115	protein_coding
ENSG00000119547	ONECUT2	1.792830264	3.25E-05	protein_coding
ENSG00000168769	TET2	1.796930299	4.59E-05	protein_coding
ENSG00000070669	ASNS	1.797253478	0.000251613	protein_coding
ENSG00000057468	MSH4	1.802890026	0.033376343	protein_coding
ENSG00000119121	TRPM6	1.802890026	0.033376343	protein_coding
ENSG00000064309	CDON	1.803896811	3.54E-05	protein_coding
ENSG00000156011	PSD3	1.803928892	3.40E-05	protein_coding
ENSG00000074410	CA12	1.813447702	4.82E-05	protein_coding
ENSG00000162490	DRAXIN	1.819474048	0.019137366	protein_coding
ENSG00000103534	TMC5	1.819474048	0.019137366	protein_coding
ENSG00000230453	ANKRD18B	1.820314568	9.13E-05	protein_coding
ENSG00000134532	SOX5	1.821627016	0.000256125	protein_coding

ENSG00000123104	ITPR2	1.826962631	2.84E-05	protein_coding
ENSG00000138119	MYOF	1.829736683	0.003870034	protein_coding
ENSG00000113810	SMC4	1.830974398	1.66E-05	protein_coding
ENSG00000156218	ADAMTSL3	1.835336115	0.003476403	protein_coding
ENSG00000177483	RBM44	1.8401208	0.007582043	protein_coding
ENSG00000258881	AC007040.2	1.840125196	0.002638872	protein_coding
ENSG00000173517	PEAK1	1.845405353	1.92E-05	protein_coding
ENSG00000139209	SLC38A4	1.84698292	0.00512978	protein_coding
ENSG00000140937	CDH11	1.847887059	0.001614421	protein_coding
ENSG00000176244	ACBD7	1.852558997	0.000433815	protein_coding
ENSG00000107249	GLIS3	1.864339988	0.000511591	protein_coding
ENSG00000253729	PRKDC	1.865211579	1.09E-05	protein_coding
ENSG00000119285	HEATR1	1.865747931	1.14E-05	protein_coding
ENSG00000204524	ZNF805	1.868660561	0.000338464	protein_coding
ENSG00000284862	CCDC39	1.870238424	0.032292239	protein_coding
ENSG00000090339	ICAM1	1.870238424	0.032292239	protein_coding
ENSG00000182400	TRAPPC6B	1.873204296	0.000121634	protein_coding
ENSG00000151835	SACS	1.875909836	1.19E-05	protein_coding
ENSG00000188158	NHS	1.876694399	8.97E-05	protein_coding
ENSG00000005810	MYCBP2	1.8770259	1.05E-05	protein_coding
ENSG00000113522	RAD50	1.877836135	0.000147997	protein_coding
ENSG00000119737	GPR75	1.879289672	0.000524355	protein_coding
ENSG00000145826	LECT2	1.880538599	0.014380224	protein_coding
ENSG00000127329	PTPRB	1.883505264	0.00898543	protein_coding
ENSG00000171724	VAT1L	1.883505264	0.00898543	protein_coding
ENSG00000196660	SLC30A10	1.890015129	0.002125384	protein_coding
ENSG00000103888	CEMIP	1.89096877	0.002641227	protein_coding
ENSG00000165323	FAT3	1.903268712	1.92E-05	protein_coding
ENSG00000177200	CHD9	1.905022104	1.17E-05	protein_coding
ENSG00000173166	RAPH1	1.909250224	0.000174393	protein_coding
ENSG00000145242	EPHA5	1.911578956	0.000351831	protein_coding
ENSG00000182901	RGS7	1.924382764	0.002322339	protein_coding
ENSG00000143341	HMCN1	1.930744995	0.000481604	protein_coding
ENSG00000107890	ANKRD26	1.94048301	1.98E-05	protein_coding
ENSG00000182389	CACNB4	1.944851693	1.32E-05	protein_coding
ENSG00000242265	PEG10	1.946351172	0.018003167	protein_coding
ENSG00000097096	SYDE2	1.948220768	1.81E-05	protein_coding
ENSG00000198963	RORB	1.952670797	7.86E-05	protein_coding
ENSG00000165813	CCDC186	1.959398793	1.18E-05	protein_coding
ENSG00000198729	PPP1R14C	1.962666637	9.20E-06	protein_coding
ENSG00000156869	FRRS1	1.96556378	6.07E-05	protein_coding
ENSG00000146858	ZC3HAV1L	1.966993866	9.73E-06	protein_coding
ENSG00000241484	ARHGAP8	1.968682577	6.30E-05	protein_coding

ENSG00000000005	TNMD	1.972858635	0.00360343	protein_coding
ENSG00000001626	CFTR	1.974359603	0.043991311	protein_coding
ENSG00000073464	CLCN4	1.974359603	0.043991311	protein_coding
ENSG00000206384	COL6A6	1.974359603	0.043991311	protein_coding
ENSG00000173258	ZNF483	1.987027818	0.000189959	protein_coding
ENSG00000160401	CFAP157	1.997639638	0.000150461	protein_coding
ENSG00000177076	ACER2	2.006147899	0.001496038	protein_coding
ENSG00000196159	FAT4	2.013227867	1.48E-05	protein_coding
ENSG00000133863	TEX15	2.014496886	4.10E-06	protein_coding
ENSG00000185477	GPRIN3	2.016214427	5.15E-06	protein_coding
ENSG00000131016	AKAP12	2.019819803	2.52E-06	protein_coding
ENSG00000188848	BEND4	2.021710131	5.70E-06	protein_coding
ENSG00000255423	EBLN2	2.023913006	0.000977512	protein_coding
ENSG00000166004	CEP295	2.024453143	5.88E-06	protein_coding
ENSG00000155629	PIK3AP1	2.043914303	3.38E-05	protein_coding
ENSG00000189184	PCDH18	2.052036269	3.13E-05	protein_coding
ENSG00000182986	ZNF320	2.055017197	0.002205393	protein_coding
ENSG00000112379	ARFGEF3	2.066732585	5.54E-06	protein_coding
ENSG00000157106	SMG1	2.069643947	1.38E-06	protein_coding
ENSG00000020577	SAMD4A	2.070436178	3.66E-06	protein_coding
ENSG00000134317	GRHL1	2.076376212	2.92E-05	protein_coding
ENSG00000099260	PALMD	2.076828015	0.009732694	protein_coding
ENSG00000163377	FAM19A4	2.080259875	0.030348148	protein_coding
ENSG00000198146	ZNF770	2.09297018	1.66E-06	protein_coding
ENSG00000004799	PDK4	2.101826493	0.004621058	protein_coding
ENSG00000102908	NFAT5	2.10674693	1.58E-06	protein_coding
ENSG00000165194	PCDH19	2.110641212	1.02E-05	protein_coding
ENSG00000166126	AMN	2.116339181	0.016492881	protein_coding
ENSG00000137571	SLCO5A1	2.118142393	1.53E-05	protein_coding
ENSG00000138769	CDKL2	2.130372933	0.000890608	protein_coding
ENSG00000169432	SCN9A	2.138437934	3.68E-05	protein_coding
ENSG00000172292	CERS6	2.138760603	1.19E-06	protein_coding
ENSG00000180801	ARSJ	2.14666337	6.76E-05	protein_coding
ENSG00000267216	AC020915.1	2.176045253	0.00064876	protein_coding
ENSG00000234284	ZNF879	2.178477119	0.00013211	protein_coding
ENSG00000148204	CRB2	2.178915363	0.020952111	protein_coding
ENSG00000028137	TNFRSF1B	2.178915363	0.020952111	protein_coding
ENSG00000133124	IRS4	2.18959203	3.34E-07	protein_coding
ENSG00000106278	PTPRZ1	2.189852236	0.011803031	protein_coding
ENSG00000111913	RIPOR2	2.189852236	0.011803031	protein_coding
ENSG00000115464	USP34	2.190390421	4.43E-07	protein_coding
ENSG00000115232	ITGA4	2.190633679	1.41E-06	protein_coding
ENSG00000112137	PHACTR1	2.193012989	0.000204205	protein_coding

ENSG00000120500	ARR3	2.196477074	0.005286278	protein_coding
ENSG00000169856	ONECUT1	2.196477074	0.005286278	protein_coding
ENSG00000169403	PTAFR	2.196477074	0.005286278	protein_coding
ENSG00000159905	ZNF221	2.204106808	0.001436097	protein_coding
ENSG00000046889	PREX2	2.215859245	2.61E-06	protein_coding
ENSG00000151012	SLC7A11	2.218139691	4.49E-07	protein_coding
ENSG00000173678	SPDYE2B	2.248026154	0.001998931	protein_coding
ENSG00000189195	BTBD8	2.266310999	0.000273264	protein_coding
ENSG00000023445	BIRC3	2.271254164	0.014485426	protein_coding
ENSG00000186047	DLEU7	2.271254164	0.014485426	protein_coding
ENSG00000179331	RAB39A	2.283506676	3.36E-06	protein_coding
ENSG00000132561	MATN2	2.283990175	0.000860935	protein_coding
ENSG00000164142	FAM160A1	2.285009301	3.62E-06	protein_coding
ENSG00000165376	CLDN2	2.293253063	0.041032031	protein_coding
ENSG00000255154	HTD2	2.293253063	0.041032031	protein_coding
ENSG00000142512	SIGLEC10	2.293253063	0.041032031	protein_coding
ENSG00000110436	SLC1A2	2.293253063	0.041032031	protein_coding
ENSG00000183638	RP1L1	2.293642619	0.001516645	protein_coding
ENSG00000197837	HIST4H4	2.306958564	0.00288962	protein_coding
ENSG00000188167	TMPPE	2.318718056	2.18E-05	protein_coding
ENSG00000197779	ZNF81	2.324104073	5.78E-07	protein_coding
ENSG00000152580	IGSF10	2.326513282	0.006072876	protein_coding
ENSG00000092969	TGFB2	2.326513282	0.006072876	protein_coding
ENSG00000139973	SYT16	2.34502156	2.80E-05	protein_coding
ENSG00000146414	SHPRH	2.35299151	1.08E-07	protein_coding
ENSG00000179902	C1orf194	2.358036719	0.010033723	protein_coding
ENSG00000131730	CKMT2	2.359681431	0.000519431	protein_coding
ENSG00000188033	ZNF490	2.359681431	0.000519431	protein_coding
ENSG00000144893	MED12L	2.374339114	7.67E-07	protein_coding
ENSG00000233757	AC092835.1	2.396088153	0.000404469	protein_coding
ENSG00000149970	CNKSR2	2.417386885	0.02704271	protein_coding
ENSG00000204882	GPR20	2.417386885	0.02704271	protein_coding
ENSG00000164532	TBX20	2.431848671	1.69E-05	protein_coding
ENSG00000109906	ZBTB16	2.434070994	5.74E-05	protein_coding
ENSG00000280987	MATR3	2.439893911	0.010033723	protein_coding
ENSG00000106125	MINDY4	2.439893911	0.010033723	protein_coding
ENSG00000104321	TRPA1	2.439893911	0.010033723	protein_coding
ENSG00000135913	USP37	2.450374023	7.17E-08	protein_coding
ENSG00000008311	AASS	2.450469332	9.42E-08	protein_coding
ENSG00000171189	GRIK1	2.451341722	0.003149424	protein_coding
ENSG00000033122	LRRC7	2.451585365	7.64E-05	protein_coding
ENSG00000100852	ARHGAP5	2.485741296	2.43E-08	protein_coding
ENSG00000140009	ESR2	2.495221266	5.31E-05	protein_coding

ENSG00000166439	RNF169	2.496854103	2.59E-08	protein_coding
ENSG00000163635	ATXN7	2.505380121	0.000883969	protein_coding
ENSG00000174130	TLR6	2.509926229	0.002275868	protein_coding
ENSG00000182108	DEXI	2.515317833	7.76E-07	protein_coding
ENSG00000053328	METTL24	2.51735492	0.006966163	protein_coding
ENSG00000160229	ZNF66	2.531680917	0.017862218	protein_coding
ENSG00000197714	ZNF460	2.53640587	6.32E-06	protein_coding
ENSG00000133816	MICAL2	2.542918878	2.45E-07	protein_coding
ENSG00000172575	RASGRP1	2.549679762	2.08E-06	protein_coding
ENSG00000135436	FAM186B	2.566224299	0.001648699	protein_coding
ENSG00000249931	GOLGA8K	2.566224299	0.001648699	protein_coding
ENSG00000185900	POMK	2.575406271	5.31E-08	protein_coding
ENSG00000137801	THBS1	2.590867976	0.004849114	protein_coding
ENSG00000198785	GRIN3A	2.620407719	0.001197455	protein_coding
ENSG00000218305	CDC14C	2.637581188	0.011832427	protein_coding
ENSG00000157999	ANKRD61	2.64991994	0.000137515	protein_coding
ENSG00000122420	PTGFR	2.660816067	0.003385132	protein_coding
ENSG00000261732	AL031708.1	2.727529021	0.002370371	protein_coding
ENSG00000155011	DKK2	2.736236676	0.007864773	protein_coding
ENSG00000151322	NPAS3	2.736236676	0.007864773	protein_coding
ENSG00000248405	PRR5-ARHGAP8	2.736236676	0.007864773	protein_coding
ENSG00000144426	NBEAL1	2.75269007	5.06E-08	protein_coding
ENSG00000137959	IFI44L	2.771723241	0.000466301	protein_coding
ENSG00000188211	NCR3LG1	2.789086287	4.75E-10	protein_coding
ENSG00000168454	TXNDC2	2.79129291	0.001665133	protein_coding
ENSG00000080200	CRYBG3	2.810448053	7.49E-10	protein_coding
ENSG00000175556	LONRF3	2.827566775	1.97E-09	protein_coding
ENSG00000187987	ZSCAN23	2.864752801	2.96E-06	protein_coding
ENSG00000134042	MRO	2.910941968	0.000829981	protein_coding
ENSG00000136206	SPDYE1	2.915358032	0.003515158	protein_coding
ENSG00000283208	AC001226.2	2.927583998	0.034866602	protein_coding
ENSG00000175820	CCDC168	2.927583998	0.034866602	protein_coding
ENSG00000176153	GPX2	2.927583998	0.034866602	protein_coding
ENSG00000148082	SHC3	2.927583998	0.034866602	protein_coding
ENSG00000162753	SLC9C2	2.927583998	0.034866602	protein_coding
ENSG00000177301	KCNA2	2.930698097	7.50E-06	protein_coding
ENSG00000205413	SAMD9	2.967240038	0.000589	protein_coding
ENSG00000151967	SCHIP1	2.993231159	0.000102431	protein_coding
ENSG00000133107	TRPC4	2.997215225	0.003515158	protein_coding
ENSG00000185666	SYN3	3.008844338	2.42E-05	protein_coding
ENSG00000165521	EML5	3.020040061	7.50E-10	protein_coding
ENSG00000041353	RAB27B	3.073645355	0.000299755	protein_coding

ENSG00000129048	ACKR4	3.074676234	0.002364823	protein_coding
ENSG00000169435	RASSF6	3.074676234	0.002364823	protein_coding
ENSG00000165972	CCDC38	3.077542886	0.021566548	protein_coding
ENSG00000172404	DNAJB7	3.077542886	0.021566548	protein_coding
ENSG00000182732	RGS6	3.077542886	0.021566548	protein_coding
ENSG00000112212	TSPO2	3.077542886	0.021566548	protein_coding
ENSG00000178502	KLHL11	3.180205542	1.51E-10	protein_coding
ENSG00000283761	AC118553.2	3.21337172	0.013412089	protein_coding
ENSG00000167601	AXL	3.21337172	0.013412089	protein_coding
ENSG00000165168	CYBB	3.21337172	0.013412089	protein_coding
ENSG00000122735	DNAI1	3.21337172	0.013412089	protein_coding
ENSG00000184599	FAM19A3	3.21337172	0.013412089	protein_coding
ENSG00000182329	KIAA2012	3.21337172	0.013412089	protein_coding
ENSG00000262180	OCLM	3.21337172	0.013412089	protein_coding
ENSG00000114757	PEX5L	3.21337172	0.013412089	protein_coding
ENSG00000158683	PKD1L1	3.21337172	0.013412089	protein_coding
ENSG00000165646	SLC18A2	3.21337172	0.013412089	protein_coding
ENSG00000259224	SLC35G6	3.21337172	0.013412089	protein_coding
ENSG00000105989	WNT2	3.21337172	0.013412089	protein_coding
ENSG00000131379	C3orf20	3.218137381	0.001084588	protein_coding
ENSG00000173157	ADAMTS20	3.308352831	9.30E-08	protein_coding
ENSG00000265107	GJA5	3.337505542	0.008390282	protein_coding
ENSG00000188921	HACD4	3.337505542	0.008390282	protein_coding
ENSG00000174145	NWD2	3.337505542	0.008390282	protein_coding
ENSG00000101349	PAK5	3.337505542	0.008390282	protein_coding
ENSG00000162924	REL	3.345425804	8.37E-12	protein_coding
ENSG00000171217	CLDN20	3.451799574	0.005281544	protein_coding
ENSG00000204767	INSYN2B	3.451799574	0.005281544	protein_coding
ENSG00000166800	LDHAL6A	3.451799574	0.005281544	protein_coding
ENSG00000080854	IGSF9B	3.490554174	4.86E-11	protein_coding
ENSG00000281106	TMEM272	3.524561352	0.000167283	protein_coding
ENSG00000196811	CHRNG	3.557699845	0.003346055	protein_coding
ENSG00000108950	FAM20A	3.557699845	0.003346055	protein_coding
ENSG00000164197	RNF180	3.656355333	0.002133716	protein_coding
ENSG00000104728	ARHGEF10	3.656396259	3.89E-06	protein_coding
ENSG00000157654	PALM2-AKAP2	3.748694134	0.001369576	protein_coding
ENSG00000187243	MAGED4B	3.758713601	1.10E-11	protein_coding
ENSG00000132357	CARD6	3.818196698	9.57E-07	protein_coding
ENSG00000166960	CCDC178	3.83547669	0.000884859	protein_coding
ENSG00000103184	SEC14L5	3.83547669	0.000884859	protein_coding
ENSG00000243444	PALM2	3.994794891	0.000575412	protein_coding
ENSG00000267710	EDDM13	4.204968992	0.000164371	protein_coding

ENSG00000188107	EYS	4.204968992	0.000164371	protein_coding
ENSG00000197410	DCHS2	4.329797432	7.35E-05	protein_coding
ENSG00000261247	GOLGA8T	4.650178951	7.53E-06	protein_coding
ENSG00000173208	ABCD2	5.567319661	0.040964585	protein_coding
ENSG00000281593	AC006978.2	5.567319661	0.040964585	protein_coding
ENSG00000273291	AC092042.3	5.567319661	0.040964585	protein_coding
ENSG00000258466	AL049779.1	5.567319661	0.040964585	protein_coding
ENSG00000164122	ASB5	5.567319661	0.040964585	protein_coding
ENSG00000152430	BOLL	5.567319661	0.040964585	protein_coding
ENSG00000181215	C4orf50	5.567319661	0.040964585	protein_coding
ENSG00000123500	COL10A1	5.567319661	0.040964585	protein_coding
ENSG00000124875	CXCL6	5.567319661	0.040964585	protein_coding
ENSG00000173198	CYSLTR1	5.567319661	0.040964585	protein_coding
ENSG00000124721	DNAH8	5.567319661	0.040964585	protein_coding
ENSG00000170074	FAM153A	5.567319661	0.040964585	protein_coding
ENSG00000164125	FAM198B	5.567319661	0.040964585	protein_coding
ENSG00000115474	KCNJ13	5.567319661	0.040964585	protein_coding
ENSG00000184261	KCNK12	5.567319661	0.040964585	protein_coding
ENSG00000226650	KIF4B	5.567319661	0.040964585	protein_coding
ENSG00000101977	MCF2	5.567319661	0.040964585	protein_coding
ENSG00000162992	NEUROD1	5.567319661	0.040964585	protein_coding
ENSG00000132915	PDE6A	5.567319661	0.040964585	protein_coding
ENSG00000095303	PTGS1	5.567319661	0.040964585	protein_coding
ENSG00000188050	RNF133	5.567319661	0.040964585	protein_coding
ENSG00000106327	TFR2	5.567319661	0.040964585	protein_coding
ENSG00000234224	TMEM229A	5.567319661	0.040964585	protein_coding
ENSG00000049249	TNFRSF9	5.567319661	0.040964585	protein_coding
ENSG00000106927	AMBP	5.785358955	0.022676824	protein_coding
ENSG00000091879	ANGPT2	5.785358955	0.022676824	protein_coding
ENSG00000164287	CDC20B	5.785358955	0.022676824	protein_coding
ENSG00000104432	IL7	5.785358955	0.022676824	protein_coding
ENSG00000184361	SPATA32	5.785358955	0.022676824	protein_coding
ENSG00000116194	ANGPTL1	5.974730546	0.012730848	protein_coding
ENSG00000232196	MTRNR2L4	5.974730546	0.012730848	protein_coding
ENSG00000154645	CHODL	6.142104357	0.007243414	protein_coding
ENSG00000143226	FCGR2A	6.142104357	0.007243414	protein_coding
ENSG00000153253	SCN3A	6.142104357	0.007243414	protein_coding
ENSG00000182584	ACTL10	6.292063245	0.004174171	protein_coding
ENSG00000124701	APOBEC2	6.292063245	0.004174171	protein_coding
ENSG00000205882	DEFB134	6.292063245	0.004174171	protein_coding
ENSG00000263874	LINC00672	6.292063245	0.004174171	protein_coding
ENSG00000115956	PLEK	6.292063245	0.004174171	protein_coding
ENSG00000151062	CACNA2D4	6.42789208	0.002434933	protein_coding

ENSG00000135960	EDAR	6.42789208	0.002434933	protein_coding
ENSG00000019991	HGF	6.42789208	0.002434933	protein_coding
ENSG00000056291	NPFFR2	6.42789208	0.002434933	protein_coding
ENSG00000102359	SRPX2	6.42789208	0.002434933	protein_coding
ENSG00000276203	ANKRD20A3	6.552025902	0.00143701	protein_coding
ENSG00000127362	TAS2R3	6.552025902	0.00143701	protein_coding
ENSG00000257315	ZBED6	6.552025902	0.00143701	protein_coding
ENSG00000138379	MSTN	6.666319933	0.00085757	protein_coding
ENSG00000113494	PRLR	6.666319933	0.00085757	protein_coding
ENSG00000055813	CCDC85A	6.772220205	0.000517265	protein_coding
ENSG00000204323	SMIM5	6.870875693	0.000315208	protein_coding
ENSG00000242950	ERVW-1	6.963214494	0.000193974	protein_coding
ENSG00000120215	MLANA	7.765605685	1.61E-06	protein_coding

Genes differentially expressed in shBLM cells (p<0.05; log2FoldChang>1)				
**Gene_id**	**Gene_name**	**log2FoldChange**	**P value**	**Gene_biotype**
ENSG00000162032	SPSB3	-7.339690146	3.05E-05	protein_coding
ENSG00000130489	SCO2	-7.188676326	7.55E-05	protein_coding
ENSG00000175832	ETV4	-7.019986963	0.000193974	protein_coding
ENSG00000132518	GUCY2D	-6.72298963	0.00085757	protein_coding
ENSG00000254585	MAGEL2	-6.72298963	0.00085757	protein_coding
ENSG00000100060	MFNG	-6.608650041	0.00143701	protein_coding
ENSG00000273154	AL121845.3	-6.484462474	0.002434933	protein_coding
ENSG00000171847	FAM90A1	-6.484462474	0.002434933	protein_coding
ENSG00000164855	TMEM184A	-6.484462474	0.002434933	protein_coding
ENSG00000261408	TEN1-CDK3	-6.348569279	0.004174171	protein_coding
ENSG00000169744	LDB2	-6.198531926	0.007243414	protein_coding
ENSG00000259305	ZHX1-C8orf76	-6.198531926	0.007243414	protein_coding
ENSG00000224712	NPIPA3	-6.198531926	0.007243414	protein_coding
ENSG00000102452	NALCN	-6.198531926	0.007243414	protein_coding
ENSG00000132274	TRIM22	-6.198531926	0.007243414	protein_coding
ENSG00000006047	YBX2	-6.031060341	0.012730848	protein_coding
ENSG00000114547	ROPN1B	-6.031060341	0.012730848	protein_coding
ENSG00000198844	ARHGEF15	-6.031060341	0.012730848	protein_coding
ENSG00000010704	HFE	-5.841563537	0.022676824	protein_coding
ENSG00000169181	GSG1L	-5.841563537	0.022676824	protein_coding
ENSG00000158816	VWA5B1	-5.841563537	0.022676824	protein_coding
ENSG00000109758	HGFAC	-5.623358155	0.040964585	protein_coding
ENSG00000132026	RTBDN	-5.623358155	0.040964585	protein_coding
ENSG00000182584	ACTL10	-5.623358155	0.040964585	protein_coding
ENSG00000112245	PTP4A1	-5.623358155	0.040964585	protein_coding
ENSG00000100985	MMP9	-5.623358155	0.040964585	protein_coding
ENSG00000197479	PCDHB11	-5.623358155	0.040964585	protein_coding
ENSG00000102931	ARL2BP	-5.304971107	5.77E-15	protein_coding
ENSG00000154545	MAGED4	-4.431882214	1.32E-16	protein_coding
ENSG00000188263	IL17REL	-4.314255447	0.000109596	protein_coding
ENSG00000183153	GJD3	-4.177561833	0.000248027	protein_coding
ENSG00000159884	CCDC107	-3.900509294	7.18E-10	protein_coding
ENSG00000125533	BHLHE23	-3.765490121	0.002133716	protein_coding
ENSG00000166920	C15orf48	-3.765490121	0.002133716	protein_coding
ENSG00000128645	HOXD1	-3.666800696	0.003346055	protein_coding
ENSG00000258674	AC011448.1	-3.626030568	9.35E-06	protein_coding
ENSG00000127774	EMC6	-3.616919864	2.49E-07	protein_coding
ENSG00000185105	MYADML2	-3.560861317	0.005281544	protein_coding
ENSG00000241697	TMEFF1	-3.472256066	8.61E-08	protein_coding

ENSG00000171450	CDK5R2	-3.460955611	0.000348534	protein_coding
ENSG00000167476	JSRP1	-3.446521728	0.008390282	protein_coding
ENSG00000278558	TMEM191B	-3.41803842	1.56E-06	protein_coding
ENSG00000161958	FGF11	-3.397177551	0.000506526	protein_coding
ENSG00000073067	CYP2W1	-3.322334161	0.013412089	protein_coding
ENSG00000241360	PDXP	-3.265392632	3.94E-12	protein_coding
ENSG00000205670	SMIM11A	-3.257429287	1.00E-09	protein_coding
ENSG00000101084	RAB5IF	-3.233948316	6.03E-12	protein_coding
ENSG00000168282	MGAT2	-3.231665043	9.00E-07	protein_coding
ENSG00000180875	GREM2	-3.186952043	0.001597933	protein_coding
ENSG00000179388	EGR3	-3.186440965	0.021566548	protein_coding
ENSG00000135144	DTX1	-3.186440965	0.021566548	protein_coding
ENSG00000176472	ZNF575	-3.186440965	0.021566548	protein_coding
ENSG00000128482	RNF112	-3.186440965	0.021566548	protein_coding
ENSG00000058673	ZC3H11A	-3.186440965	0.021566548	protein_coding
ENSG00000244187	TMEM141	-3.173406503	3.81E-11	protein_coding
ENSG00000181029	TRAPPC5	-3.126940244	1.03E-06	protein_coding
ENSG00000179284	DAND5	-3.109470117	0.002364823	protein_coding
ENSG00000184357	HIST1H1B	-3.036403613	0.034866602	protein_coding
ENSG00000179580	RNF151	-3.036403613	0.034866602	protein_coding
ENSG00000161929	SCIMP	-3.036403613	0.034866602	protein_coding
ENSG00000116014	KISS1R	-3.024401896	0.000589	protein_coding
ENSG00000158427	TMSB15B	-2.897294553	6.17E-08	protein_coding
ENSG00000177989	ODF3B	-2.888440236	1.83E-05	protein_coding
ENSG00000139540	SLC39A5	-2.848412224	0.007864773	protein_coding
ENSG00000135100	HNF1A	-2.848412224	0.007864773	protein_coding
ENSG00000259803	SLC22A31	-2.845946005	1.48E-06	protein_coding
ENSG00000254413	CHKB-CPT1B	-2.840949818	0.001665133	protein_coding
ENSG00000284874	SEPT5-GP1BB	-2.77422135	0.002370371	protein_coding
ENSG00000187902	SHISA7	-2.77422135	0.002370371	protein_coding
ENSG00000168481	LGI3	-2.752560433	0.000280171	protein_coding
ENSG00000230778	ANKRD63	-2.729114802	0.000137515	protein_coding
ENSG00000179673	RPRML	-2.721793598	6.24E-07	protein_coding
ENSG00000162959	MEMO1	-2.686711021	6.53E-06	protein_coding
ENSG00000167996	FTH1	-2.671633874	1.17E-09	protein_coding
ENSG00000213996	TM6SF2	-2.643783421	0.017862218	protein_coding
ENSG00000173621	LRFN4	-2.593074649	8.91E-09	protein_coding
ENSG00000243989	ACY1	-2.565352822	0.002275868	protein_coding
ENSG00000001630	CYP51A1	-2.547082086	2.52E-07	protein_coding
ENSG00000130748	TMEM160	-2.542065788	1.32E-08	protein_coding
ENSG00000258429	PDF	-2.534975972	5.25E-08	protein_coding
ENSG00000185883	ATP6V0C	-2.529984575	3.27E-06	protein_coding
ENSG00000146221	TCTE1	-2.529443832	0.02704271	protein_coding

ENSG00000105251	SHD	-2.529443832	0.02704271	protein_coding
ENSG00000174791	RIN1	-2.529443832	0.02704271	protein_coding
ENSG00000009790	TRAF3IP3	-2.529443832	0.02704271	protein_coding
ENSG00000106003	LFNG	-2.486360495	3.71E-05	protein_coding
ENSG00000186010	NDUFA13	-2.48504363	1.96E-05	protein_coding
ENSG00000149925	ALDOA	-2.472084412	1.14E-06	protein_coding
ENSG00000162068	NTN3	-2.471361832	0.010033723	protein_coding
ENSG00000168913	ENHO	-2.460844186	2.10E-07	protein_coding
ENSG00000215612	HMX1	-2.447195519	3.98E-08	protein_coding
ENSG00000176919	C8G	-2.405256265	0.041032031	protein_coding
ENSG00000269226	TMSB15B	-2.405256265	0.041032031	protein_coding
ENSG00000146216	TTBK1	-2.405256265	0.041032031	protein_coding
ENSG00000176153	GPX2	-2.405256265	0.041032031	protein_coding
ENSG00000204335	SP5	-2.405256265	0.041032031	protein_coding
ENSG00000169314	C22orf15	-2.384553021	0.014485426	protein_coding
ENSG00000141744	PNMT	-2.373768745	0.006072876	protein_coding
ENSG00000100300	TSPO	-2.352138624	6.66E-08	protein_coding
ENSG00000161992	PRR35	-2.351271263	0.000110315	protein_coding
ENSG00000156875	MFSD14A	-2.328395513	9.69E-05	protein_coding
ENSG00000099866	MADCAM1	-2.321501041	0.000660415	protein_coding
ENSG00000271303	SRXN1	-2.321422709	1.01E-05	protein_coding
ENSG00000130700	GATA5	-2.31556555	0.001998931	protein_coding
ENSG00000132522	GPS2	-2.309260746	1.13E-05	protein_coding
ENSG00000183397	C19orf71	-2.2369455	0.000197348	protein_coding
ENSG00000105963	ADAP1	-2.230559877	6.96E-06	protein_coding
ENSG00000184702	5-Sep	-2.224235728	0.000812684	protein_coding
ENSG00000116032	GRIN3B	-2.209388459	0.001356148	protein_coding
ENSG00000179886	TIGD5	-2.205856409	6.81E-07	protein_coding
ENSG00000164794	KCNV1	-2.193495067	0.030348148	protein_coding
ENSG00000100038	TOP3B	-2.189574085	0.001019268	protein_coding
ENSG00000251493	FOXD1	-2.179038484	7.84E-07	protein_coding
ENSG00000160255	ITGB2	-2.164223407	0.004621058	protein_coding
ENSG00000149489	ROM1	-2.164223407	0.004621058	protein_coding
ENSG00000280789	PAGR1	-2.153001984	2.01E-06	protein_coding
ENSG00000182327	GLTPD2	-2.141142966	0.000974164	protein_coding
ENSG00000168209	DDIT4	-2.1375022	9.44E-07	protein_coding
ENSG00000262814	MRPL12	-2.130133222	1.24E-06	protein_coding
ENSG00000272325	NUDT3	-2.109573689	7.07E-06	protein_coding
ENSG00000167680	SEMA6B	-2.095270688	6.21E-06	protein_coding
ENSG00000178809	TRIM73	-2.087555688	0.043991311	protein_coding
ENSG00000148357	HMCN2	-2.087555688	0.043991311	protein_coding
ENSG00000213658	LAT	-2.087555688	0.043991311	protein_coding
ENSG00000179304	FAM156B	-2.087555688	0.043991311	protein_coding

ENSG00000130287	NCAN	-2.087555688	0.043991311	protein_coding
ENSG00000116176	TPSG1	-2.087555688	0.043991311	protein_coding
ENSG00000177374	HIC1	-2.07177523	0.000284129	protein_coding
ENSG00000151322	NPAS3	-2.070909227	0.023070854	protein_coding
ENSG00000240972	MIF	-2.053720637	0.008135331	protein_coding
ENSG00000243449	C4orf48	-2.05284796	3.40E-06	protein_coding
ENSG00000131650	KREMEN2	-2.050475964	2.00E-05	protein_coding
ENSG00000185668	POU3F1	-2.049505845	0.000283294	protein_coding
ENSG00000187045	TMPRSS6	-2.04870614	0.005290723	protein_coding
ENSG00000172936	MYD88	-2.040688197	0.000105733	protein_coding
ENSG00000167136	ENDOG	-2.039623575	4.70E-06	protein_coding
ENSG00000015413	DPEP1	-2.000689275	0.004613369	protein_coding
ENSG00000159674	SPON2	-1.998299844	0.006891763	protein_coding
ENSG00000273238	TMEM271	-1.996840618	0.000168126	protein_coding
ENSG00000181284	TMEM102	-1.996482614	2.55E-05	protein_coding
ENSG00000267216	AC020915.1	-1.990697743	0.018003167	protein_coding
ENSG00000130176	CNN1	-1.990697743	0.018003167	protein_coding
ENSG00000245848	CEBPA	-1.985133365	1.20E-05	protein_coding
ENSG00000203896	LIME1	-1.984343973	0.000784949	protein_coding
ENSG00000182103	FAM181B	-1.984343973	0.000784949	protein_coding
ENSG00000183628	DGCR6	-1.984100416	0.032292239	protein_coding
ENSG00000178531	CTXN1	-1.974980718	6.36E-06	protein_coding
ENSG00000142694	EVA1B	-1.97018595	0.000170467	protein_coding
ENSG00000204176	SYT15	-1.963919068	0.000243382	protein_coding
ENSG00000181409	AATK	-1.960546366	6.69E-05	protein_coding
ENSG00000249931	GOLGA8K	-1.955065557	0.005911861	protein_coding
ENSG00000167550	RHEBL1	-1.938348528	0.002617946	protein_coding
ENSG00000213977	TAX1BP3	-1.93404662	0.014380224	protein_coding
ENSG00000235169	SMIM1	-1.93404662	0.014380224	protein_coding
ENSG00000181408	UTS2R	-1.918942783	0.000310788	protein_coding
ENSG00000183048	SLC25A10	-1.917883634	1.39E-05	protein_coding
ENSG00000107738	VSIR	-1.91716585	0.024510598	protein_coding
ENSG00000183476	SH2D7	-1.91716585	0.024510598	protein_coding
ENSG00000248643	RBM14-RBM4	-1.91716585	0.024510598	protein_coding
ENSG00000274791	F8A2	-1.91716585	0.024510598	protein_coding
ENSG00000100836	PABPN1	-1.914210053	7.97E-06	protein_coding
ENSG00000198242	RPL23A	-1.911432662	6.85E-06	protein_coding
ENSG00000167700	MFSD3	-1.910407228	8.96E-06	protein_coding
ENSG00000162373	BEND5	-1.891731886	0.045202178	protein_coding
ENSG00000166337	TAF10	-1.875205813	3.76E-05	protein_coding
ENSG00000166206	GABRB3	-1.874113722	0.000614658	protein_coding
ENSG00000179168	GGN	-1.870268559	0.019137366	protein_coding
ENSG00000203883	SOX18	-1.864247677	0.000177085	protein_coding

ENSG00000167964	RAB26	-1.853594609	0.000291081	protein_coding
ENSG00000103260	METRN	-1.850300774	2.86E-05	protein_coding
ENSG00000182511	FES	-1.848025648	0.00355334	protein_coding
ENSG00000196118	CCDC189	-1.846404794	0.000288547	protein_coding
ENSG00000186193	SAPCD2	-1.846104423	2.05E-05	protein_coding
ENSG00000259529	AL136295.5	-1.839683923	0.033376343	protein_coding
ENSG00000100399	CHADL	-1.839683923	0.033376343	protein_coding
ENSG00000253304	TMEM200B	-1.839683923	0.033376343	protein_coding
ENSG00000261052	SULT1A3	-1.839683923	0.033376343	protein_coding
ENSG00000105404	RABAC1	-1.834822233	8.47E-05	protein_coding
ENSG00000278500	AC009336.2	-1.833247366	0.008204261	protein_coding
ENSG00000198517	MAFK	-1.833182353	3.71E-05	protein_coding
ENSG00000160161	CILP2	-1.830804302	0.000154123	protein_coding
ENSG00000111775	COX6A1	-1.813333709	2.38E-05	protein_coding
ENSG00000106992	AK1	-1.812504755	0.000536078	protein_coding
ENSG00000008516	MMP25	-1.812038524	0.00701327	protein_coding
ENSG00000125652	ALKBH7	-1.810669497	4.21E-05	protein_coding
ENSG00000234965	SHISA8	-1.809508696	0.000124997	protein_coding
ENSG00000145949	MYLK4	-1.808841046	0.012495401	protein_coding
ENSG00000181781	ODF3L2	-1.803540091	0.025475297	protein_coding
ENSG00000179588	ZFPM1	-1.802510128	7.10E-05	protein_coding
ENSG00000117984	CTSD	-1.785162737	3.48E-05	protein_coding
ENSG00000169972	PUSL1	-1.784149117	4.54E-05	protein_coding
ENSG00000168061	SAC3D1	-1.782693185	3.77E-05	protein_coding
ENSG00000127588	GNG13	-1.776969204	0.019987884	protein_coding
ENSG00000173928	SWSAP1	-1.77667478	0.000469394	protein_coding
ENSG00000131730	CKMT2	-1.770368427	0.008764824	protein_coding
ENSG00000103024	NME3	-1.764825985	3.75E-05	protein_coding
ENSG00000268606	MAGEA2	-1.761820358	0.001379077	protein_coding
ENSG00000100926	TM9SF1	-1.760523929	0.00025249	protein_coding
ENSG00000157766	ACAN	-1.757803372	0.045435959	protein_coding
ENSG00000130812	ANGPTL6	-1.757803372	0.045435959	protein_coding
ENSG00000144485	HES6	-1.756019231	5.89E-05	protein_coding
ENSG00000198835	GJC2	-1.755536952	0.002621569	protein_coding
ENSG00000174886	NDUFA11	-1.7523753	8.54E-05	protein_coding
ENSG00000130675	MNX1	-1.751605187	5.74E-05	protein_coding
ENSG00000225663	MCRIP1	-1.746415291	4.71E-05	protein_coding
ENSG00000100726	TELO2	-1.745634563	3.91E-05	protein_coding
ENSG00000187688	TRPV2	-1.733574945	0.033910222	protein_coding
ENSG00000179862	CITED4	-1.729290739	0.000921866	protein_coding
ENSG00000184163	C1QTNF12	-1.727458862	0.010958385	protein_coding
ENSG00000180834	MAP6D1	-1.727458862	0.010958385	protein_coding
ENSG00000160325	CACFD1	-1.725067717	0.000120811	protein_coding

ENSG00000257704	INAFM1	-1.724626615	0.000371577	protein_coding
ENSG00000168040	FADD	-1.713340591	0.000106933	protein_coding
ENSG00000167701	GPT	-1.709872628	0.000992022	protein_coding
ENSG00000159423	ALDH4A1	-1.709031715	0.000384966	protein_coding
ENSG00000161653	NAGS	-1.699851509	0.006889608	protein_coding
ENSG00000239857	GET4	-1.697248025	0.000175395	protein_coding
ENSG00000184897	H1FX	-1.694640269	6.12E-05	protein_coding
ENSG00000213638	ADAT3	-1.694420859	0.000167991	protein_coding
ENSG00000051523	CYBA	-1.693963712	0.000142788	protein_coding
ENSG00000116544	DLGAP3	-1.687357982	0.005336669	protein_coding
ENSG00000177045	SIX5	-1.677350881	0.00022047	protein_coding
ENSG00000127564	PKMYT1	-1.672360128	8.51E-05	protein_coding
ENSG00000132702	HAPLN2	-1.6700316	0.003514632	protein_coding
ENSG00000130751	NPAS1	-1.665919435	0.000111871	protein_coding
ENSG00000196976	LAGE3	-1.665298123	9.22E-05	protein_coding
ENSG00000105327	BBC3	-1.660554226	0.000143524	protein_coding
ENSG00000176532	PRR15	-1.660043051	0.045117465	protein_coding
ENSG00000180044	C3orf80	-1.65836274	0.000829098	protein_coding
ENSG00000188290	HES4	-1.655142807	9.80E-05	protein_coding
ENSG00000188368	PRR19	-1.652113595	0.034088382	protein_coding
ENSG00000113721	PDGFRB	-1.652113595	0.034088382	protein_coding
ENSG00000103067	ESRP2	-1.646106876	0.026497687	protein_coding
ENSG00000186998	EMID1	-1.64161145	0.000277653	protein_coding
ENSG00000188152	NUTM2G	-1.641399137	0.021096335	protein_coding
ENSG00000244274	DBNDD2	-1.641399137	0.021096335	protein_coding
ENSG00000090971	NAT14	-1.639702413	0.000138832	protein_coding
ENSG00000182871	COL18A1	-1.637530104	0.000126577	protein_coding
ENSG00000261221	ZNF865	-1.636565973	0.000176016	protein_coding
ENSG00000099625	CBARP	-1.632244504	0.000436055	protein_coding
ENSG00000158792	SPATA2L	-1.63194428	0.000200983	protein_coding
ENSG00000198576	ARC	-1.622247281	0.005296084	protein_coding
ENSG00000143382	ADAMTSL4	-1.620303293	0.004313732	protein_coding
ENSG00000161955	TNFSF13	-1.619468525	0.003928027	protein_coding
ENSG00000125657	TNFSF9	-1.616024352	0.000625839	protein_coding
ENSG00000163362	INAVA	-1.613367862	0.001575555	protein_coding
ENSG00000204060	FOXO6	-1.611066591	0.001096601	protein_coding
ENSG00000104899	AMH	-1.610898616	0.000189366	protein_coding
ENSG00000161847	RAVER1	-1.606259028	0.000159246	protein_coding
ENSG00000130589	HELZ2	-1.600021213	0.000231284	protein_coding
ENSG00000119673	ACOT2	-1.599032526	0.000202281	protein_coding
ENSG00000163995	ABLIM2	-1.596505422	0.004998675	protein_coding
ENSG00000172346	CSDC2	-1.59618791	0.005549857	protein_coding
ENSG00000285043	AC093512.2	-1.59618791	0.005549857	protein_coding

ENSG00000174276	ZNHIT2	-1.594523944	0.000324665	protein_coding
ENSG00000130643	CALY	-1.593923998	0.010511842	protein_coding
ENSG00000166546	BEAN1	-1.590496342	0.021443929	protein_coding
ENSG00000158050	DUSP2	-1.586241643	0.001591919	protein_coding
ENSG00000183186	C2CD4C	-1.586241643	0.001591919	protein_coding
ENSG00000171017	LRRC8E	-1.585385127	0.044489089	protein_coding
ENSG00000130829	DUSP9	-1.583915424	0.000186446	protein_coding
ENSG00000244486	SCARF2	-1.579339663	0.000234094	protein_coding
ENSG00000197019	SERTAD1	-1.57716137	0.000849028	protein_coding
ENSG00000162591	MEGF6	-1.575306479	0.001531537	protein_coding
ENSG00000104524	PYCR3	-1.574983191	0.000224227	protein_coding
ENSG00000175756	AURKAIP1	-1.573816034	0.000204152	protein_coding
ENSG00000196421	C20orf204	-1.573754999	0.00522492	protein_coding
ENSG00000161682	FAM171A2	-1.572729854	0.000249673	protein_coding
ENSG00000181588	MEX3D	-1.570954588	0.000264409	protein_coding
ENSG00000218891	ZNF579	-1.568788315	0.000304621	protein_coding
ENSG00000198113	TOR4A	-1.56843775	0.002266125	protein_coding
ENSG00000026036	RTEL1-TNFRSF6B	-1.567327832	0.002403488	protein_coding
ENSG00000160401	CFAP157	-1.567327832	0.002403488	protein_coding
ENSG00000103254	FAM173A	-1.564879665	0.00032642	protein_coding
ENSG00000176340	COX8A	-1.563921952	0.000240056	protein_coding
ENSG00000185269	NOTUM	-1.562165648	0.010850785	protein_coding
ENSG00000169750	RAC3	-1.553093639	0.000238837	protein_coding
ENSG00000006327	TNFRSF12A	-1.546730772	0.001549286	protein_coding
ENSG00000146826	C7orf43	-1.539534275	0.000474189	protein_coding
ENSG00000129911	KLF16	-1.538945922	0.000352641	protein_coding
ENSG00000136826	KLF4	-1.535852656	0.00167674	protein_coding
ENSG00000283378	CNTNAP3C	-1.534974084	0.033832332	protein_coding
ENSG00000131116	ZNF428	-1.533588802	0.000371098	protein_coding
ENSG00000180828	BHLHE22	-1.529093248	0.01296303	protein_coding
ENSG00000129932	DOHH	-1.527397988	0.000384771	protein_coding
ENSG00000249992	TMEM158	-1.52468671	0.000615234	protein_coding
ENSG00000168936	TMEM129	-1.523893988	0.000329865	protein_coding
ENSG00000140323	DISP2	-1.522074389	0.004879682	protein_coding
ENSG00000213563	C8orf82	-1.519092291	0.00039331	protein_coding
ENSG00000174871	CNIH2	-1.519031864	0.003322547	protein_coding
ENSG00000171811	CFAP46	-1.517562331	0.018121122	protein_coding
ENSG00000104529	EEF1D	-1.513644708	0.000338249	protein_coding
ENSG00000151651	ADAM8	-1.511641142	0.003034831	protein_coding
ENSG00000220008	LINGO3	-1.510323509	0.021872151	protein_coding
ENSG00000139192	TAPBPL	-1.510323509	0.021872151	protein_coding
ENSG00000185988	PLK5	-1.508197631	0.011440165	protein_coding

ENSG00000185215	TNFAIP2	-1.508197631	0.011440165	protein_coding
ENSG00000103269	RHBDL1	-1.507007349	0.001954481	protein_coding
ENSG00000103326	CAPN15	-1.489813759	0.000407232	protein_coding
ENSG00000167397	VKORC1	-1.487667529	0.001382596	protein_coding
ENSG00000185761	ADAMTSL5	-1.487336061	0.018332243	protein_coding
ENSG00000169230	PRELID1	-1.48588687	0.00041089	protein_coding
ENSG00000161956	SENP3	-1.47917144	0.000520095	protein_coding
ENSG00000151006	PRSS53	-1.477903224	0.013488043	protein_coding
ENSG00000105135	ILVBL	-1.477512373	0.000494941	protein_coding
ENSG00000173638	SLC19A1	-1.472577967	0.000496992	protein_coding
ENSG00000185347	TEDC1	-1.47070664	0.000560849	protein_coding
ENSG00000184343	SRPK3	-1.470696337	0.003296007	protein_coding
ENSG00000105722	ERF	-1.467610931	0.000562435	protein_coding
ENSG00000221821	C6orf226	-1.467413944	0.026780029	protein_coding
ENSG00000167100	SAMD14	-1.466295295	0.008470153	protein_coding
ENSG00000129951	PLPPR3	-1.463186832	0.001051065	protein_coding
ENSG00000285976	AL135905.2	-1.462436335	0.002982265	protein_coding
ENSG00000174021	GNG5	-1.462414178	0.001391223	protein_coding
ENSG00000165804	ZNF219	-1.461827192	0.000695952	protein_coding
ENSG00000129993	CBFA2T3	-1.456602972	0.007809391	protein_coding
ENSG00000126062	TMEM115	-1.456082998	0.00062013	protein_coding
ENSG00000168961	LGALS9	-1.45462268	0.033173684	protein_coding
ENSG00000137267	TUBB2A	-1.453433533	0.000708874	protein_coding
ENSG00000243678	NME2	-1.448900869	0.004090345	protein_coding
ENSG00000182325	FBXL6	-1.447717078	0.00077698	protein_coding
ENSG00000261150	EPPK1	-1.44695901	0.015902772	protein_coding
ENSG00000122592	HOXA7	-1.444405504	0.004370755	protein_coding
ENSG00000212123	PRR22	-1.44370519	0.001506118	protein_coding
ENSG00000168993	CPLX1	-1.439481129	0.001129045	protein_coding
ENSG00000160446	ZDHHC12	-1.439416324	0.000910093	protein_coding
ENSG00000175602	CCDC85B	-1.435706886	0.000724138	protein_coding
ENSG00000126603	GLIS2	-1.433720872	0.002518779	protein_coding
ENSG00000128713	HOXD11	-1.431960125	0.001082401	protein_coding
ENSG00000172366	MCRIP2	-1.430211973	0.000893224	protein_coding
ENSG00000127585	FBXL16	-1.429717333	0.00433205	protein_coding
ENSG00000158747	NBL1	-1.416426598	0.001404642	protein_coding
ENSG00000188157	AGRN	-1.414829477	0.000806122	protein_coding
ENSG00000183114	FAM43B	-1.412177801	0.004566454	protein_coding
ENSG00000205544	TMEM256	-1.406535489	0.002551178	protein_coding
ENSG00000144488	ESPNL	-1.406378744	0.001523294	protein_coding
ENSG00000205609	EIF3CL	-1.405972189	0.001327587	protein_coding
ENSG00000168802	CHTF8	-1.405895171	0.000871169	protein_coding
ENSG00000125820	NKX2-2	-1.402558722	0.006166847	protein_coding

ENSG00000188322	SBK1	-1.401639701	0.001340018	protein_coding
ENSG00000142235	LMTK3	-1.401100663	0.004513382	protein_coding
ENSG00000138119	MYOF	-1.400297207	0.010248829	protein_coding
ENSG00000183741	CBX6	-1.39788218	0.000852223	protein_coding
ENSG00000128626	MRPS12	-1.395255061	0.000977952	protein_coding
ENSG00000099974	DDTL	-1.39450865	0.0029212	protein_coding
ENSG00000161970	RPL26	-1.394330913	0.001028732	protein_coding
ENSG00000131187	F12	-1.394026179	0.001842597	protein_coding
ENSG00000185090	MANEAL	-1.393669345	0.001113075	protein_coding
ENSG00000133597	ADCK2	-1.393607793	0.000991389	protein_coding
ENSG00000123576	ESX1	-1.392374621	0.008684563	protein_coding
ENSG00000185022	MAFF	-1.391629371	0.011442905	protein_coding
ENSG00000182612	TSPAN10	-1.391267345	0.003028139	protein_coding
ENSG00000179044	EXOC3L1	-1.388656935	0.026398783	protein_coding
ENSG00000167702	KIFC2	-1.387956726	0.001353959	protein_coding
ENSG00000254470	AP5B1	-1.387649647	0.001104642	protein_coding
ENSG00000184990	SIVA1	-1.387340759	0.00095807	protein_coding
ENSG00000276345	AC004556.1	-1.384584798	0.0095587	protein_coding
ENSG00000184916	JAG2	-1.3825477	0.00112842	protein_coding
ENSG00000066230	SLC9A3	-1.379384914	0.005822763	protein_coding
ENSG00000132003	ZSWIM4	-1.379218481	0.008225538	protein_coding
ENSG00000177943	MAMDC4	-1.378180106	0.004048099	protein_coding
ENSG00000132329	RAMP1	-1.37761371	0.001649936	protein_coding
ENSG00000125775	SDCBP2	-1.377565193	0.040101413	protein_coding
ENSG00000184281	TSSC4	-1.377188185	0.001270967	protein_coding
ENSG00000171443	ZNF524	-1.373769351	0.004247991	protein_coding
ENSG00000213445	SIPA1	-1.373687616	0.001305075	protein_coding
ENSG00000162413	KLHL21	-1.372197991	0.001268824	protein_coding
ENSG00000162302	RPS6KA4	-1.370278378	0.001335543	protein_coding
ENSG00000117862	TXNDC12	-1.3702094	0.002189851	protein_coding
ENSG00000185615	PDIA2	-1.369181327	0.004464258	protein_coding
ENSG00000238227	TMEM250	-1.368658926	0.001135571	protein_coding
ENSG00000160113	NR2F6	-1.368101521	0.001151411	protein_coding
ENSG00000105559	PLEKHA4	-1.366667351	0.006714756	protein_coding
ENSG00000008710	PKD1	-1.366393646	0.001200451	protein_coding
ENSG00000115266	APC2	-1.365993073	0.002567315	protein_coding
ENSG00000129757	CDKN1C	-1.365326769	0.006369487	protein_coding
ENSG00000161996	WDR90	-1.362523633	0.001325042	protein_coding
ENSG00000165655	ZNF503	-1.361161074	0.001207805	protein_coding
ENSG00000123360	PDE1B	-1.361070426	0.005330258	protein_coding
ENSG00000122971	ACADS	-1.36062162	0.001749744	protein_coding
ENSG00000183317	EPHA10	-1.360498938	0.016548945	protein_coding
ENSG00000160685	ZBTB7B	-1.359887632	0.001555867	protein_coding

ENSG00000167543	TP53I13	-1.35797079	0.001510799	protein_coding
ENSG00000130511	SSBP4	-1.356654547	0.00133384	protein_coding
ENSG00000262246	CORO7	-1.355725985	0.004153771	protein_coding
ENSG00000174939	ASPHD1	-1.354960289	0.010860269	protein_coding
ENSG00000185803	SLC52A2	-1.348490377	0.001573675	protein_coding
ENSG00000161677	JOSD2	-1.346841356	0.002666411	protein_coding
ENSG00000178149	DALRD3	-1.345879776	0.002179059	protein_coding
ENSG00000106006	HOXA6	-1.345821169	0.003923782	protein_coding
ENSG00000126709	IFI6	-1.345263198	0.010994238	protein_coding
ENSG00000130052	STARD8	-1.343871295	0.0070897	protein_coding
ENSG00000188760	TMEM198	-1.340958501	0.002529213	protein_coding
ENSG00000107281	NPDC1	-1.338521934	0.001705223	protein_coding
ENSG00000171425	ZNF581	-1.33816155	0.001825393	protein_coding
ENSG00000176428	VPS37D	-1.337423398	0.005728899	protein_coding
ENSG00000149761	NUDT22	-1.336489671	0.002559538	protein_coding
ENSG00000187730	GABRD	-1.336489285	0.008637514	protein_coding
ENSG00000159214	CCDC24	-1.335323606	0.014864917	protein_coding
ENSG00000100429	HDAC10	-1.334974326	0.0167062	protein_coding
ENSG00000170608	FOXA3	-1.334067115	0.021979843	protein_coding
ENSG00000156860	FBRS	-1.333802682	0.001483329	protein_coding
ENSG00000168477	TNXB	-1.332708768	0.031103696	protein_coding
ENSG00000100918	REC8	-1.332708768	0.031103696	protein_coding
ENSG00000069482	GAL	-1.331337472	0.001595051	protein_coding
ENSG00000127220	ABHD8	-1.330932928	0.003639631	protein_coding
ENSG00000168071	CCDC88B	-1.330677135	0.006953612	protein_coding
ENSG00000254995	STX16-NPEPL1	-1.330451424	0.049028188	protein_coding
ENSG00000204388	HSPA1B	-1.320040048	0.001663938	protein_coding
ENSG00000160326	SLC2A6	-1.318739986	0.002915705	protein_coding
ENSG00000177427	MIEF2	-1.316724581	0.002837651	protein_coding
ENSG00000117013	KCNQ4	-1.316549776	0.013582069	protein_coding
ENSG00000128242	GAL3ST1	-1.31608452	0.030695741	protein_coding
ENSG00000170604	IRF2BP1	-1.314350757	0.001815214	protein_coding
ENSG00000151611	MMAA	-1.312998796	0.016825845	protein_coding
ENSG00000181649	PHLDA2	-1.309781524	0.012477797	protein_coding
ENSG00000127666	TICAM1	-1.309781524	0.012477797	protein_coding
ENSG00000261794	GOLGA8H	-1.308863147	0.04754413	protein_coding
ENSG00000185112	FAM43A	-1.305395119	0.005916014	protein_coding
ENSG00000239264	TXNDC5	-1.304809098	0.0035895	protein_coding
ENSG00000213906	LTB4R2	-1.304113006	0.010498239	protein_coding
ENSG00000025708	TYMP	-1.302010095	0.012577059	protein_coding
ENSG00000104883	PEX11G	-1.302010095	0.012577059	protein_coding
ENSG00000214967	NPIPA7	-1.302010095	0.012577059	protein_coding

ENSG00000136295	TTYH3	-1.300515356	0.001920975	protein_coding
ENSG00000163874	ZC3H12A	-1.300373534	0.00690822	protein_coding
ENSG00000149809	TM7SF2	-1.299025738	0.002939148	protein_coding
ENSG00000099849	RASSF7	-1.298627581	0.003122318	protein_coding
ENSG00000173546	CSPG4	-1.296174414	0.005761229	protein_coding
ENSG00000160226	CFAP410	-1.294244348	0.004697147	protein_coding
ENSG00000188763	FZD9	-1.29312195	0.003701595	protein_coding
ENSG00000136167	LCP1	-1.29033177	0.003023635	protein_coding
ENSG00000142544	CTU1	-1.289869645	0.002994335	protein_coding
ENSG00000184160	ADRA2C	-1.28896409	0.002676693	protein_coding
ENSG00000161835	GRASP	-1.287609968	0.029914245	protein_coding
ENSG00000063176	SPHK2	-1.287010695	0.003058123	protein_coding
ENSG00000105376	ICAM5	-1.286950453	0.003130647	protein_coding
ENSG00000068137	PLEKHH3	-1.286157506	0.002463842	protein_coding
ENSG00000185818	NAT8L	-1.284035882	0.002304281	protein_coding
ENSG00000186907	RTN4RL2	-1.282056262	0.004636612	protein_coding
ENSG00000160345	C9orf116	-1.280823322	0.006242505	protein_coding
ENSG00000198931	APRT	-1.280593277	0.002185917	protein_coding
ENSG00000148832	PAOX	-1.277088579	0.016984831	protein_coding
ENSG00000147119	CHST7	-1.276140506	0.004573553	protein_coding
ENSG00000129194	SOX15	-1.275322412	0.029542889	protein_coding
ENSG00000109906	ZBTB16	-1.275322412	0.029542889	protein_coding
ENSG00000214026	MRPL23	-1.272473443	0.002516834	protein_coding
ENSG00000163623	NKX6-1	-1.267987523	0.004069632	protein_coding
ENSG00000136490	LIMD2	-1.266889828	0.003579474	protein_coding
ENSG00000111786	SRSF9	-1.261109563	0.0025382	protein_coding
ENSG00000168000	BSCL2	-1.258943275	0.043684225	protein_coding
ENSG00000161328	LRRC56	-1.258808248	0.021473972	protein_coding
ENSG00000119669	IRF2BPL	-1.256991907	0.003399879	protein_coding
ENSG00000128011	LRFN1	-1.256213367	0.003293086	protein_coding
ENSG00000267796	LIN37	-1.25598746	0.034842559	protein_coding
ENSG00000162576	MXRA8	-1.255806331	0.005461128	protein_coding
ENSG00000008283	CYB561	-1.255793606	0.004510333	protein_coding
ENSG00000143590	EFNA3	-1.254984185	0.007182091	protein_coding
ENSG00000126391	FRMD8	-1.250426799	0.003175703	protein_coding
ENSG00000119559	C19orf25	-1.249386046	0.003203421	protein_coding
ENSG00000169884	WNT10B	-1.248941764	0.004876986	protein_coding
ENSG00000126461	SCAF1	-1.24873143	0.002868972	protein_coding
ENSG00000174307	PHLDA3	-1.248159492	0.003284778	protein_coding
ENSG00000188549	CCDC9B	-1.246647469	0.01145021	protein_coding
ENSG00000167968	DNASE1L2	-1.245907925	0.042571852	protein_coding
ENSG00000162066	AMDHD2	-1.245735581	0.005003214	protein_coding
ENSG00000112787	FBRSL1	-1.244274465	0.002912174	protein_coding

ENSG00000142330	CAPN10	-1.244168393	0.003600321	protein_coding
ENSG00000007541	PIGQ	-1.244165011	0.003281797	protein_coding
ENSG00000213923	CSNK1E	-1.241899901	0.0029589	protein_coding
ENSG00000243279	PRAF2	-1.239026686	0.005577294	protein_coding
ENSG00000125931	CITED1	-1.237851965	0.004995688	protein_coding
ENSG00000104044	OCA2	-1.234346983	0.010492033	protein_coding
ENSG00000136856	SLC2A8	-1.234276275	0.004011908	protein_coding
ENSG00000167600	CYP2S1	-1.234021272	0.011041262	protein_coding
ENSG00000115884	SDC1	-1.232996722	0.004620553	protein_coding
ENSG00000130881	LRP3	-1.232420323	0.003287898	protein_coding
ENSG00000161618	ALDH16A1	-1.23101991	0.004073274	protein_coding
ENSG00000120913	PDLIM2	-1.230860585	0.018901676	protein_coding
ENSG00000066735	KIF26A	-1.229772187	0.003468016	protein_coding
ENSG00000221838	AP4M1	-1.228582769	0.005454297	protein_coding
ENSG00000173153	ESRRA	-1.22528042	0.003776767	protein_coding
ENSG00000163888	CAMK2N2	-1.223656254	0.040567706	protein_coding
ENSG00000118231	CRYGD	-1.22245883	0.009862478	protein_coding
ENSG00000132507	EIF5A	-1.221978876	0.003341394	protein_coding
ENSG00000165644	COMTD1	-1.221667942	0.003976322	protein_coding
ENSG00000198324	PHETA1	-1.220589926	0.005507158	protein_coding
ENSG00000167536	DHRS13	-1.218440374	0.005948764	protein_coding
ENSG00000128311	TST	-1.217707141	0.004225961	protein_coding
ENSG00000128274	A4GALT	-1.215796078	0.023740669	protein_coding
ENSG00000074181	NOTCH3	-1.215154806	0.003850168	protein_coding
ENSG00000179431	FJX1	-1.213583197	0.004426543	protein_coding
ENSG00000167716	WDR81	-1.213099061	0.004361129	protein_coding
ENSG00000196711	ALKAL1	-1.211852901	0.013557267	protein_coding
ENSG00000008735	MAPK8IP2	-1.210433725	0.003973741	protein_coding
ENSG00000160360	GPSM1	-1.209112325	0.004042095	protein_coding
ENSG00000183691	NOG	-1.206050154	0.007983103	protein_coding
ENSG00000126749	EMG1	-1.205582318	0.004406156	protein_coding
ENSG00000125510	OPRL1	-1.205362441	0.038817849	protein_coding
ENSG00000165886	UBTD1	-1.205244554	0.005685451	protein_coding
ENSG00000160991	ORAI2	-1.204567574	0.004730026	protein_coding
ENSG00000139832	RAB20	-1.204221154	0.023443495	protein_coding
ENSG00000277758	FO681492.1	-1.202299244	0.014618718	protein_coding
ENSG00000040608	RTN4R	-1.200696193	0.010018876	protein_coding
ENSG00000009694	TENM1	-1.200469408	0.006077466	protein_coding
ENSG00000152082	MZT2B	-1.200361585	0.004410352	protein_coding
ENSG00000179111	HES7	-1.199549415	0.048597149	protein_coding
ENSG00000081692	JMJD4	-1.199517795	0.005145234	protein_coding
ENSG00000108839	ALOX12	-1.197838705	0.013743256	protein_coding
ENSG00000141965	FEM1A	-1.197007439	0.00700597	protein_coding

ENSG00000103245	CIAO3	-1.196263532	0.004797722	protein_coding
ENSG00000204387	C6orf48	-1.196090099	0.005847008	protein_coding
ENSG00000112658	SRF	-1.195538717	0.004382231	protein_coding
ENSG00000197982	C1orf122	-1.194002323	0.005152789	protein_coding
ENSG00000125753	VASP	-1.193956331	0.004878744	protein_coding
ENSG00000198108	CHSY3	-1.192263441	0.015803617	protein_coding
ENSG00000186654	PRR5	-1.191538025	0.006022428	protein_coding
ENSG00000185504	FAAP100	-1.190978751	0.004632174	protein_coding
ENSG00000004838	ZMYND10	-1.190583855	0.047212297	protein_coding
ENSG00000105289	TJP3	-1.190583855	0.047212297	protein_coding
ENSG00000185800	DMWD	-1.190282193	0.004997608	protein_coding
ENSG00000185838	GNB1L	-1.190068217	0.005489694	protein_coding
ENSG00000213533	STIMATE	-1.190055613	0.037281246	protein_coding
ENSG00000105699	LSR	-1.189975615	0.006051246	protein_coding
ENSG00000198598	MMP17	-1.188142267	0.008680636	protein_coding
ENSG00000166189	HPS6	-1.187116941	0.005786778	protein_coding
ENSG00000090266	NDUFB2	-1.185430394	0.004817797	protein_coding
ENSG00000174721	FGFBP3	-1.184988516	0.018736468	protein_coding
ENSG00000177733	HNRNPA0	-1.183814894	0.004500265	protein_coding
ENSG00000275993	SIK1B	-1.183302429	0.036582079	protein_coding
ENSG00000167604	NFKBID	-1.182932172	0.01393897	protein_coding
ENSG00000109089	CDR2L	-1.182506723	0.005430587	protein_coding
ENSG00000189120	SP6	-1.182461283	0.045931264	protein_coding
ENSG00000147813	NAPRT	-1.1821825	0.015857762	protein_coding
ENSG00000213347	MXD3	-1.181689957	0.005850198	protein_coding
ENSG00000177595	PIDD1	-1.181539898	0.005297996	protein_coding
ENSG00000128591	FLNC	-1.180572495	0.004966583	protein_coding
ENSG00000213859	KCTD11	-1.17970241	0.014839224	protein_coding
ENSG00000186222	BLOC1S4	-1.178114186	0.005992335	protein_coding
ENSG00000173457	PPP1R14B	-1.177605569	0.004751913	protein_coding
ENSG00000148411	NACC2	-1.176704781	0.005749299	protein_coding
ENSG00000102981	PARD6A	-1.175068034	0.044743519	protein_coding
ENSG00000103495	MAZ	-1.173892635	0.004905849	protein_coding
ENSG00000117410	ATP6V0B	-1.17342585	0.005050741	protein_coding
ENSG00000055118	KCNH2	-1.173202811	0.015901775	protein_coding
ENSG00000128268	MGAT3	-1.173042604	0.006157794	protein_coding
ENSG00000145214	DGKQ	-1.170942093	0.006021378	protein_coding
ENSG00000160201	U2AF1	-1.170500429	0.008243672	protein_coding
ENSG00000187140	FOXD3	-1.17021658	0.012812646	protein_coding
ENSG00000214063	TSPAN4	-1.169219273	0.00639358	protein_coding
ENSG00000099385	BCL7C	-1.168105642	0.006079629	protein_coding
ENSG00000167470	MIDN	-1.166482313	0.005520879	protein_coding
ENSG00000101220	C20orf27	-1.164382739	0.005307507	protein_coding

ENSG00000125740	FOSB	-1.160866205	0.034165251	protein_coding
ENSG00000177542	SLC25A22	-1.160859635	0.006128465	protein_coding
ENSG00000204922	UQCC3	-1.160467593	0.007560008	protein_coding
ENSG00000140320	BAHD1	-1.159692366	0.006117718	protein_coding
ENSG00000164107	HAND2	-1.159357507	0.024868925	protein_coding
ENSG00000088727	KIF9	-1.15847506	0.018507342	protein_coding
ENSG00000125835	SNRPB	-1.155777457	0.005508556	protein_coding
ENSG00000162849	KIF26B	-1.155681806	0.017093035	protein_coding
ENSG00000131094	C1QL1	-1.155455595	0.011067613	protein_coding
ENSG00000108784	NAGLU	-1.155428333	0.006300305	protein_coding
ENSG00000020129	NCDN	-1.155272517	0.005843537	protein_coding
ENSG00000139438	FAM222A	-1.154197674	0.010119109	protein_coding
ENSG00000108557	RAI1	-1.152711842	0.006241563	protein_coding
ENSG00000142627	EPHA2	-1.152674479	0.007952166	protein_coding
ENSG00000273604	EPOP	-1.152341238	0.007014917	protein_coding
ENSG00000165879	FRAT1	-1.152136247	0.009490703	protein_coding
ENSG00000100065	CARD10	-1.151890213	0.00715608	protein_coding
ENSG00000183779	ZNF703	-1.151740887	0.005665021	protein_coding
ENSG00000140691	ARMC5	-1.150689318	0.008076974	protein_coding
ENSG00000161179	YDJC	-1.148632413	0.005924888	protein_coding
ENSG00000130810	PPAN	-1.147538018	0.011013732	protein_coding
ENSG00000140968	IRF8	-1.14665502	0.014402495	protein_coding
ENSG00000160877	NACC1	-1.145816726	0.006129493	protein_coding
ENSG00000184986	TMEM121	-1.145055475	0.008997218	protein_coding
ENSG00000103066	PLA2G15	-1.144861622	0.009276308	protein_coding
ENSG00000111671	SPSB2	-1.144816208	0.020003231	protein_coding
ENSG00000156381	ANKRD9	-1.143325051	0.006959518	protein_coding
ENSG00000106330	MOSPD3	-1.143324915	0.010896389	protein_coding
ENSG00000101311	FERMT1	-1.142335406	0.019947493	protein_coding
ENSG00000187634	SAMD11	-1.142159108	0.006564423	protein_coding
ENSG00000189366	ALG1L	-1.142105185	0.027297833	protein_coding
ENSG00000275074	NUDT18	-1.141845148	0.014462221	protein_coding
ENSG00000144649	FAM198A	-1.140881216	0.021996589	protein_coding
ENSG00000119715	ESRRB	-1.140522976	0.017045813	protein_coding
ENSG00000070423	RNF126	-1.138449059	0.006503478	protein_coding
ENSG00000171159	C9orf16	-1.137477369	0.007810535	protein_coding
ENSG00000127084	FGD3	-1.137454528	0.038387234	protein_coding
ENSG00000130702	LAMA5	-1.136086056	0.006828803	protein_coding
ENSG00000130382	MLLT1	-1.135905756	0.006620667	protein_coding
ENSG00000188566	NDOR1	-1.134489078	0.007681472	protein_coding
ENSG00000176170	SPHK1	-1.134357825	0.00942682	protein_coding
ENSG00000109736	MFSD10	-1.132370595	0.007195771	protein_coding
ENSG00000104880	ARHGEF18	-1.131254482	0.01968691	protein_coding

ENSG00000130208	APOC1	-1.131050054	0.010024373	protein_coding
ENSG00000063660	GPC1	-1.130252003	0.007591794	protein_coding
ENSG00000148343	MIGA2	-1.13018627	0.007622649	protein_coding
ENSG00000120738	EGR1	-1.129807786	0.012437341	protein_coding
ENSG00000107816	LZTS2	-1.128103601	0.007368029	protein_coding
ENSG00000050820	BCAR1	-1.127532291	0.00744024	protein_coding
ENSG00000169635	HIC2	-1.127076103	0.007242824	protein_coding
ENSG00000170906	NDUFA3	-1.125416218	0.007754895	protein_coding
ENSG00000167775	CD320	-1.124428502	0.007207943	protein_coding
ENSG00000184058	TBX1	-1.122401514	0.007387855	protein_coding
ENSG00000180447	GAS1	-1.12221183	0.008390892	protein_coding
ENSG00000107859	PITX3	-1.120752969	0.045117399	protein_coding
ENSG00000072195	SPEG	-1.120252218	0.008869534	protein_coding
ENSG00000159714	ZDHHC1	-1.120124828	0.011481661	protein_coding
ENSG00000159314	ARHGAP27	-1.118121291	0.013506089	protein_coding
ENSG00000175040	CHST2	-1.117643124	0.010469837	protein_coding
ENSG00000141570	CBX8	-1.114609987	0.010011362	protein_coding
ENSG00000180900	SCRIB	-1.114600919	0.007459868	protein_coding
ENSG00000166595	CIAO2B	-1.114272363	0.008108398	protein_coding
ENSG00000100263	RHBDD3	-1.114168952	0.008162588	protein_coding
ENSG00000099617	EFNA2	-1.113656658	0.043119316	protein_coding
ENSG00000103335	PIEZO1	-1.113309789	0.00769284	protein_coding
ENSG00000284976	BX255925.3	-1.111597019	0.009071626	protein_coding
ENSG00000138835	RGS3	-1.110462594	0.042214057	protein_coding
ENSG00000142327	RNPEPL1	-1.110461846	0.00906644	protein_coding
ENSG00000104960	PTOV1	-1.109461993	0.00793785	protein_coding
ENSG00000144589	STK11IP	-1.109074665	0.009380459	protein_coding
ENSG00000158106	RHPN1	-1.107521893	0.009681928	protein_coding
ENSG00000099814	CEP170B	-1.107227887	0.008310346	protein_coding
ENSG00000107404	DVL1	-1.107087227	0.008091372	protein_coding
ENSG00000102109	PCSK1N	-1.106428561	0.008602489	protein_coding
ENSG00000162073	PAQR4	-1.106410557	0.009631756	protein_coding
ENSG00000064545	TMEM161A	-1.105914562	0.008211469	protein_coding
ENSG00000214706	IFRD2	-1.104117571	0.008160716	protein_coding
ENSG00000142409	ZNF787	-1.10282476	0.008882003	protein_coding
ENSG00000123146	ADGRE5	-1.102183412	0.01167373	protein_coding
ENSG00000124507	PACSIN1	-1.102039655	0.039811039	protein_coding
ENSG00000142798	HSPG2	-1.101225772	0.012165774	protein_coding
ENSG00000141933	TPGS1	-1.099941131	0.012838679	protein_coding
ENSG00000163870	TPRA1	-1.099718105	0.009243396	protein_coding
ENSG00000068001	HYAL2	-1.096937865	0.010198881	protein_coding
ENSG00000006062	MAP3K14	-1.094074623	0.021834404	protein_coding
ENSG00000251322	SHANK3	-1.093918743	0.009369883	protein_coding

ENSG00000197119	SLC25A29	-1.093832527	0.012288879	protein_coding
ENSG00000136830	FAM129B	-1.093638255	0.008975579	protein_coding
ENSG00000163794	UCN	-1.092930008	0.037190124	protein_coding
ENSG00000205476	CCDC85C	-1.091656589	0.009288847	protein_coding
ENSG00000203772	SPRN	-1.087528242	0.023682354	protein_coding
ENSG00000105520	PLPPR2	-1.087174573	0.01017802	protein_coding
ENSG00000168273	SMIM4	-1.086550634	0.01327429	protein_coding
ENSG00000181790	ADGRB1	-1.086507865	0.015474095	protein_coding
ENSG00000115844	DLX2	-1.086119602	0.009642413	protein_coding
ENSG00000185522	LMNTD2	-1.085594891	0.0350671	protein_coding
ENSG00000148824	MTG1	-1.085535132	0.014475445	protein_coding
ENSG00000078900	TP73	-1.085102696	0.013120503	protein_coding
ENSG00000006118	TMEM132A	-1.08450056	0.009533045	protein_coding
ENSG00000184470	TXNRD2	-1.084282873	0.009935738	protein_coding
ENSG00000127561	SYNGR3	-1.084228327	0.023339508	protein_coding
ENSG00000125503	PPP1R12C	-1.083919286	0.009637029	protein_coding
ENSG00000164362	TERT	-1.083495075	0.013601213	protein_coding
ENSG00000179403	VWA1	-1.083005369	0.013391952	protein_coding
ENSG00000167703	SLC43A2	-1.082448403	0.014536118	protein_coding
ENSG00000122490	PQLC1	-1.08166423	0.010543425	protein_coding
ENSG00000212864	RNF208	-1.079995165	0.025927384	protein_coding
ENSG00000112667	DNPH1	-1.07998811	0.009658496	protein_coding
ENSG00000197858	GPAA1	-1.076603676	0.00995706	protein_coding
ENSG00000006638	TBXA2R	-1.075422307	0.041706837	protein_coding
ENSG00000115649	CNPPD1	-1.074930779	0.011560094	protein_coding
ENSG00000177700	POLR2L	-1.07292361	0.010246408	protein_coding
ENSG00000176022	B3GALT6	-1.072579955	0.011081681	protein_coding
ENSG00000235034	C19orf81	-1.07220402	0.022061377	protein_coding
ENSG00000011105	TSPAN9	-1.071643551	0.013202403	protein_coding
ENSG00000130762	ARHGEF16	-1.071587149	0.018465063	protein_coding
ENSG00000169991	IFFO2	-1.070771905	0.014763849	protein_coding
ENSG00000167925	GHDC	-1.068787274	0.012678429	protein_coding
ENSG00000197696	NMB	-1.068557268	0.014806683	protein_coding
ENSG00000161395	PGAP3	-1.067295435	0.015165069	protein_coding
ENSG00000103253	HAGHL	-1.067246696	0.014312481	protein_coding
ENSG00000107819	SFXN3	-1.066390618	0.01457207	protein_coding
ENSG00000100299	ARSA	-1.0654519	0.012507969	protein_coding
ENSG00000166823	MESP1	-1.065423217	0.019443105	protein_coding
ENSG00000197114	ZGPAT	-1.063646661	0.023354046	protein_coding
ENSG00000169710	FASN	-1.062512587	0.010471664	protein_coding
ENSG00000099875	MKNK2	-1.059814226	0.011045592	protein_coding
ENSG00000213420	GPC2	-1.059179295	0.031516168	protein_coding
ENSG00000177519	RPRM	-1.059050659	0.049131109	protein_coding

ENSG00000183655	KLHL25	-1.057258888	0.015630023	protein_coding
ENSG00000030110	BAK1	-1.057245671	0.012260039	protein_coding
ENSG00000114395	CYB561D2	-1.056383151	0.017200456	protein_coding
ENSG00000186666	BCDIN3D	-1.056353554	0.025128849	protein_coding
ENSG00000198355	PIM3	-1.056151808	0.011260876	protein_coding
ENSG00000185909	KLHDC8B	-1.056080817	0.016034731	protein_coding
ENSG00000135424	ITGA7	-1.055440394	0.01652734	protein_coding
ENSG00000100445	SDR39U1	-1.055399072	0.016032055	protein_coding
ENSG00000188486	H2AFX	-1.054475596	0.011328536	protein_coding
ENSG00000204580	DDR1	-1.054314437	0.013202044	protein_coding
ENSG00000164403	SHROOM1	-1.054014525	0.015647243	protein_coding
ENSG00000167799	NUDT8	-1.053310377	0.017104255	protein_coding
ENSG00000106009	BRAT1	-1.052897265	0.011687749	protein_coding
ENSG00000070404	FSTL3	-1.052052399	0.021840876	protein_coding
ENSG00000244165	P2RY11	-1.051947982	0.019800385	protein_coding
ENSG00000170684	ZNF296	-1.049314732	0.021381169	protein_coding
ENSG00000179627	ZBTB42	-1.049075174	0.027592909	protein_coding
ENSG00000171858	RPS21	-1.048839689	0.011671488	protein_coding
ENSG00000160963	COL26A1	-1.04861446	0.019432215	protein_coding
ENSG00000061656	SPAG4	-1.04859108	0.036686451	protein_coding
ENSG00000130332	LSM7	-1.048518675	0.011965783	protein_coding
ENSG00000130821	SLC6A8	-1.047844192	0.011804023	protein_coding
ENSG00000177283	FZD8	-1.046259859	0.015669297	protein_coding
ENSG00000224940	PRRT4	-1.045921523	0.017526681	protein_coding
ENSG00000183479	TREX2	-1.045402315	0.034402898	protein_coding
ENSG00000110628	SLC22A18	-1.045402315	0.034402898	protein_coding
ENSG00000152669	CCNO	-1.045290291	0.026127706	protein_coding
ENSG00000083807	SLC27A5	-1.044925202	0.025986633	protein_coding
ENSG00000169258	GPRIN1	-1.044609865	0.013013139	protein_coding
ENSG00000185340	GAS2L1	-1.044569456	0.01835622	protein_coding
ENSG00000160959	LRRC14	-1.044373292	0.013480286	protein_coding
ENSG00000176182	MYPOP	-1.044252973	0.015995594	protein_coding
ENSG00000131409	LRRC4B	-1.043657156	0.017398061	protein_coding
ENSG00000155034	FBXL18	-1.043641946	0.015991852	protein_coding
ENSG00000115457	IGFBP2	-1.043502869	0.012791772	protein_coding
ENSG00000254858	MPV17L2	-1.043421244	0.014124514	protein_coding
ENSG00000125912	NCLN	-1.043387974	0.012154716	protein_coding
ENSG00000137409	MTCH1	-1.04300886	0.012224782	protein_coding
ENSG00000161513	FDXR	-1.042963196	0.014151678	protein_coding
ENSG00000079313	REXO1	-1.040572172	0.013349203	protein_coding
ENSG00000247596	TWF2	-1.038500092	0.014170767	protein_coding
ENSG00000126464	PRR12	-1.038410419	0.01353717	protein_coding
ENSG00000099364	FBXL19	-1.037516877	0.012776929	protein_coding

ENSG00000275183	LENG9	-1.037296299	0.028677061	protein_coding
ENSG00000171119	NRTN	-1.037180584	0.021301892	protein_coding
ENSG00000178252	WDR6	-1.036767031	0.012672682	protein_coding
ENSG00000074071	MRPS34	-1.035440619	0.012780927	protein_coding
ENSG00000075399	VPS9D1	-1.035413536	0.017519473	protein_coding
ENSG00000224420	ADM5	-1.034923812	0.049644069	protein_coding
ENSG00000135454	B4GALNT1	-1.034730982	0.018352883	protein_coding
ENSG00000168890	TMEM150A	-1.034363879	0.024191052	protein_coding
ENSG00000136840	ST6GALNAC4	-1.034300578	0.015294333	protein_coding
ENSG00000107140	TESK1	-1.033200688	0.018129142	protein_coding
ENSG00000004777	ARHGAP33	-1.031883086	0.022930178	protein_coding
ENSG00000153976	HS3ST3A1	-1.031563586	0.017887715	protein_coding
ENSG00000188070	C11orf95	-1.031311267	0.01419527	protein_coding
ENSG00000100968	NFATC4	-1.029685362	0.015713248	protein_coding
ENSG00000171813	PWWP2B	-1.029591234	0.01867871	protein_coding
ENSG00000164167	LSM6	-1.028752577	0.018477718	protein_coding
ENSG00000079999	KEAP1	-1.02801019	0.013882969	protein_coding
ENSG00000071655	MBD3	-1.027590555	0.013656087	protein_coding
ENSG00000269113	TRABD2B	-1.025848157	0.02872419	protein_coding
ENSG00000118046	STK11	-1.025714462	0.013790837	protein_coding
ENSG00000166016	ABTB2	-1.025253184	0.01677698	protein_coding
ENSG00000160072	ATAD3B	-1.025016335	0.014189747	protein_coding
ENSG00000095596	CYP26A1	-1.024003486	0.02362233	protein_coding
ENSG00000140983	RHOT2	-1.023998126	0.014011891	protein_coding
ENSG00000188747	NOXA1	-1.023013114	0.022365683	protein_coding
ENSG00000280071	GATD3B	-1.023013114	0.022365683	protein_coding
ENSG00000185085	INTS5	-1.02184121	0.015580902	protein_coding
ENSG00000198816	ZNF358	-1.021615551	0.016583931	protein_coding
ENSG00000076826	CAMSAP3	-1.019698204	0.016932778	protein_coding
ENSG00000187961	KLHL17	-1.019065028	0.01606264	protein_coding
ENSG00000153443	UBALD1	-1.017439117	0.015861772	protein_coding
ENSG00000076351	SLC46A1	-1.017106112	0.019845673	protein_coding
ENSG00000105516	DBP	-1.017039197	0.020413161	protein_coding
ENSG00000134072	CAMK1	-1.016942058	0.021241841	protein_coding
ENSG00000136352	NKX2-1	-1.016646668	0.02484791	protein_coding
ENSG00000100258	LMF2	-1.016299085	0.014928976	protein_coding
ENSG00000156453	PCDH1	-1.016169614	0.033356057	protein_coding
ENSG00000166289	PLEKHF1	-1.016099268	0.034333329	protein_coding
ENSG00000283154	IQCJ-SCHIP1	-1.016048403	0.035045218	protein_coding
ENSG00000182749	PAQR7	-1.016021657	0.035421272	protein_coding
ENSG00000187479	C11orf96	-1.015367818	0.049024715	protein_coding
ENSG00000103152	MPG	-1.015250925	0.017226587	protein_coding
ENSG00000160285	LSS	-1.014158794	0.015072296	protein_coding

ENSG00000008441	NFIX	-1.013996594	0.016592381	protein_coding
ENSG00000166886	NAB2	-1.013985595	0.019102624	protein_coding
ENSG00000164880	INTS1	-1.013575006	0.014707962	protein_coding
ENSG00000127399	LRRC61	-1.013070156	0.017489674	protein_coding
ENSG00000161091	MFSD12	-1.012122457	0.015738784	protein_coding
ENSG00000171222	SCAND1	-1.01126733	0.016238236	protein_coding
ENSG00000187051	RPS19BP1	-1.010929883	0.015375822	protein_coding
ENSG00000177697	CD151	-1.009337618	0.015530114	protein_coding
ENSG00000007264	MATK	-1.009192098	0.016374912	protein_coding
ENSG00000189319	FAM53B	-1.009112035	0.016880043	protein_coding
ENSG00000140859	KIFC3	-1.008863161	0.021086073	protein_coding
ENSG00000168101	NUDT16L1	-1.008242618	0.018106369	protein_coding
ENSG00000101199	ARFGAP1	-1.006827511	0.015676531	protein_coding
ENSG00000162062	TEDC2	-1.00674735	0.016981717	protein_coding
ENSG00000126106	TMEM53	-1.00579038	0.033808665	protein_coding
ENSG00000095906	NUBP2	-1.005594418	0.015873931	protein_coding
ENSG00000139546	TARBP2	-1.004799129	0.01691636	protein_coding
ENSG00000184207	PGP	-1.004262785	0.016253828	protein_coding
ENSG00000099904	ZDHHC8	-1.003773648	0.016394883	protein_coding
ENSG00000104964	AES	-1.003528004	0.016383561	protein_coding
ENSG00000184489	PTP4A3	-1.001070576	0.02197215	protein_coding
ENSG00000075618	FSCN1	-1.000467282	0.016182601	protein_coding
ENSG00000064225	ST3GAL6	1.002125332	0.021513207	protein_coding
ENSG00000176209	SMIM19	1.002820603	0.020662192	protein_coding
ENSG00000106052	TAX1BP1	1.003350983	0.016357959	protein_coding
ENSG00000113387	SUB1	1.003679111	0.015781879	protein_coding
ENSG00000120860	WASHC3	1.005956237	0.022717051	protein_coding
ENSG00000256294	ZNF225	1.008146465	0.038011217	protein_coding
ENSG00000163634	THOC7	1.010125488	0.015825052	protein_coding
ENSG00000003436	TFPI	1.010851687	0.040725658	protein_coding
ENSG00000197603	CPLANE1	1.011476041	0.018533563	protein_coding
ENSG00000139117	CPNE8	1.012555754	0.019312824	protein_coding
ENSG00000147118	ZNF182	1.012988896	0.022602575	protein_coding
ENSG00000151422	FER	1.019438059	0.018534001	protein_coding
ENSG00000173230	GOLGB1	1.020508188	0.014545221	protein_coding
ENSG00000123595	RAB9A	1.021980013	0.015581262	protein_coding
ENSG00000166200	COPS2	1.022981504	0.014545666	protein_coding
ENSG00000082146	STRADB	1.025456938	0.014679274	protein_coding
ENSG00000116030	SUMO1	1.025978179	0.013825109	protein_coding
ENSG00000116044	NFE2L2	1.028844384	0.014570899	protein_coding
ENSG00000180574	EIF2S3B	1.030030917	0.019682578	protein_coding
ENSG00000205571	SMN2	1.030777412	0.016989336	protein_coding
ENSG00000140905	GCSH	1.032836103	0.016631176	protein_coding

ENSG00000274276	CBSL	1.037709075	0.013856152	protein_coding
ENSG00000153132	CLGN	1.039931376	0.016370148	protein_coding
ENSG00000087502	ERGIC2	1.044495053	0.013815891	protein_coding
ENSG00000162994	CLHC1	1.045838437	0.036686451	protein_coding
ENSG00000132357	CARD6	1.047311088	0.037209159	protein_coding
ENSG00000152404	CWF19L2	1.047925114	0.015657741	protein_coding
ENSG00000130021	PUDP	1.048266881	0.01897787	protein_coding
ENSG00000206530	CFAP44	1.048630489	0.020379833	protein_coding
ENSG00000031691	CENPQ	1.051554678	0.018284874	protein_coding
ENSG00000117262	GPR89A	1.051717846	0.016984613	protein_coding
ENSG00000118292	C1orf54	1.05217005	0.035558915	protein_coding
ENSG00000104412	EMC2	1.053344992	0.013502186	protein_coding
ENSG00000174720	LARP7	1.057243709	0.01234026	protein_coding
ENSG00000274419	TBC1D3D	1.058331384	0.032924312	protein_coding
ENSG00000159256	MORC3	1.063368935	0.013650699	protein_coding
ENSG00000138433	CIR1	1.064968766	0.013574352	protein_coding
ENSG00000083535	PIBF1	1.06547327	0.012166632	protein_coding
ENSG00000153975	ZUP1	1.070118607	0.012501753	protein_coding
ENSG00000164221	CCDC112	1.072420204	0.014410862	protein_coding
ENSG00000276180	HIST1H4I	1.075221917	0.013772335	protein_coding
ENSG00000087495	PHACTR3	1.075439238	0.023682354	protein_coding
ENSG00000141642	ELAC1	1.075452996	0.019938131	protein_coding
ENSG00000133112	TPT1	1.076357444	0.009524794	protein_coding
ENSG00000151743	AMN1	1.077503532	0.030996237	protein_coding
ENSG00000163029	SMC6	1.084765552	0.009880827	protein_coding
ENSG00000240344	PPIL3	1.089349603	0.011595878	protein_coding
ENSG00000135205	CCDC146	1.089633458	0.046223025	protein_coding
ENSG00000151366	NDUFC2	1.093508997	0.009705878	protein_coding
ENSG00000135968	GCC2	1.093550485	0.009524648	protein_coding
ENSG00000127914	AKAP9	1.093930072	0.009651232	protein_coding
ENSG00000133710	SPINK5	1.095889298	0.025248005	protein_coding
ENSG00000175354	PTPN2	1.096067149	0.01078456	protein_coding
ENSG00000129534	MIS18BP1	1.09900599	0.009317663	protein_coding
ENSG00000198839	ZNF277	1.100440522	0.010736504	protein_coding
ENSG00000095261	PSMD5	1.100872242	0.014420139	protein_coding
ENSG00000137821	LRRC49	1.109735173	0.013357164	protein_coding
ENSG00000166896	ATP23	1.111968492	0.014749201	protein_coding
ENSG00000166192	SENP8	1.116904189	0.015229158	protein_coding
ENSG00000107890	ANKRD26	1.122639199	0.010594506	protein_coding
ENSG00000153140	CETN3	1.128302951	0.00879816	protein_coding
ENSG00000096872	IFT74	1.129492403	0.009787573	protein_coding
ENSG00000139618	BRCA2	1.129622886	0.007593524	protein_coding
ENSG00000174718	KIAA1551	1.130030204	0.009642512	protein_coding

ENSG00000147687	TATDN1	1.130363564	0.009112864	protein_coding
ENSG00000137500	CCDC90B	1.131408886	0.008237673	protein_coding
ENSG00000140386	SCAPER	1.141533345	0.008745638	protein_coding
ENSG00000122008	POLK	1.142182591	0.008466081	protein_coding
ENSG00000135336	ORC3	1.145042464	0.006804293	protein_coding
ENSG00000131013	PPIL4	1.145316217	0.006476972	protein_coding
ENSG00000130649	CYP2E1	1.148341896	0.014188412	protein_coding
ENSG00000168679	SLC16A4	1.149590822	0.022901013	protein_coding
ENSG00000020426	MNAT1	1.15294259	0.006762291	protein_coding
ENSG00000224578	HNRNPA1P48	1.152971568	0.006861736	protein_coding
ENSG00000164162	ANAPC10	1.153366035	0.014959466	protein_coding
ENSG00000137831	UACA	1.153851622	0.006121945	protein_coding
ENSG00000169621	APLF	1.154078416	0.023030426	protein_coding
ENSG00000090612	ZNF268	1.154884185	0.008756328	protein_coding
ENSG00000143971	ETAA1	1.165146822	0.006804991	protein_coding
ENSG00000170860	LSM3	1.169165152	0.005168978	protein_coding
ENSG00000106013	ANKRD7	1.170307959	0.038817849	protein_coding
ENSG00000056277	ZNF280C	1.172446482	0.005507475	protein_coding
ENSG00000100629	CEP128	1.17393847	0.007140174	protein_coding
ENSG00000117477	CCDC181	1.174364008	0.007733495	protein_coding
ENSG00000101871	MID1	1.176854681	0.00497534	protein_coding
ENSG00000198707	CEP290	1.180101605	0.005483223	protein_coding
ENSG00000133640	LRRIQ1	1.182403675	0.032606579	protein_coding
ENSG00000176204	LRRTM4	1.18627475	0.018765093	protein_coding
ENSG00000118514	ALDH8A1	1.190820547	0.033121091	protein_coding
ENSG00000233087	RAB6D	1.192495751	0.027887688	protein_coding
ENSG00000196172	ZNF681	1.192495751	0.027887688	protein_coding
ENSG00000111832	RWDD1	1.193649445	0.004442718	protein_coding
ENSG00000109063	MYH3	1.193848379	0.014618718	protein_coding
ENSG00000188452	CERKL	1.198364955	0.009728465	protein_coding
ENSG00000180336	MEIOC	1.199180327	0.041535125	protein_coding
ENSG00000151490	PTPRO	1.199180327	0.041535125	protein_coding
ENSG00000164414	SLC35A1	1.203321443	0.009547575	protein_coding
ENSG00000152422	XRCC4	1.207985909	0.007816474	protein_coding
ENSG00000196873	CBWD3	1.208568834	0.008913313	protein_coding
ENSG00000185829	ARL17A	1.208787203	0.006610262	protein_coding
ENSG00000138311	ZNF365	1.217104898	0.024563218	protein_coding
ENSG00000251192	ZNF674	1.217613777	0.010563916	protein_coding
ENSG00000166750	SLFN5	1.220443385	0.008238935	protein_coding
ENSG00000241399	CD302	1.221545742	0.010492033	protein_coding
ENSG00000152932	RAB3C	1.228491768	0.009862954	protein_coding
ENSG00000100815	TRIP11	1.230248253	0.003908673	protein_coding
ENSG00000165169	DYNLT3	1.231338491	0.004195302	protein_coding

ENSG00000179021	C3orf38	1.236465113	0.004078934	protein_coding
ENSG00000196912	ANKRD36B	1.238707351	0.006940806	protein_coding
ENSG00000165512	ZNF22	1.245860873	0.003597623	protein_coding
ENSG00000205707	ETFRF1	1.246745553	0.00570038	protein_coding
ENSG00000134198	TSPAN2	1.251908257	0.008196656	protein_coding
ENSG00000084453	SLCO1A2	1.25256836	0.029914245	protein_coding
ENSG00000166352	C11orf74	1.252686533	0.00405002	protein_coding
ENSG00000171817	ZNF540	1.255086126	0.046163285	protein_coding
ENSG00000138380	CARF	1.260376354	0.009278173	protein_coding
ENSG00000147041	SYTL5	1.260848891	0.007128095	protein_coding
ENSG00000168944	CEP120	1.261343538	0.003245518	protein_coding
ENSG00000176971	FIBIN	1.265098571	0.019047235	protein_coding
ENSG00000173210	ABLIM3	1.266098735	0.030298791	protein_coding
ENSG00000146757	ZNF92	1.266330762	0.003803657	protein_coding
ENSG00000163141	BNIPL	1.27389492	0.04754413	protein_coding
ENSG00000166669	ATF7IP2	1.274114308	0.005833977	protein_coding
ENSG00000180479	ZNF571	1.286278989	0.010719151	protein_coding
ENSG00000204869	IGFL4	1.296754387	0.038410682	protein_coding
ENSG00000158055	GRHL3	1.297695698	0.031103696	protein_coding
ENSG00000001561	ENPP4	1.299573999	0.003228083	protein_coding
ENSG00000113396	SLC27A6	1.301001795	0.01112167	protein_coding
ENSG00000152582	SPEF2	1.301791612	0.008570113	protein_coding
ENSG00000129991	TNNI3	1.302107826	0.007273056	protein_coding
ENSG00000163319	MRPS18C	1.316048883	0.002076061	protein_coding
ENSG00000072201	LNX1	1.31630161	0.031520396	protein_coding
ENSG00000176046	NUPR1	1.332235601	0.004030262	protein_coding
ENSG00000243696	AC006254.1	1.337252882	0.031942348	protein_coding
ENSG00000143248	RGS5	1.342385654	0.006611351	protein_coding
ENSG00000135315	CEP162	1.343408101	0.002637051	protein_coding
ENSG00000172086	KRCC1	1.348902817	0.003337939	protein_coding
ENSG00000175197	DDIT3	1.355731974	0.003676385	protein_coding
ENSG00000115594	IL1R1	1.360993488	0.018835279	protein_coding
ENSG00000172159	FRMD3	1.372547878	0.016215654	protein_coding
ENSG00000147082	CCNB3	1.378530467	0.0100741	protein_coding
ENSG00000169627	BOLA2B	1.384772588	0.011094044	protein_coding
ENSG00000101132	PFDN4	1.388824901	0.001042569	protein_coding
ENSG00000223658	C1GALT1C1L	1.391286925	0.01607015	protein_coding
ENSG00000188611	ASAH2	1.409284709	0.005307868	protein_coding
ENSG00000168491	CCDC110	1.414991035	0.022064739	protein_coding
ENSG00000183137	CEP57L1	1.415623661	0.001179573	protein_coding
ENSG00000187554	TLR5	1.443789307	0.042814164	protein_coding
ENSG00000133739	LRRCC1	1.448803431	0.001226946	protein_coding
ENSG00000219626	FAM228B	1.450029804	0.011696955	protein_coding

ENSG00000175564	UCP3	1.450029804	0.011696955	protein_coding
ENSG00000197580	BCO2	1.456476784	0.033533086	protein_coding
ENSG00000089199	CHGB	1.456476784	0.033533086	protein_coding
ENSG00000070081	NUCB2	1.458130072	0.000625575	protein_coding
ENSG00000170542	SERPINB9	1.462126846	0.009030878	protein_coding
ENSG00000109452	INPP4B	1.473167477	0.011440165	protein_coding
ENSG00000118557	PMFBP1	1.475365094	0.021872151	protein_coding
ENSG00000185267	CDNF	1.506474109	0.008515683	protein_coding
ENSG00000163666	HESX1	1.517033355	0.017860441	protein_coding
ENSG00000197405	C5AR1	1.517033355	0.017860441	protein_coding
ENSG00000145700	ANKRD31	1.527156627	0.010850785	protein_coding
ENSG00000240038	AMY2B	1.533889169	0.007267388	protein_coding
ENSG00000106686	SPATA6L	1.53565874	0.006465915	protein_coding
ENSG00000086717	PPEF1	1.550593732	0.044489089	protein_coding
ENSG00000196267	ZNF836	1.552668053	0.034035355	protein_coding
ENSG00000060140	STYK1	1.556645043	0.017539069	protein_coding
ENSG00000129204	USP6	1.591108179	0.007606008	protein_coding
ENSG00000198075	SULT1C4	1.605645375	0.001503329	protein_coding
ENSG00000182459	TEX19	1.625312966	0.045117465	protein_coding
ENSG00000118307	CASC1	1.625312966	0.045117465	protein_coding
ENSG00000118997	DNAH7	1.625312966	0.045117465	protein_coding
ENSG00000164989	CCDC171	1.627404741	0.000784384	protein_coding
ENSG00000197279	ZNF165	1.631692615	0.003215074	protein_coding
ENSG00000128052	KDR	1.681091252	0.026105331	protein_coding
ENSG00000135346	CGA	1.681091252	0.026105331	protein_coding
ENSG00000170006	TMEM154	1.698839143	0.033910222	protein_coding
ENSG00000267508	ZNF285	1.698839143	0.033910222	protein_coding
ENSG00000075673	ATP12A	1.705619684	0.013149317	protein_coding
ENSG00000122477	LRRC39	1.713443113	0.00168007	protein_coding
ENSG00000188738	FSIP2	1.720204879	0.001306375	protein_coding
ENSG00000129749	CHRNA10	1.721112036	0.007498916	protein_coding
ENSG00000264343	NOTCH2NLA	1.723156814	0.045435959	protein_coding
ENSG00000274290	HIST1H2BE	1.723156814	0.045435959	protein_coding
ENSG00000119888	EPCAM	1.731785431	0.004688294	protein_coding
ENSG00000114638	UPK1B	1.735445856	0.008764824	protein_coding
ENSG00000123689	G0S2	1.75189985	0.000137656	protein_coding
ENSG00000172803	SNX32	1.798352624	0.008204261	protein_coding
ENSG00000226763	SRRM5	1.805030277	0.033376343	protein_coding
ENSG00000136206	SPDYE1	1.805030277	0.033376343	protein_coding
ENSG00000183346	CABCOCO1	1.805030277	0.033376343	protein_coding
ENSG00000137101	CD72	1.813053776	0.00355334	protein_coding
ENSG00000162526	TSSK3	1.824389332	0.009730816	protein_coding
ENSG00000183161	FANCF	1.835522874	0.019137366	protein_coding

ENSG00000241106	HLA-DOB	1.84257559	0.00648527	protein_coding
ENSG00000104848	KCNA7	1.857206395	0.045202178	protein_coding
ENSG00000125851	PCSK2	1.857206395	0.045202178	protein_coding
ENSG00000165621	OXGR1	1.87309127	0.007582043	protein_coding
ENSG00000172264	MACROD2	1.874198558	0.001315159	protein_coding
ENSG00000197980	LEKR1	1.882505856	0.024510598	protein_coding
ENSG00000276600	RAB7B	1.882505856	0.024510598	protein_coding
ENSG00000222014	RAB6C	1.895350766	0.003605466	protein_coding
ENSG00000171121	KCNMB3	1.899296634	0.014380224	protein_coding
ENSG00000203872	C6orf163	1.920202692	0.005911861	protein_coding
ENSG00000106804	C5	1.932703443	0.002892433	protein_coding
ENSG00000100362	PVALB	1.949565903	0.032292239	protein_coding
ENSG00000206384	COL6A6	1.949565903	0.032292239	protein_coding
ENSG00000148942	SLC5A12	1.965824209	0.004613369	protein_coding
ENSG00000196335	STK31	1.972160805	0.001027717	protein_coding
ENSG00000120215	MLANA	2.036366746	0.023070854	protein_coding
ENSG00000275895	U2AF1L5	2.041568501	4.75E-06	protein_coding
ENSG00000172062	SMN1	2.047432769	2.87E-06	protein_coding
ENSG00000188107	EYS	2.053223049	0.043991311	protein_coding
ENSG00000164100	NDST3	2.053223049	0.043991311	protein_coding
ENSG00000169548	ZNF280A	2.053223049	0.043991311	protein_coding
ENSG00000104112	SCG3	2.056872743	0.000243475	protein_coding
ENSG00000256591	AP003108.2	2.092715764	0.009732694	protein_coding
ENSG00000242220	TCP10L	2.156489525	0.007167784	protein_coding
ENSG00000249437	NAIP	2.15915056	0.030348148	protein_coding
ENSG00000184113	CLDN5	2.15915056	0.030348148	protein_coding
ENSG00000226650	KIF4B	2.15915056	0.030348148	protein_coding
ENSG00000145920	CPLX2	2.15915056	0.030348148	protein_coding
ENSG00000152822	GRM1	2.15915056	0.030348148	protein_coding
ENSG00000230667	SETSIP	2.257829686	0.020952111	protein_coding
ENSG00000005102	MEOX1	2.257829686	0.020952111	protein_coding
ENSG00000196747	HIST1H2AI	2.257829686	0.020952111	protein_coding
ENSG00000092200	RPGRIP1	2.28662232	0.000660415	protein_coding
ENSG00000154269	ENPP3	2.339201936	0.006072876	protein_coding
ENSG00000100191	SLC5A4	2.350189193	0.014485426	protein_coding
ENSG00000153165	RGPD3	2.371285094	0.041032031	protein_coding
ENSG00000165917	RAPSN	2.371285094	0.041032031	protein_coding
ENSG00000124678	TCP11	2.371285094	0.041032031	protein_coding
ENSG00000112706	IMPG1	2.371285094	0.041032031	protein_coding
ENSG00000189058	APOD	2.371285094	0.041032031	protein_coding
ENSG00000171522	PTGER4	2.405925696	0.004368548	protein_coding
ENSG00000254852	NPIPA2	2.436990037	0.010033723	protein_coding
ENSG00000100298	APOBEC3H	2.469699457	0.003149424	protein_coding

ENSG00000132639	SNAP25	2.5188635	0.006966163	protein_coding
ENSG00000183889	PKD1P1	2.576365131	2.97E-05	protein_coding
ENSG00000120440	TTLL2	2.585097769	0.000660977	protein_coding
ENSG00000081041	CXCL2	2.715709624	0.011832427	protein_coding
ENSG00000124091	GCNT7	2.715709624	0.011832427	protein_coding
ENSG00000197153	HIST1H3J	2.715709624	0.011832427	protein_coding
ENSG00000154263	ABCA10	2.739825227	0.002370371	protein_coding
ENSG00000166278	C2	2.806548987	0.001665133	protein_coding
ENSG00000127364	TAS2R4	2.81438875	0.007864773	protein_coding
ENSG00000276966	HIST1H4E	2.81438875	0.007864773	protein_coding
ENSG00000169393	ELSPBP1	2.993549101	0.003515158	protein_coding
ENSG00000187944	C2orf66	3.003396154	0.034866602	protein_coding
ENSG00000083814	ZNF671	3.003396154	0.034866602	protein_coding
ENSG00000182916	TCEAL7	3.003396154	0.034866602	protein_coding
ENSG00000249156	TAF11L12	3.003396154	0.034866602	protein_coding
ENSG00000205572	SERF1B	3.061832655	1.02E-10	protein_coding
ENSG00000104435	STMN2	3.075422564	0.002364823	protein_coding
ENSG00000187243	MAGED4B	3.116888107	3.93E-07	protein_coding
ENSG00000151164	RAD9B	3.152898143	0.001597933	protein_coding
ENSG00000267710	EDDM13	3.152898143	0.001597933	protein_coding
ENSG00000250673	REELD1	3.153409692	0.021566548	protein_coding
ENSG00000186453	FAM228A	3.153409692	0.021566548	protein_coding
ENSG00000140093	SERPINA10	3.251819569	0.000111734	protein_coding
ENSG00000198939	ZFP2	3.289283354	0.013412089	protein_coding
ENSG00000124334	IL9R	3.289283354	0.013412089	protein_coding
ENSG00000123338	NCKAP1L	3.289283354	0.013412089	protein_coding
ENSG00000146674	IGFBP3	3.41345461	0.008390282	protein_coding
ENSG00000187398	LUZP2	3.41345461	0.008390282	protein_coding
ENSG00000127507	ADGRE2	3.41345461	0.008390282	protein_coding
ENSG00000078596	ITM2A	3.60285402	0.000116677	protein_coding
ENSG00000102271	KLHL4	5.068848838	1.08E-13	protein_coding
ENSG00000000005	TNMD	5.277000344	5.34E-08	protein_coding
ENSG00000176358	TAC4	5.606115201	0.040964585	protein_coding
ENSG00000166869	CHP2	5.606115201	0.040964585	protein_coding
ENSG00000169302	STK32A	5.606115201	0.040964585	protein_coding
ENSG00000168404	MLKL	5.606115201	0.040964585	protein_coding
ENSG00000261582	AL121753.1	5.606115201	0.040964585	protein_coding
ENSG00000184995	IFNE	5.606115201	0.040964585	protein_coding
ENSG00000179873	NLRP11	5.606115201	0.040964585	protein_coding
ENSG00000168843	FSTL5	5.606115201	0.040964585	protein_coding
ENSG00000078579	FGF20	5.606115201	0.040964585	protein_coding
ENSG00000262576	PCDHGA4	5.606115201	0.040964585	protein_coding
ENSG00000178460	MCMDC2	5.824270165	0.022676824	protein_coding

ENSG00000147613	PSKH2	5.824270165	0.022676824	protein_coding
ENSG00000101842	VSIG1	5.824270165	0.022676824	protein_coding
ENSG00000126838	PZP	5.824270165	0.022676824	protein_coding
ENSG00000138356	AOX1	5.824270165	0.022676824	protein_coding
ENSG00000185955	C7orf61	5.824270165	0.022676824	protein_coding
ENSG00000149970	CNKSR2	6.013728963	0.012730848	protein_coding
ENSG00000004948	CALCR	6.013728963	0.012730848	protein_coding
ENSG00000010932	FMO1	6.013728963	0.012730848	protein_coding
ENSG00000162843	WDR64	6.013728963	0.012730848	protein_coding
ENSG00000124343	XG	6.013728963	0.012730848	protein_coding
ENSG00000183378	OVCH2	6.013728963	0.012730848	protein_coding
ENSG00000213714	FAM209B	6.181170872	0.007243414	protein_coding
ENSG00000172717	FAM71D	6.181170872	0.007243414	protein_coding
ENSG00000230561	CCDC192	6.181170872	0.007243414	protein_coding
ENSG00000188037	CLCN1	6.181170872	0.007243414	protein_coding
ENSG00000050628	PTGER3	6.331184409	0.004174171	protein_coding
ENSG00000204287	HLA-DRA	6.331184409	0.004174171	protein_coding
ENSG00000163705	FANCD2OS	6.331184409	0.004174171	protein_coding
ENSG00000175426	PCSK1	6.591229327	0.00143701	protein_coding
ENSG00000001626	CFTR	6.811482601	0.000517265	protein_coding

**Commonly changed genes**
**Gene name**
SPSB3
AL121845.3
TEN1-CDK3
HGFAC
ACTL10
MAGED4
TMEFF1
PDXP
SMIM11A
MGAT2
GREM2
TRAPPC5
TMSB15B
SLC22A31
CYP51A1
TMEM160
ATP6V0C
ALDOA
GPX2
C22orf15
PNMT
TSPO
PRR35
MADCAM1
GPS2
ROM1
MRPL12
LAT
NPAS3
C4orf48
DPEP1
AC020915.1
CTXN1
GOLGA8K
FES
CHADL
RABAC1
COX6A1
ALKBH7
SHISA8

SAC3D1
GNG13
SWSAP1
CKMT2
NME3
NDUFA11
MCRIP1
TRPV2
CITED4
H1FX
CYBA
HAPLN2
LAGE3
HES4
PRR19
EMID1
NUTM2G
CBARP
ARC
CSDC2
AC093512.2
MEGF6
RTEL1-TNFRSF6B
CFAP157
FAM173A
COX8A
RAC3
KLF16
ZNF428
C8orf82
CNIH2
ADAM8
PLK5
VKORC1
PRSS53
ILVBL
SRPK3
AL135905.2
GNG5
EPPK1
PRR22
ZDHHC12

NBL1
FAM43B
TMEM256
MYOF
F12
ESX1
KIFC2
MAMDC4
ZNF524
PLEKHA4
WDR90
ZNF503
ACADS
TP53I13
DALRD3
IFI6
TMEM198
NPDC1
VPS37D
TNXB
IRF2BP1
CFAP410
CTU1
ICAM5
SOX15
ZBTB16
MRPL23
LRRC56
LIN37
SCAF1
CITED1
ALDH16A1
COMTD1
TENM1
C6orf48
ZMYND10
STIMATE
NDUFB2
NAPRT
MXD3
BCL7C
UQCC3
SNRPB

KIF26B
ARMC5
TMEM121
MOSPD3
SAMD11
MFSD10
BX255925.3
PTOV1
STK11IP
PCSK1N
TPGS1
HYAL2
UCN
SMIM4
POLR2L
GHDC
NMB
PGAP3
ZGPAT
CYB561D2
SDR39U1
H2AFX
NUDT8
P2RY11
SPAG4
TREX2
SLC22A18
CCNO
GAS2L1
TWF2
NRTN
TMEM150A
NFATC4
RHOT2
UBALD1
DBP
NUBP2
SMIM19
CPLANE1
EIF2S3B
CARD6
CFAP44
GPR89A

C1orf54
TBC1D3D
MORC3
TPT1
GCC2
AKAP9
MIS18BP1
ANKRD26
BRCA2
KIAA1551
POLK
ANAPC10
CEP290
SLFN5
TRIP11
ANKRD36B
SYTL5
SLC27A6
SERPINB9
USP6
FSIP2
SPDYE1
COL6A6
MLANA
EYS
KIF4B
SERF1B
MAGED4B
EDDM13
TNMD
CNKSR2
CFTR
Gene name
SPSB3
AL121845.3
TEN1-CDK3
HGFAC
ACTL10
MAGED4
TMEFF1
PDXP
SMIM11A
MGAT2

GREM2
TRAPPC5
TMSB15B
SLC22A31
CYP51A1
TMEM160
ATP6V0C
ALDOA
GPX2
C22orf15
PNMT
TSPO
PRR35
MADCAM1
GPS2
ROM1
MRPL12
LAT
NPAS3
C4orf48
DPEP1
AC020915.1
CTXN1
GOLGA8K
FES
CHADL
RABAC1
COX6A1
ALKBH7
SHISA8
SAC3D1
GNG13
SWSAP1
CKMT2
NME3
NDUFA11
MCRIP1
TRPV2
CITED4
H1FX
CYBA
HAPLN2
LAGE3

HES4
PRR19
EMID1
NUTM2G
CBARP
ARC
CSDC2
AC093512.2
MEGF6
RTEL1-TNFRSF6B
CFAP157
FAM173A
COX8A
RAC3
KLF16
ZNF428
C8orf82
CNIH2
ADAM8
PLK5
VKORC1
PRSS53
ILVBL
SRPK3
AL135905.2
GNG5
EPPK1
PRR22
ZDHHC12
NBL1
FAM43B
TMEM256
MYOF
F12
ESX1
KIFC2
MAMDC4
ZNF524
PLEKHA4
WDR90
ZNF503
ACADS

TP53I13
DALRD3
IFI6
TMEM198
NPDC1
VPS37D
TNXB
IRF2BP1
CFAP410
CTU1
ICAM5
SOX15
ZBTB16
MRPL23
LRRC56
LIN37
SCAF1
CITED1
ALDH16A1
COMTD1
TENM1
C6orf48
ZMYND10
STIMATE
NDUFB2
NAPRT
MXD3
BCL7C
UQCC3
SNRPB
KIF26B
ARMC5
TMEM121
MOSPD3
SAMD11
MFSD10
BX255925.3
PTOV1
STK11IP
PCSK1N
TPGS1
HYAL2
UCN

SMIM4
POLR2L
GHDC
NMB
PGAP3
ZGPAT
CYB561D2
SDR39U1
H2AFX
NUDT8
P2RY11
SPAG4
TREX2
SLC22A18
CCNO
GAS2L1
TWF2
NRTN
TMEM150A
NFATC4
RHOT2
UBALD1
DBP
NUBP2
SMIM19
CPLANE1
EIF2S3B
CARD6
CFAP44
GPR89A
C1orf54
TBC1D3D
MORC3
TPT1
GCC2
AKAP9
MIS18BP1
ANKRD26
BRCA2
KIAA1551
POLK
ANAPC10
CEP290

SLFN5
TRIP11
ANKRD36B
SYTL5
SLC27A6
SERPINB9
USP6
FSIP2
SPDYE1
COL6A6
MLANA
EYS
KIF4B
SERF1B
MAGED4B
EDDM13
TNMD
CNKSR2
CFTR
