# Supplementary material for: ML216 Prevents DNA Damage-Induced Senescence by Modulating DBC1–BLM Interaction
Source: Cells. 2022 Dec 29;12(1):145. doi: 10.3390/cells12010145 (PMC9818470; doi:10.3390/cells12010145)
Supplement: Supplementary file 1 [file cells-12-00145-s001.zip › cells-2034577-supplementary Table S2.pdf]

gene name	forward sequence	reverse sequence
p21 (human)	GACACCACTGGAGGGTGACT	CAGGTCCACATGGTCTTCCT
p16 (human)	CCAACGCACCGAATAGTTACG	GCGCTGCCCATCATCATG
cxcl1 (human)	GAAAGCTTGCCTCAATCCTG	CACCAGTGAGCTTCCTCCTC
cxcl2 (human)	AACTGCGCTGCCAGTGCT	CCCATTCTTGAGTGTGGCTA
IL-6 (human)	CCGGGAACGAAAGAGAAGCT	GCGCTTGTGGAGAAGGAGTT
IL-8 (human)	CTTTCCACCCCAAATTTATCAAAG	CAGACAGAGCTCTCTTCCATCAGA
MMP-2 (human)	CCCACTGCGGTTTTCTCGAAT	CAAAGGGGTATCCATCGCCAT
MMP-9 (human)	GCCACTACTGTGCCTTTGAGTC	CCCTCAGAGAATCGCCAGTACT
MMP-3 (human)	ACTCACAGACCTGACTCGGTT	AAGCAGGATCACAGTTGGCTGG
MMP-12 (human)	GATGCTGTCACTACCGTGGGAA	CAATGCCAGATGGCAAGGTTGG
TIMP1 (human)	GGAGAGTGTCTGCGGATACTTC	GCAGGTAGTGATGTGCAAGAGTC
IL-1α (human)	TGTATGTGACTGCCCAAGATGAAG	AGAGGAGGTTGGTCTCACTACC
cyclinD1 (human)	TCTACACCGACAACTCCATCCG	TCTGGCATTTTGGAGAGGAAGTG
cyclinE (human)	TGTGTCCTGGATGTTGACTGCC	CTCTATGTCGCACCACTGATACC
18sRNA (human)	GCTTAATTTGACTCAACACGGGA	AGCTATCAATCTGTCAATCCTGTC
DBC1 (human)	ATGTCCCAGTTTAAGCGCCAG	CCCAAAGTAGTCATGCAAGCTG
BLM (human)	CAGACTCCGAAGGAAGTTGTATG	TTTGGGGTGGTGTAACAAATGAT
HPRT (human)	CCTGGCGTCGTGATTAGTGAT	AGACGTTCAGTCCTGTCCATAA
Gapdh (mouse)	GGAGAGTGTTTCCTCGTCCC	ACTGTGCCGTTGAATTTGCC
Col1a1(mouse)	GCTCCTCTTAGGGGCCACT	ATTGGGGACCCTTAGGCCAT
Col2A1 (mouse)	TGGTGCTCGGGGTAACGAT	GGCTCCAGGAATACCATCAGT

Ctgf (mouse)	GCGCCTGTTCTAAGACCTGT	TTCATGATCTCGCCATCGGG
Mmp2 (mouse)	ACCTGAACACTTTCTATGGCTG	CTTCCGCATGGTCTCGATG
Mmp9 (mouse)	GCAGAGGCATACTTGTACCG	TGATGTTATGATGGTCCCACTTG
Fn1 (mouse)	AGGAAGCCGAGGTTTTAACTG	AGGACGCTCATAAGTGTCACC
Tgf-β (mouse)	TCAGACATTCGGGAAGCAGT	ACGCCAGGAATTGTTGCTAT
ANF (mouse)	GTGCGGTGTCCAACACAGAT	TCCAATCCTGTCAATCCTACCC
MHC (mouse)	CCTGCGGAAGTCTGAGAAGG	CTCGGGACACGATCTTGGC
Col1a2 (mouse)	CTGGTCCTGTTGGAAGTCGT	CAGATGCACCTGTTTCTCCA
PPIA (mouse)	GAGCTGTTTGCAGACAAAGTTC	CCCTGGCACATGAATCCTGG
actin (mouse)	GTGACGTTGACATCCGTAAAGA	GCCGGACTCATCGTACTCC
Collagen I (mouse)	ACCTTACCAGACTGAGACTCC	TTTGGCTCATTCGATCCTGATACCC

Supplementary Table S2. The list for the primer sequences used in RT-PCR.
